# Asymmetric Synthesis of Trifluoroethyl-Based, Chiral 3-Benzyloxy-1- and -2-Phenyl-quinazolinones of Biomedicinal Interest by Radical Type Cross-Coupling to Olefins

**DOI:** 10.3390/ijms24010513

**Published:** 2022-12-28

**Authors:** Chien-Tien Chen, Yu-Chang Chang, Pin-Xuan Tseng, Chien-I Lein, Shiang-Fu Hung, Hsyueh-Liang Wu

**Affiliations:** 1 Department of Chemistry, National Tsing Hua University, Hsinchu 300044, Taiwan; 2 Department of Chemistry, National Taiwan Normal University, Taipei 11677, Taiwan

**Keywords:** EGFR, asymmetric, cross-coupling, radical, vanadyl complex

## Abstract

Several 2-substituted (H, Ph, and S-Me) and 1-substituted (H, Ph, and Bn), 3-hydroxy-1,3-quinazolin(di)ones were utilized for the first time as radical trapping agents in asymmetric 1,2-oxytrifluoromethylation of styrenes catalyzed by chiral vanadyl methoxide complexes bearing 3,5-disubstituted-*N*-salicylidene-*t*-leucinate templates. The effects of catalysts and solvents on the asymmetric induction were systematically examined. The best and complementary scenarios involved the use of vanadyl complexes **V(O)-1** and **V(O)-2**, which bear 3-(2,5-dimethyl)phenyl-5-bromophenyl and 3-*t*-butyl-5-bromophenyl groups in an *i*-propanol solvent at ambient temperature. The corresponding (*R*)-cross-coupling products by **V(O)-1** were obtained in 45–71% (for 2-substituted series) and 59–93% yields (for 1-substituted series) for *p*-/*m*-methylstyrenes and *m*-halo/CF_3_/CO_2_Me-styrenes in 38–63% ees (the best in 2-H case) and 60–84% ees (the best in 1-benzyl cases), respectively. The corresponding (*S*)-cross-coupling products by **V(O)-2** were obtained in 28–55% (for 2-substituted series) and 45–72% yields (for 1-substituted series) for the same substrate class in 50–91% ees (85–91% ees in 2-phenyl cases) and 64–75% ees (up to 74–75% ees for each 1-H, Ph, and Bn cases), respectively. Theoretical calculations were carried out to explain the origin and extent of enantiocontrols. They both may serve as potential inhibitors of acetohydroxyacid synthase and epidermal growth factor receptor (EGFR) kinases.

## 1. Introduction

**Figure 1:**
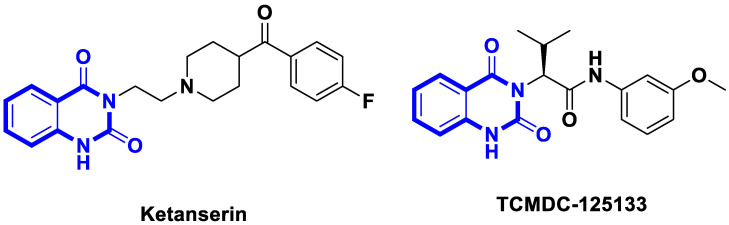
Ketanserin and TCMDC-125133 based on quinazolin-2,4-dione.

Quinazolin-4-ones and -2,4-diones (Quizones) have been well documented as an important template of potential pharmacological and biochemical interests. Relevant studies of **Ketanserin** in these areas have strongly suggested that these derivatives can be used for treating hypertensive symptoms by reducing peripheral vascular resistance by blocking the effect of serotonin functions (Figure 1) [1,2]. In addition, an amide variant TCMDC-125133 bearing a chiral α-Quizone group was found to exhibit effective antimalarial activity against the *Plasmodium falciparum* 3D7 strain (Figure 1) [3,4].

**Figure 2:**
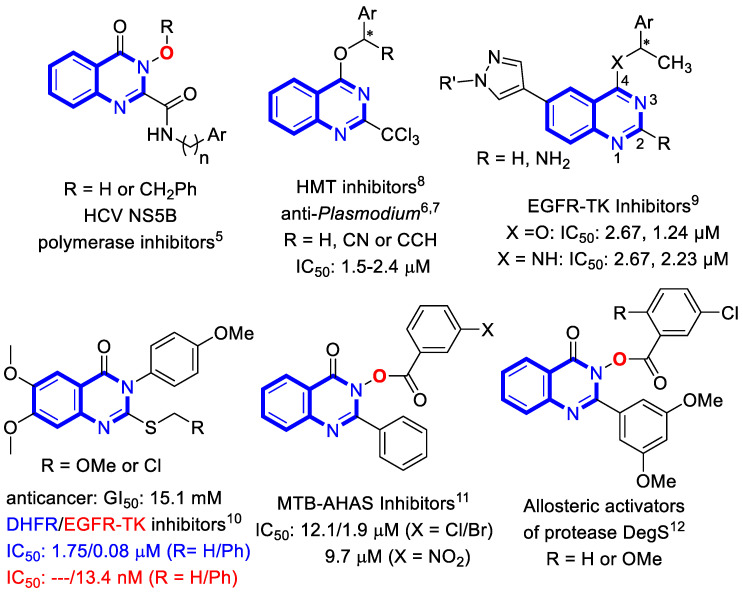
Documented quinazolines bearing either 2-benzylthio, 3/4-benzyloxy, 3-benzoyloxy, or chiral 4-benzyl-oxy/-amino groups as drug candidates. (* stands for chiral center).

In 2012, J.-W. Chern et al. introduced 3-hydroxy/benzyloxy and 2-carbamoyl groups into the bicyclic skeleton of 1,3-quinazolin-4-ones (Quiz) as potential pharmacophores [5]. These derivatives can be used as candidates for HCV NS5B polymerase inhibition. In particular, they serve to block the synthesis of hepatitis C virus RNA, thus inhibiting the replication of the virus (Figure 2). In 2016, P. Verhaeghe, P. Vanelle, et al. introduced 4-benzyloxy/4-amino and 2-trichloromethyl groups into the 1,3-quinazolines [6,7]. The resulting compounds as well as 2-amino analogs [8] may serve as inhibitors for histone (lysine) methyltransferase (HMT). They may thus inhibit the mechanism of G9a during metastasis of cancer cells in vivo. Among 35 different analogs tested, those containing (chiral) 4-benzyl-oxy and -amino groups showed interesting anti-Plasmodium activities [6].

Quinazoline derivatives were found to exhibit good inhibitory activities against the epidermal growth factor receptor (EGFR) by X. Wu, J. Zhang, et al. [9] Notably, certain lung cancer cells tend to evolve with EGFR mutations, which cause their abnormal proliferation and thus accelerate cancer progression. By modifying the 4-arylamino groups to (chiral) 4-benzyl-oxy and -amino groups, it was found that they possessed similar inhibition activities towards EGFR-TKs. Therefore, they may also serve as potential anti-tumor drug candidates.

On the other hand, 4-oxo-3-aryl-2-methyl/benzylthio-Quiz have shown remarkable dihydrofolate reductase (DHFR) and EGFR-TK inhibitions at 0.1–0.9/0.2–1.8 μM (SMe/SBn) and ---/13.4 nM (SMe/SBn) ranges, respectively, indicating the unique role of the 2-mercapto groups [10]. Furthermore, acetohydroxyacid synthase (AHAS) constitutes the first key catalytic step of biosynthesis toward branched-chain amino acids and acts as a potential drug candidate against *Mycobacterium tuberculosis* (MTB). 4-Oxo-2-aryl-3-benzoyloxy-Quiz were identified as promising inhibitors of MTB-AHAS [11]. Serine protease DegS play a key role in the outer membrane stress response pathway for bacteria. It was believed that covalently modifying lysine residues to Serine protease DegS allows for its unique allosteric activation. Similarly, 3-benzoyloxy-quinazoline analogs also may function as allosteric activators for Degs by lysine benzoylation [12].

**Scheme 1:**
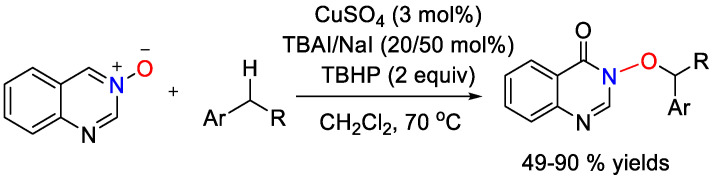
Cu(II)-catalyzed oxidative cross-coupling of quinazoline 3-oxide to benzyl-containing compounds.

Very recently, efficient racemic synthesis of chiral 3-benzyloxy-1,3-quinazoline-4-ones were reported by using Cu(II)/*t*-butyl hydroperoxide combination to catalyze oxidative anchoring of quinazoline-3-oxides to the benzylic positions of various aryl containing compounds (Scheme 1) [13].

**Figure 3:**
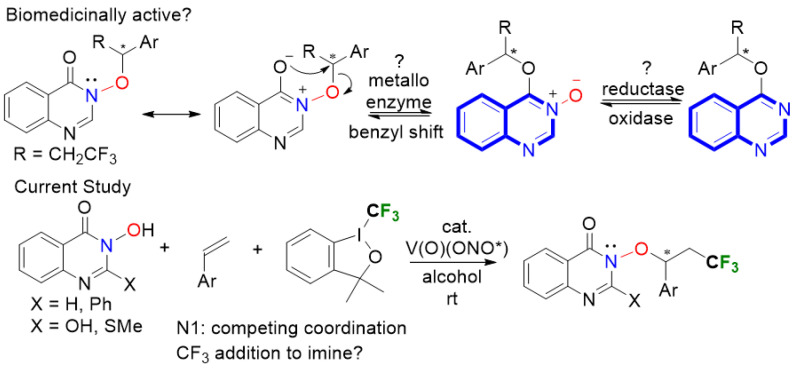
Current study design concept (* stands for chiral center).

In view of the documented 2-H, 2-NH_2_, and 2-carboxyl equivalent (i.e., amide and CCl_3_) groups in the 3-hydroxy-quinazolinone type drug candidates and potential reversible benzyl group shift [from N-O to C(O)], we sought to examine a series of 3OH-Quiz systems bearing 2-H and 2-SMe-groups as a new type of radical trapping agent (Figure 3) based on our previous vanadyl complex-catalyzed 1,2-oxytrifluoromethylation protocol with *N*-hydroxyphthalimide (NHPI) [14,15,16]. The compatibility of the imine N1, 2-hydroxy, and thio units towards incipient radical species during the reaction will be determined. In addition, 2-phenyl-3hydroxy-Quiz was included as a new type of 2-group in drug candidate design. We report herein the preliminary successful results of asymmetric 1,2-oxy-trifluoromethylation. 

## 2. Results

Based on our previous experience, we selected the two best vanadyl complexes, **V(O)-1** and **V(O)-2** bearing 3-(2,5-dimethyl)phenyl-5-bromophenyl (i.e., 3-DMP-5-Br) and 3-(*tert*-butyl)-5-bromophenyl (i.e., 3-*tert*-Bu-5-Br) groups, respectively, as the *N*-salicylidene-*tert*-leucinate templates for the current study in order to attain complementary results in terms of enantiocontrol. 3-Bromostyrene was chosen as the best standard substrate for the asymmetric cross-coupling reactions with these two catalysts in *i*-propanol at ambient temperature. In addition, both 3-methyl and 4-methylstyrenes were examined for comparison.

When the 3-DMP-5-Br vanadyl complex **V(O)-1** was used as the catalyst, the cross-coupling reactions proceeded smoothly in 68 h and 84 h, respectively, with 2-H and 2-Ph-substituted 3OH-Quizs **1** and **2** (entries 1 and 3, Table 1). The corresponding products **8a** and **9a,** enriched in *R*-enantiomers, were isolated in 71 and 45% yields and in 63% and 50% ees, respectively. In the case of 2-SMe substituted 3OH-Quiz **3** (entry 4), the corresponding product, (*R*)-**9a,** was isolated with a 62% yield and in 38% ee in 30 h. Notably, the use of 2-thioxo substituted 3OH-Quiz **7** as the radical trapping agent led to its dramatic decomposition, presumably due to interrupting thio trapping (entry 13). The corresponding desired product **14a** was isolated in only 4% yield and in 60% ee (*R*). In both series, the reaction efficiency, yields, and extent of asymmetric induction decreased with increasing steric sof the 2-substituents (i.e., H was better than Ph and C=S was better than SMe).

In the cases of 2-Ph-3OH-Quiz **2** as the trapping agent with 3-methyl and 4-methyl styrenes as the substrates (entry 2), the reactions proceeded with shorter reaction times of 70 and 52 h (cf 84 h in entry 3), respectively. The corresponding products **9b** and **9c** were obtained at higher yields of 60% and 70% (cf 45% yield in entry 3), but with lower asymmetric inductions of 45% and 26% ees (cf 50% ee in entry 3), respectively.

For a much stricter comparison, 3-hydroxy-1-H- and 1-phenylquinazoline-2,4(1H,3H)-diones **4** and **5** (i.e., 1-H- and 1-Ph-3OH-Quizdiones) were utilized. For 3-Br- and 3-Cl-styrenes (entry 7) with **4**, the reactions went to completion in much shorter reaction time of 43 h/18 h (cf 68 h in entry 1). The corresponding products **11a** and **11a’** furnished much higher yields of 88/83% (cf 71% yield in entry 1), respectively, and improved asymmetric induction of 77/72% ee (cf 63% ee in entry 1), respectively. For the 3-Br-styrene (entry 9) with **5**, the reaction went to completion in a much shorter reaction time of 18 h (cf 84 h in entry 3). The corresponding product **12a** was furnished at a higher yield of 93% (cf 45% yield in entry 3) and improved asymmetric induction of 68% ee (cf 50% ee in entry 3).

A trend similar to that of **4** was observed for styrene (4-H) and 3-methyl-styrene cases (entries 5 and 6). Both of the reactions went to completion in much shorter reaction times of 51 and 38 h (cf 68 h in entry 1), respectively. The corresponding products **11b** and **11c** were isolated at higher yields of 84 and 83% (cf 71% yield in entry 1), respectively, and with comparable to improved asymmetric inductions of 63 and 71% ees (cf 63% ee in entry 1), respectively.

Cross-couplings of **5** with 3-methyl and 4-methyl-styrene cases (entry 8), both the reactions also went to completion in much shorter reaction time of 17 h and 24 h (cf 70 h and 52 h in entry 2), respectively. The corresponding products **12b** and **12c** were isolated at comparable or higher yields of 59/80% (cf 60/70% yields in entry 2), respectively, and with improved asymmetric inductions of 77/48% ee (cf 45/26% ee in entry 3), respectively. In the 3-methylstyrene case, an even better yield (83%) and asymmetric induction (82% ee) can be achieved by carrying out the reaction in EtOH.

Since the uses of 1-H- and 1-Ph-3OH-Quizdiones **4** and **5** as the radical trapping agents led to similar results (cf entries 6–7 and 8–9), 1-benzyl-Quizdione **6** was further synthesized and examined. In the case of 3-bromostyrene (first set of data in entry 10), the corresponding product **13a** was isolated in 91% which was comparable to 93% yield in 1-Ph case (entry 9), albeit with much longer reaction time of 44 h (cf 18 h in entry 9). However, a much better enantioselectivity of 84% ee (*R*) was observed (cf 68% ee in entry 9). Comparable asymmetric induction of 81% ee (*R*) was also achieved with 3-chlorostyrene (second set of data in entry 10). The desired product **13a′** was obtained with an 83% yield in 65 h. Further studies with 3-trifluoromethyl and 3-carbomethoxy-styrenes led to the cross-coupling products **13b** and **13b′** with similarly satisfactory yields (88 and 90%, respectively) and asymmetric induction (82 and 81% ees in entries 11 and 12, respectively). 

Therefore among seven different Quizs and Quizdiones examined, 1-benzyl-Quizdione **6** showed the best results (83–91% yields and 81–84% ees) by 3-DMP-5-Br catalyst **V(O)-1**.

When the 3-*tert*-Bu-5-Br vanadyl complex **V(O)-2** was used as the catalyst (entries 14–23, Table 1), the cross-coupling reactions proceeded somewhat sluggishly in 4–5 days with 2-H and 2-Ph-substituted 3OH-Quizs **1** and **2** (entries 14 and 16). The corresponding products **7a** and **8a,** enriched in complementary *S*-enantiomers were isolated in 31 and 28% yields, respectively, but in comparable or much higher asymmetric induction of 66% and **91%** ees, respectively (cf 63% and 50% ees in entries 1 and 3). In the case of 2-SMe substituted 3OH-Quiz case **3** (entry 17), the corresponding product (*S*)-**9a** was isolated at a lower yield of 39% (cf 62% yield in entry 4) and a comparable asymmetric induction of 50% ee (cf 38% ee in entry 4) in 50 h. Again, the use of 2-thioxo substituted 3OH-Quiz **7** as the radical trapping agent led to its dramatic decomposition presumably due to interrupting thio trapping. No desired product was isolated. In marked contrast, an opposite trend was observed in the former series (i.e., 2-Ph better than 2-H). Therefore, one would expect a unique and advantageous π-π interaction in the former series when catalyzed by 3-*tert*-5-By vanadyl complex **V(O)-2** (vide infra).

In the cases of 2-Ph-3OH-Quiz **2** as the trapping agent with 3-methyl and 4-methyl styrenes as the substrates (entry 15), the reactions also proceeded with a longer reaction time of 4–4.5 days (cf 70/52 h in entry 2). The corresponding products **8b** and **8c** were obtained at lower yields of 31% and 55% (cf 60/70% yields in entry 2), respectively, but with much improved asymmetric inductions of 88 and 85% ees (cf 45/26% ees in entry 2), respectively.

For a fair comparison, 1-H- and 1-Ph-3OH-Quizdiones **4** and **5** were similarly examined. For the 3-Br and 3-Me-styrenes with **4** (entries 18–20), the reaction went to completion in a prolonged reaction time of 5 days (cf 38 and 48 h in entries 6–7, 30 h in entry 4, and 68 h in entry 1). The corresponding products **11a-c** were provided at satisfactory yields of 53–72% (cf 83–88% yields in entries 5–7, 62% yield in entry 4, and 28% yield in entry 16) and comparable to higher asymmetric inductions of 69–75% ees (cf 63–77% ee in entries 5–7, 63% ee in entry 1, and 38% ee in entry 4).

For 3-Me- and 4-Me-styrenes with **5** (entry 21), the reaction went to completion in a prolonged reaction time of 5.4/4 days (cf 17/24 h in entry 8 and 108 h in entry 15), respectively. The corresponding products **12b/21c** were provided in satisfactory yields of 56/64% (cf 31/55% yields in entry 15) and a much lower asymmetric induction of 64/74% ees (cf 88/85% ees in entry 15). A better asymmetric induction of 75% ee can be similarly achieved by performing the reaction in EtOH (cf entries 21 and 8). For 3-Br-styrene with **5** (entry 22), the reaction went to completion in 96 h even in EtOH (cf 18 h in entry 9 and 92 h in entry 16). The corresponding product **11a** was obtained with a better yield of 46% (cf 28% yield in entry 16) but with a lower asymmetric induction of 72% ee (cf 91% ee in entry 16). Evidently, the position and orientation of the phenyl substituent (cf 2-Ph and 1-Ph, entries 16 and 21) play a decisive role in asymmetric induction during the radical trapping events (see below). In comparison, the catalyst **V(O)-2 was used to investigate** 1-benzyl-Quizdione **6** with 3-bromostyrene. The reaction went to completion in 5 days, and the expected product **13a** was isolated in 45% yield and 74% ee (entry 23).

Therefore, among the three Quizzes **1**–**3** examined, Ph-Quiz **2** led to the best asymmetric induction of 85–91% ees. On the other hand, all of three Quizdiones **4**–**6** led to similar levels of asymmetric induction of up to 74–75% ees by the **V(O)-2** catalyst.

## 3. Discussions

**Figure 4:**
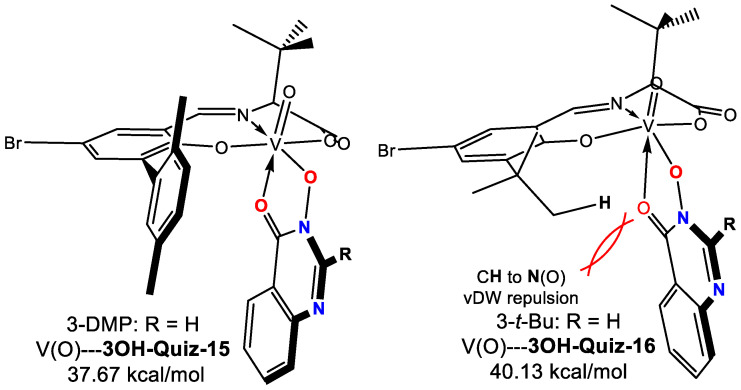
Optimized geometries of vanadyl-bound bidentate 3OH-Quiz-**15** and 3OH-Quiz-**16**.

Based on our previous study using NHPI as the radical trapping agent,^9^ one would expect that 3OH-Quiz (**1**–**3**) and 3OH-Quizdione (**4**–**6**) first undergo facile exchanges with the methoxide and methanol ligands. In the 3OH-Quiz case for both studied vanadyl complexes, they would form the corresponding vanadyl-bound bidentates, 3OH-Quiz-**15** and 3OH-Quiz-**16**, respectively (Figure 4). The relative energies for 3-DMP and 3-*tert*-Bu complexes chelated by **1** by energy minimization were 37.7 and 40.1 kcal/mol, respectively. The higher energy (and thus less stability) in the latter complex-**16** may be attributed to the unfavorable steric interaction between the *tert*-butyl group and the 3OH-Quiz chelates (**1**–**3**) in view of the C-**H** to **N**-O vDW repulsion. This weakness explains why the reaction time was much longer and in a range of 50 h to 4–5 days (cf 30 h to 2–3.5 days by using 3-DMP catalyst). A similar trend was observed in the corresponding 3OH-Quizdione (**4**–**6**) chelates, the reaction time was much longer ranging from 5–5.4 days (cf 17/24 for 1-Ph and 38/51 h for 1-H by using 3-DMP catalyst).

**Figure 5:**
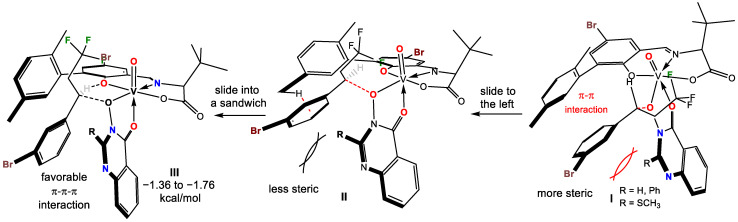
Optimized geometries from the front-**I**, slightly left-**II,** and complete sandwich-**III** orientations of the approaching benzylic radical to the vanadyl bound 3OH-Quiz chelate.

A series of theoretical calculations were carried out by placing the incipient 3-Br-styrene-derived benzylic radical first directly in front of the bound 3OH-Quiz chelate (**I**) followed by a slight shift to the left and proximal to the 3-DMP group with the CF_3_CH_2_ group away from the axial vanadyl (i.e., V=O) unit (**II**), Figure 5. A weak interaction between the benzylic radical and the 3-**O**(N) unit was then introduced. In view of the favorable π-π -π interaction, further slide in between 3-DMP and the bound 3OH-Quiz chelate in a sandwich manner to form **III** is preferred.

**Figure 6:**
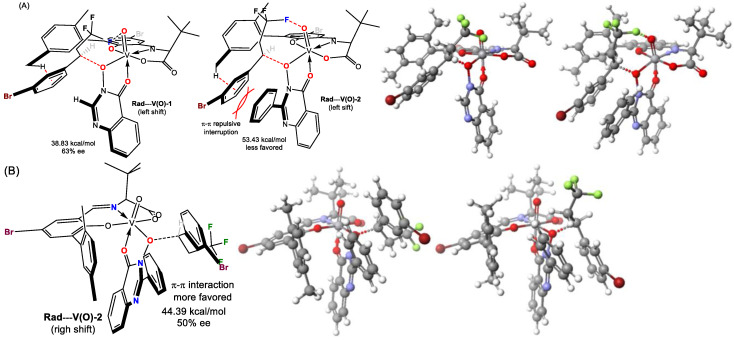
Chem Draw and Scigress 3D presentations for the optimized geometries of the 3-Br-benzylic radical approaching the 2-H and 2-phenyl substituted 3OH-Quiz chelates **Rad---V(O)-1** and **Rad---V(O)-2**: (**A**)-left shift and (**B**)-right shift for the 2-Ph case leading to (*R*) and (*S*) products.

Based on the progressive snap shots obtained by tracing the approaching benzylic radical to the bound 3OH-Quiz chelate, it was found that the 2-phenyl group would encounter much more severe steric repulsion during the sliding progress of the benzylic radical into a sandwich stacking. Therefore, the overall energy of the 2-phenyl system **Rad---V(O)-2 (left shift)** was 14.6 kcal/mol higher than that of the 2-H system **Rad---V(O)-1 (left shift)** (i.e., 53.43 kcal/mol vs 38.83 kcal/mol), Figure 6A. As a result, the approaching radical prefers a slight shift to the right to avoid the steric repulsion of the bound 3OH-Quiz chelate [i.e., **Rad---V(O)-1 (right shift)**]. Under such circumstances, the overall energy of the 2-phenyl system **Rad---V(O)-2 (right shift)** dramatically decreased to 44.39 kcal/mol (Figure 6B) in view of the attractive π-π interaction. Therefore, the enantioselectivity in the 2-phenyl system dropped to 50% ee (*R*) since a competing approach of the incipient radical directly from the right-hand side proximal to the vanadyl carboxyl side would lead to the opposite (*S*)-enantiomer.

**Figure 7:**
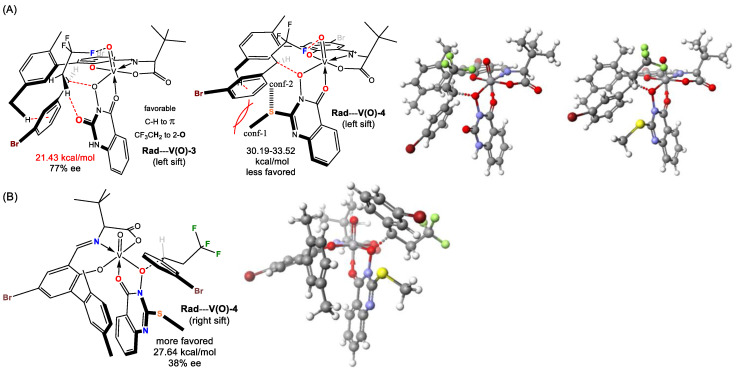
Chem Draw and Scigress 3D presentations for the optimized geometries of the 3-Br-benzylic radical approaching to the 1H-2-C(O) and 2-SMe substituted, 3OH-Quiz chelates **Rad---V(O)-3** and **Rad---V(O)-4**: (**A**)-left shift and (**B**)-right shift for the 2-SMe case.

The best enantiocontrol in the 1-H-2-C(O) system (77% ee *R* in entry 7, Table 1) can be rationalized in terms of its electron-withdrawing nature and its hydrogen-bonding type non-covalent interaction (NCI) with the methylene H of the CH_2_CF_3_ group. The overall energy of the 1-H-2-C(O) system **Rad---V(O)-3** (left shift) (21.43 kcal/mol) was the lowest (Figure 7A).

In contrast, the worst enantiocontrol in the 2-SMe system can be explained in terms of the unfavorable steric repulsion between the approaching benzylic radical and the methylthio group, particularly in conformation-2 (33.52 kcal/mol) in **Rad---V(O)-4 (left shift)**, Figure 7A. As a result, the approaching radical would prefer to slightly shift to the right to avoid the steric repulsion to the bound 3OH-Quiz chelate [i.e., **Rad---V(O)-4 (right shift)**]. Under such circumstances, the overall energy of the 2-SMe system significantly decreased to 27.64 kcal/mol (Figure 7B) in view of the attractive π-π interaction. Therefore, the enantioselectivity in the 2-SMe system dropped to only 38% ee (*R*) since a similar approach from the right leading to the opposite (*S*)-enantiomer would also compete.

**Figure 8:**
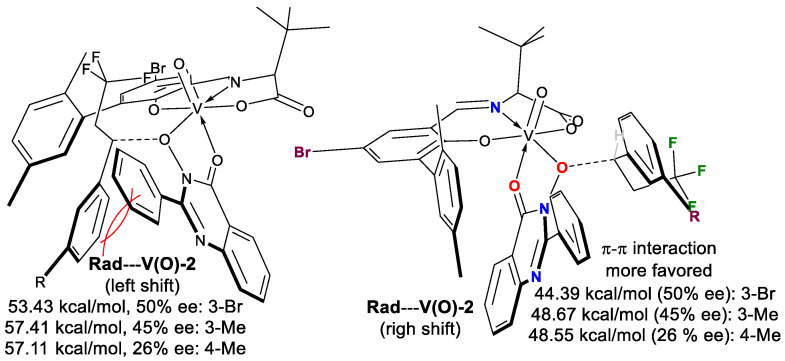
Optimized geometries of 3-Br- and 3-Me/4-Me benzylic radicals approaching to the 2-Ph substituted 3OH-Quiz chelates with the left shift and the right shift.

The significant drop in enantioselectivities in the 2-phenyl systems caused by switching the substrate to 3-methyl and 4-methylstyrenes can be envisioned in terms of the increasing steric repulsions between the 3-Me/4-Me groups and the 5-methyl group in DMP during the initial front approach en route to their left or right shift (Figure 8).

**Figure 9:**
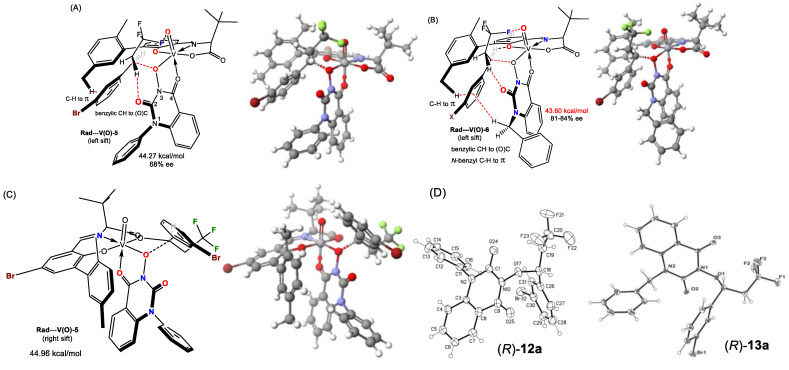
Chem Draw and Scigress 3D presentations for the optimized geometries of 3-Br-benzylic radicals approaching to the 1-Ph-2-oxo and 1-benzyl-2-oxo substituted 3OH-Quizone chelates with left shift (**A**) and right shift (**B**). The 1-Ph-2-oxo substituted 3OH-Quizone case with the right shift (**C**). Ortep drawings of enantiomerically pure (*R*)-**12a** and **13a** at 50% probability (**D**).

The higher enantioselectivity of **11a** (cf 68% ee *R* in entry 9 and 50% ee in entry 3) observed in the 1-Ph-2-oxo system **Rad---V(O)-5** (left shift) can also be rationalized in view of the favorable H-bonding type NCI and absence of steric repulsion between the 1-phenyl group and the 3-Br-phenyl group in the benzyl radical (Figure 9A). Alternatively, **Rad---V(O)-5** (right shift) can also be operative with similar enantio-control in view of its similar overall energy (Figure 9C). The even better asymmetric induction in **13a** (84% ee in entry 10) may be visualized by the **Rad---V(O)-6** (left shift) interaction model (Figure 9B) but with an additional *N*-benzyl CH for π interaction. Both the absolute stereochemistries of the corresponding products **12a** and **13a** were confirmed to be (*R*) by X-ray crystallographic analyses of their recrystallized products in almost enantiomerically pure forms (Figure 9D). The selected crystal data and structure refinement for (*R*)-**12a** and **13a** are included as Table S1 and the first paragraph on page S34. Their Platon drawings are included as Figures S1 and S2).

**Figure 10:**
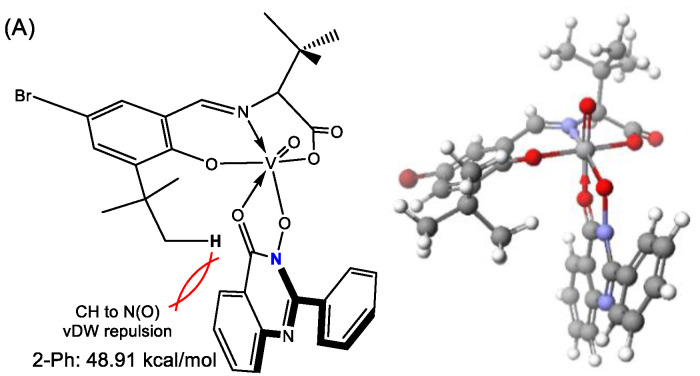

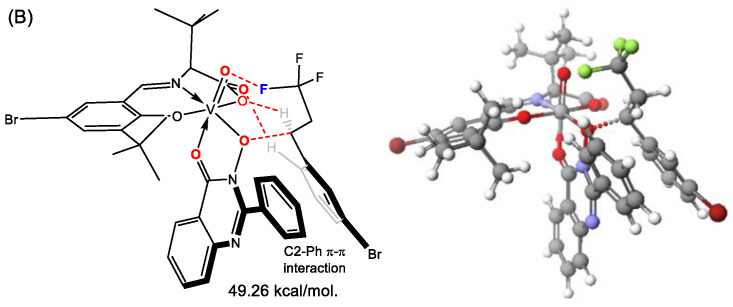
Chem Draw and Scigress 3D presentations for the optimized geometries of the vanadyl bound 2-phenyl-3OH-Quiz (**A**) and its corresponding adduct with the 3-Br-benzylic radical ap-proaching directly from the right proximal to the carboxyl moiety (**B**).

In the 3-*tert*-butyl-5-Br vanadyl catalyst **V(O)-2** system, the best result observed in the 2-phenyl case (91% ee in entry 13) can be rationalized in terms of the favorable π-π interaction between the 2-phenyl group and the 3-Br-phenyl ring in the benzylic radical as shown in the optimized geometries of the catalyst (Figure 10A) and the interaction of the vanadyl-bound 2-Ph-Quiz with the benzylic radical (Figure 10B). The significant drop in enantio-selectivity in the 1-phenyl-2C(O) case (56% ee) can thus be understood to be due to the lack of π-π interaction. The lower asymmetric induction in 2-SMe (50% ee in entry14) also supports the decisive π-π interaction in the 2-phenyl case. In addition, a slightly better asymmetric induction in 1-Ph-2-C(O) system with catalyst **V(O)-1** (cf 68% ee and 56% ee in entries 10 and 18) supports a slightly better π-π interaction in **Rad---V(O)1-5** as compared to **Rad---V(O)2-5** (cf Figure 9A and Figure 10B). A similar range of asymmetric inductions in 1-H-2-C(O) system for both **V(O)-1** and **V(O)-2** catalysts (cf 63–77% ees and 69–75% ees) also supports a participating role for π-π interaction in the 1-Ph-2-C(O) system (i.e., entries 10 and 18).

## 4. Materials and Methods

### 4.1. General

Mass analyses were obtained by the Department of Chemistry, National Tsing Hua University, or the Department of Photonics, National Chiao Tung University, Taiwan. Filed-ionization (FI) and electrospray ionization (ESI) mass spectra were recorded, with data reported in the form m/e (intensity relative to the base peak). Analytical high pressure liquid chromatography (HPLC) was performed with a built-in photometric detector (λ = 254 nm) using Diacel Chiralpak AD-H and AS-H (0.46 cm × 25 cm) columns. Solvents for HPLC analyses were of spectroscopic grade and filtered before use. Enantiomeric excess determinations for optically enriched products were correlated with the corresponding racemic samples by HPLC analyses on chiral columns. Optical rotations are reported as follows: $[\alpha {]}_{D}^{T}$ (c = g/100 mL, solvent). Reaction products were isolated as chromatographically pure materials. Olefins were passed through basic alumina to remove the radical inhibitor BHT before use. Catalysts were synthesized according to our reported procedures [8].

### 4.2. Representative Synthesis of 12b by Catalyst V(O)-1

An oven dried reaction tube (1.5 cm OD × 15.0 cm height) was filled with V(O)-1 (10.3 mg, 0.02 mmol, 5 mol%) and 5 (101.7 mg, 0.4 mmol, 1.0 equiv) in anhydrous *i*-PrOH (degassed by Ar, 2 mL), followed by the addition of 3-methylstyrene (74.48 μL, 0.56 mmol, 1.4 equiv) through a microsyringe under Ar atmosphere. A solution of Togni reagent (224.5 mg, 0.68 mmol, 1.7 equiv) in anhydrous *i*-PrOH (1 + 1 mL) was added. The resulting reaction mixture was stirred at ambient temperature for 17 h and concentrated by rotatory evaporation. The crude mixture was purified by flash column chromatography (EtOAc/ hexanes, 1/6) on silica gel to give 104.2 mg (59%) of (*R*)-12b as a white solid in 77% ee. A similar procedure in EtOH led to the corresponding product (*R*)-12b in 83% yield and 82% ee.

### 4.3. Representative Synthesis of 9a-9c by Catalyst V(O)-2

An oven dried reaction tube (1.5 cm OD × 15.0 cm height) was filled with V(O)-2 (9.3 mg, 0.02 mmol, 5 mol%) and 2-Ph-Quiz 2 (95.5 mg, 0.4 mmol, 1.0 equiv) in anhydrous *i*-PrOH (degassed by Ar, 2 mL), followed by the addition of 3-bromostyrene (73 μL, 0.56 mmol, 1.4 equiv) through a microsyringe under Ar atmosphere. A solution of Togni reagent (224.6 mg, 0.68 mmol, 1.7 equiv) in anhydrous *i*-PrOH (1 + 1 mL) was added. The resulting reaction mixture was stirred at ambient temperature for 92 h and concentrated by rotatory evaporation. The crude mixture was purified by flash column chromatography (EtOAc/ hexanes, 1/10) on silica gel to give 54.8 mg (28%) of (*S*)-9a as a white solid in 91% ee. Similar procedures with 3-methyl- and 4-methyl-styrenes led to the corresponding products (*S*)-9b and (*S*)-9c in 31% and 55% isolated yields in 88% and 85% ees, respectively.

### 4.4. Theoretical Calculation

Optimized geometry for the structure of a chemical sample corresponds to an energy minimum using the Scigress program. The structure of the chemical sample was refined by performing an optimize geometry calculation in Mechanics using Augmented MM3 parameters. The sandwich structure optimizations were performed with an optimize geometry calculation in Mechanics using Augmented MM2 parameters.

## 5. Conclusions

We have documented a new type of asymmetric 1,2-oxytrifluoromethylation of styrenes catalyzed by our two vanadyl complexes **V(O)-1** (3-DMP) and **V(O)-2** (3-*t*-Bu), in a complementary manner with various 1- and 2-substituted-3-hydroxy-1,3-quinazolin(ones) [(3(OH)Quiz(one)] as radical trapping agents. The imine and carbamate units in these substrates were confirmed to be compatible with CF_3_ radicals and benzylic radicals. Among three different 2-substituted-(3(OH)Quizs (i.e., H, Ph, and SMe) examined in *i*-propanol except the 2-phenyl case by the catalyst **V(O)-2**, the extents of asymmetric induction decreased with increasing sterics of the 2-substituents [i.e., 2-H (63/66% ee) > 2-Ph (45/50% ees) > 2-SMe (38/50% ees)]. The corresponding (*R*)- and (*S*)-cross-coupling products were obtained in up to 71% and 55% yields for *p*-/*m*-methylstyrenes and *m*-bromostyrenes in up to (*R*)-63% ee (in 2-H case) and (*S*)-91% ee (in 2-Ph case by catalyst **V(O)-2**), respectively. Among three different 1-substituted-3(OH)Quiz-2-ones (i.e., H, Ph, and Bn) examined, 1- Bn-Quiz-2-one **6** showed the best results (83–91% yields and 81–84% ees) by the 3-DMP-5-Br catalyst **V(O)-1**. In marked contrast, the results from catalysts **V(O)-1** and **V(O)-2** by using 3-hydroxy-1-H and 3-hydroxy-1-phenylquinazoline-2,4-diones as the trapping agents were found to be less enantioselective. The best results in the *m*-bromo-products were obtained in 88/93% and 53/46% yields and in 77/68% ees (*R*) and 74/72% ees (*S*), respectively, despite with some improvement in EtOH.

Theoretical calculations were performed to rationalize the experimental trends. In the former case, the incipient benzylic radical after CF_3_ radical addition to a given styrene substrate approaches the vanadyl-bound [(3OH)Quiz(one)] from the side proximal to the 3-DMP group in the catalyst in a preferred sandwich type DMP (C-H)-π-π interaction. In contrast, the benzylic radical approaches [V(O)-(3OH)Quiz(one)] from the side opposite to the sterically demanding 3-*tert*-butyl group, thus leading to a complementary enantiocontrol. In the 2-phenyl-(3OH)Quiz case, the steric interaction of the two interrupting π-π systems between the incipient 3-Br-benzylic radical and the vanadyl-bound 2-phenyl-[V(O)-(3OH)Quiz] was identified to be responsible for the decreased enantioselectivities to (*R*)-50% ee by using a 3-DMP5-Br based catalyst. In contrast, the best enantioselectivities of (*S*)-85–91% ees were achieved using the **V(O)-2** catalyst in terms of favorable π-π interactions with its approach from the right hand side. Conversely in the 1-H-2-C(O) system, the asymmetric inductions were similar in up to 75–77% ees with both catalysts in view of the lack of 2-Ph or 1-Ph group. Therefore, the 2-Ph- and 1-Ph/Bn -2-C(O)-(3OH)Quiz(one)s serve as the best radical trapping agents, which bode well for their potential applications as key intermediates for acetohydroxyacid synthase and EGFR kinase inhibitors. Either enantiomeric products and racemic mixtures will be subjected to the sequences of enzymatic and/or biomedicinal tests to gain insight into the effect of absolute chirality to biomedical activities (see Table S2 for their preliminary single point analyses).

## Figures and Tables

**Table 1:**
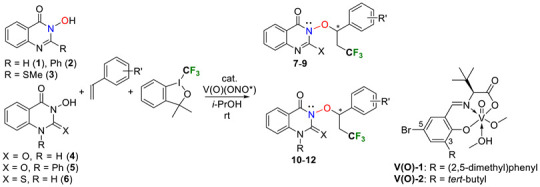
CF_3_-Directed asymmetric cross-coupling of 2- and 1-substituted 3-hydroxy-1,3-quinazolin(di)ones to styrenes catalyzed by vanadyl complexes (* stands for chiral center).

			Catalyst: 3-(2,5-dimethyl)phenyl-5-Br V(O)-1		
Entry	R	R′	Time, h	Yield, *^a^* %	ee, *^b,d^* %
1	2-H	3-Br	68	71 (**8a**)	63 (*R*)
2	2-Ph	3/4-Me	70/52	60/70 (**9b**/**9c**)	45/26 (*R*)
3	2-Ph	3-Br	84	45 (**9a**)	50 (*R*)
4	2-SMe	3-Br	30	62 (**10a**)	38 (*R*)
5	1-H (X = O)	4-H	51	83 (**11c**)	71 (*R*)
6	1-H (X = O)	3-Me	38	84 (**11b**)	63 (*R*)
7	1-H (X = O)	3-Br/3-Cl	43/18	88/83 (**11a**/**11a′**)	77/72 (*R*)
8	1-Ph (X = O)	3/4-Me	17 (66*^c^*)/24	59 (83*^c^*)/80 (**12b**/**12c**)	77 (82*^c^*)/48 (*R*)
9	1-Ph (X = O)	3-Br	18	93 (**12a**)	68 (*R*)
10	1-CH_2_Ph (X = O)	3-Br/3-Cl	44/65	91/83 (**13a**/**13a′**)	84/81 (*R*)
11	1-CH_2_Ph (X = O)	3-CF_3_	46	88 (**13b**)	82 (*R*)
12	1-CH_2_Ph (X = O)	3-CO_2_Me	40	90 (**13b′**)	81 (*R*)
13	1-H (X = S)	3-Br	48	4% (**14a**)	60 (*R*)
			catalyst: 3-*tert*-Bu-5-Br **V(O)-2**		
14	2-H	3-Br	120	31 (**8a**)	66 (*S*)
15	2-Ph	3/4-Me	108/92	31/55 (**9b**/**9c**)	88/85 (*S*)
16	2-Ph	3-Br	92	28 (**9a**)	91 (*S*)
17	2-SMe	3-Br	50	39 (**10a**)	50 (*S*)
18	1-H (X = O)	H	120	72 (**11c**)	75 (*S*)
19	1-H (X = O)	3-Me	48	54 (**11b**)	69 (*S*)
20	1-H (X = O)	3-Br	120	53 (**11a**)	74 (*S*)
21	1-Ph (X = O)	3/4-Me	130 (24 *^c^*)/96	56 (54 *^c^*)/64 *^c^* (**12b**/**12c**)	64 (75 *^c^*)/74 *^c^* (*S*)
22	1-Ph (X = O)	3-Br	96 *^c^*	46 *^c^* (**12a**)	72 *^c^* (*S*)
23	1-CH_2_Ph (X = O)	3-Br	120	45 (**13a**)	74 (*S*)

*^a^* Isolated yields. *^b^* Determined by HPLC analysis on Chiralpak AD-H or AS-H. *^c^* Performed in EtOH. *^d^* Racemic products were made by the reactions catalyzed by V(O)(acac)_2_ or by racemic **V(O)-1**.

## Data Availability

Not applicable.

## Supplementary Materials

The following supporting information can be downloaded at: https://www.mdpi.com/article/10.3390/ijms24010513/s1.
