# Supplementary material for: Asymmetric Synthesis of Trifluoroethyl-Based, Chiral 3-Benzyloxy-1- and -2-Phenyl-quinazolinones of Biomedicinal Interest by Radical Type Cross-Coupling to Olefins
Source: Int J Mol Sci. 2022 Dec 28;24(1):513. doi: 10.3390/ijms24010513 (PMC9820497; doi:10.3390/ijms24010513)
Supplement: Supplementary file 1 [file ijms-24-00513-s001.zip › ijms-2028159-supplementary.pdf]

Asymmetric Synthesis of Trifluoroethyl-based, Chiral 3-Benzoxy-1- and -2-Phenyl-quinazolinones of Biomedicinal Interests by Radical Type Cross Coupling to Olefins

Chien-Tien Chen,*[1] Yu-Chang Chang,[1] Pin-Xuan Tseng,[1] Chien-I Lein,[1] Shiang-Fu Hung,[1] and Hsyueh-Liang Wu*[2]

[1]Department of Chemistry, National Tsing Hua University, Taiwan, ROC

[2]Department of Chemistry, National Taiwan Normal University, Taiwan, ROC

e-mail: ctchen@mx.nthuedu.tw[1]; hlw@ntnu.edu.tw[2]

Supporting Information

Table of content

Preparation of radical trapping agents **1-3**..S2-S5

Preparation of radical trapping agents **4-7**..S5-S10

Representative catalytic procedures, Characterization Data, and HPLC plots for **8-10**… …...S11-S18

Procedures, Characterization Data, and HPLC plots for **11-14**……………………………..S18-S31

Ortep drawings and selected X-ray data for (*R*)-**12a** and (*R*)-**13a**………….. ……………....S31-S33

EGFR-TK inhibition results……………………………………………………………...S33-S35

Experimental References………………………………………………………………S35-S36

Computational details and Cartesian coordinates……………………………………...S37-S73

Scanned spectra……………………………………………………………..……...S74-S97

Experimental procedures for the preparation of 3-hydroxy-quinazolin(di)ones as radical tarpping agents

2-Amino-*N*-(benzyloxy)benzamide[1a]

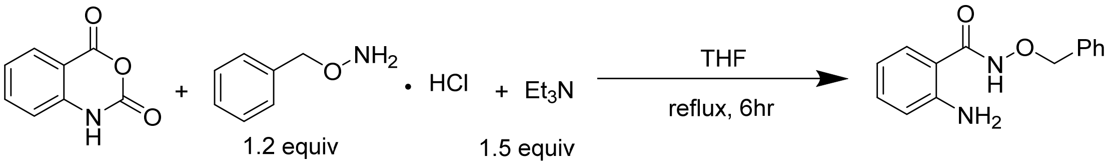

In a 100-mL three-necked flask was placed *N*-carboxyaminobenzenecarboxylic anhydride (1.63 g, 10.0 mmol) and *O*-benzylhydroxylamine hydrochloride (1.93 g, 12.0 mmol, 1.2 equiv) in anhydrous THF (20 mL). Triethylamine (2.1 mL, 15.0 mmol, 1.5 equiv) was then added. The resulting mixture was stirred under reflux for 18 h and the quenched with sat. aqueous NH_4Cl solution (20 mL) at ambient temperature. The reaction mixture was extracted with ethyl acetate (20 mL × 3). The combined organic layer was dried over $MgSO_4$ (1.5 g), filtered, and evaporated. The crude product was purified by column chromatography on silica gel (ethyl acetate/hexanes, 1/3) to obtain 1.99 g (82% yield) of 2-mino-*N*-(benzyloxy)benzamide: ^{1}H NMR (400 MHz, DMSO-d_6) δ 11.44 (s, 1H), 7.48-7.30 (m, 6H), 7.16 (td, *J* = 7.8, 1.4 Hz, 1H), 6,73 (dd, *J* = 8.2, 1.0 Hz), 6.49 (td, *J* = 7.8, 1.0 Hz), 6.30 (s, 2H), 4.90 (s, 2H); ^{13}C NMR (100 MHz, DMSO-d_6) δ 167.7, 150.1, 136.6, 132.6, 129.3, 128.8, 128.7, 128.2, 116.8, 115.1, 112.8, 77.4; TLC R_f 0.25 (EtOAc/hexanes, 1/3); HRMS (FD) $[M]^+$ Calcd for $C_{14}H_{14}N_2O_2$: 242.1050, found: 242.1051.

3-Hydroxyquinazolin-4(3H)-one 1[1a,b]

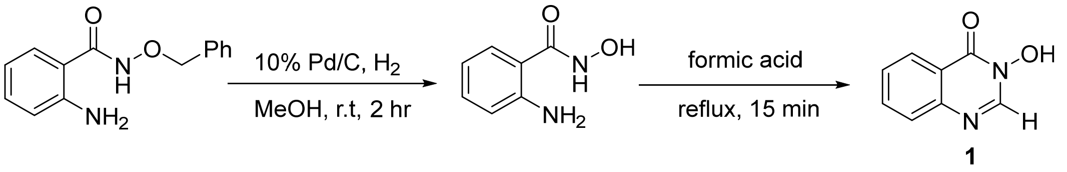

To a 100-mL double-necked flask was placed 2-amino-*N*-(benzyloxy)benzamide (1.34 g, 5.5 mmol) in methanol (50 mL). Ten percent Pd/C catalyst (52.3 mg) was then added. The reaction mixture was degassed with argon and then introduced hydrogen gas for 2 min. The reaction flask was kept

under a hydrogen gas balloon at ambient temperature for 2 h with stirring with complete reaction as evidenced by TLC analysis. The reaction mixture was passed through a short plug of Celite and the filtrate was concentrated under reduced pressure to provide 743.2 mg of crude 2-amino-*N*-hydroxybenzamide. The crude product was placed in a 25-mL double-necked flask in formic acid (3 mL). The reaction mixture was stirred under reflux for 15 min. Deionized water (15 mL) was then added and the reaction mixture was kept boiling for another 15 min and gradually cooled to ambient temperature to induce precipitation of the product. The solid was collected by filtration through a sintered glass funnel and rinsed with deionized water (20 mL). The wet crude product was recrystallized from acetone. (30 mL) to give 394.6 mg of **1** in 44% yield: ^{1}H NMR (400 MHz, DMSO-d_6) δ 11.91 (s, 1H), 8.54 (s, 1H), 8.18 (d, *J* = 8.0 Hz, 1H), 7.83 (td, *J* = 8.2, 1.2 Hz, 1H), 7.72 (d, *J* = 8.2 Hz, 1H), 7.57 (td, *J* = 8.0, 1.2 Hz, 1H); ^{13}C NMR (100 MHz, DMSO-d_6) δ 157.3, 146.9, 145.9, 134.0, 127.5, 127.0, 125.9, 122.5; TLC R*f* 0.19 (Hexanes/EtOAc, 1/3); HRMS (FI) $[M]^+$ Calcd for $C_8H_6N_2O_2$: 162.0424, found: 162.0425.

3-(Benzyloxy)-2-phenyl-quinazolin-4(3H)-one[2]

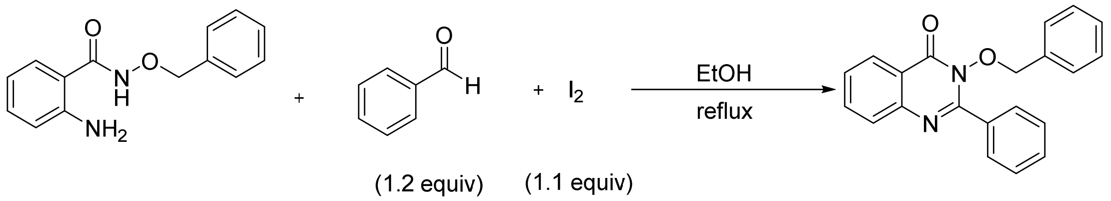

In a 100-mL three-necked flask was placed 2-amino-*N*-benzyloxy-benzamide (2.18 g, 9.0 mmol) and dissolve it in ethanol (30 mL). Iodine (2.51 g, 9.9 mmol, 1.1 equiv) was added and benzaldehyde (1.1 mL, 10.8 mmol, 1.2 equiv) was injected. The reaction mixture was stirred at ambient temperature for 2 h and then quenched by adding sat. aqueous sodium thiosulfate (9 mL). The reaction mixture was extracted with ethyl acetate (25 mL×3). The combined organic layer dried over $MgSO_4$ (1.5 g), filtered, and evaporated. The crude residue was purified by column chromatography on silica gel

(EtOAc/hexanes, 1/8) to give 1.48 g (50% yield) of 3-(benzyloxy)-2-phenylquinazolin-4(3H)-one: ^{1}H NMR (400 MHz, DMSO-d$_6$) δ 8.25 (dd, *J* = 7.9, Hz, 1H), 7.90 (t, *J* = 7.6 Hz, 1H), 7.76 (d, *J* = 7.3 Hz, 3H), 7.62 (q, *J* = 6.8 Hz, 2H), 7.53 (t, *J* = 7.7 Hz, 2H), 7.32 (t, *J* = 7.2 Hz, 1H), 7.25 (t, *J* = 7.4 Hz, 2H), 6,94 (d, *J* = 7.4 Hz, 2H), 4.93 (s, 2H); ^{13}C NMR (100 MHz, DMSO-d$_6$) δ 157.3, 153.7, 146.1, 134.6, 133.1, 132.3, 130.4, 129.5, 129.3, 129.0, 128.3, 127.9, 127.7, 127.1, 126.2, 122.4, 77.7; TLC R$_f$0.17 (EtOAc/hexanes, 1/8); HRMS (FD) [M]$^+$ Calcd for $C_{21}H_{16}N_2O_2$:328.1217, found:328.1226.

3-Hydroxy-2-phenyl-quinazolin-4(3H)-one 2[3]

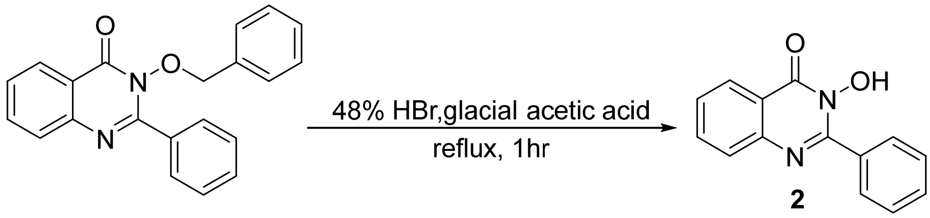

In a 100-mL double-necked flask was placed 3-(benzyloxy)-2-phenylquinazolin-4(3H)-one (328.4 mg, 1.0 mmol) in glacial acetic acid (2 mL). The reaction was heated to reflux with stirring for 3 min to reach a homogeneous state and 48% hydrobromic acid (2 mL) was added. After having been stirred for1 h, the reaction mixture was cooled to ambient temperature. Deionized water (20 mL) was added to quench the reaction and to induce precipitation of the product. The solid was collected by passing thru a sintered glass and the rinsed with deionized water (30 mL). The resulting wet crude product was recrystallized from acetone (10 mL) to obtain 162.0 mg (68%) of 2 as a white solid: ^{1}H NMR (400 MHz, DMSO-d$_6$) δ 11.67 (s, 1H) 8.20 (dd, *J* = 8.0, 1.1 Hz, 1H), 7.87-7.81 (m, 3H), 7.74 (d, *J* = 8.0 Hz, 1H), 7.60-7.47 (m, 4H); ^{13}C NMR (100 MHz, DMSO-d$_6$) δ 158.3, 153.3, 146.2, 134.2, 132.8, 130.3, 129.4, 127.9, 127.5, 126.8, 125.9, 121.4; TLC R$_f$0.77 (EtOAc/hexanes, 1/1); HRMS (FD) [M]$^+$ Calcd for $C_{14}H_{10}N_2O_2$:238.0748, found:238.0741.

3-Hydroxy-2-(methylthio)quinazolin-4(3H)-one 3[4]

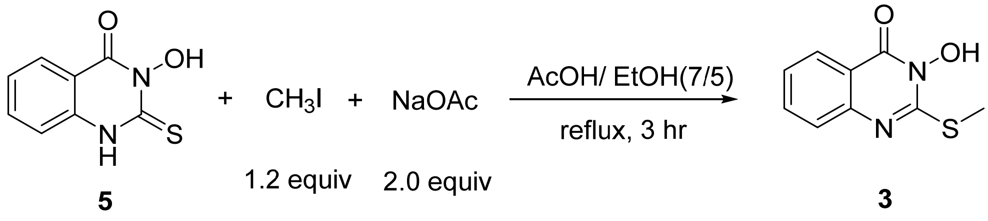

To a 50-mL double-necked flask was placed **5** (972.3 mg, 5.0 mmol) and anhydrous sodium acetate (828.2 mg, 10.0 mmol, 2.0 equiv) in glacial acetic acid/ethanol (7/5) mixed solvent (12 mL). The reaction mixture was heated to reflux till completely homogeneous and then returned to ambient temperature. Iodomethane (382.2 μL, 6.0 mmol, 1.2 equiv) was added and the resulting reaction mixture was stirred under reflux for 3 h. The reaction mixture was gradually cooled to ambient temperature and concentrated to remove EtOH by rotatory evaporation. Deionized water (30 mL) was added to induce product precipitation. The crude product was washed with deionized water (30 mL) and the wet solid was recrystallized from acetone (40 mL) to give 994.2 mg (96% yield) of **3** as a white solid: ^{1}H NMR (400 MHz, DMSO-d$_6$) δ 12.11 (s, 1H), 8.08 (d, *J* = 7.6 Hz, 1H), 7.78 (t, *J* = 7.6 Hz, 1H), 7.59 (d, *J* = 8.2 Hz, 1H), 7.45 (t, *J* = 7.6 Hz, 1H), 2.52 (s, 3H); ^{13}C NMR (100 MHz, DMSO-d$_6$) δ 157.8, 157.3, 146.3, 134.2, 126.1, 126.1, 125.5, 119.9, 13.2; TLC R$_f$ 0.38 (MeOH/EtOAc, 1/10); HRMS (FD) $[M]^+$ Calcd for $C_9H_8N_2O_2S$: 208.0301, found: 208.0303.

3-(Benzyloxy)quinazoline-2,4(1H,3H)-dione[2]

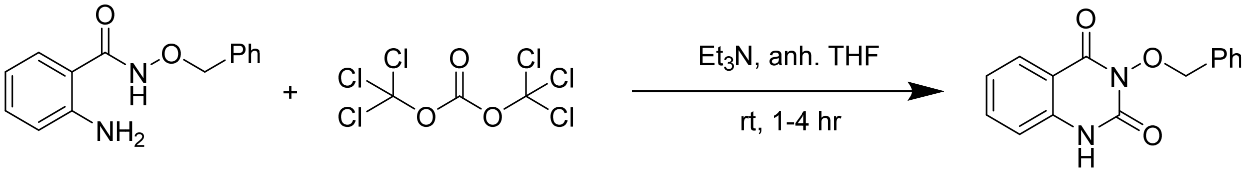

In a 50-mL, two-necked, round-bottomed flask was placed 2-amino-*N*-benzyloxy-benzamide (726.8 mg, 3.0 mmol) and triphosgene (356.1mg, 1.2 mmol, 0.4 equiv) in anhydrous tetrahydrofuran (30 mL). Triethylamine (1.04 mL, 7.5 mol, 2.5 equiv) was added dropwise. The resulting reaction mixture was stirred at ambient temperature for 1h and then quenched with H_2O (80 mL). The resulting

white solid was collected and then washed consecutively with H_2O (2 × 250 mL) and acetone (50 mL). The wet solid was dried under vacuum to give 579.5 mg (72% yield) of ***N*-Bn-QuizOn** as a white solid: ^{1}H NMR (400 MHz, d6-DMSO) δ 11.66 (s, 1H), 7.96 (d, *J* = 7.8 Hz, 1H), 7.68 (t, *J* = 7.8 Hz, 1H), 7.57 (d, *J* = 6.6 Hz, 2H), 7.41 (d, *J* = 6.6 Hz, 3H), 7.26-7.20 (m, 2H), 5.10 (s, 2H); ^{13}C NMR (100 MHz, d6-DMSO) δ 159.0, 148.0, 138.7, 135.0, 134.5, 129.4, 128.8, 128.3, 127.2, 122.6, 115.4, 114.4, 77.5; R_f 0.26 (EtOAc/hexanes, 1/2); HRMS (FD) Calcd for $C_{15}H_{12}N_2O_3$: 268.0842, found: 268.0846.

3-Hydroxyquinazoline-2,4(1H,3H)-dione 4[1a,3]

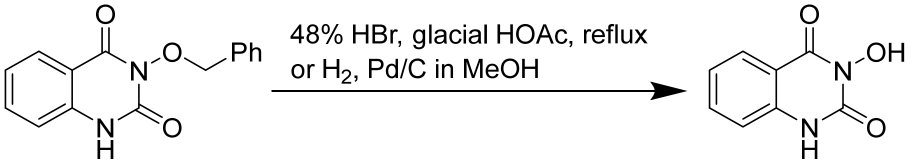

In a 25-mL, two-necked, round-bottomed flask was placed *N*-Bn-QuizOn (804.8 mg, 3.0 mmol) and hydrobromic acid (48%, 6 mL) in glacial acetic acid (6 mL). the resulting reaction mixture was heated to reflux for 4-5 h. The resulting solution was gradually cooled to ambient temperature and then quenched with water (50 mL). The resulting precipitate was collected by filtration and the solid was washed with water (10 mL). The wet solid was dried in vacuo and then recrystallized from MeOH (? mL) to give 272 mg (51% yield) of *N*-OH-**QuizOn** as a white solid:

In a 250-mL, round-bottomed flask was placed *N*-Bn-**QuizOn** (804.8 mg, 3.0 mmol) and Pd/C (10 wt% loading, 15.9 mg, 5 mol%) in MeOH (90 mL). The reaction flask was then topped with a H_2 gas balloon. After three cycles of rotavap. evacuation/H_2-gas saturation, the reaction mixture was kept stiiring under H2 at ambient temperature for1h. The reaction mixture was then passed through a shot plug of Celite (2.8 g) and the Celite pad was washed with MeOH (? mL). The combined filtrates were concentrated by rotary evaporation to give 375 mg (70% yield) of *N*-OH-**QuizOn** as a white solid: ^{1}H NMR (400 MHz, d6-DMSO) δ 11.55 (s, 1H), 10.58 (s, 1H), 7.94 (dd, *J* = 8.2, 1.0 Hz, 1H), 7.65 (td, *J*

= 7.8, 1.4 Hz, 1H), 7.24-7.18 (m, 2H); ^{13}C NMR (100 MHz, d_6-DMSO) δ 159.4, 148.7, 138.3, 134.7, 127.1, 122.5, 115.3, 114.1; R_f 0.33 (CH_3OH/CH_2Cl_2, 1/6).

N-(Benzyloxy)-2-(phenylamino)benzamide[4a]

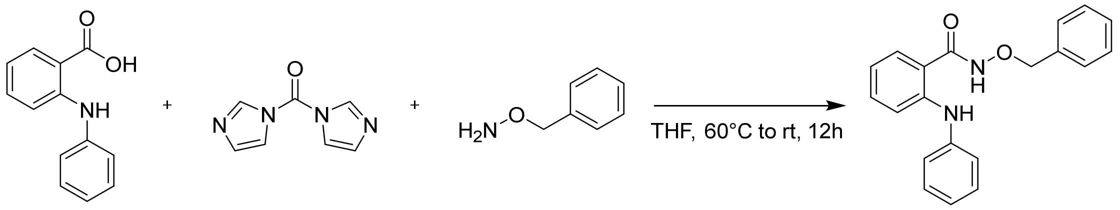

In a 100-mL double-necked bottle was placed Fenamic acid (2.1 g, 10 mmol) in anhydrous THF (15 mL) followed by addition of 1,1'-carbonyldiimidazole (1.8 g, 11 mL, 1.1 equiv). The reaction mixture was stirred at 60 °C for 20 min after which *O*-benzylhydroxylamine (1.5 g, 12 mmol, 1.2 equiv) was added through syringe. The reaction mixture was stirred at ambient temperature for 12 h and then concentrated. The resulting residue was treated with 1*N* aqueous hydrochloric acid (15 mL) and then extracted with ether (20 mL × 3). The combined organic layer was washed with sat. aqueous $NaHCO_3$ (15 mL×3), dried (MgSO4), filtered, evaporated. The crude product was recrystallized from ether/*n*-hexane mixture to obtain 1.7 g (53% yield) of *N*-(Benzyloxy)-2-(phenylamino)benzamide: ^{1}H NMR (400 MHz, $CDCl_3$): δ 8.96 (s, 1H), 8.82 (s, 1H), 7.46-7.25 (m, 10H), 7.19 (s, 1H), 7.17 (s, 1H), 7.04 (t, J = 7.3 Hz, 1H), 6.70 (t, J = 7.5 Hz, 1H), 5.01 (s, 2H); ^{13}C NMR (100 MHz, $CDCl_3$): δ 168.3, 145.6, 141.2, 135.2, 132.7, 129.3, 128.7, 128.6, 127.5, 122.7, 120.9, 117.9, 115.5, 115.4, 78.4; TLC R_f 0.30 (EtOAc/hexanes, 1/2); HRMS (ESI) $[M+Na]^+$ Calcd for $C_{20}H_{18}N_2Na_1O_2$: 341.1266, found: 341.1267.

3-(Benzyloxy)-1-phenylquinazoline-2,4(1H,3H)-dione[4b]

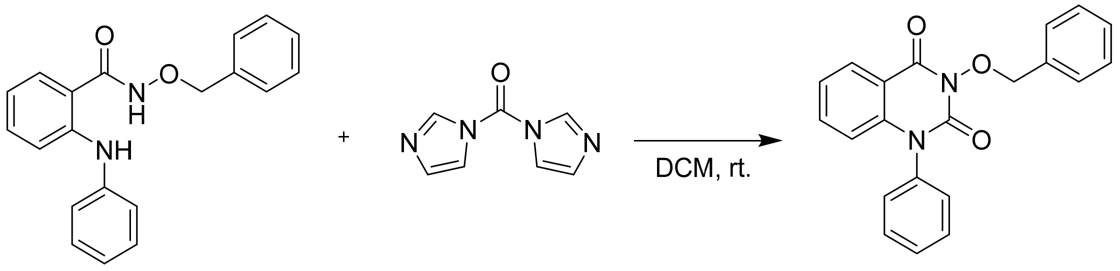

In a 250-m flask wwas placed *N*-(benzyloxy)-2-(phenylamino)benzamide (2.2 g, 7.0 mmol) and 1,1′- carbonyldiimidazole (2.5 g, 14.0 mmol, 2.0 equiv) in CH_2Cl_2 (70 mL). The reaction mixture was stirred at ambient temperature for 12 h. The mixture was concentrated to 30% of its original volume and then washed with aqueous 1*N* HCl solution (15 mL× 3). The remaining organic layer was concentrated by rotatory evaporation to give 1.89 g (yield 55% yield) of 3-(benzyloxy)-1-phenylquinazoline-2,4(1H,3H)-dione: ^{1}H NMR (400 MHz, $CDCl_3$): δ 8.14 (dd, *J* = 7.8, 1.3 Hz, 1H), 7.67-7.40 (m, 11H), 7.33 (t, *J* = 7.3 Hz, 1H), 6.47 (d, *J* = 8.4 Hz, 1H), 5.16 (s, 2H); ^{13}C NMR (100 MHz, $CDCl_3$): δ 158.5, 148.2, 140.9, 136.2, 135.0, 134.4, 130.2, 129.5, 129.3, 128.9, 128.4, 127.6, 123.1, 115.4, 115.1, 77.5; TLC R*f* 0.11 (EtOAc/hexanes, 1/2); HRMS (ESI) $[M+Na]^+$ Calcd for $C_{21}H_{16}N_2Na_1O_3$: 367.1059, found: 367.1059.

3-Hydroxy-1-phenylquinazoline-2,4(1H,3H)-dione 5[3]

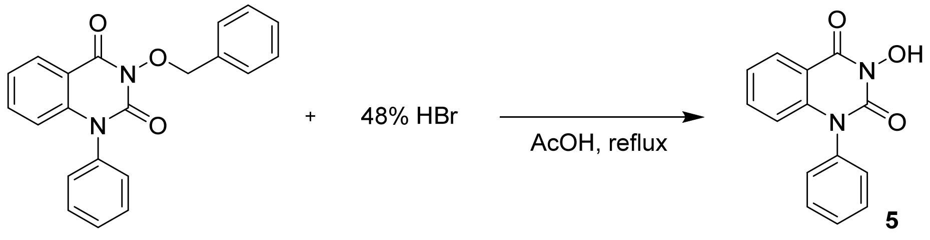

5

In a 100-mL double-necked flask was placed 3-(benzyloxy)-1-phenylquinazoline-2,4(1H,3H)-dione (1.7 g, 5.0 mmol) in glacial acetic acid (10 mL). The mixture was heated till dissolution of the substrate and 48% hydrobromic acid (5.0 mL) was then added. After having been stirred under reflux for 2.5 h, the reaction mixture was quenched with deionized water (50 mL) to induce precipitation of the product out. The collected solid was sequentially washed with deionized water (30 mL×3) and n-hexane (30 mL×3), and then dried under vacuum to obtain 720 mg (57% yield) of **5** as a white solid: ^{1}H NMR (400 MHz, DMSO-d_6): δ 10.85 (s, 1H), δ 8.10 (dd, *J* = 6.6, 1.3 Hz, 1H), δ 7.65-7.56 (m, 2H), δ 7.48-7.46 (m, 2H), δ 7.29 (t, *J* = 7.5Hz, 1H), δ 6.44 (d, *J* = 8.4 Hz, 1H); ^{13}C NMR (100 MHz, DMSO-d_6): δ 158.8, 149.0, 140.5, 136.4, 134.7, 130.2, 129.3, 127.5, 123.0, 115.3, 114.8; TLC R*f* 0.61 (EtOAc/hexanes, 1/1); HRMS (ESI) $[M+Na]^+$ Calcd for $C_{14}H_{10}N_2Na_1O_3$: 277.0589, found: 277.0587.

1-Benzyl-3-(benzyloxy)quinazoline-2,4(1H,3H)-dione 6-Bn[5]

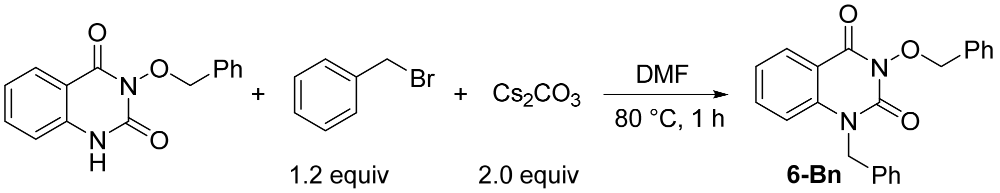

In a a 50-mL double-necked flask was placed 3-(benzyloxy)quinazoline-2,4(1H,3H)-dione (537.8 mg, 2.0 mmol) in *N*,*N*-dimethylformamide (DMF, 10 mL). Cesium carbonate (1321.4 mg, 4.0 mmol, 2.0 equiv) and benzyl bromide (286.3 μL, 1.2 mmol, 1.2 equiv) were added. After having been stirred at 80 °C for 1 h, the reaction was quenched with deionized water (30 mL) at ambient temperature. A copious amount of white solid was formed. The mixture was filtered through a suction funnel and the solid was rinsed with deionized water (30 mL). The collected solid was re-dissolved in acetone (40 mL) and concentrated by rotatory evaporation to remove acetone and the remaining DMF to obtain 586.9 mg (82% yield) of 6-Bn as a white solid: ^{1}H NMR (400 MHz, $CDCl_3$) δ 8.26 (dd, *J* = 7.8, 1.4 Hz 1H), 7.65-7.63 (m, 2H), 7.55 (td, *J* = 8.4, 1.6 Hz, 1H), 7.40-7.38 (m, 3H), 7.35-7.31 (m, 2H), 7.29 (m, 1H), 7.26-7.19 (m, 3H), 7.11 (d, *J* = 8.4 Hz, 1H), 5.37 (s, 2H), 5.29 (s, 2H); ^{13}C NMR (100 MHz, $CDCl_3$) δ 158.7, 149.6, 139.2, 135.2, 133.7, 130.2, 129.1, 129.0, 128.9, 128.4, 127.8, 126.5, 123.4, 115.9, 114.7, 78.4, 47.4; TLC R*f* 0.48 (EtOAc/hexanes, 1/1); HRMS (ESI) $[M+Na]^+$ Calcd for $C_{22}H_{18}N_2O_3Na$: 381.1215, found: 381.1209.

1-Benzyl-3-hydroxyquinazoline-2,4(1H,3H)-dione 6[3]

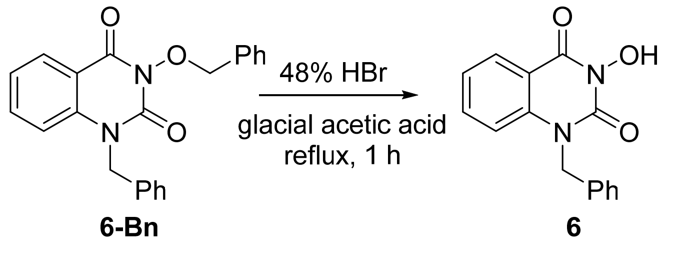

In a 25-mL two-necked flask was placed **6-Bn** (360.2 mg, 1.0 mmol) in glacial acetic acid (2 mL). The reaction mixture was heated to reflux in an oil bath and became homogeneous after 3 min. Hydrobromic acid (48%, 2 mL) was then added. After having been stirred for 1 h, the reaction

gradually cooled to ambient temperature. Deionized water (15 mL) was added to quench the reaction. The product precipitated out as a white solid. The solid was suction-filtered and washed with deionized water (30 mL). The wet solid was recrystallized from acetone (15 mL) to obtain 171.7 mg (64% yield) of **6**: ^{1}H NMR (400 MHz, DMSO-d$_6$) δ 10.86 (s, 1H), 8.08 (dd, *J* = 7.8, 1.2 Hz, 1H), 7.67 (t, *J* = 8.4, 1H), 7.35-7.26 (m, 7H), 5.39 (s, 2H); ^{13}C NMR (100 MHz, DMSO-d$_6$) δ 158.5, 149.8, 138.6, 136.1, 134.9, 128.7, 127.7, 127.3, 126.4, 123.1, 115.2, 115.1, 46.4; TLC R_f 0.19 (hexanes/EtOAc, 1/2); HRMS (ESI) [M+Na]$^+$ Calcd for $C_{15}H_{12}N_2O_3$+Na: 291.0746, found: 291.1070

3-Hydroxy-2-thioxo-2,3-dihydroquinazolin-4(1H)-one 7[6]

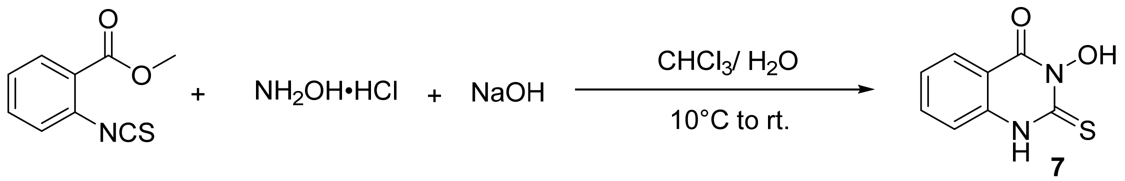

A solution of methyl 2-isothiocyanatobenzoate (956.3 mg, 5.0 mmol, 1.0 equiv) in chloroform (2 mL) was added at 5-10 °C to a stirred solution of NH_2OH•HCl (384.5 mg, 5.5 mmol, 1.1 equiv) and NaOH (234 mg, 5.5 mmol, 1.1 equiv) in water (10 mL). After 20 minutes added chloroform (5 mL) and water (20 mL) to let reaction dissolved. The reaction mixture was stirred at room temperature for 2 h. The precipitate that formed was filtered off, washed with water (10 mL) and hexane (20 mL), and dried to give a colorless solid: 732.4 mg (76%)^{1}H NMR (400 MHz, DMSO-d$_6$) δ 12.96 (s, 1H), 11.26 (s, 1H), 7.97 (d, *J* = 7.6 Hz, 1H), 7.73 (t, *J* = 7.2 Hz, 1H), 7.39 (d, *J* = 8.2 Hz, 1H), 7.33 (t, *J* = 7.6 Hz, 1H); ^{13}C NMR (100 MHz, DMSO-d$_6$) δ 172.7, 156.6, 138.7, 135.1, 127.0, 124.3, 115.9, 115.8; R_f 0.22 (MeOH/CH_2Cl_2, 1/5); HRMS (ESI) [M+Na]$^+$ Calcd for $C_8H_6N_2O_2S$+Na: 217.0048, found: 217.0061

Representative catalytic procedure by V(O)-1

(*R*)-3-(1-(3-Bromophenyl)-3,3,3-trifluoropropoxy)quinazolin-4(3H)-one 8a

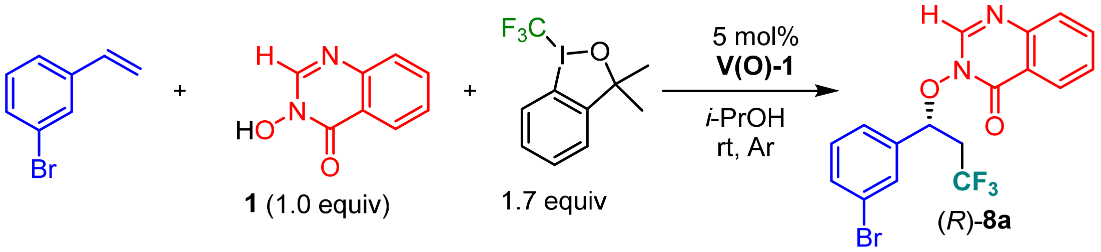

To an oven dried reaction tube (1.5 cm OD × 15.0 cm height) was placed **V(O)-1** (10.3 mg, 0.02 mmol, 5 mol%) and 2-H-Quiz **1** (65.3 mg, 0.4 mmol) in anhydrous *i*-PrOH (degassed by Ar, 2 mL) followed by addition of 3-bromo-styrene (52.7 µL, 0.4 mmol, 1.0 equiv) thru a microsyringe under Ar atmosphere. A solution of Togni reagent (225.9 mg, 0.68 mmol, 1.7 equiv) in anhydrous *i*-PrOH (1+1 mL) was added. The resulting reaction mixture was stirred at ambient temperature for 68 h and was concentrated by rotatory evaporation. The crude mixture was purified by flash column chromatography (EtOAc/ hexanes, 1/4) on silica gel to give 127.2 mg (71% yield) of **8a** as a white solid: ^{1}H NMR (400 MHz, $CDCl_3$) δ 8.31 (dd, J = 8.0, 1.4 Hz 1H), 7.75 (td, J = 7.6, 1.6 Hz, 1H), 7.64-7.62 (m, 2H), 7.55-7.50 (m, 3H), 7.30-7.23 (m, 2H), 5.76 (t, J = 6.6 Hz, 1H), 3.24-3.11 (m, 1H), 2.84-2.71 (m, 1H); ^{13}C NMR (100 MHz, $CDCl_3$) δ 157.4, 146.6, 144.0, 137.7, 134.6, 133.6, 130.9, 130.8, 127.9, 127.6, 126.8, 124.7 (q, $^1J_{C\text{-}F}$ = 275.3 Hz), 123.5, 123.3, 81.3, 39.0 (q, $^2J_{C\text{-}F}$ = 29.5 Hz); ^{19}F NMR (471 MHz, $CDCl_3$) δ -62.96 (s, 3F); TLC R_f 0.25 (EtOAc/Hexanes, 1/4); HRMS (FD) $[M]^+$ Calcd for $C_{17}H_{12}F_3N_2O_2Br$: 412.0029, found: 412.0030; HPLC analysis from racemic product: t_R 16.4 min (*S*), 19.5 min (*R*) (Chiralpak AS-H, *i*-PrOH/hexane, 5/95, 1.0 ml/min, λ = 254 nm); For the reaction catalyzed by **V(O)-1**: t_R 16.2 min (minor, 18.4%), 18.9 min (major, 81.6%) for 63 % ee (*R*); $[\alpha]_D^{30}$ +301.4 (*c* 0.48, $CHCl_3$) for 63% ee (*R*); For the reaction catalyzed by **V(O)-2** (51.1 mg, 31% yield): t_R 25.4 min (major, 83.2%), 33.0 min (minor, 16.8%); $[\alpha]_D^{29}$ -313.7 (*c* 0.25, $CHCl_3$) for 66 % ee (*S*); For racemic synthesis catalyzed by racemic **V(O)-1** in *i*-PrOH: 75% yield (134.3 mg, 68h).

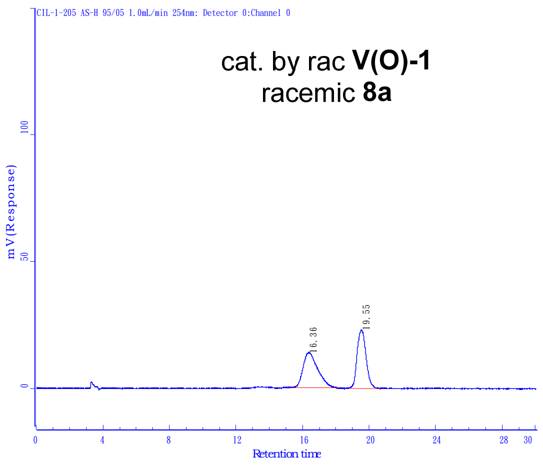

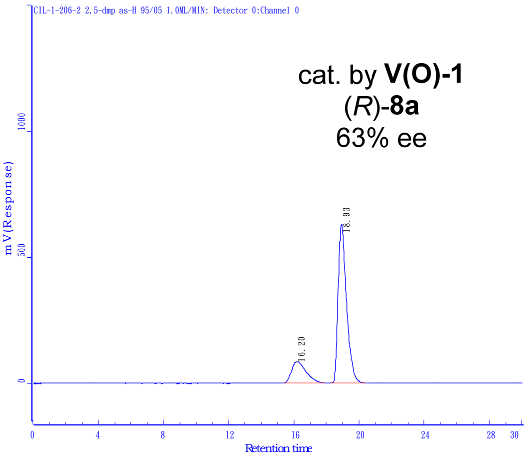

#	Peak name	ax/min (mim)	Area	Area %	Assay %
0		16.362	871.91	49.93	0.00
1		19.549	874.48	50.07	0.00

#	Peak name	ax/min (mim)	Area	Area %	Assay %
0		16.201	5085.47	18.36	0.00
1		18.929	22610.92	81.64	0.00

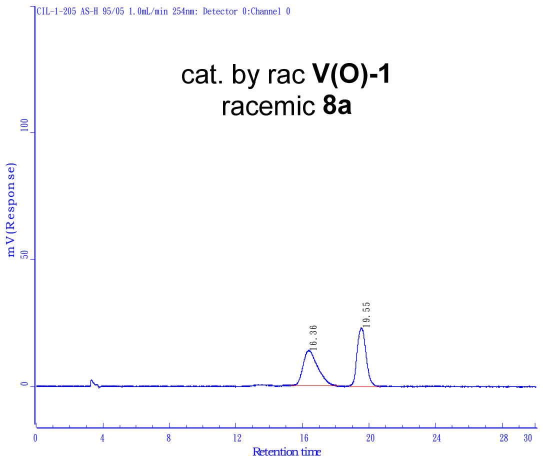

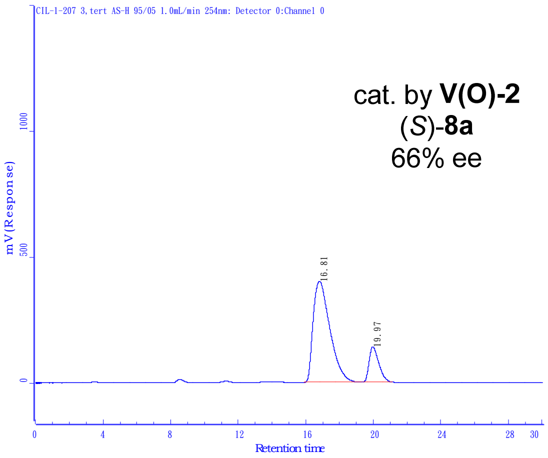

#	Peak name	ax/min (mim)	Area	Area %	Assay %
0		16.362	871.91	49.93	0.00
1		19.549	874.48	50.07	0.00

#	Peak name	ax/min (mim)	Area	Area %	Assay %
0		16.807	27530.51	83.20	0.00
1		19.966	5560.15	16.80	0.00

Representative catalytic procedure by V(O)-2

(*S*)-3-(1-(3-Bromophenyl)-3,3,3-trifluoropropoxy)-2-phenylquinazolin-4(3H)-one 9a

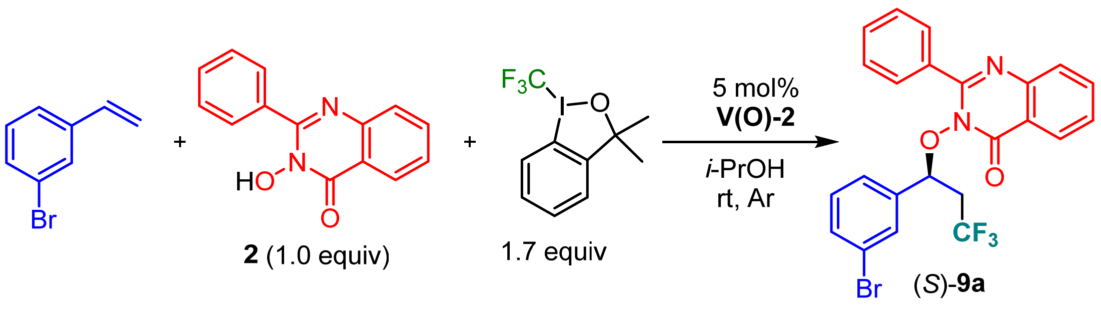

In an oven dried reaction tube (1.5 cm OD × 15.0 cm height) was placed **V(O)-2** (9.3 mg, 0.02

mmol, 5 mol%) and 2-Ph-Quiz **2** (95.5 mg, 0.4 mmol, 1.0 equiv) in anhydrous *i*-PrOH (degassed by Ar, 2 mL) followed by addition of 3-bromo-styrene (73 μL, 0.56 mmol, 1.4 equiv) thru a microsyringe under Ar atmosphere. A solution of Togni reagent (224.6 mg, 0.68 mmol, 1.7 equiv) in anhydrous *i*-PrOH (1+1 mL) was added. The resulting reaction mixture was stirred at ambient temperature for 92 h and was concentrated by rotatory evaporation. The crude mixture was purified by flash column chromatography (EtOAc/ hexanes, 1/10) on silica gel to give 54.8 mg (28%) of (*S*)-**9a** as a white solid: ^{1}H NMR (400 MHz, $CDCl_3$) δ 8.34 (dd, *J* = 8.0, 1.2 Hz, 1H), 7.77 (td, *J* = 7.6, 1.5 Hz, 1H), 7.70 (d, *J* = 7.9 Hz, 1H), 7.56-7.49 (m, 4H), 7.46-7.40 (m, 2H), 7.35 (d, *J* = 8.4 Hz, 1H), 6.94 (t, *J* = 7.9 Hz, 1H), 6.81-6.73 (m, 2H), 5.66 (t, *J* = 6.7 Hz, 1H), 3.10-2.91 (m, 1H), 2.67-2.49 (m, 1H); ^{13}C NMR (100 MHz, $CDCl_3$) δ 158.3, 153.5, 146.3, 136.4, 134.6, 133.0, 132.3, 130.9, 130.7, 129.9, 129.6, 128.1, 128.0, 127.4, 127.1, 126.7, 124.7 (q, $^1J_{C\text{-}F}$ = 277.2 Hz), 122.7, 122.1, 80.4, 38.1 (q, $^2J_{C\text{-}F}$ = 29.6 Hz); ^{19}F NMR (471 MHz, $CDCl_3$) δ -63.01 (s, 3F); TLC R_f 0.19 (CH_2Cl_2/Hexanes, 1/4); HRMS (FD) $[M]^+$ Calcd for $C_{23}H_{16}F_3N_2O_2Br$: 488.0236, found: 488.0354; HPLC analysis from racemic product: t_R 25.1 min (*S*), 32.6 min (*R*) (Chiralpak AS-H, *i*-PrOH/hexane, 1/99, 0.5 ml/min, λ = 254 nm); From the reaction catalyzed by **V(O)-2**: t_R 25.4 min (major, 95.6%), 33.0 min (minor, 4.4%) for 91.2 % ee (*S*); $[\alpha]_D^{22}$ +122.4 (*c* 1.0, $CHCl_3$) for 91% ee (*R*); From the reaction catalyzed by **V(O)-1** (88.1mg, 45% yield, 84h): t_R 26.5 min (minor, 24.9%), 32.8 min (major, 75.1%) for 50 % ee (*R*); For racemic synthesis catalyzed by racemic **V(O)-1** in *i*-PrOH: 31% yield (60.7 mg, 92h).

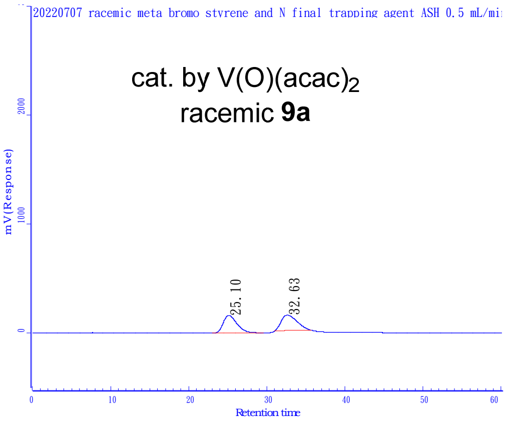

#	Peak name	ax/min (mim)	Area	Area %	Assay %
0		25.103	19442.05	49.71	0.00
1		32.626	19671.63	50.29	0.00

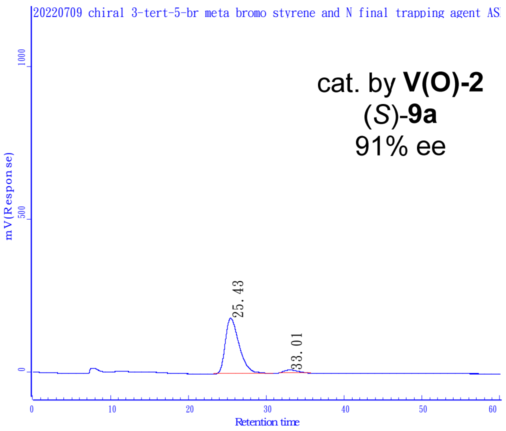

#	Peak name	ax/min (mim)	Area	Area %	Assay %
0		25.434	22367.08	95.60	0.00
1		33.011	1029.88	4.40	0.00

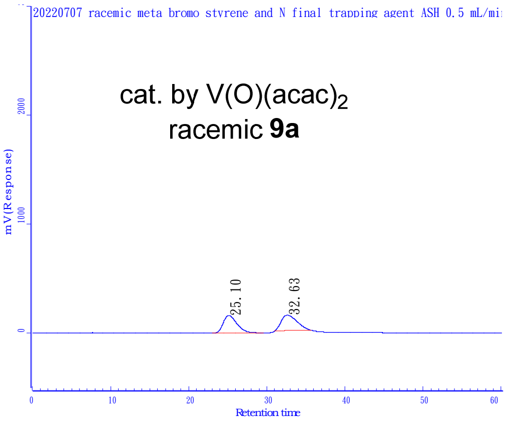

#	Peak name	ax/min (mim)	Area	Area %	Assay %
0		25.103	19442.05	49.71	0.00
1		32.626	19671.63	50.29	0.00

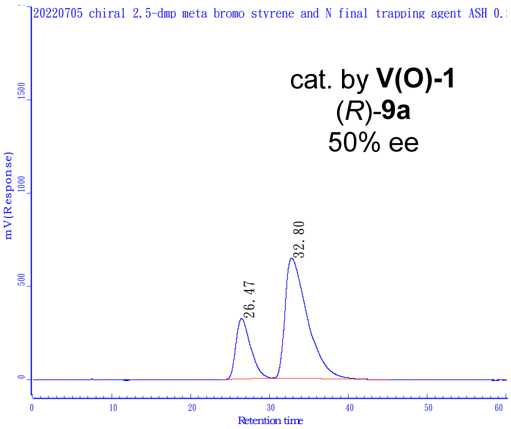

#	Peak name	ax/min (mim)	Area	Area %	Assay %
0		26.468	41803.19	24.88	0.00
1		32.804	126238.69	75.12	0.00

(*S*)-2-Phenyl-3-(3,3,3-trifluoro-1-(*m*-tolyl)propoxy)quinazolin-4(3H)-one 9b

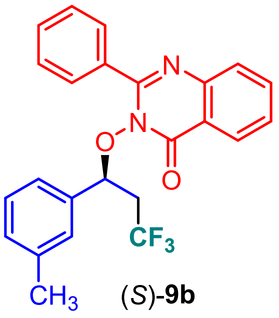

(*S*)-**9b**

Data for **9b**: 61.5 mg, 31% yield, 108h: ^{1}H NMR (400 MHz, $CDCl_3$) δ 8.34 (dd, *J* = 8.0, 1.3 Hz, 1H), 7.74 (td, *J* = 7.8, 1.4 Hz, 1H), 7.68 (d, *J* = 7.5 Hz, 1H), 7.54-7.45 (m, 4H), 7.40 (t, *J* = 7.8 Hz, 2H), 7.02 (d, *J* = 7.6 Hz, 1H), 6.93 (t, *J* = 7.6 Hz, 1H), 6.57 (d, *J* = 7.6 Hz, 1H), 6.47 (s, 1H), 5.72 (t, *J* = 6.7 Hz, 1H), 3.05-2.90 (m, 1H), 2.70-2.51 (m, 1H) 2.07 (s, 3H); ^{13}C NMR (100 MHz, $CDCl_3$) δ 158.4, 153.7, 146.3, 142.7, 138.1, 134.4, 133.9, 132.7, 130.6, 130.3, 129.8, 128.7, 128.2, 127.8, 126.9, 126.7, 125.9, 125.0 (q, $^{1}J_{C\text{-}F}$ = 277.6 Hz), 122.1, 80.8, 38.0 (q, $^{2}J_{C\text{-}F}$ = 29.3 Hz), 21.2; ^{19}F NMR (471 MHz, $CDCl_3$) δ -63.15 (s, 3F); TLC R_f 0.44 (EtOAc/Hexanes, 1/6); HRMS (FD)

$[M]^+$ Calcd for $C_{24}H_{19}F_3N_2O_2$: 424.14041, found: 424.14078; HPLC analysis from racemic product: t_R 21.1 min (*S*), 24.8 min (*R*) (Chiralpak AS-H, *i*-PrOH/hexane, 1/99, 0.5 ml/min, λ = 254 nm); From the reaction catalyzed by **V(O)-2**: t_R 19.9 min (major, 93.8%), 23.3 min (minor, 6.2%); $[\alpha]_D^{22}$ +111.5 (*c* 1.0, $CHCl_3$) for 88% ee (*S*); From the reactiuon catalyzed by **V(O)-1** (118.2 mg, 60% yield, 70h): t_R 21.1 min (minor, 27.3%), 24.6 min (major, 72.7%) for 45% ee (*R*).

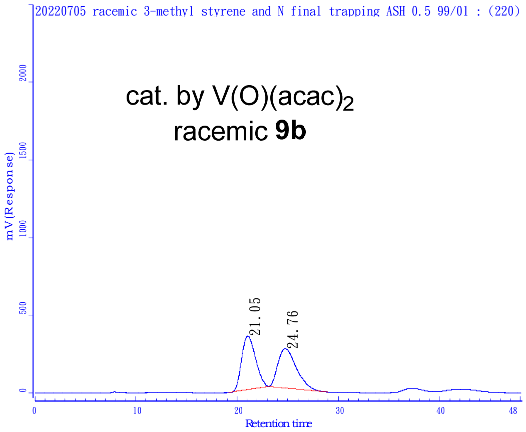

#	Peak name	ax/min (mim)	Area	Area %	Assay %
0		21.051	32834.79	50.39	0.00
1		24.762	32321.23	49.61	0.00

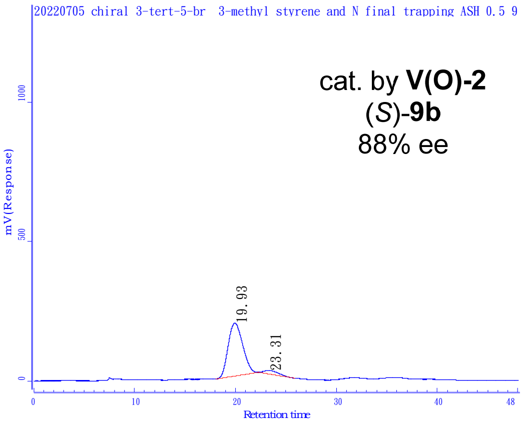

#	Peak name	ax/min (mim)	Area	Area %	Assay %
0		19.932	19084.85	93.75	0.00
1		23.311	1273.37	6.25	0.00

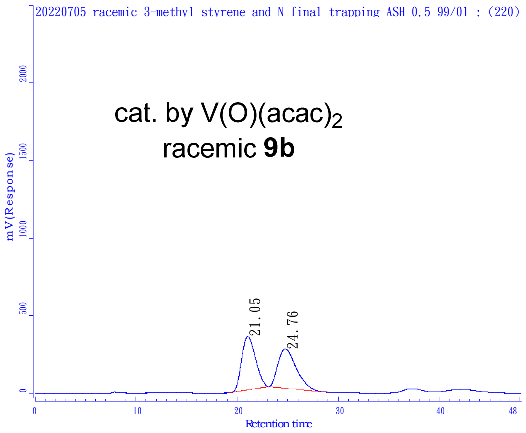

#	Peak name	ax/min (mim)	Area	Area %	Assay %
0		21.051	32834.79	50.39	0.00
1		24.762	32321.23	49.61	0.00

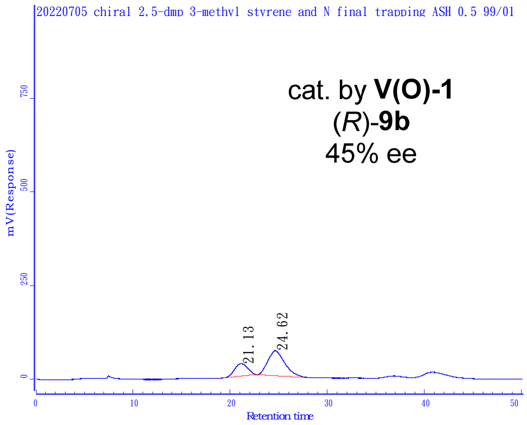

#	Peak name	ax/min (mim)	Area	Area %	Assay %
0		21.128	3056.77	27.33	0.00
1		24.624	8129.88	72.67	0.00

(*S*)-2-Phenyl-3-(3,3,3-trifluoro-1-(*p*-tolyl)propoxy)quinazolin-4(3H)-one 9c

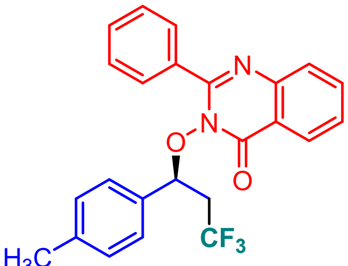

(*S*)-**9c**

Data for **9c**: 107.3 mg, 55% yield, 92h: ^{1}H NMR (400 MHz, $CDCl_3$) δ 8.33 (d, *J* = 7.8 Hz, 1H), 7.74 (td, *J* = 7.7, 1.2 Hz, 1H), 7.68 (d, *J* = 8.0 Hz, 1H), 7.53-7.44 (m, 4H), 7.38 (t, *J* = 7.7 Hz, 2H), 6.82 (d, *J* = 7.9 Hz, 2H), 6.62 (d, *J* = 7.9 Hz, 2H), 5.67 (t, *J* = 6.7 Hz, 1H), 3.05-2.90 (m, 1H), 2.68-2.52 (m, 1H) 2.24 (s, 3H); ^{13}C NMR (100 MHz, $CDCl_3$) δ 158.4, 153.7, 146.3, 139.7, 134.4, 132.7, 131.0, 130.3, 129.8, 129.1, 128.3, 127.9, 127.7, 126.9, 126.7, 125.0 (q, $^{1}J_{C\text{-}F}$ = 277.4 Hz), 122.2, 80.7, 38.0 (q, $^{2}J_{C\text{-}F}$ = 29.8 Hz), 21.2; ^{19}F NMR (471 MHz, $CDCl_3$) δ -63.10 (s, 3F); TLC R_f 0.36 (EtOAc/Hexanes, 1/6); HRMS (FD) $[M]^+$ Calcd for $C_{24}H_{19}F_3N_2O_2$: 424.14041, found: 424.14026; HPLC analysis from racemic product: t_R 23.6 min (*S*), 31.4 min (*R*) (Chiralpak OJ-H, *i*-PrOH/hexane, 2.5/97.5, 0.5 ml/min, λ = 254 nm); From the reaction catalyzed by **V(O)-2**: t_R 19.9 min (major, 93.8%), 23.3 min (minor, 6.2%); $[\alpha]_D^{22}$ +108.3 (*c* 1.0, $CHCl_3$) for 85% ee (*S*); From the reactiuon catalyzed by **V(O)-1** (137.1 mg, 70% yield, 52h): t_R 22.4 min (minor, 37.1%), 29.7 min (major, 62.9%) for 26% ee (*R*).

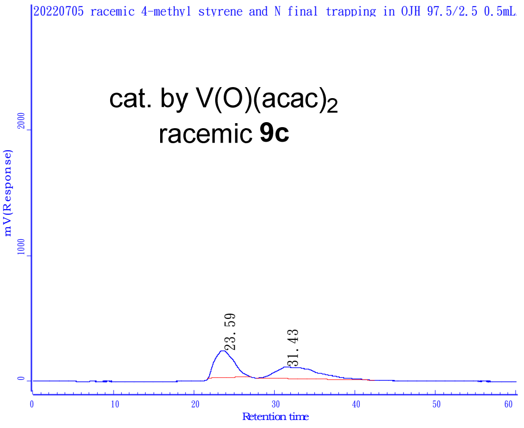

#	Peak name	ax/min (mim)	Area	Area %	Assay %
0		23.588	35243.57	50.43	0.00
1		31.427	34638.60	49.57	0.00

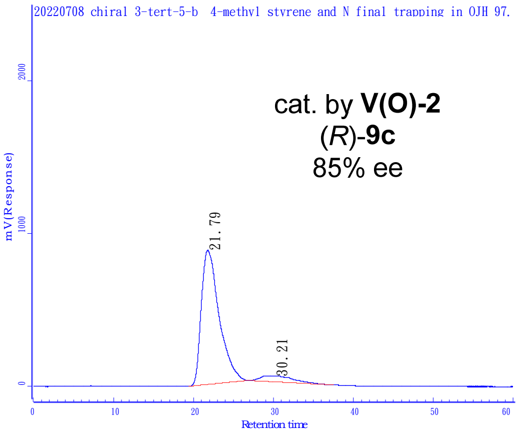

#	Peak name	ax/min (mim)	Area	Area %	Assay %
0		21.786	136480.56	92.57	0.00
1		30.214	10949.51	7.43	0.00

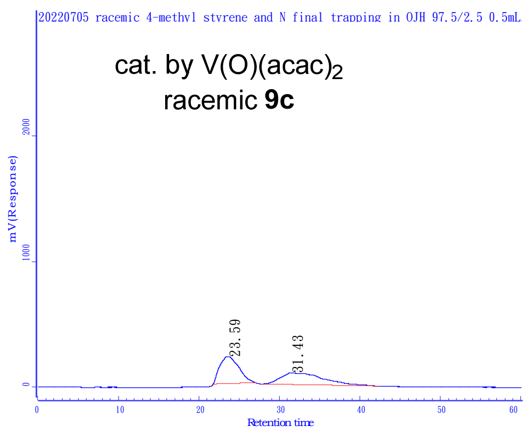

#	Peak name	ax/min (mim)	Area	Area %	Assay %
0		23.588	35243.57	50.43	0.00
1		31.427	34638.60	49.57	0.00

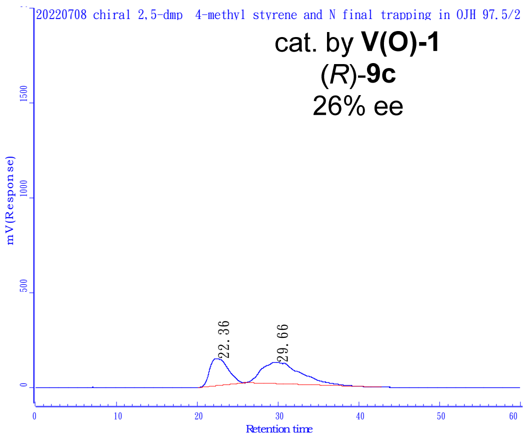

#	Peak name	ax/min (mim)	Area	Area %	Assay %
0		22.361	22673.62	37.10	0.00
1		29.662	38437.98	62.90	0.00

(*R*)-3-(1-(3-Bromophenyl)-3,3,3-trifluoropropoxy)-2-(methylthio)quinazolin-4(3H)-one 10a

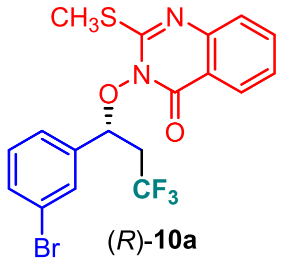

(*R*)-**10a**

Data for **10a**: 114.7mg, 62% yield, 30h: ^{1}H NMR (500 MHz, $CDCl_3$) δ 8.18 (d, *J* = 8.0 Hz, 1H), 7.70 (s, 1H), 7.68 (d, *J* = 7.6 Hz, 1H), 7.53 (d, *J* = 8.2 Hz, 1H), 7.49 (d, *J* = 8.2 Hz, 1H), 7.41-7.37 (m, 2H), 7.20 (t, *J* = 8.0 Hz, 1H), 5.98 (dd, *J* = 8.0, 5.4 Hz, 1H), 3.25-3.15 (m, 1H), 2.98-2.87 (m, 1H), 2.52 (s, 3H); ^{13}C NMR (100 MHz, $CDCl_3$) δ 158.3, 156.5, 146.5, 137.0, 133.2, 131.6, 130.1, 127.5, 126.8, 126.4, 125.8, 124.8 (q, $^1J_{C\text{-}F}$ = 275.4 Hz), 122.5, 120.5, 81.1 (q, $^3J_{C\text{-}F}$ = 2.4 Hz, 38.3 (q, $^2J_{C\text{-}F}$ = 28.8 Hz), 13.8; ^{19}F NMR (471 MHz, $CDCl_3$) δ -62.83 (s, 3F); TLC R_f 0.25 (EtOAc/hexanes, 1/20); HRMS (FD) $[M]^+$ Calcd for $C_{18}H_{14}N_2O_2F_3BrS$: 457.9906, found: 457.9897; HPLC analysis from racemic product: t_R 15.1 min (*S*), 20.2 min (*R*) (Chiralpak AS-H, *i*-PrOH/hexane, 1.5/98.5, 0.8 ml/min, λ = 254 nm); For the reaction catalyzed by **V(O)-1**: t_R 15.8 min (minor, 31.0%), 21.7 min (major, 69.0%); $[\alpha]_D^{29}$ +60.1 (*c* 0.42, $CHCl_3$) for 38% ee (*R*); For the reaction catalyzed by **V(O)-2** (71.2 mg, 39% yield, 50h): t_R 15.4 min (major, 75.0%), 21.4 min (minor, 25.0%); $[\alpha]_D^{29}$ -77.2 (*c* 0.14, $CHCl_3$) for 50% ee (*S*); For racemic synthesis catalyzed by racemic **V(O)-1** in *i*-PrOH: 62% yield (114.3 mg, 30h).

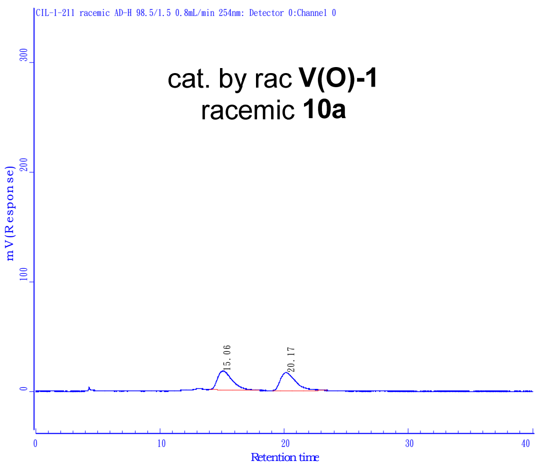

#	Peak name	ax/min (mim)	Area	Area %	Assay %
0		15.056	1428.16	50.07	0.00
1		20.171	1424.13	49.93	0.00

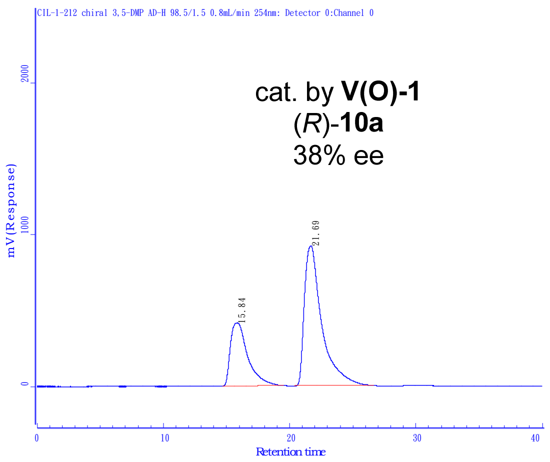

#	Peak name	ax/min (mim)	Area	Area %	Assay %
0		15.837	39710.54	31.01	0.00
1		21.687	88349.59	68.99	0.00

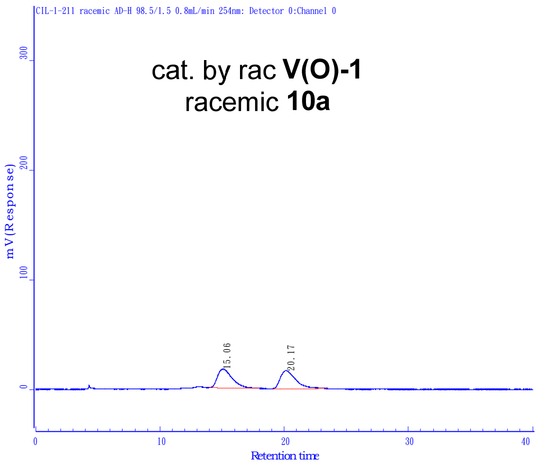

#	Peak name	ax/min (mim)	Area	Area %	Assay %
0		15.056	1428.16	50.07	0.00
1		20.171	1424.13	49.93	0.00

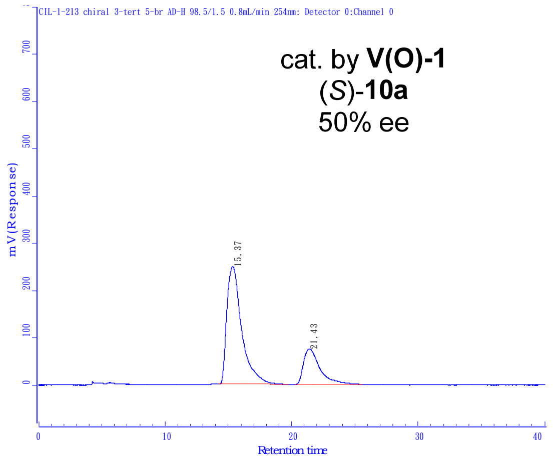

#	Peak name	ax/min (mim)	Area	Area %	Assay %
0		15.368	20268.12	74.98	0.00
1		21.429	6763.41	25.02	0.00

Representative catalytic procedure by V(O)-1

3-(3,3,3-Trifluoro-1-phenylpropoxy)quinazoline-2,4(1H,3H)-dione 11c

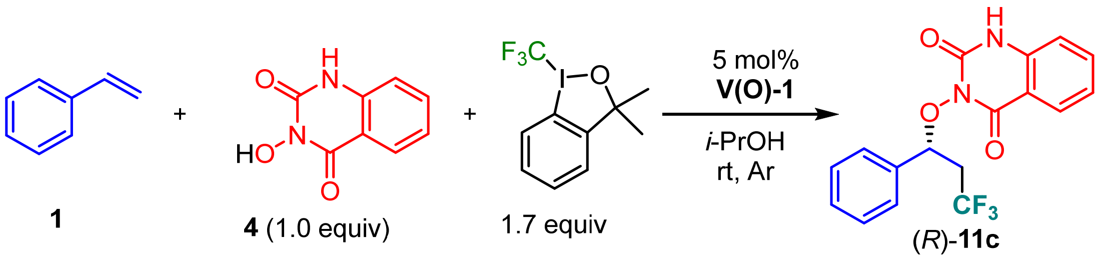

To an oven dried reaction tube (1.5 cm OD × 15.0 cm height) was placed **V(O)-1** (9.3 mg, 0.019 mmol, 4.5 mol%) and **4** (71.3 mg, 0.4 mmol, 1.0 equiv) in anhydrous *i*-PrOH (degassed by Ar, 2 mL)

followed by addition of styrene (45.8 μL, 0.4 mmol) thru a microsyringe under Ar atmosphere. A solution of Togni reagent (224.5 mg, 0.68 mmol, 1.7 equiv) in anhydrous *i*-PrOH (1+1 mL) was added. The resulting reaction mixture was stirred at ambient temperature for 51 h and was concentrated by rotatory evaporation. The crude mixture was purified by flash column chromatography (EtOAc/ hexanes, 1/2) on silica gel to give 122.7 mg (84%) of **11c** as a white solid: ^{1}H NMR (400 MHz, $CDCl_3$) δ 10.25 (s, 1H), 8.06 (d, *J* = 7.8 Hz, 1H), 7.62 (td, *J* = 7.2, 1.2 Hz, 1H), 7.52-7.50 (m, 2H), 7.32-7.30 (m, 3H), 7.24 (t, *J* = 7.2 Hz, 1H), 7.08 (d, *J* = 8.2 Hz, 1H), 5.80 (t, *J* = 6.4 Hz, 1H), 3.30-3.16 (m, 1H), 2.93-2.80 (m, 1H); ^{13}C NMR (100 MHz, $CDCl_3$) δ 159.4, 150.2, 137.5, 135.6, 135.3, 129.8, 128.5, 128.4, 128.3, 125.2 (q, $^{1}J_{C\text{-}F}$ = 275.3 Hz), 123.7, 115.3, 114.6, 81.6, 39.0 (q, $^{2}J_{C\text{-}F}$ = 28.7 Hz); ^{19}F NMR (471 MHz, $CDCl_3$): δ -63.2 (s, 3F); R_f 0.25 (EtOAc/hexanes, 1/2); HRMS (FD) $[M+H]^+$ Calcd for $C_{17}H_{14}F_3N_2O_3$: 351.0951, found: 351.0954; HPLC analysis from racemic product: t_R 15.5 min (*S*), 23.3 min (*R*) (Chiralpak AD-H, *i*-PrOH/*n*-hexane, 12/88, 0.8 mL/min, λ = 254 nm); For the reaction reaction catalysed by **V(O)-1**: HPLC t_R 15.3 min (major, 14.7%), 22.8 min (minor, 85.3%) for 71 %ee (*R*); For the reaction reaction catalysed by **V(O)-2** (120 h, 105.3 mg, 75%): HPLC t_R 15.5 min (major, 87.5%), 23.4 min (minor, 12.5%) for 75 %ee (*S*). Racemic reaction catalyzed by $V(O)(acac)_2$ in CH_3CN in 5h: 48.9 mg, 34% yield.

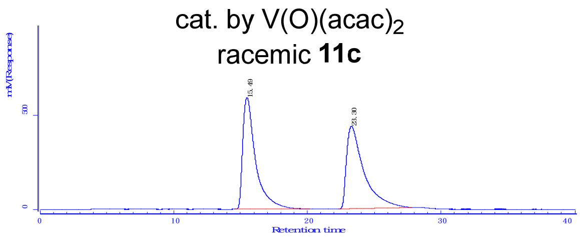

#	Peak name	ax/min (mim)	Area	Area %	Assay %
0		15.488	39716.10	50.05	0.00
1		23.298	39634.96	49.95	0.00

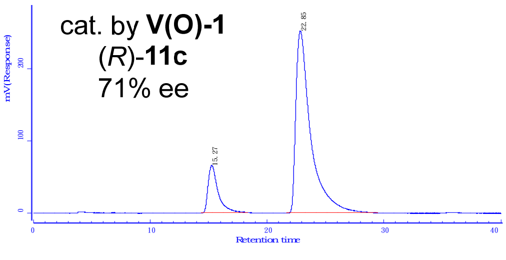

#	Peak name	ax/min (mim)	Area	Area %	Assay %
0		15.272	3816.13	14.69	0.00
1		22.849	22163.39	85.31	0.00

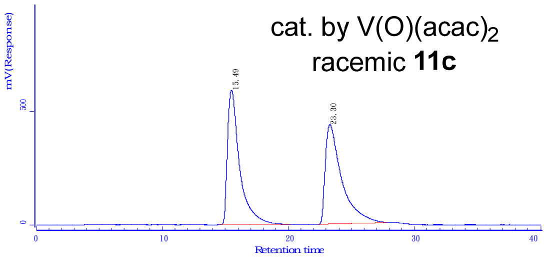

#	Peak name	ax/min (mim)	Area	Area %	Assay %
0		15.488	39716.10	50.05	0.00
1		23.298	39634.96	49.95	0.00

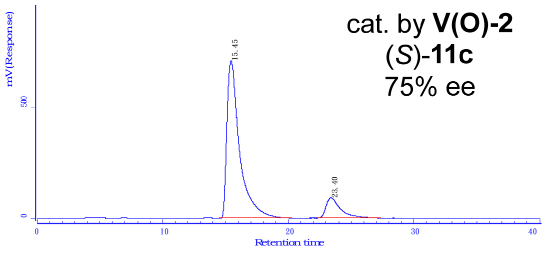

#	Peak name	ax/min (mim)	Area	Area %	Assay %
0		15.449	49345.80	87.46	0.00
1		23.405	7072.98	12.54	0.00

3-(3,3,3-Trifluoro-1-(m-tolyl)propoxy)quinazoline-2,4(1H,3H)-dione 11b

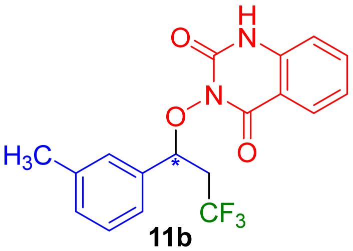

11b

White solid; Yield: 84%/54% (122.7/78.8 mg, 10 mol% cat. **V(O)-1**/5 mol% cat. **V(O)-2**, 38/48 h); ^{1}H NMR (400 MHz, $CDCl_3$) δ 10.51 (s, 1H), 8.07 (d, J = 8.0 Hz, 1H), 7.62 (td, J = 7.8, 1.2 Hz, 1H), 7.33-7.31 (m, 2H), 7.25 (d, J = 7.8 Hz, 1H), 7.20 (t, J = 8.2 Hz, 1H), 7.14-7.11 (m, 2H), 5.75 (t, J = 6.4 Hz, 1H), 3.28-3.15 (m, 1H), 2.92-2.79 (m, 1H), 2.29 (s, 3H); ^{13}C NMR (100 MHz, $CDCl_3$) δ 159.4, 150.4, 138.1, 137.6, 135.7, 135.3, 130.5, 129. 0, 128.3, 125.5, 125.3 (q, $^{1}J_{C\text{-}F}$ = 275.4 Hz), 123.7, 115.4, 114.7, 81.7, 39.1 (q, $^{2}J_{C\text{-}F}$ = 28.6 Hz), 21.3; ^{19}F NMR (471 MHz, $CDCl_3$) δ -63.08 (s, 3F); R_f 0.25 (EtOAc/Hexanes, 1/2); HRMS (ESI) $[M]^+$ Calcd for $C_{18}H_{15}F_3N_2O_3$: 364.1029, found: 364.1026; HPLC analysis from racemic product: t_R 19.4 min (*S*), 27.1 min (*R*) (Chiralpak AD-H, *i*-PrOH/n-hexane, 10/90, 0.6 ml/min, λ = 254 nm). For the reaction reaction catalyzed by **V(O)-1**: t_R 19.9 min (minor, 18.5%), 27.7 min (major, 81.5%); $[\alpha]_D^{25}$ +122.5 (*c*, 0.74, $CHCl_3$) for 63% ee (*R*); For the reaction catalyzed by **V(O)-2** (54% yield, 48 h): t_R 19.7 min (major, 84.0%), 27.8 min (minor, 16.0%) for 68% ee (*S*). For racemic synthesis by $V(O)(acac)_2$ in CH_3CN: 30.1% (43.9 mg, 96 h).

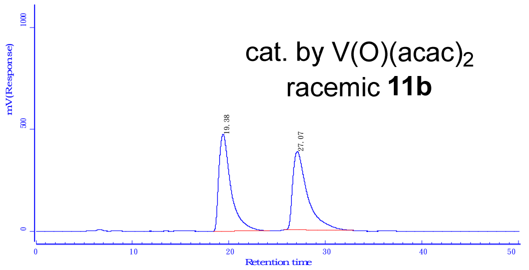

#	Peak name	ax/min (mim)	Area	Area %	Assay %
0		19.375	41164.82	50.00	0.00
1		27.069	41157.93	50.00	0.00

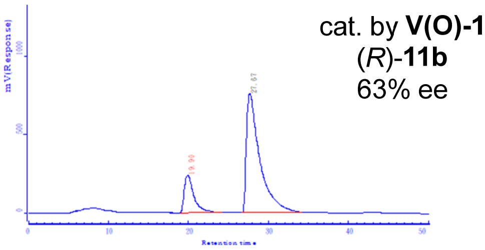

#	Peak name	ax/min (mim)	Area	Area %	Assay %
0		19.902	20130.70	18.52	0.00
1		27.671	88548.41	81.48	0.00

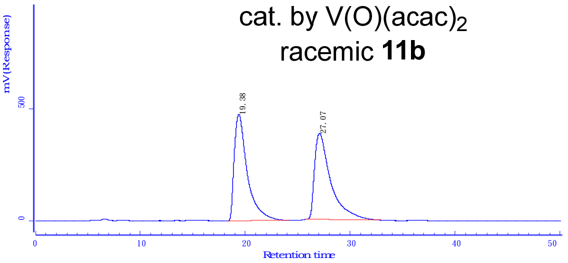

#	Peak name	ax/min (mim)	Area	Area %	Assay %
0		19.375	41164.82	50.00	0.00
1		27.069	41157.93	50.00	0.00

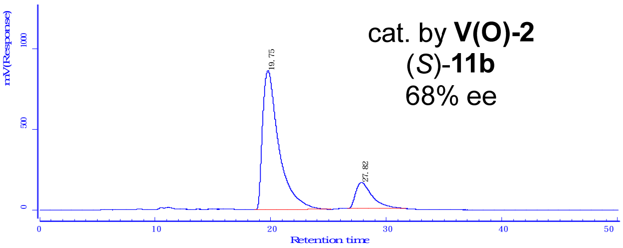

#	Peak name	ax/min (mim)	Area	Area %	Assay %
0		19.746	83838.64	84.01	0.00
1		27.824	15962.32	15.99	0.00

(*R*)-3-(1-(3-Chlorophenyl)-3,3,3-trifluoropropoxy)quinazoline-2,4(1H,3H)-dione 11a'

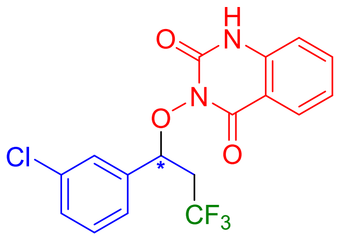

11a'

White solid; Yield: 83.1% (127.9 mg, 18h); ^{1}H NMR (400 MHz, $CDCl_3$) δ 10.48 (s, 1H), 8.08 (d, *J* = 8.0 Hz, 1H), 7.65 (td, *J* = 7.8, 1.0 Hz, 1H), 7.57 (s, 1H), 7.40 (d, *J* = 7.2 Hz, 1H), 7.33-7.30 (m, 1H), 7.29-7.27 (m, 1H), 7.62-7.24 (m, 1H), 7.12 (d, *J* = 8.0 Hz, 1H), 5.71 (t, *J* = 6.6 Hz, 1H), 3.27-3.14 (m, 1H), 2.89-2.76 (m, 1H); ^{13}C NMR (100 MHz, $CDCl_3$) δ 159.4, 150.3, 138.0, 137.5, 135.5, 134.3, 129.9, 129.8, 128.5, 128.4, 126.6, 125.0 (q, $^{1}J_{C\text{-}F}$ = 275.4 Hz), 123.9, 115.5, 114.6, 81.2, 39.2 (q, $^{2}J_{C\text{-}F}$ = 28.9 Hz); ^{19}F NMR (471 MHz, $CDCl_3$) δ -63.08 (s, 3F); R_f 0.25 (EtOAc/Hexanes, 1/2); HRMS (FD) $[M]^+$ Calcd for $C_{17}H_{12}F_3N_2O_3Cl$: 384.0483, found: 384.0480; HPLC analysis from racemic product: t_R 19.05 min (*S*), 26.40 min (*R*) (Chiralpak AD-H, *i*-PrOH/hexane, 12/88, 0.6 ml/min, λ = 254 nm). For the reaction catalyzed by **V(O)-1**: t_R 19.13 min (minor, 14.1%), 26.22 min (major, 85.9%); $[\alpha]_D^{30}$ +124.0 (*c*, 1.0, $CHCl_3$) for 72% ee (*R*); 74.3% yield (114.3 mg, 18h) for racemic synthesis catalyzed by racemic **V(O)-1**.

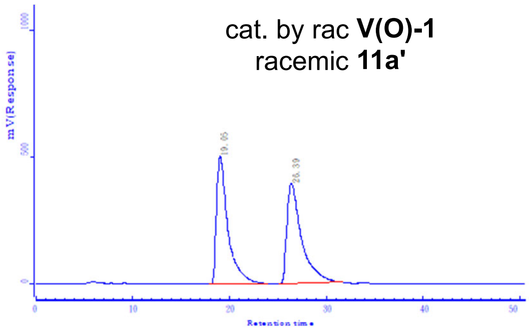

#	Peak name	ax/min (mim)	Area	Area %	Assay %
0		19.052	41641.29	50.02	0.00
1		26.395	41601.52	49.98	0.00

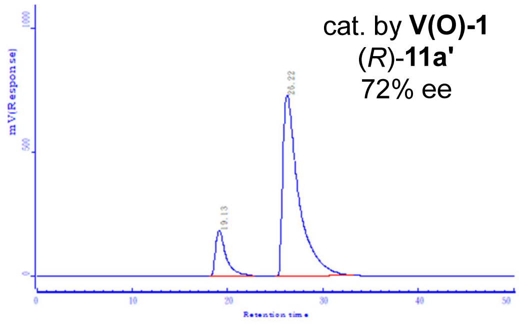

#	Peak name	ax/min (mim)	Area	Area %	Assay %
0		19.126	13951.66	14.08	0.00
1		26.221	85135.70	85.92	0.00

(*R*)-3-(1-(3-Bromophenyl)-3,3,3-trifluoropropoxy)quinazoline-2,4(1H,3H)-dione 11a

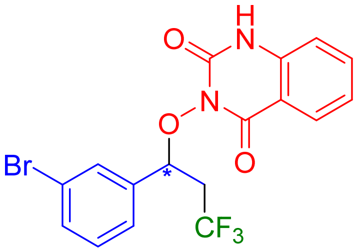

11a

White solid; Yield 88% (144.2 mg, 43h); Data for **10a**: ^{1}H NMR (400 MHz, $CDCl_3$) δ 10.49 (s, 1H), 8.08 (d, *J* = 7.8 Hz, 1H), 7.62 (td, *J* = 7.2, 1.4 Hz, 1H), 7.48-7.45 (m, 2H), 7.26 (t, *J* = 8.0 Hz, 1H), 7.21 (t, *J* = 7.8 Hz, 1H), 7.13 (d, *J* = 8.2 Hz, 1H), 5.70 (t, *J* = 6.6 Hz, 1H), 3.27-3.14 (m, 1H), 2.89-2.76 (m, 1H); ^{13}C NMR (100 MHz, $CDCl_3$) δ 159.4, 150.3, 138.2, 137.6, 135.5, 132.8, 131.4, 130.0, 128.4, 127.0, 125.0 (q, $^{1}J_{C-F}$ = 275.3 Hz), 123.9, 122.4, 115.6, 114.6, 81.1, 39.2 (q, $^{2}J_{C-F}$ = 28.8 Hz); ^{19}F NMR (471 MHz, $CDCl_3$) δ -63.05 (s, 3F); R_f 0.25 (EtOAc/Hexanes, 1/2); HRMS (FD) $[M]^+$ Calcd for $C_{17}H_{12}F_3N_2O_3Br$: 427.9978, found: 427.9976; HPLC analysis from racemic product: t_R 12.8 min (*S*), 18.3 min (*R*) (Chiralpak AD-H, *i*-PrOH/hexane, 14/86, 0.8 ml/min, λ = 254 nm). For the reaction reaction catalyzed by **V(O)-1**: t_R 12.8 min (minor, 11.7%), 18.3 min (major, 88.3%); $[\alpha]_D^{24}$ +65.9 (*c*, 1.0, $CHCl_3$) for 77% ee (*R*). For the reaction catalyzed by **V(O)-2** (53% yield, 120h): t_R 12.9 min (major, 86.9%), 18.7 min (major, 13.1%) for 74% ee (*S*); For racemic synthesis catalyzed by V(O)(acac)$_2$ in CH_3CN: 30.6% yield (52.5 mg, 17h).

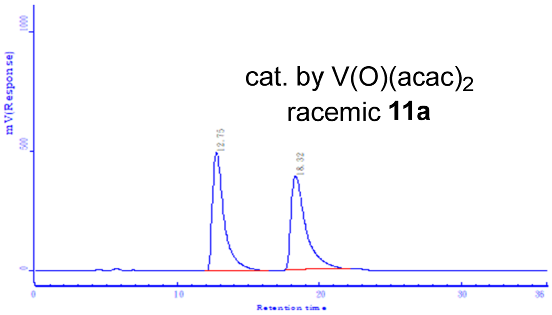

#	Peak name	ax/min (mim)	Area	Area %	Assay %
0		12.747	28170.73	50.00	0.00
1		18.316	28165.17	50.00	0.00

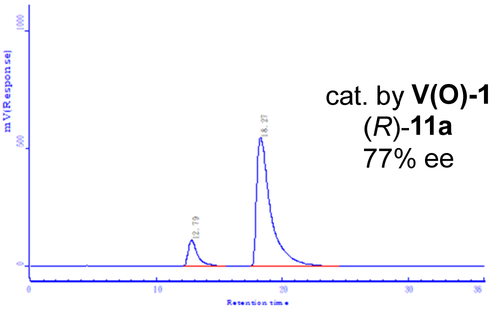

#	Peak name	ax/min (mim)	Area	Area %	Assay %
0		12.787	5602.01	11.74	0.00
1		18.272	42121.59	88.26	0.00

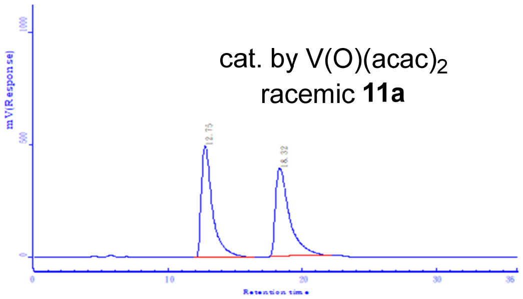

#	Peak name	ax/min (mim)	Area	Area %	Assay %
0		12.747	28170.73	50.00	0.00
1		18.316	28165.17	50.00	0.00

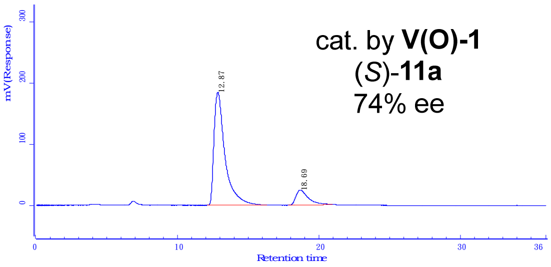

#	Peak name	ax/min (mim)	Area	Area %	Assay %
0		12.868	10002.91	86.94	0.00
1		18.695	1502.72	13.06	0.00

Representative catalytic procedure by V(O)-1

(*R*)-3-(1-(3-Bromophenyl)-3,3,3-trifluoropropoxy)-1-phenylquinazoline-2,4(1H,3H)-dione 12a

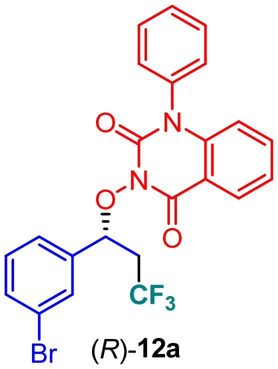

Data for **12a**: 187.3 mg, 93% yield, 18h: ^{1}H NMR (400 MHz, $CDCl_3$): δ 8.18 (dd, *J* = 7.9, 1.4, 1H), 7.66 (t, *J* = 1.8 Hz, 1H), 7.61-7.43 (m, 6H), 7.28-7.19 (m, 4H), 6.50 (d, *J* = 8.4 Hz, 1H), 5.78 (t, *J* = 6.5 Hz, 1H), 3.25-3.12 (m, 1H), 2.91-2.78 (m, 1H); ^{13}C NMR (126 MHz, $CDCl_3$): δ 158.9, 148.9, 140.6, 138.0, 135.6, 134.9, 132.7, 131.5, 130.4, 130.4, 130.1, 129.7, 128.9, 128.8, 128.6, 127.0, 125.0 (q, $^1J_{C\text{-}F}$ = 279.0 Hz), 123.6, 122.2, 115.6, 114.8, 80.7 (q, $^3J_{C\text{-}F}$ = 3.0 Hz), 38.9 (q, $^2J_{C\text{-}F}$ = 28.7 Hz); ^{19}F NMR (471 MHz, $CDCl_3$): δ -62.98 (s, 3F); TLC R_f 0.30 (EtOAc/hexanes, 1/6); HRMS (FD) $[M]^+$ Calcd for $C_{23}H_{16}BrF_3N_2O_3$: 504.0291, found: 504.0287; HPLC analysis from racemic product: t_R 19.3 min (*R*), 22.8 min (*S*); From the reaction catalyzed by **V(O)-1**: t_R 20.65 min (major, 84.1%), 24.59 min (minor, 15.9%) (Chiralpak AS-H, *i*-PrOH/*n*-hexane, 05/95, 1.0 mL/min, λ = 254 nm); $[\alpha]_D^{22}$ +108.4 (*c* 1.0, $CHCl_3$) for 68% ee (*R*); From the reaction

catalyzed by **V(O)-2** in EtOH (46% yield, 96 h): t_R 20.6 min (minor, 13.8%), 24.9 min (major, 86.2%) for 72% ee (*S*).

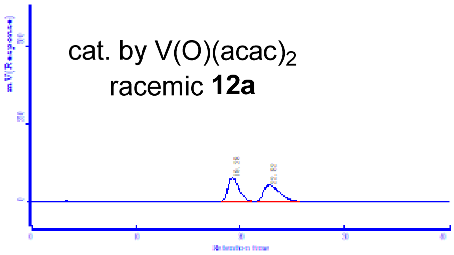

#	Peak name	ax/min (min)	Area	Area %	Assay %
0		19.278	5267.07	50.02	0.00
1		22.821	5263.19	49.98	0.00

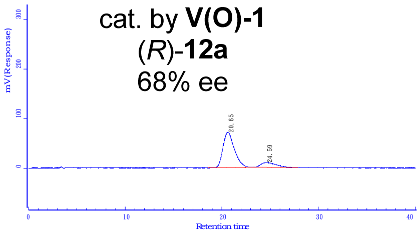

#	Peak name	ax/min (min)	Area	Area %	Assay %
0		20.650	5850.37	84.11	0.00
1		24.586	1105.51	15.89	0.00

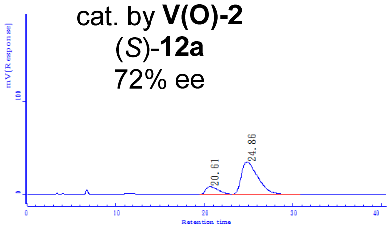

#	Peak name	ax/min (min)	Area	Area %	Assay %
0		20.611	713.70	13.80	0.00
1		24.860	4457.93	86.20	0.00

(*R*)-1-Phenyl-3-(3,3,3-trifluoro-1-(*m*-tolyl)propoxy)quinazoline-2,4(1H,3H)-dione 12b

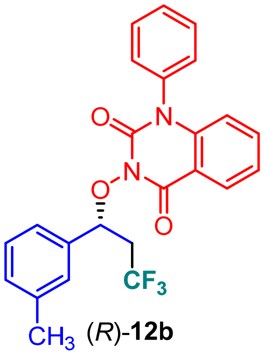

(*R*)-**12b**

Data for **12b**: 104.2 mg, 59% yield, 17h: ^{1}H NMR (400 MHz, $CDCl_3$) δ 8.18 (dd, *J* = 7.9, 1.0 Hz, 1H), 7.60-7.15 (m, 11H), 6.48 (d, *J* = 8.7 Hz, 1H), 5.83 (t, *J* = 6.2 Hz, 1H), 3.25-3.15 (m, 1H), 2.93-2.83 (m, 1H), 2.32 (s, 1H); ^{13}C NMR (100 MHz, $CDCl_3$) δ 159.0, 149.0, 140.6, 138.0, 135.7, 135.5, 134.8, 130.4, 129.6, 129.2, 128.9, 128.9, 128.6, 128.3, 125.6, 125.3 ($^1J_{C\text{-}F}$ = 277.2 Hz), 123.4, 115.5, 114.9, 81.4 ($^3J_{C\text{-}F}$ = 2.9 Hz), 38.9 ($^2J_{C\text{-}F}$ = 28.5 Hz), 21.2; ^{19}F NMR (471 MHz, $CDCl_3$) δ -63.2 (s, 3F); TLC R_f 0.28 (EtOAc/hexanes, 1/6);HRMS (FD) Calcd for $C_{24}H_{19}F_3N_2O_3$: 440.1353, found: 440.1359; HPLC from racemic product: t_R 19.3 min (*R*), 22.8 min (*S*); From the reaction catalyzed by **V(O)-1**: t_R 25.80 min (major, 88.6%), 29.74 min (minor, 11.4%) (Chiralpak AS-H, *i*-PrOH/*n*-hexane, 2/98, 1.0 mL/min, λ = 254 nm); $[\alpha]_D^{22}$ +72.6 (*c* 1.0, $CHCl_3$) for 77% ee (*R*); From the reaction catalyzed by **V(O)-2** in *i*-PrOH (56% yield, 130h): t_R 26.9 min (minor, 18%), 30.0 min (major, 82%) for 64% ee (*S*); From the reaction catalyzed by **V(O)-2** in EtOH (54% yield, 24h): t_R 27.1 min (minor, 12.3%), 30.5 min (major, 87.7%) for 75.4% ee (*S*).

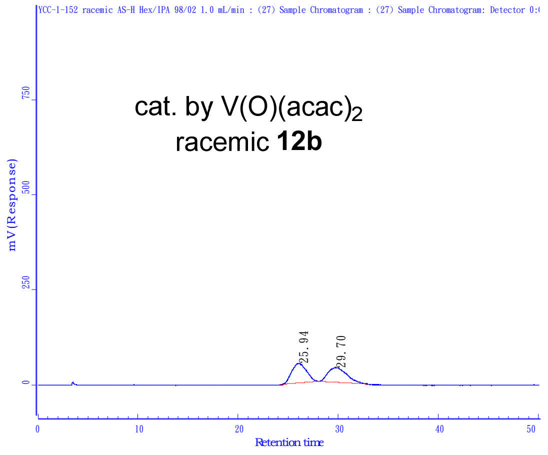

#	Peak name	ax/min (mim)	Area	Area %	Assay %
0		25.943	5225.68	50.80	0.00
1		29.696	5061.21	49.20	0.00

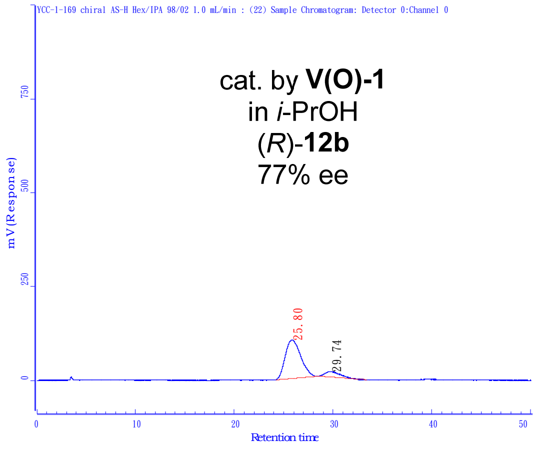

#	Peak name	ax/min (mim)	Area	Area %	Assay %
0		25.804	11159.31	88.58	0.00
1		29.744	1438.38	11.42	0.00

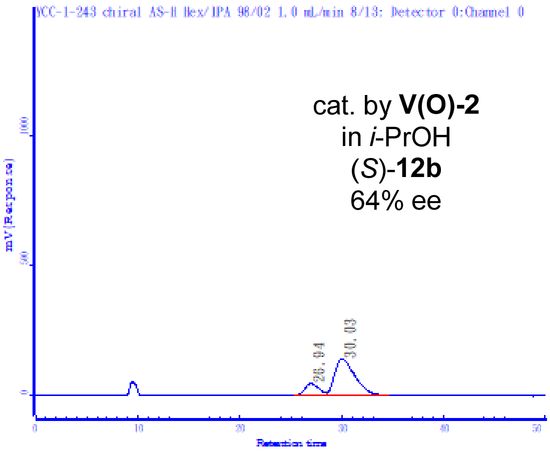

#	Peak name	ax/min (mim)	Area	Area %	Assay %
0		26.942	4246.58	17.94	0.00
1		30.030	19418.90	82.06	0.00

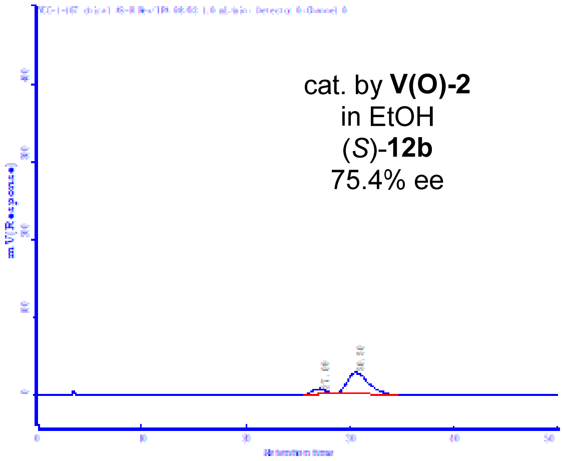

#	Peak name	ax/min (mim)	Area	Area %	Assay %
0		27.093	513.06	12.29	0.00
1		30.500	3660.08	87.71	0.00

(*R*)-1-Phenyl-3-(3,3,3-trifluoro-1-(*p*-tolyl)propoxy)quinazoline-2,4(1H,3H)-dione 12c

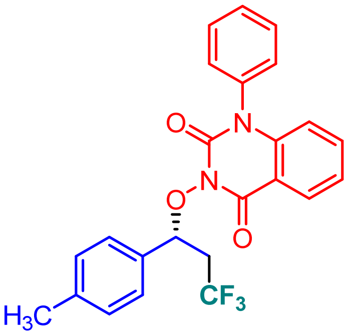

(*R*)-**12c**

Data for **12c**: 104.5 mg, 80% yield, 24h: ^{1}H NMR (400 MHz, $CDCl_3$) δ 8.17 (dd, *J* = 7.9, 1.1 Hz, 1H), 7.60-7.11 (m, 11 H), 6.47 (d, *J* = 8.3 Hz, 1H), 5.83 (t, *J* = 6.4 Hz, 1H), 3.26-3.13 (m, 1H), 2.94-2.80 (m, 1H), 2.32 (s, 3H); ^{13}C NMR (100 MHz, $CDCl_3$) δ 159.0, 148.9, 140.6, 139.6, 135.7, 134.7, 132.5, 130.4, 129.6, 129.0, 128.9, 128.6, 125.3 ($^1J_{C\text{-}F}$ = 276.9 Hz), 123.4, 115.5, 114.9, 81.1, 38.8 ($^2J_{C\text{-}F}$ = 28.6 Hz), 21.2; ^{19}F NMR (471 MHz, $CDCl_3$) δ -63.1 (s, 3F); TLC R_f 0.27 (EtOAc/hexanes, 1/6); HRMS (FD) Calcd for

$C_{24}H_{19}F_3N_2O_3$: 440.1353, found: 440.1340; HPLC analysis form racemic product: t_R 32.93 min (*R*), 39.22 min (*S*) (Chiralpak AS-H, *i*-PrOH/*n*-hexane, 2/98, 1.0 mL/min, λ = 254 nm); From the reaction catalyzed by **V(O)-1**: t_R 32.34 min (major, 74%), 38.27 min (minor, 26%); $[\alpha]_D^{22}$ +112.9 (*c* 1.0, $CHCl_3$) for 48% ee (*R*); From the reaction catalyzed by **V(O)-2** in EtOH (64% yield, 96 h): t_R 33.4 min (minor, 13.2%), 38.3 min (major, 86.8%) for 74% ee (*S*).

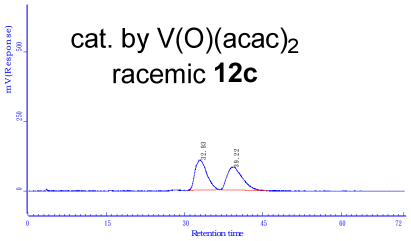

#	Peak name	ax/min (mim)	Area	Area %	Assay %
0		32.927	17103.26	50.09	0.00
1		39.217	17043.03	49.91	0.00

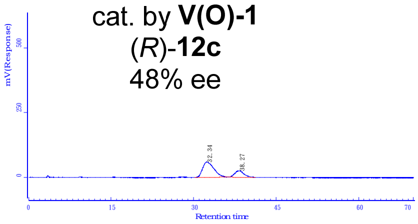

#	Peak name	ax/min (mim)	Area	Area %	Assay %
0		32.339	8469.19	73.95	0.00
1		38.269	2982.93	26.05	0.00

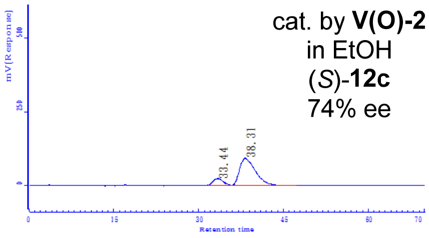

#	Peak name	ax/min (mim)	Area	Area %	Assay %
0		33.441	2686.82	13.22	0.00
1		38.313	17637.39	86.78	0.00

(*R*)-1-Benzyl-3-(1-(3-bromophenyl)-3,3,3-trifluoropropoxy)quinazoline-2,4(1H,3H)-dione 13a

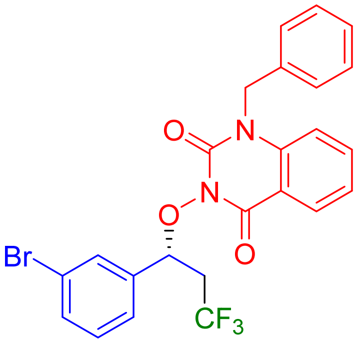

13a

White solid; Yield 96/82% (198.6 mg for 0.4 mmol scale in EtOH or *i*-PrOH, 26h/44h); Data for **13a**: ^{1}H NMR (400 MHz, $CDCl_3$) δ 8.17 (dd, *J* = 7.8, 1.4 Hz 1H), 7.67 (t, *J* = 1.8 Hz, 1H), 7.54-7.49 (m, 3H), 7.33-7.28 (m, 3H), 7.25-7.20 (m, 2H), 7.04-7.01 (m, 3H), 5.82 (t, *J* = 6.4 Hz, 1H), 5.39 (d, *J* = 16.4 Hz, 1H), 5.16 (d, *J* = 16.4 Hz, 1H), 3.27-3.14 (m, 1H), 2.91-2.78 (m, 1H); ^{13}C NMR (100 MHz, $CDCl_3$) δ 159.0, 149.8, 139.2, 138.1, 135.5, 135.1, 133.0, 131.9, 130.2, 129.2, 129.2, 128.0, 127.4, 126.4, 125.2 (q, $^1J_{C\text{-}F}$ = 275.5 Hz), 123.7, 122.5, 115.6, 114.8, 80.7, 47.5, 39.2 (q, $^2J_{C\text{-}F}$ = 28.8 Hz); ^{19}F NMR (471 Hz, $CDCl_3$) δ -63.1 (s, 3F); TLC R_f 0.25 (EtOAc/hexanes, 1/4); HRMS (FD) Calcd for $C_{24}H_{18}BrF_3N_2O_3$: 518.0447, found: 518.0455.

For racemic synthesis catalyzed by $V(O)(acac)_2$ in CH_3CN: 26% yield (53.6 mg, 17h): HPLC analysis from racemic product: t_R 15.8min (*R*), 23.3 min (*S*) (Chiralpak AS-H, *i*-PrOH/hexane, 10/90, 1.0 ml/min, λ = 254 nm); For the reaction catalyzed by **V(O)-1**: t_R 16.8 min (major, 91.9%), 25.3 min

(minor, 8.1%); $[\alpha]_D^{25}$ +108.0 (*c*, 1.0, $CHCl_3$) for 84% ee (*R*); X-ray crystallographic analysis was done for recrystallized (*R*)-**13a** (95% ee) from a mixed solvent of *n*-hexane/acetone (1/2.5).

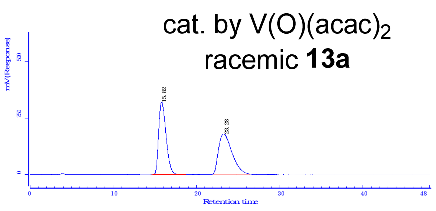

#	Peak name	ax/min (mim)	Area	Area %	Assay %
0		15.817	19677.88	49.91	0.00
1		23.281	19747.66	50.09	0.00

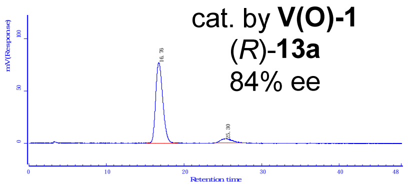

#	Peak name	ax/min (mim)	Area	Area %	Assay %
0		16.761	4918.59	91.85	0.00
1		25.302	436.28	8.15	0.00

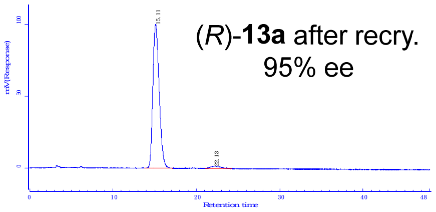

#	Peak name	ax/min (mim)	Area	Area %	Assay %
0		15.105	5285.54	97.73	0.00
1		22.130	122.92	2.27	0.00

For the reaction catalyzed by **V(O)-2**: Yield 45% (93 mg, 120h); t_R 15.3 min (minor, 13.1%), 22.6 min (major, 86.9%) for 74% ee (*S*).

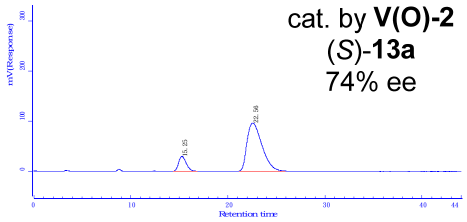

#	Peak name	ax/min (mim)	Area	Area %	Assay %
0		15.250	1535.81	13.14	0.00
1		22.556	10156.28	86.86	0.00

(*R*)-1-Benzyl-3-(1-(3-chlorophenyl)-3,3,3-trifluoropropoxy)quinazoline-2,4(1H,3H)-dione 13a′

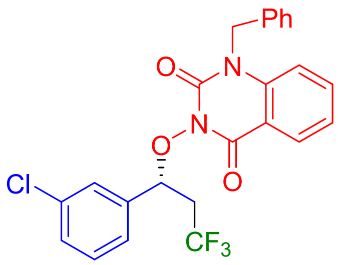

13a'

White solid; Yield: 82.5% (156.7 mg, 65h); ^1H NMR (400 MHz, $CDCl_3$) δ 8.17 (dd, *J* = 7.8, 1.4 Hz 1H), 7.53-7.49 (m, 3H), 7.46 (d, *J* = 7.6 Hz, 1H), 7.36-7.24 (m, 5H), 7.21 (t, *J* = 7.8 Hz, 2H), 7.04-7.01 (m, 3H), 5.83 (t, *J* = 6.4 Hz, 1H), 5.39 (d, *J* = 16.4 Hz, 1H), 5.16 (d, *J* = 16.4 Hz, 1H), 3.27-3.14 (m, 1H), 2.92-2.79 (m, 1H); ^{13}C NMR (100 MHz, $CDCl_3$) δ 158.8, 149.6, 139.0, 137.7, 135.3, 135.0, 134.3, 129.9, 129.8, 129.0, 128.9, 128.8, 127.8, 126.8, 126.2, 125.0 (q, $^1J_{C\text{-}F}$ = 275.4 Hz), 123.5, 115.4, 114.6, 80.6 (q, $^3J_{C\text{-}F}$ = 2.5 Hz), 47.3, 39.0 (q, $^2J_{C\text{-}F}$ = 28.8 Hz); ^{19}F NMR (471 MHz, $CDCl_3$) δ -63.1 (s, 3F); R_f 0.25 (EtOAc/Hexanes, 1/4); HRMS (FD) $[M]^+$ Calcd for $C_{24}H_{18}F_3N_2O_3Cl$: 474.0953, found: 474.0949.

For racemic synthesis catalyzed by $V(O)(acac)_2$ in CH_3CN: 23% yield (43.7 mg, 18h); HPLC analysis from racemic product: t_R 12.22 min (*R*), 17.92 min (*R*) (Chiralpak AS-H, *i*-PrOH/hexane, 10/90, 1.2 ml/min, λ = 254 nm); For the reaction catalyzed by **V(O)-1**: t_R 12.11 min (major, 92.3%),

17.65 min (minor, 9.7%); $[\alpha]_D^{30}$ +112.6 (*c*, 1.0, $CHCl_3$) for 81% ee (*R*).

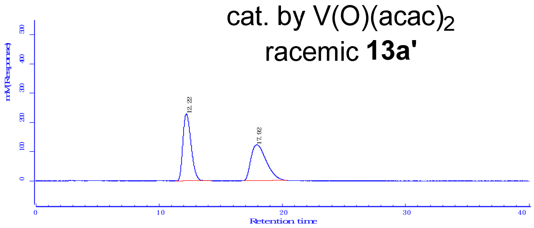

#	Peak name	ax/min (mim)	Area	Area %	Assay %
0		12.216	10581.44	49.98	0.00
1		17.921	10591.89	50.02	0.00

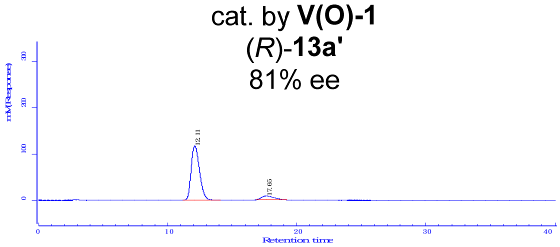

#	Peak name	ax/min (mim)	Area	Area %	Assay %
0		12.111	5253.58	90.29	0.00
1		17.652	565.20	9.71	0.00

(*R*)-1-Benzyl-3-(3,3,3-trifluoro-1-(3-(trifluoromethyl)phenyl)propoxy)quinazoline-2,4(1H,3H)-dione 13b

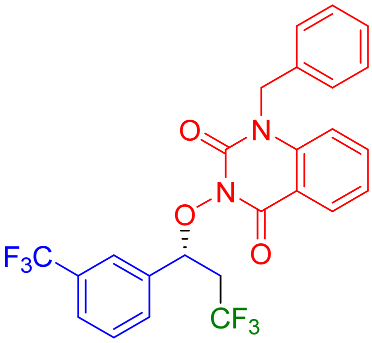

13b

White solid; Yield 88% (178.3 mg, 46h); Data for **13b**: ^{1}H NMR (400 MHz, $CDCl_3$) δ 8.16 (dd, *J* = 7.9, 1.1 Hz, 1H), 7.80-7.83 (m, 2H), 7.63 (d, *J* = 7.9 Hz, 1H), 7.53-7.48 (m, 2H), 7.30-7.24 (m, 3H), 7.21 (t, *J* = 7.6 Hz, 1H), 7.05-7.01 (m, 3H), 5.91 (t, *J* = 6.7 Hz, 1H), 5.35 (d, *J* = 16.4 Hz, 1H), 5.19 (d, *J* = 16.4 Hz, 1H), 3.30-3.20 (m, 1H), 2.94-2.84 (m, 1H); ^{13}C NMR (100 MHz, $CDCl_3$) δ 158.8, 149.6, 139.0, 136.8, 135.4, 134.9, 132.0, 130.8 (q, $^2J_{C-F}$ = 32.4 Hz), 129.0, 129.0, 127.8, 126.5 (q, $^3J_{C-F}$ = 3.3 Hz), 126.1, 125.5 (q, $^3J_{C-F}$ = 3.3 Hz), 125.0 (q, $^1J_{C-F}$ = 275.4 Hz), 123.7 (q, $^1J_{C-F}$ = 271.0 Hz), 123.5, 115.3, 114.7, 80.6 (q, $^3J_{C-F}$ = 2.3 Hz), 47.4, 39.0 (q, $^2J_{C-F}$ = 28.9 Hz); ^{19}F NMR (471 Hz, $CDCl_3$) δ -62.69 (s, 3F), -63.02 (s, 3F); TLC R_f 0.25 (EtOAc/hexanes, 1/5); HRMS (FD) Calcd for $C_{25}H_{18}N_2O_3F_6$: 508.1216, found: 508.1213; HPLC analysis from racemic product: t_R 11.4 min (*R*), 15.9 min (*S*) (Chiralpak AS-H, *i*-PrOH/hexane, 7/93, 1.0 ml/min, λ = 254 nm). For the reaction catalyzed by **V(O)-1**: t_R 10.8 min (major, 91.0%), 15.2 min (minor, 9.0%); $[\alpha]_D^{30}$ +119.9 (*c*, 1.0, $CHCl_3$) for 82% ee (*R*). For racemic synthesis catalyzed by $V(O)(acac)_2$ in CH_3CN: 27% yield (54.9 mg, 20h).

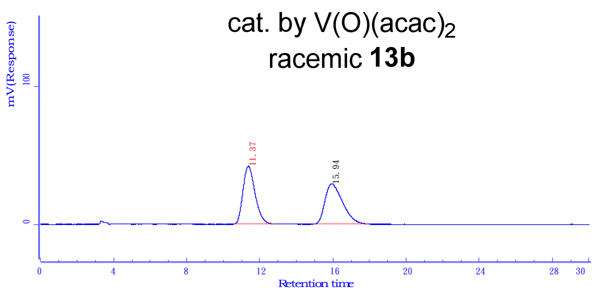

#	Peak name	ax/min (mim)	Area	Area %	Assay %
0		11.369	1938.19	50.00	0.00
1		15.937	1937.94	50.00	0.00

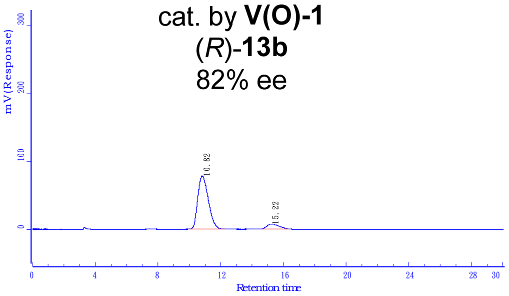

#	Peak name	ax/min (mim)	Area	Area %	Assay %
0		10.818	3640.50	90.98	0.00
1		15.222	361.05	9.02	0.00

Methyl (*R*)-3-(1-((1-Benzyl-2,4-dioxo-1,4-dihydroquinazolin-3(2H)-yl)oxy)-3,3,3-trifluoropropyl) -benzoate 13b'

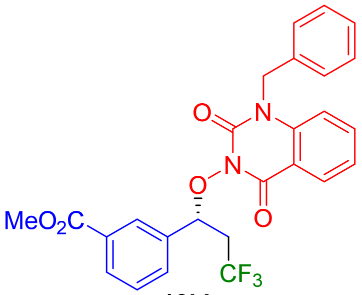

13b'

White solid; Yield 90% (180.1 mg, 46h); Data for **13b'**: ^{1}H NMR (400 MHz, $CDCl_3$) δ 8.16-8.13 (m, 2H), 8.05 (d, *J* = 7.8 Hz, 1H), 7.81 (d, *J* = 7.8 Hz, 1H), 7.51-7.42 (m, 2H), 7.26-7.24 (m, 3H), 7.18 (t, *J* = 7.6 Hz, 1H), 7.01 (d, *J* = 8.4 Hz, 1H), 6.97-6.95 (m, 2H), 5.90 (t, *J* = 6.6 Hz, 1H), 5.36 (d, *J* = 16.4 Hz, 1H), 5.11 (d, *J* = 16.4 Hz, 1H), 3.87 (s, 3H), 3.22-3.19 (m, 1H), 2.97-2.83 (m, 1H); ^{13}C NMR (100 MHz, $CDCl_3$) δ 166.3, 158.7, 149.5, 138.9, 136.1, 135.3, 134.9, 133.1, 130.9, 130.3, 129.8, 128.9, 128.6, 127.7, 126.1, 125.2 (q, $^1J_{C-F}$ = 275.3 Hz), 123.4, 115.3, 114.6, 80.8, 52.1, 47.2, 38.9 (q, $^2J_{C-F}$ = 28.8 Hz); ^{19}F NMR (471 Hz, $CDCl_3$) δ -62.99 (s, 3F); TLC R_f 0.25 (EtOAc/hexanes, 1/2); HRMS (FD) $[M]^+$ Calcd for $C_{26}H_{21}N_2O_5F_3$: 498.1398, found: 498.1397; HPLC analysis from racemic product: t_R 14.7 min (*R*), 18.4 min (*S*) (Chiralpak AS-H, *i*-PrOH/hexane, 15/85, 1.2 ml/min, λ = 254 nm). For the reaction catalyzed by **V(O)-1**: t_R 14.8 min (major, 90.4%), 18.5 min (minor, 9.6%); $[\alpha]_D^{30}$ +137.1 (*c*, 1.0, $CHCl_3$) for 81% ee (*R*). For racemic synthesis catalyzed by V(O)(acac)2 in CH_3CN: 28% yield (55.8 mg, 17h).

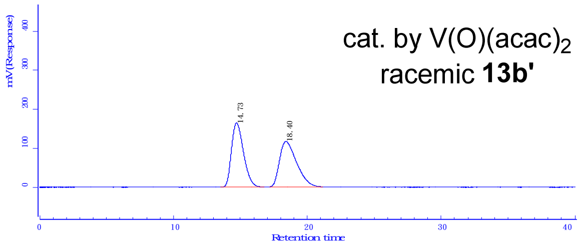

#	Peak name	ax/min (mim)	Area	Area %	Assay %
0		14.725	10274.04	49.96	0.00
1		18.399	10289.90	50.04	0.00

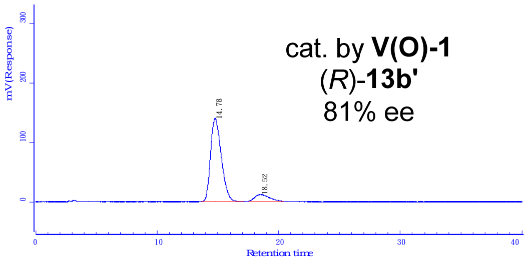

#	Peak name	ax/min (mim)	Area	Area %	Assay %
0		14.779	8581.88	90.44	0.00
1		18.523	906.99	9.56	0.00

3-(1-(3-bromophenyl)-3,3,3-trifluoropropoxy)-2-thioxo-2,3-dihydroquinazolin-4(1H)-one 14a

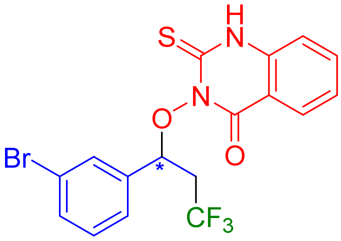

14a

White solid; Yield 4.3% (7.4 mg for 0.4 mmol scale in acetone/*i*-PrOH 0.5/4 mL, 48 h); Data for **14a**: ^{1}H NMR (400 MHz, $CDCl_3$) δ 9.83 (s, 1H), 8.09 (d, J = 7.8 Hz, 1H), 7.71 (t, J = 1.6 Hz, 1H), 7.67-7.63 (m, 1H), 7.49-7.45 (m, 2H), 7.26 (t, J = 7.8 Hz, 1H), 7.21 (t, J = 7.8 Hz, 1H), 7.09 (d, J = 8.0 Hz, 1H), 5.71 (t, J = 6.6 Hz, 1H), 3.26-3.13 (m, 1H), 2.89-2.76 (m, 1H); ^{13}C NMR (100 MHz, $CDCl_3$) δ 159.4, 149.9, 138.2, 137.5, 135.5, 132.9, 131.4, 130.0, 128.5, 127.0, 125.0 (q, $^{1}J_{C\text{-}F}$ = 275.3 Hz), 123.9, 122.4, 115.4, 114.6, 81.1, 39.2 (q, $^{2}J_{C\text{-}F}$ = 28.8 Hz); ^{19}F NMR (471 Hz, $CDCl_3$) δ -63.1 (s, 3F); TLC R_f 0.25 (EtOAc/hexanes, 1/2); HRMS (FD) Calcd for $C_{17}H_{12}BrF_3N_2O_2S$: 443.9798, found: 443.9803; HPLC analysis from racemic product: t_R 12.7 min (*S*), 18.3 min (*R*) (Chiralpak AD-H, *i*-PrOH/hexane, 14/86, 0.8 ml/min, λ = 254 nm). For the reaction catalyzed by **V(O)-1**: t_R 12.7 min (minor, 20.0%), 18.3 min (major, 80.0%) for 60% ee (*R*); $[\alpha]_D^{25}$ +90.0 (*c*, 0.06, $CHCl_3$) for 60% ee (*R*). For racemic synthesis catalyzed by $V(O)(acac)_2$ in CH_3CN: 7.4% yield (13.2 mg, 30h).

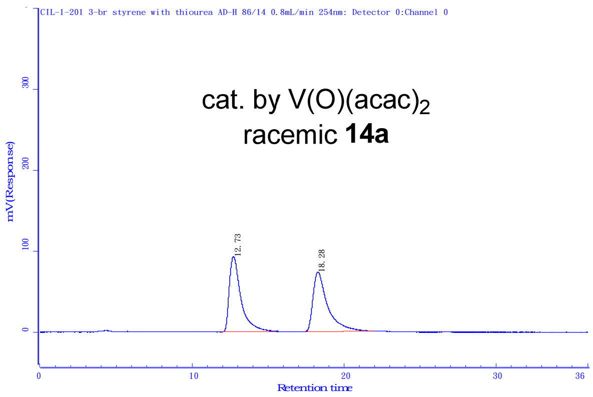

#	Peak name	ax/min (mim)	Area	Area %	Assay %
0		12.731	4989.25	50.12	0.00
1		18.277	4965.15	49.88	0.00

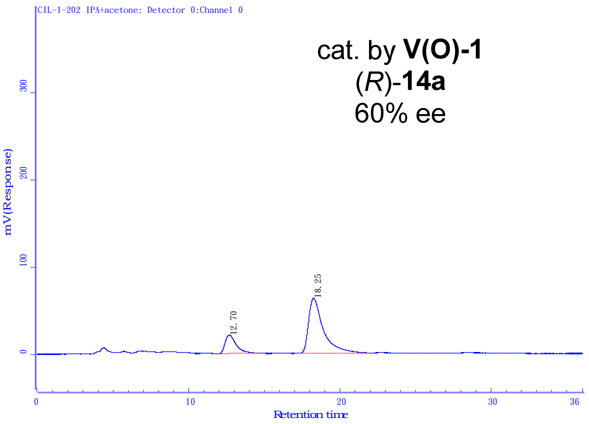

#	Peak name	ax/min (mim)	Area	Area %	Assay %
0		12.704	1058.35	19.99	0.00
1		18.254	4237.18	80.01	0.00

Figure S1: X-ray crystal structure of (*R*)-**12a** (CCDC 2215699) in Platon drawing (ellipsoids are shown at 50% probability level)

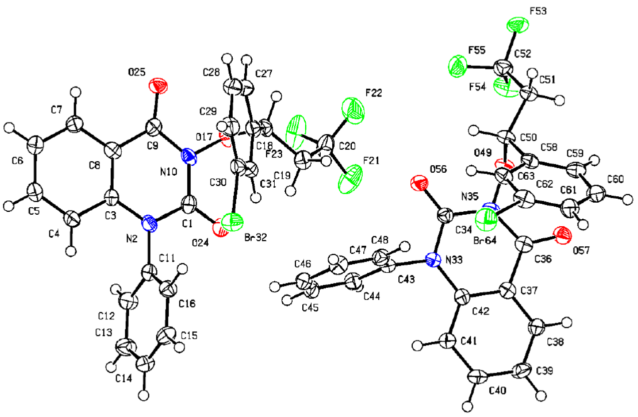

Table S1 Crystal data and structure refinement for (*R*)-12a (2208117lt2_auto).

Identification code	2208117lt2_auto
Empirical formula	$C_{46}H_{32}Br_2F_6N_4O_6$
Formula weight	1010.57
Temperature/K	100.01(10)
Crystal system	monoclinic
Space group	$P2_1$
a/Å	10.22560(11)
b/Å	20.67971(18)
c/Å	10.38564(11)
α/°	90
β/°	110.7384(12)

γ/°	90
Volume/Å^3	2053.88(4)
Z	2
ρ_{calc}g/cm^3	1.634
μ/mm^{-1}	3.239
F(000)	1016.0
Crystal size/mm^3	0.14 × 0.09 × 0.03
Radiation	Cu Kα (λ = 1.54184)
2Θ range for data collection/°	8.552 to 134.146
Index ranges	-11 ≤ h ≤ 12, -24 ≤ k ≤ 24, -12 ≤ l ≤ 12
Reflections collected	23889
Independent reflections	7328 [R_{int} = 0.0235, R_{sigma} = 0.0223]
Data/restraints/parameters	7328/1/577
Goodness-of-fit on F^2	1.031
Final R indexes [I>=2σ (I)]	R_1 = 0.0378, wR_2 = 0.1058
Final R indexes [all data]	R_1 = 0.0384, wR_2 = 0.1065
Largest diff. peak/hole / e Å^{-3}	1.99/-0.55
Flack parameter	-0.016(8)

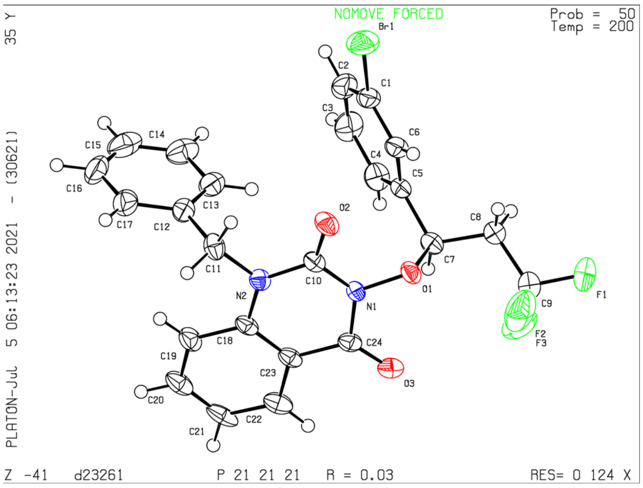

Figure S2. X-ray crystal structure of (*R*)-**13a** (CCDC 2094844) in Platon drawing (ellipsoids are shown at 50% probability level)

Selected data for X-ray crystal structure of (*R*)-**13a**

Crystal data and structural refinement for (*R*)-13a: $C_{24}H_{18}BrF_3N_2O_3$, Mr = 519.3, prism, space group P 21 21 21, a = 9.0260(3) Å, b = 13.0929(5) Å, c = 18.3842(7) Å, α = 90.0, β = 90, γ = 90, V = 2172.58(14) Å^3, Z = 4, ρ_{calcd} = 1.588 Mgm^{-3}, T = 200(2) K, Bruker Kappa CCD diffractometer, Mo K_α radiation (λ = 0.71073), μ = 1.948 mm^{-1}. The structure was solved by SHELXL-86 (Sheldrick, 1986) Solver. All non-hydrogen atoms were refined anisotropically (SHELXS-97; Sheldrick, 1997). Final block-diagonal matrix least-square refinement on F^2 with all 3835 reflections and 298 variables converged to $R1$ ($I > 2\sigma(I)$) = 0.0327, $wR2$ (all data) = 0.0759 and GOF = 0.940. Absolute structure parameter 0.023(8).

EGFR-TK inhibition results:

Preliminary EGFR-TK inhibition test results for our compounds at single point analyses at 1 μM concentrations are attached at the end. These results are compiled as a Table in SI as shown below: Project #: SSBK13429_63766 (samples 1-15; on Oct. 28); SSBK13429_64251 (sample 1 on Dec. 16); Test Compound Concentration: 1,000 nM; [ATP] Tested: Km app

Kinase Tested: EGFR (ErbB1); Kinase lot#: PV3872/2281133

Table S2: EGFR-TK inhibition results

Sample name	structure	Sample number in the paper	% Inhibition Point 1/point 2	% inhibition mean
sample1	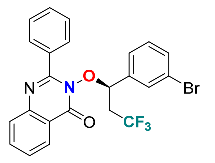	(*R*)-**9a**<br>(*S*)-**9a**	-3/-2	-3<br>-3
sample2	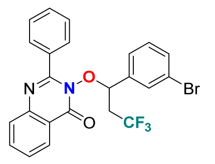	Racemic-**9a**	0/-5	-3
sample3	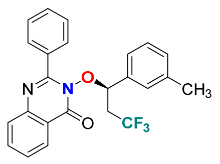	(*R*)-**9b**<br>(*S*)-**9b**	-4/-6	-5<br>-10

sample4	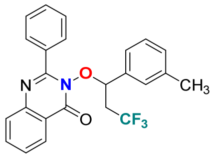	Racemic-**9b**	-8/-7	-8
sample5	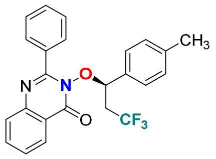	(*R*)-**9c**<br>(*S*)-**9c**	-2/-2	-2<br>-2
sample6	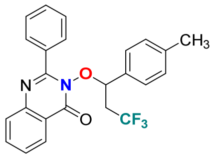	Racemic-**9c**	-3/-2	-2
sample7	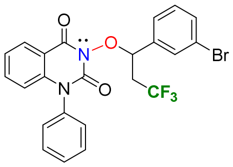	Racemic-**12a**	0/-1	-1
sample8	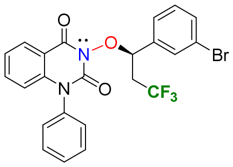	(*R*)-**12a**<br>(*S*)-**12a**	-5/-3	-4<br>2 (predicted)
sample9	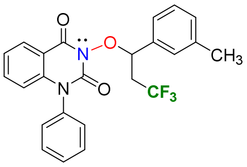	Racemic-**12b**	-5/-5	-5
sample10	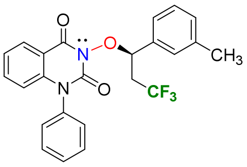	(*R*)-**12b**<br>(*S*)-**12b**	-17/1<br>----	-8<br>-2 (predicted)
sample11	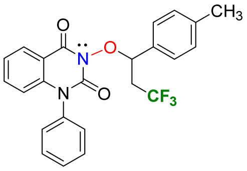	Racemic-**12c**	-5/-4	-4
sample12	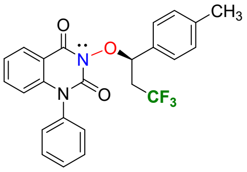	(*R*)-**12c**<br>(*S*)-**12b**	-6/-3	-4<br>-4

sample13	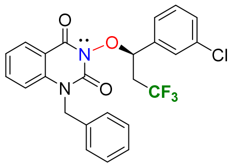	(*R*)-**13a'**	-4/-2	-3
sample14	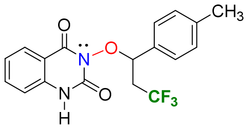	Racemic-**11a'**	-4/1	-1
sample15	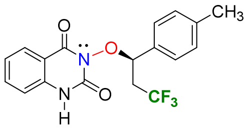	(*R*)-**11a'**<br>(*S*)-**11a'**	-4/2	-1<br>-1
sample 1	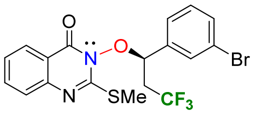	(*R*)-**10a**	3/5	4

The data in green are calculated from the data of the (*R*) and racemic products In general, EGFR-TK inhibition activity profile is in the order of (*S*) 1-Ph (IC_{50}: 25 μM) > 2-H > 2-Ph in the quinazoline moieties and in the order of *m*-Cl/Br > *p*-Me > *m*-Me in the styrene substrate units. Notably, (*R*)-**10a** (2-SMe and *m*-Br) showed discernible EGFR-TK inhibition activity whose IC_{50} is estimated to be around 10-12.5 μM.

Experimental References:

(S1) (a) Deore, R. R.; Chen, G. S.; Chang, P.-T.; Chern, T.-R.; Lai, S.-Y.; Chuang, M.-H.; Lin, J.-H.; Kung, F.-L.; Chen, C.-S.; Chiou, C.-T.; Chern, J.-W. Discovery of *N*-Arylalkyl-3-hydroxy-4-oxo-3,4-dihydroquinazolin-2-carboxamide Derivatives as HCV NS5B Polymerase Inhibitors. *ChemMedChem* **2012**, *7*, 850–860. (b) Tanaka, K.; Matsuo, K.; Nakanishi, A.; Kataoka, Y.; Takase, K.; Otsuki, S. *Chem. & Pharm. Bull.* **1988**, *36*, 2323–2330.

(S2) Cheng, R.; Guo, T.; Zhang-Negrerie, D.; Du, Y.; Zhao, K. One-Pot Synthesis of Quinazolinones From Anthranilamides And Aldehydes via *P*-Toluenesulfonic Acid Catalyzed Cyclocondensation and Phenyliodine Diacetate Mediated Oxidative Dehydrogenation. *Synthesis* **2013**, *45*, 2998–3006.

(S3) (a) Falsini, M.; Squarcialupi, L.; Catarzi, D.; Varano, F.; Betti, M.; Di Cesare Mannelli, L.; Tenci, B.; Ghelardini, C.; Tanc, M.; Angeli, A.; Supuran, C. T.; Colotta, V. 3-Hydroxy-1H-quinazoline-2,4-

dione as a new scaffold to develop potent and selective inhibitors of the tumor-associated carbonic anhydrases IX and XII. *J. Med. Chem.* **2017**, *60*, 6428–6439. (b) Kang, D.; Zhang, H.; Zhou, Z.; Huang, B.; Naesens, L.; Zhan, P.; Liu, X. First Discovery of Novel 3-Hydroxy-Quinazoline-2,4(1H,3H)-Diones as Specific Anti-Vaccinia And Adenovirus Agents via 'Privileged Scaffold' Refining Approach. *Bioorg. Med. Chem. Lett.* **2016**, *26*, 5182–5186.

(S4) (a) Geffken, D.; Koellner, M. A. Oxalylierung Von *N*-Phenylanthranilo-*O*-Alkylhydroxamsäuren zu 4-Alkoxy-2,3,4,5-Tetrahydro-1H-1,4-Benzodiazepin-2,3,5-Trionen. Z. *fur Naturforsch. – B: Chem*. Sci. **2005**, *60*, 337–340. (b) Domagala, John Michael; Ellsworth, Edmund L.; Huang, L.; Renau, T. E.; Singh, R.; Stier, M. A. WO9921840 A1 **1999**.

(S5) Tang, J.; Maddali, K.; Dreis, C. D.; Sham, Y. Y.; Vince, R.; Pommier, Y.; Wang, Z. 3-Hydroxy-pyrimidine-2,4-diones as an Inhibitor Scaffold of HIV Integrase. *J. Med. Chem*. **2011**, *2*, 63−67.

(S6) Khokhlov, P. S.; Osipov, V. N.; Roshchin, A.V. 3-Hydroxy- and 3-Alkoxy-2-Sulfanylquinazolin-4(3*H*)-Ones: Synthesis and Reactions with Alkylating and Acylating Agents. *Russ. Chem. Bull.* **2011**, *60*, 153–156.

Theoretical calculation:

Optimized geometry: The structure of a chemical sample corresponding to an energy minimum by using Scigress program. The structure of the chemical sample is refined by performing an optimize geometry calculation in Mechanics using Augmented MM3 parameters. The sandwich structure optimizations were done by performing an optimize geometry calculation in Mechanics using Augmented MM2 parameters.

Representative Cartesian coordinates

Figure S4

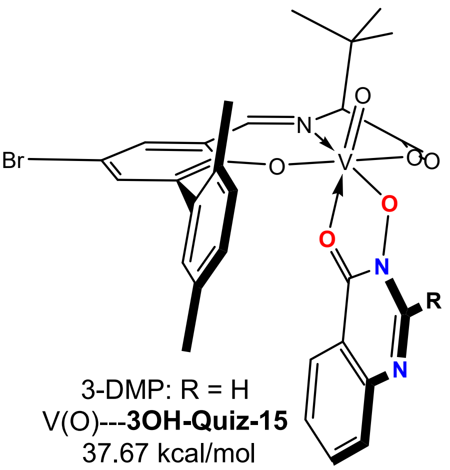

3-DMP: R = H
V(O)---**3OH-Quiz-15**
37.67 kcal/mol

Total number of atoms: 67 for 3-DMP: R = H: **V(O)---3OH-Quiz-15**

O	1	6.73873	-6.11915	-2.10071
Br	2	3.88336	-8.26748	-9.22057
V	3	5.55584	-5.10638	-2.78859
O	4	4.74563	-4.81818	-1.03396
O	5	2.73769	-5.11288	-0.073871
O	6	6.39121	-5.39113	-4.52495
N	7	4.24996	-6.56281	-2.92489
H	8	5.852	-8.29955	-1.2635
H	9	2.35456	-9.28569	-2.24777
H	10	2.38779	-6.76036	-2.07229
H	11	3.09999	-7.8136	-4.0403
H	12	3.19443	-8.18126	-6.34802
H	13	5.42615	-7.32532	0.18678
H	14	2.97377	-7.3866	1.04438
H	15	1.66333	-7.98343	-0.029932
H	16	2.77054	-9.13702	0.774775
H	17	4.10056	-9.41344	-2.66628
H	18	3.46182	-10.2591	-1.23456

H	19	6.19221	-6.42207	-8.89175
H	20	5.25227	-9.10562	0.212011
C	21	3.59611	-5.52296	-0.822372
C	22	5.12598	-8.20318	-0.427088
C	23	2.72266	-8.14558	0.27112
C	24	3.38269	-9.31685	-1.82225
C	25	3.95095	-7.11369	-4.03839
C	26	4.62273	-6.88652	-5.34354
C	27	3.6782	-8.0794	-0.944277
C	28	3.45169	-6.75014	-1.73742
C	29	4.08148	-7.53685	-6.46117
C	30	4.64213	-7.37893	-7.72423
C	31	5.75126	-6.56021	-7.89083
C	32	6.31486	-5.91395	-6.79087
C	33	5.76929	-6.09106	-5.50975
O	34	4.258	-3.8774	-3.57387
O	35	6.71952	-3.55148	-2.59304
C	36	4.6695	-2.74358	-3.61814
N	37	5.92263	-2.4335	-3.15244
C	38	7.47458	-5.01892	-7.00436
C	39	6.35527	-1.09525	-3.24398
C	40	4.49912	-0.339831	-4.14949
C	41	3.89948	-1.60773	-4.15365
C	42	3.76651	0.73737	-4.65308
C	43	2.47318	0.557795	-5.14737
C	44	1.89138	-0.70907	-5.14352
C	45	2.60503	-1.79728	-4.64483
H	46	7.37567	-0.942876	-2.85604
H	47	4.21717	1.74361	-4.65808
H	48	1.90955	1.42079	-5.54059
H	49	0.868394	-0.849741	-5.53183
H	50	2.14642	-2.80031	-4.63705
N	51	5.68208	-0.114375	-3.70937
C	52	7.29566	-3.85883	-7.76375
C	53	8.34269	-2.96225	-7.98823
C	54	9.59455	-3.24314	-7.44061
C	55	9.79019	-4.40125	-6.69104
C	56	8.74199	-5.30137	-6.46908

C	57	8.10482	-1.71742	-8.80445
H	58	10.4378	-2.55187	-7.60355
C	59	9.02943	-6.56034	-5.68687
H	60	6.30066	-3.63662	-8.18324
H	61	10.7891	-4.61089	-6.27381
H	62	7.89738	-1.97553	-9.86683
H	63	7.23247	-1.14568	-8.41637
H	64	8.98177	-1.03276	-8.79149
H	65	8.32179	-7.38275	-5.9306
H	66	8.96754	-6.36925	-4.59251
H	67	10.0514	-6.94551	-5.90026

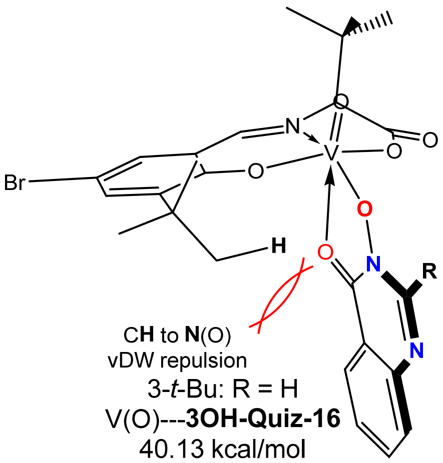

Total number of atoms: 63; for 3-*t*-Bu: R = H: **V(O)---3OH-Quiz-16**

O	1	-9.659336	1.797626	2.127046
Br	2	-16.811954	2.813393	-1.338457
V	3	-10.024867	0.893162	0.732303
O	4	-8.194422	1.113654	0.081715
O	5	-7.132976	2.020316	-1.678004
O	6	-11.844519	0.703528	1.406472
N	7	-10.377574	2.494479	-0.337559
C	8	-8.043286	2.069887	-0.881647
C	9	-8.322933	4.41988	1.090048
C	10	-7.586466	5.032671	-1.221773
C	11	-9.918575	5.505432	-0.490072
C	12	-11.558538	2.820453	-0.698855
C	13	-12.830293	2.196559	-0.248407
C	14	-8.766082	4.480191	-0.386292
C	15	-9.201025	3.079991	-0.931968

C	16	-14.000412	2.661073	-0.859163
C	17	-15.241238	2.160993	-0.491969
C	18	-15.331261	1.183259	0.4902
C	19	-14.183162	0.690297	1.128053
C	20	-12.924117	1.222132	0.763367
O	21	-10.431136	-0.2242	-0.816365
O	22	-9.571693	-0.768099	1.652041
C	23	-10.23081	-1.395096	-0.601871
N	24	-9.779994	-1.815812	0.6242
C	25	-14.321308	-0.391773	2.203636
C	26	-9.54327	-3.192647	0.810356
C	27	-10.145026	-3.782419	-1.221053
C	28	-10.43529	-2.462169	-1.595965
C	29	-10.331252	-4.792923	-2.166791
C	30	-10.798673	-4.498513	-3.449008
C	31	-11.08865	-3.181832	-3.80368
C	32	-10.905783	-2.158026	-2.876093
N	33	-9.716254	-4.112544	-0.058693
C	34	-13.739633	0.089862	3.555184
C	35	-15.791765	-0.787494	2.494914
C	36	-13.608381	-1.691682	1.761671
H	37	-11.131097	-1.114866	-3.154888
H	38	-9.170927	4.164301	1.761477
H	39	-7.517525	3.670506	1.255652
H	40	-7.929108	5.402194	1.434899
H	41	-7.825196	5.050329	-2.308903
H	42	-6.660305	4.431448	-1.090322
H	43	-7.330581	6.075302	-0.927649
H	44	-10.301892	5.592685	-1.53123
H	45	-10.774748	5.241586	0.169406
H	46	-9.586818	6.522188	-0.182268
H	47	-11.648488	3.64261	-1.426859
H	48	-9.435288	3.225341	-2.012648
H	49	-13.947015	3.435373	-1.641435
H	50	-16.332149	0.807498	0.748641
H	51	-10.104255	-5.837379	-1.895776
H	52	-10.939057	-5.309802	-4.183485
H	53	-11.459296	-2.95002	-4.816486

H	54	-14.210108	1.044753	3.880884
H	55	-12.641897	0.257104	3.525659
H	56	-13.91854	-0.655018	4.362384
H	57	-16.394094	0.074979	2.860064
H	58	-15.850713	-1.567316	3.287038
H	59	-16.298332	-1.214447	1.60007
H	60	-14.000322	-2.057975	0.786219
H	61	-12.511018	-1.56617	1.647277
H	62	-13.756559	-2.508144	2.503392
H	63	-9.175706	-3.435738	1.820707

Figure S5 for R = H

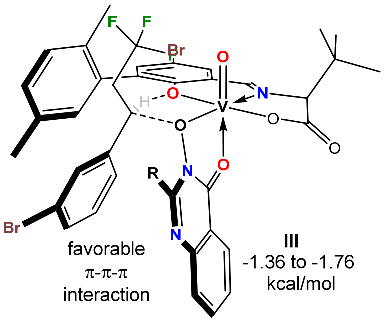

Total number of atoms: 87

C	1	-5.915243	1.654716	-0.123429
F	2	-4.219972	3.084721	0.853296
F	3	-6.330921	3.814627	0.833511
F	4	-5.164406	3.762307	-1.073491
C	5	-5.396961	3.074249	0.11045
C	6	-5.209255	-1.523195	-2.246461
C	7	-5.640979	-2.841375	-2.400084
C	8	-6.529722	-3.39596	-1.480361
C	9	-6.977025	-2.61477	-0.413628
C	10	-6.534319	-1.297151	-0.266652
C	11	-5.130677	0.736701	-1.046837
C	12	-5.633888	-0.727296	-1.175525
O	13	-2.512363	3.008522	-1.456179
Br	14	-3.885763	1.982587	-8.982528
V	15	-2.221992	1.359263	-1.807802
O	16	-0.582012	1.405857	-0.764667
O	17	1.550357	0.769885	-1.014079
O	18	-3.851356	1.372177	-2.909682

N	19	-0.978024	1.836481	-3.240824
Br	20	-8.191016	-3.344698	0.839911
C	21	0.57227	1.330851	-1.454501
C	22	0.439455	4.368686	-1.947979
C	23	2.606355	3.262997	-2.51421
C	24	1.064546	3.965614	-4.32785
C	25	-1.380037	1.882501	-4.4413
C	26	-2.607014	1.760899	-4.996393
C	27	1.115066	3.367838	-2.90597
C	28	0.447285	1.965094	-2.844928
C	29	-2.656817	1.8912	-6.392706
C	30	-3.854948	1.797678	-7.098669
C	31	-5.03452	1.570184	-6.396209
C	32	-4.994078	1.444126	-5.006214
C	33	-3.797937	1.531715	-4.271995
O	34	-1.868931	-0.539922	-2.102673
O	35	-3.51279	0.699949	-0.4785
C	36	-2.251547	-1.273195	-1.217086
N	37	-3.044041	-0.695229	-0.237004
C	38	-6.174462	1.238622	-4.399799
C	39	-3.419473	-1.462329	0.804685
C	40	-2.408342	-3.26334	-0.011654
C	41	-1.939187	-2.588434	-1.145063
C	42	-2.120479	-4.624293	0.126273
C	43	-1.379639	-5.291969	-0.852001
C	44	-0.918021	-4.605627	-1.977389
C	45	-1.195425	-3.246047	-2.129868
N	46	-3.094126	-2.682599	0.883034
C	47	-6.867286	2.295804	-3.795585
C	48	-8.088523	2.021544	-3.172021
C	49	-8.62503	0.73292	-3.177724
C	50	-7.96032	-0.314431	-3.818256
C	51	-6.731151	-0.04388	-4.425368
C	52	-8.567814	-1.692262	-3.84725
C	53	-6.333077	3.705674	-3.839849
H	54	-7.892202	-2.441526	-4.318347
H	55	-4.882391	1.162736	-2.080831
H	56	-6.902256	-0.723401	0.57176

H	57	-5.986737	1.193449	0.887213
H	58	-6.947081	1.73214	-0.537704
H	59	-4.521606	-1.127934	-2.981567
H	60	-5.292688	-3.432693	-3.238975
H	61	-6.86685	-4.417704	-1.609138
H	62	-0.614294	4.568442	-2.243805
H	63	0.442709	4.002328	-0.896904
H	64	0.96235	5.352095	-1.95106
H	65	3.142196	2.523243	-3.151534
H	66	2.739897	2.963288	-1.451253
H	67	3.131147	4.239106	-2.629262
H	68	1.513968	3.274428	-5.076544
H	69	0.024028	4.204648	-4.642832
H	70	1.629782	4.923645	-4.389177
H	71	-0.547039	2.03335	-5.145631
H	72	1.022305	1.287944	-3.522028
H	73	-1.752899	2.073622	-6.963361
H	74	-5.981725	1.497601	-6.919279
H	75	-2.474434	-5.172248	0.992253
H	76	-1.16172	-6.348062	-0.736875
H	77	-0.343075	-5.130243	-2.732139
H	78	-0.834027	-2.721139	-3.005724
H	79	-8.640527	2.81781	-2.686658
H	80	-9.578297	0.546479	-2.696974
H	81	-6.196482	-0.841162	-4.927229
H	82	-9.516834	-1.672597	-4.429408
H	83	-8.793596	-2.037091	-2.812983
H	84	-6.453053	4.112106	-4.869362
H	85	-6.87544	4.380948	-3.140091
H	86	-5.252254	3.746264	-3.580918
H	87	-4.06846	-1.06664	1.602237

Figure S6:

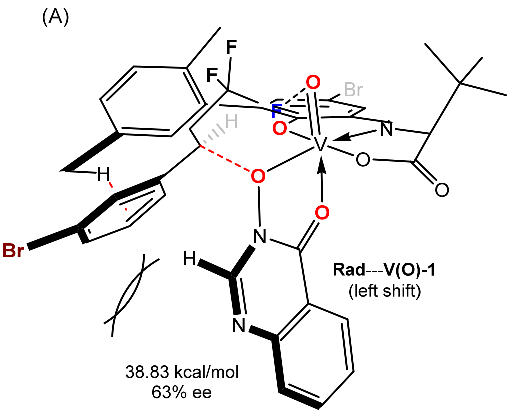

Total number of atoms: 87

C	1	-5.177653	1.902319	1.042077
F	2	-2.970765	2.909252	1.052562
F	3	-4.561733	3.833595	2.310054
F	4	-4.644022	4.059367	0.091208
C	5	-4.331493	3.170257	1.110632
C	6	-5.973427	-0.889321	-1.566698
C	7	-6.737866	-2.047369	-1.699604
C	8	-7.557589	-2.465197	-0.655267
C	9	-7.589864	-1.72671	0.524952
C	10	-6.80275	-0.582883	0.665369
C	11	-5.083438	1.119664	-0.254032
C	12	-5.982646	-0.144036	-0.382744
O	13	-2.761431	2.925458	-1.790743
Br	14	-3.301081	1.169628	-9.419549
V	15	-2.290487	1.313662	-2.063241
O	16	-0.838594	1.473211	-0.766726
O	17	1.390706	1.220263	-0.761856
O	18	-3.713376	1.170239	-3.384536
N	19	-0.932572	1.871712	-3.363285
Br	20	-8.700614	-2.287584	1.9586
C	21	0.39672	1.619056	-1.326402
C	22	-0.109343	4.579535	-2.080081
C	23	2.25156	3.757359	-2.187932
C	24	0.972794	4.136048	-4.285071
C	25	-1.087563	1.780591	-4.630266
C	26	-2.343682	1.462183	-5.361091
C	27	0.848426	3.62989	-2.829231

C	28	0.358597	2.142185	-2.772725
C	29	-2.281726	1.448068	-6.76112
C	30	-3.413909	1.182073	-7.524138
C	31	-4.631179	0.946466	-6.898403
C	32	-4.722228	0.964046	-5.506101
C	33	-3.573215	1.205438	-4.733595
O	34	-1.781574	-0.542542	-2.366042
O	35	-3.483822	0.619146	-0.66319
C	36	-2.205473	-1.278915	-1.510441
N	37	-3.039482	-0.804232	-0.529433
C	38	-6.044128	0.728671	-4.881599
C	39	-3.346966	-1.681433	0.535467
C	40	-2.342338	-3.436572	-0.325137
C	41	-1.86543	-2.708655	-1.42305
C	42	-2.024023	-4.794262	-0.245392
C	43	-1.252361	-5.409337	-1.232775
C	44	-0.781749	-4.669547	-2.316704
C	45	-1.088618	-3.313786	-2.414103
N	46	-3.043967	-2.920323	0.614399
C	47	-6.682435	1.715229	-4.112378
C	48	-7.933028	1.43693	-3.550711
C	49	-8.565891	0.216946	-3.779465
C	50	-7.960782	-0.751359	-4.580561
C	51	-6.69908	-0.484823	-5.1169
C	52	-8.651676	-2.063584	-4.853825
C	53	-6.10414	3.098564	-3.930474
H	54	-7.920858	-2.888453	-5.010155
H	55	-5.295143	1.806348	-1.096858
H	56	-6.84295	-0.029093	1.615678
H	57	-4.885292	1.244283	1.891765
H	58	-6.239852	2.193225	1.21278
H	59	-5.333871	-0.577583	-2.407634
H	60	-6.692131	-2.631391	-2.633885
H	61	-8.170748	-3.374824	-0.766074
H	62	-1.105189	4.629509	-2.572172
H	63	-0.26566	4.26865	-1.023175
H	64	0.287653	5.619461	-2.057201
H	65	2.97502	3.0409	-2.63831

H	66	2.231741	3.574825	-1.090854
H	67	2.673133	4.777919	-2.329002
H	68	1.621894	3.471478	-4.898727
H	69	-0.01652	4.215054	-4.787817
H	70	1.423278	5.152915	-4.322304
H	71	-0.202502	1.978162	-5.255194
H	72	1.101838	1.537734	-3.344429
H	73	-1.327081	1.651092	-7.274045
H	74	-5.53253	0.757733	-7.503058
H	75	-2.387805	-5.387021	0.610518
H	76	-1.010787	-6.483027	-1.152109
H	77	-0.167806	-5.154812	-3.094723
H	78	-0.71965	-2.72558	-3.271195
H	79	-8.44139	2.199773	-2.938387
H	80	-9.558398	0.026392	-3.339068
H	81	-6.207588	-1.24997	-5.739191
H	82	-9.282713	-1.992198	-5.767596
H	83	-9.310847	-2.363984	-4.008732
H	84	-6.905805	3.867318	-3.866801
H	85	-5.507667	3.164804	-2.994903
H	86	-5.446627	3.394635	-4.777104
H	87	-3.938663	-1.208005	1.334146

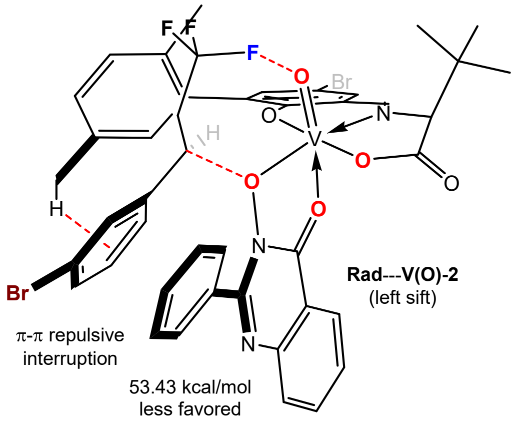

Total number of atoms: 97

C	1	-5.564381	1.485704	0.709957
F	2	-3.364533	2.363022	1.230627
F	3	-5.136449	3.299977	2.206512
F	4	-4.758064	3.662077	0.038043
C	5	-4.696743	2.696945	1.033911

C	6	-5.924359	-1.193834	-2.081838
C	7	-6.686773	-2.320358	-2.386762
C	8	-7.668214	-2.757909	-1.502072
C	9	-7.871647	-2.066	-0.31101
C	10	-7.089233	-0.954618	0.003196
C	11	-5.188298	0.716542	-0.543951
C	12	-6.095687	-0.503854	-0.876449
O	13	-2.757883	2.684904	-1.552092
Br	14	-2.888106	1.54862	-9.390789
V	15	-2.314274	1.107889	-2.007612
O	16	-0.918541	1.067647	-0.642365
O	17	1.311816	0.838822	-0.557352
O	18	-3.670677	1.174605	-3.404236
N	19	-0.891814	1.799739 -	.166957
Br	20	-9.205086	-2.651891	0.906701
C	21	0.340045	1.295696	-1.116277
C	22	-0.145665	4.329488	1.491221
C	23	2.221829	3.537865	-1.635502
C	24	0.997223	4.176736	-3.703648
C	25	-0.972066	1.828202	-4.443873
C	26	-2.180999	1.572638	-5.27172
C	27	0.839212	3.487297	-2.329211
C	28	0.364363	2.002102	-2.482342
C	29	-2.032661	1.64737	-6.662962
C	30	-3.117872	1.44793	-7.509254
C	31	-4.37489	1.194282	-6.97566
C	32	-4.551785	1.126474	-5.592817
C	33	-3.450146	1.299321	-4.737563
O	34	-1.883437	-0.725004	-2.488624
O	35	-3.5307	0.203354	-0.766853
C	36	-2.368796	-1.567574	-1.77041
N	37	-3.194382	-1.264	-0.705904
C	38	-5.912626	0.876553	-5.065081
C	39	-3.660195	-2.313022	0.131126
C	40	-2.803289	-3.910693	-1.142537
C	41	-2.169669	-3.006987	-2.001845
C	42	-2.640452	-5.276477	-1.38398
C	43	-1.87466 -5.729317	-2.459898	

C	44	-1.250355	-4.815966	-3.307644
C	45	-1.400354	-3.44923	-3.081912
N	46	-3.487868	-3.556219	-0.121335
C	47	-6.572239	1.813522	-4.252314
C	48	-7.855318	1.51992	-3.777594
C	49	-8.501781	0.341329	-4.142478
C	50	-7.877964	-0.568647	-4.996116
C	51	-6.581848	-0.293616	-5.439285
C	52	-8.590749	-1.825237	-5.427688
C	53	-5.985195	3.16861	-3.935778
H	54	-7.876559	-2.644384	-5.666575
H	55	-5.282001	1.433121	-1.381474
H	56	-7.264303	-0.443657	0.962232
H	57	-5.536215	0.804124	1.586398
H	58	-6.619132	1.835384	0.62169
H	59	-5.157364	-0.863999	-2.800755
H	60	-6.511542	-2.862541	-3.331121
H	61	-8.275171	-3.645112	-1.746343
H	62	-1.120565	4.456512	-2.009909
H	63	-0.344256	3.873909	-0.495816
H	64	0.252147	5.352382	-1.306308
H	65	2.965776	2.892764	-2.154556
H	66	2.171783	3.209823	-0.574122
H	67	2.63568	4.571167	-1.6262
H	68	1.682588	3.610161	-4.373063
H	69	0.023503	.295023	-4.227972
H	70	1.421523	.20021	-3.597057
H	71	-0.054621	2.088043	-4.995891
H	72	1.142119	1.48058	-3.088166
H	73	-1.0456	1.866973	-7.101464
H	74	-5.239335	1.058307	-7.646292
H	75	-3.117578	-6.006139	-0.709145
H	76	-1.75598	-6.812172	-2.634687
H	77	-0.641791	-5.172775	-4.155775
H	78	-0.916301	-2.723736	-3.75736
H	79	-8.378928	2.242804	-3.130284
H	80	-9.520418	0.140134	-3.771618
H	81	-6.075735	-1.017051	-6.099307

H	82	-9.203529	-1.635989	-6.336997
H	83	-9.271204	-2.204154	-4.632856
H	84	-6.775806	3.950376	-3.894948
H	85	-5.471928	3.165782	-2.949719
H	86	-5.251371	3.501225	-4.702249
C	87	-4.207879	-1.949712	1.461653
C	88	-5.254284	-2.690653	2.023282
C	89	-3.649034	-0.90351	2.205455
C	90	-4.153226	-0.578511	3.464807
C	91	-5.222004	-1.297918	3.995487
C	92	-5.769866	-2.35728	3.275288
H	93	-5.688098	-3.540437	1.469732
H	94	-2.788517	-0.337752	1.809723
H	95	-3.70176	0.245946	4.042523
H	96	-5.622947	-1.039596	4.990028
H	97	-6.607052	-2.937762	3.698472

(B)

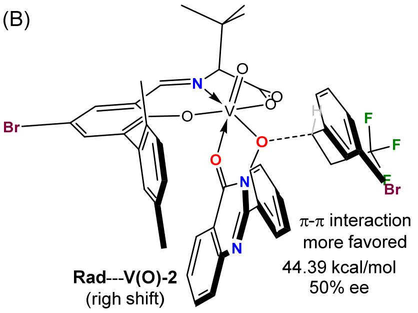

Rad---V(O)-2
(righ shift)

Total Number of atoms: 97

C	1	-3.313861	-0.209945	1.344002
F	2	-2.221362	1.54134	2.607276
F	3	-2.20087	-0.529668	3.446342
F	4	-4.091808	0.656716	3.465168
C	5	-2.96129	0.371227	2.71181
C	6	-6.320236	2.059818	0.30834
C	7	-7.672875	2.27588	0.570482
C	8	-8.385932	1.362694	1.34356
C	9	-7.740218	0.232374	1.840635
C	10	-6.391073	0.009545	1.562316
C	11	-4.147727	0.748618	0.51003
C	12	-5.660743	0.924887	0.794054
O	13	-3.44534	2.795704	-2.021493
Br	14	-1.810859	0.10389	-9.462666

V	15	-2.598568	1.336411	-2.247229
O	16	-1.433588	1.726329	-0.728188
O	17	0.733793	2.117919	-0.303938
O	18	-3.716918	0.991081	-3.799788
N	19	-1.181359	2.19854	-3.293337
Br	20	-8.704347	-1.011537	2.90426
C	21	-0.209468	2.234422	-1.053373
C	22	-1.266481	5.012087	-1.943564
C	23	1.210201	4.786354	-1.672861
C	24	0.211286	4.855993	-3.949756
C	25	-1.040463	2.007961	-4.55245
C	26	-1.976937	1.285727	-5.456647
C	27	-0.006089	4.329226	-2.513039
C	28	-0.124317	2.769355	-2.492965
C	29	-1.568939	1.067149	-6.779208
C	30	-2.39199	0.396413	-7.679429
C	31	-3.635839	-0.069377	-7.270283
C	32	-4.065961	0.149893	-5.9622
C	33	-3.250812	0.849329	-5.063346
O	34	-1.696127	-0.374856	-2.4726
O	35	-3.886542	0.325754	-1.161789
C	36	-2.404171	-1.301598	-2.166957
N	37	-3.639847	-1.086623	-1.604414
C	38	-5.383017	-0.369729	-5.539288
C	39	-4.54083	-2.186838	-1.543261
C	40	-2.988897	-3.680156	-2.088046
C	41	-2.026715	-2.707719	-2.369326
C	42	-2.642757	-5.021441	-2.263892
C	43	-1.364973	-5.380635	-2.697408
C	44	-0.416009	-4.396812	-2.971712
C	45	-0.74606	-3.052983	-2.807704
N	46	-4.182595	-3.404605	-1.720223
C	47	-6.50106	0.473232	-5.458837
C	48	-7.736354	-0.068611	-5.090151
C	49	-7.860168	-1.42471	-4.794225
C	50	-6.747865	-2.265622	-4.852946
C	51	-5.516446	-1.726188	-5.232099
C	52	-6.868215	-3.726445	-4.501832

C	53	-6.393875	1.94428	-5.777732
H	54	-5.950203	-4.101857	-3.997878
H	55	-3.657113	1.74082	0.504427
H	56	-5.909519	-0.893125	1.967502
H	57	-2.363342	-0.441209	0.811718
H	58	-3.835739	-1.185035	1.46554
H	59	-5.767068	2.799754	-0.293709
H	60	-8.17572	3.173618	0.172818
H	61	-9.452906	1.537586	1.558976
H	62	-2.154747	4.835715	-2.588324
H	63	-1.510093	4.651007	-0.919718
H	64	-1.134401	6.115202	-1.876669
H	65	2.144841	4.270469	-1.988256
H	66	1.067257	4.592971	-0.587055
H	67	1.389808	5.879861	-1.777038
H	68	1.099264	4.387694	-4.430496
H	69	-0.67016	4.669633	-4.6021
H	70	0.381257	5.955894	-3.957125
H	71	-0.120467	2.395835	-5.018447
H	72	0.833091	2.376187	-2.908988
H	73	-0.581033	1.420527	-7.116567
H	74	-4.286498	-0.612173	-7.975712
H	75	-3.391981	-5.80548	-2.064354
H	76	-1.107648	-6.445327	-2.830394
H	77	0.592838	-4.680456	-3.316433
H	78	0.002299	-2.270572	-3.018423
H	79	-8.625636	0.579809	-5.026497
H	80	-8.841465	-1.829938	-4.496726
H	81	-4.631272	-2.381157	-5.285506
H	82	-7.028274	-4.33904	-5.416593
H	83	-7.720994	-3.915828	-3.812571
H	84	-7.39289	2.419915	-5.892628
H	85	-5.860165	2.48865	-4.96734
H	86	-5.841481	2.116504	-6.727988
C	87	-5.982374	-1.927972	-1.286533
C	88	-6.763211	-2.890854	-0.63369
C	89	-8.128683	-2.690023	-0.435471
C	90	-8.736815	-1.526162	-0.898758

C	91	-7.975513	-0.564909	-1.558262
C	92	-6.610179	-0.768101	-1.756479
H	93	-6.303447	-3.827031	-0.275045
H	94	-8.729321	-3.456413	0.082797
H	95	-9.817523	-1.368035	-0.746682
H	96	-8.454976	0.351388	-1.940973
H	97	-6.041545	-0.01466	-2.325266

Figure S7:

(A)

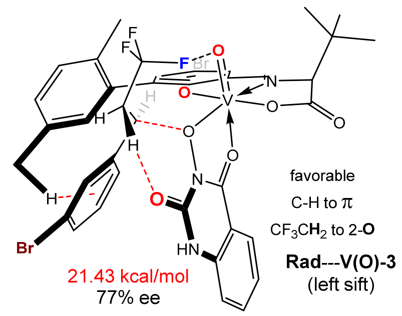

Total number of atoms: 88

C	1	-4.938664	1.775598	1.276846
F	2	-2.537073	1.663376	1.632549
F	3	-3.735092	3.046741	2.90603
F	4	-3.382398	3.552555	0.76379
C	5	-3.64315	2.500683	1.630775
C	6	-6.083029	-0.730702	-1.480419
C	7	-7.037271	-1.726803	-1.682307
C	8	-8.064742	-1.90625	-0.760966
C	9	-8.118385	-1.093459	0.368235
C	10	-7.145899	-0.116251	0.582449
C	11	-4.993875	1.142677	-0.105414
C	12	-6.107262	0.078468	-0.338349
O	13	-2.871883	2.837154	-1.838917
Br	14	-3.254676	1.039601	-9.45773
V	15	-2.330129	1.243428	-2.087327
O	16	-0.894192	1.482989	-0.784984
O	17	1.345327	1.344226	-0.757844
O	18	-3.732301	1.033626	-3.423986
N	19	-0.983803	1.844307	-3.381196
Br	20	-9.517073	-1.327569	1.630328

C	21	0.337563	1.686751	-1.334205
C	22	-0.330855	4.612064	-2.144452
C	23	2.073249	3.922252	-2.197298
C	24	0.812634	4.19279	-4.321691
C	25	-1.116247	1.726344	-4.64804
C	26	-2.351604	1.361245	-5.389548
C	27	0.691514	3.704183	-2.859711
C	28	0.285512	2.192021	-2.784996
C	29	-2.272591	1.344369	-6.788421
C	30	-3.388947	1.054576	-7.564255
C	31	-4.610427	0.808407	-6.951687
C	32	-4.718113	0.826814	-5.559969
C	33	-3.579195	1.070667	-4.772826
O	34	-1.745879	-0.593211	-2.34822
O	35	-3.498003	0.501719	-0.69033
C	36	-2.134367	-1.344594	-1.487233
N	37	-3.005854	-0.911335	-0.504186
C	38	-6.05274	0.603019	-4.958403
C	39	-3.424622	-1.666218	0.587063
C	40	-2.123868	-3.531813	-0.351127
C	41	-1.684292	-2.745954	-1.415517
C	42	-1.701712	-4.864546	-0.267591
C	43	-0.850269	-5.396929	-1.236675
C	44	-0.412548	-4.601196	-2.295096
C	45	-0.828787	-3.273257	-2.387369
N	46	-2.985066	-2.973244	0.633528
C	47	-6.693293	1.593832	-4.195351
C	48	-7.973149	1.342716	-3.688626
C	49	-8.633028	0.148598	-3.968523
C	50	-8.021727	-0.82514	-4.758443
C	51	-6.730385	-0.58893	-5.236035
C	52	-8.739063	-2.110167	-5.085155
C	53	-6.090844	2.958885	-3.962851
H	54	-8.028091	-2.955998	-5.216112
H	55	-5.137126	1.963596	-0.8356
H	56	-7.211625	0.502853	1.490072
H	57	-5.098977	0.995488	2.053725
H	58	-5.783261	2.495478	1.382654

H	59	-5.289525	-0.603952	-2.233149
H	60	-6.979066	-2.368984	-2.57676
H	61	-8.825557	-2.685964	-0.928922
H	62	-1.317356	4.601145	-2.656993
H	63	-0.492108	4.307329	-1.086631
H	64	0.009146	5.671853	-2.129145
H	65	2.844753	3.242454	-2.624009
H	66	2.044237	3.754113	-1.098229
H	67	2.437497	4.963677	-2.34632
H	68	1.508154	3.556634	-4.913647
H	69	-0.169701	4.207478	-4.842798
H	70	1.205816	5.233026	-4.370037
H	71	-0.233119	1.95584	-5.265027
H	72	1.065813	1.62236	-3.342476
H	73	-1.317126	1.56857	-7.290009
H	74	-5.503664	0.614067	-7.567923
H	75	-2.039733	-5.497123	0.569203
H	76	-0.518491	-6.446775	-1.16351
H	77	0.265454	-5.020762	-3.057448
H	78	-0.477658	-2.644887	-3.223416
H	79	-8.481969	2.108931	-3.080643
H	80	-9.648156	-0.018755	-3.572292
H	81	-6.23908	-1.356826	-5.855746
H	82	-9.320228	-2.006775	-6.028246
H	83	-9.4492	-2.400542	-4.279273
H	84	-6.873426	3.749764	-3.954809
H	85	-5.566695	3.002082	-2.983393
H	86	-5.363005	3.242804	-4.754267
O	87	-4.139101	-1.240613	1.461614
H	88	-3.327515	-3.508945	1.446132

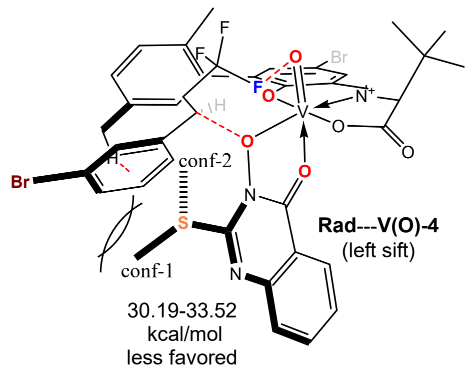

Total number of atoms: 91

C	1	-4.980415	1.864853	1.126797
F	2	-2.597434	1.90932	1.589786
F	3	-3.929689	3.270214	2.749378
F	4	-3.506635	3.711819	0.607251
C	5	-3.747092	2.680914	1.503608
C	6	-5.903269	-0.870829	-1.484338
C	7	-6.838177	-1.888173	-1.669945
C	8	-7.927791	-1.999612	-0.811226
C	9	-8.063722	-1.095603	0.239009
C	10	-7.110735	-0.096573	0.440139
C	11	-4.919003	1.119971	-0.1989
C	12	-6.008306	0.028344	-0.416391
O	13	-2.787891	2.839788	-1.788131
Br	14	-3.340149	1.073135	-9.434409
V	15	-2.260254	1.251566	-2.091004
O	16	-0.792811	1.450291	-0.817302
O	17	1.447016	1.315382	-0.852477
O	18	-3.697536	1.081904	-3.394392
N	19	-0.94841	1.889039	-3.401828
Br	20	-9.545691	-1.235439	1.416381
C	21	0.423748	1.67259	-1.392137
C	22	-0.290457	4.622922	-2.111956
C	23	2.116081	3.94876	-2.205611
C	24	0.83127	4.264976	-4.310669
C	25	-1.110309	1.793077	-4.667645
C	26	-2.357525	1.418505	-5.385876
C	27	0.729645	3.739583	-2.860303
C	28	0.333298	2.223819	-2.824679
C	29	-2.307264	1.399159	-6.786266

C	30	-3.436381	1.094724	-7.538375
C	31	-4.642416	0.833482	-6.901016
C	32	-4.721478	0.854903	-5.507558
C	33	-3.571126	1.120169	-4.745534
O	34	-1.685066	-0.573122	-2.44298
O	35	-3.387442	0.469822	-0.676882
C	36	-2.034622	-1.344639	-1.586147
N	37	-2.860358	-0.93801	-0.562923
C	38	-6.036394	0.609	-4.872952
C	39	-3.064729	-1.85613	0.505491
C	40	-1.96616	-3.521193	-0.453849
C	41	-1.588546	-2.745825	-1.551727
C	42	-1.548593	-4.853059	-0.407176
C	43	-0.773383	-5.396481	-1.433005
C	44	-0.401747	-4.608524	-2.521165
C	45	-0.808929	-3.277004	-2.582422
N	46	-2.663142	-3.069974	0.516902
C	47	-6.678397	1.594898	-4.105151
C	48	-7.934852	1.319302	-3.554589
C	49	-8.572959	0.105364	-3.798252
C	50	-7.963073	-0.862735	-4.596575
C	51	-6.692328	-0.602362	-5.115683
C	52	-8.660379	-2.166566	-4.891292
C	53	-6.103215	2.97875	-3.918401
H	54	-7.935416	-3.001395	-5.016128
H	55	-5.064589	1.887152	-0.983441
H	56	-7.244906	0.595418	1.285539
H	57	-5.15938	1.142348	1.952651
H	58	-5.860089	2.549637	1.122075
H	59	-5.06467	-0.794135	-2.194438
H	60	-6.719178	-2.596505	-2.507012
H	61	-8.674327	-2.795468	-0.968247
H	62	-1.279976	4.625249	-2.618795
H	63	-0.443886	4.286454	-1.06267
H	64	0.047258	5.682426	-2.067388
H	65	2.887434	3.285512	-2.657297
H	66	2.098343	3.751233	-1.111263
H	67	2.472175	4.995969	-2.330009

H	68	1.52645	3.650047	-4.925115
H	69	-0.15651	4.281932	-4.821685
H	70	1.213303	5.310113	-4.336775
H	71	-0.243597	2.041456	-5.300262
H	72	1.103326	1.677101	-3.418225
H	73	-1.364701	1.631251	-7.307992
H	74	-5.544926	0.622538	-7.498163
H	75	-1.830721	-5.480908	0.454341
H	76	-0.448328	-6.449114	-1.379236
H	77	0.215732	-5.035623	-3.329485
H	78	-0.513819	-2.64881	-3.439853
H	79	-8.445143	2.081631	-2.94284
H	80	-9.5714	-0.081169	-3.369768
H	81	-6.199583	-1.366479	-5.73908
H	82	-9.254479	-2.090566	-5.828895
H	83	-9.355736	-2.455762	-4.072128
H	84	-6.906171	3.74772	-3.872915
H	85	-5.524482	3.048977	-2.972089
H	86	-5.430896	3.273091	-4.753722
S	87	-4.029687	-1.412122	1.962729
C	88	-5.255964	-2.684425	2.320353
H	89	-4.741702	-3.637055	2.573878
H	90	-5.902836	-2.837167	1.429181
H	91	-5.879447	-2.357523	3.180786

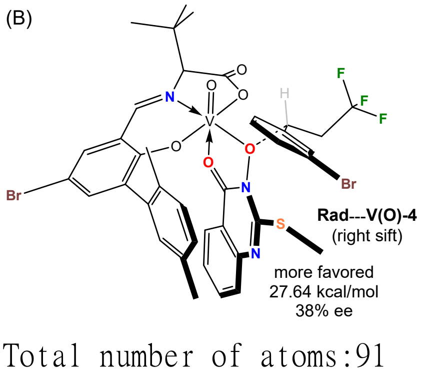

Total number of atoms:91

C	1	-2.79042	0.851531	0.630975
F	2	-1.89918	3.00542	1.27747
F	3	-1.10022	1.18573	2.30022
F	4	-3.15756	1.91245	2.77468
C	5	-2.24392	1.74375	1.74317

C	6	-6.56455	1.8772	0.122636
C	7	-7.83923	1.70322	0.660472
C	8	-8.02428	0.854613	1.74863
C	9	-6.92599	0.197198	2.29802
C	10	-5.65121	0.379379	1.7608
C	11	-4.05357	1.42077	0.009521
C	12	-5.45444	1.20512	0.646865
O	13	-4.79408	3.24071	-2.43329
Br	14	-7.42862	0.257781	-9.41693
V	15	-3.94103	1.96633	-3.17039
O	16	-2.23628	2.74185	-2.62003
O	17	-0.409935	3.65244	-3.55369
O	18	-5.64116	1.23267	-3.74389
N	19	-3.68175	3.01814	-4.80388
Br	20	-7.16296	-0.954613	3.78836
C	21	-1.60694	3.47547	-3.5859
C	22	-3.51004	5.887	-3.49912
C	23	-1.44873	6.18717	-4.88587
C	24	-3.64784	5.83085	-5.9899
C	25	-4.30158	2.75569	-5.89604
C	26	-5.39857	1.76377	-6.08849
C	27	-2.78531	5.41476	-4.77662
C	28	-2.51847	3.8722	-4.76364
C	29	-5.84736	1.51821	-7.39268
C	30	-6.85154	0.583978	-7.63813
C	31	-7.41091	-0.134738	-6.58675
C	32	-6.99069	0.107885	-5.27985
C	33	-6.02159	1.08629	-5.03133
O	34	-2.97834	0.499408	-4.00894
O	35	-4.09853	0.775351	-1.61173
C	36	-3.10895	-0.537429	-3.41082
N	37	-3.74467	-0.571367	-2.19041
C	38	-7.52633	-0.702964	-4.164
C	39	-4.1066	-1.85634	-1.69776
C	40	-3.00162	-2.98217	-3.25043
C	41	-2.65328	-1.82701	-3.95109
C	42	-2.5762	-4.21464	-3.74969
C	43	-1.8183	-4.28903	-4.91974

C	44	-1.48117	-3.12576	-5.61035
C	45	-1.90094	-1.88808	-5.12685
N	46	-3.70645	-2.96867	-2.18479
C	47	-8.32535	-0.136714	-3.15827
C	48	-8.72964	-0.931517	-2.08035
C	49	-8.36418	-2.27366	-2.00565
C	50	-7.58612	-2.84962	-3.01076
C	51	-7.1745	-2.05317	-4.08184
C	52	-7.17206	-4.29727	-2.94227
C	53	-8.78418	1.29958	-3.22195
H	54	-7.69823	-4.89538	-3.71918
H	55	-3.89395	2.4878	-0.236401
H	56	-4.80352	-0.149044	2.22235
H	57	-2.0042	0.749412	-0.151914
H	58	-2.96686	-0.17545	1.02018
H	59	-6.44357	2.54981	-0.740232
H	60	-8.70059	2.23491	0.221294
H	61	-9.03264	0.711744	2.17087
H	62	-4.53862	5.47085	-3.43435
H	63	-2.96416	5.59397	-2.57494
H	64	-3.61271	6.99507	-3.47864
H	65	-0.842305	5.83905	-5.75212
H	66	-0.824319	6.08006	-3.97208
H	67	-1.6188	7.27834	-5.02495
H	68	-3.1868	5.51295	-6.95187
H	69	-4.67367	5.40315	-5.94102
H	70	-3.77058	6.93591	-6.03978
H	71	-3.97118	3.29127	-6.80036
H	72	-1.93783	3.64801	-5.68928
H	73	-5.39481	2.05662	-8.24116
H	74	-8.1815	-0.898827	-6.78095
H	75	-2.84472	-5.13961	-3.21328
H	76	-1.48724	-5.27023	-5.30039
H	77	-0.882979	-3.18417	-6.53565
H	78	-1.63582	-0.964981	-5.66943
H	79	-9.34645	-0.496317	-1.27678
H	80	-8.69132	-2.87872	-1.14383
H	81	-6.53381	-2.49326	-4.86364

H	82	-7.40336	-4.7523	-1.9536
H	83	-6.07789	-4.41041	-3.1096
H	84	-9.73109	1.45296	-2.65852
H	85	-8.02394	1.98279	-2.78403
H	86	-8.97624	1.62637	-4.26781
S	87	-5.12053	-2.04966	-0.222228
C	88	-4.41374	-3.27165	0.897914
H	89	-4.37651	-4.26149	0.39256
H	90	-3.38632	-2.9585	1.18573
H	91	-5.0489	-3.34376	1.80755

Figure S9:

(A)

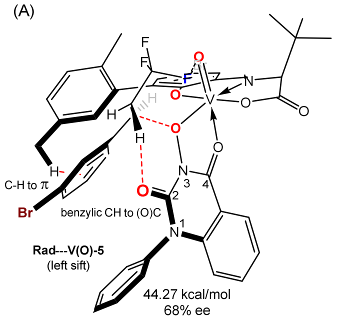

Total number of atoms: 98

C	1	-5.57351	1.49967	0.695577
F	2	-3.45404	2.64473	0.984342
F	3	-5.23297	3.42052	2.08248
F	4	-5.08455	3.71984	-0.123454
C	5	-4.82812	2.81505	0.898723
C	6	-5.99301	-1.1093	-2.17136
C	7	-6.67555	-2.29014	-2.46014
C	8	-7.47751	-2.88594	-1.49079
C	9	-7.57847	-2.29915	-0.23164
C	10	-6.88399	-1.12449	0.057908
C	11	-5.2731	0.788018	-0.610972
C	12	-6.07858	-0.510018	-0.910233
O	13	-2.82437	2.81133	-1.75597
Br	14	-3.10915	1.91601	-9.52729
V	15	-2.36143	1.22612	-2.16518
O	16	-0.958455	1.24385	-0.806292
O	17	1.26198	0.922033	-0.756366
O	18	-3.73127	1.23344	-3.54923

N	19	-0.947539	1.8842	-3.35494
Br	20	-8.65779	-3.11414	1.10092
C	21	0.299338	1.40539	-1.30873
C	22	-0.119905	4.43007	-1.7578
C	23	2.22968	3.5802	-1.91002
C	24	0.998278	4.20432	-3.97595
C	25	-1.05457	1.92712	-4.62939
C	26	-2.28786	1.72032	-5.43363
C	27	0.837702	3.54758	-2.58582
C	28	0.324425	2.07104	-2.69503
C	29	-2.17767	1.8645	-6.82301
C	30	-3.28744	1.71588	-7.64787
C	31	-4.53109	1.44256	-7.09311
C	32	-4.66828	1.30245	-5.7116
C	33	-3.54181	1.41896	-4.88004
O	34	-1.87175	-0.60241	-2.61301
O	35	-3.60265	0.401783	-0.88542
C	36	-2.27663	-1.40702	-1.80971
N	37	-3.11422	-1.01928	-0.78087
C	38	-6.01435	1.03462	-5.15703
C	39	-3.5131	-1.82022	0.280384
C	40	-2.34955	-3.686	-0.866802
C	41	-1.87261	-2.82323	-1.85161
C	42	-1.94008	-5.02482	-0.880208
C	43	-1.09576	-5.49314	-1.88824
C	44	-0.641927	-4.62662	-2.8816
C	45	-1.02597	-3.28685	-2.86253
N	46	-3.19801	-3.16379	0.148314
C	47	-6.65412	1.94943	-4.30463
C	48	-7.91995	1.63684	-3.79737
C	49	-8.56614	0.458023	-4.16246
C	50	-7.96083	-0.43204	-5.04999
C	51	-6.6841	-0.134452	-5.53292
C	52	-8.67299	-1.69259	-5.47073
C	53	-6.05768	3.29659	-3.97253
H	54	-7.96065	-2.47858	-5.80675
H	55	-5.43156	1.50715	-1.43767
H	56	-6.9699	-0.694671	1.06771

H	57	-5.33349	0.833703	1.55403
H	58	-6.66675	1.70905	0.75202
H	59	-5.36076	-0.657056	-2.95117
H	60	-6.57739	-2.75179	-3.45686
H	61	-8.0205	-3.81743	-1.72041
H	62	-1.10418	4.55006	-2.26057
H	63	-0.304387	4.01129	-0.74373
H	64	0.29376	5.45365	-1.6162
H	65	2.95176	2.90778	-2.42554
H	66	2.1867	3.27434	-0.841709
H	67	2.66735	4.60355	-1.92706
H	68	1.65252	3.60114	-4.64458
H	69	0.021324	4.34988	-4.48716
H	70	1.46092	5.2135	-3.89581
H	71	-0.143312	2.17013	-5.19837
H	72	1.07866	1.51218	-3.29753
H	73	-1.20215	2.1029	-7.27743
H	74	-5.41617	1.34884	-7.74378
H	75	-2.27121	-5.72514	-0.09668
H	76	-0.779515	-6.55022	-1.89485
H	77	0.029714	-4.99728	-3.67478
H	78	-0.654912	-2.59886	-3.64088
H	79	-8.42834	2.34188	-3.11894
H	80	-9.56965	0.239114	-3.76152
H	81	-6.19137	-0.840362	-6.22132
H	82	-9.3706	-1.48748	-6.31291
H	83	-9.26729	-2.12399	-4.63461
H	84	-6.84843	4.06779	-3.83886
H	85	-5.47239	3.2563	-3.02853
H	86	-5.38466	3.66827	-4.77616
O	87	-4.12814	-1.3946	1.23
C	88	-3.83727	-4.07464	1.02824
C	89	-3.34348	-4.2814	2.31653
C	90	-3.97773	-5.19026	3.16413
C	91	-5.09408	-5.89838	2.71687
C	92	-5.57591	-5.70377	1.42209
C	93	-4.94339	-4.79469	0.574705
H	94	-2.46152	-3.7183	2.6628

H	95	-3.59968	-5.34547	4.18878
H	96	-5.59877	-6.61244	3.38941
H	97	-6.45878	-6.26418	1.07098
H	98	-5.31854	-4.63756	-0.44939

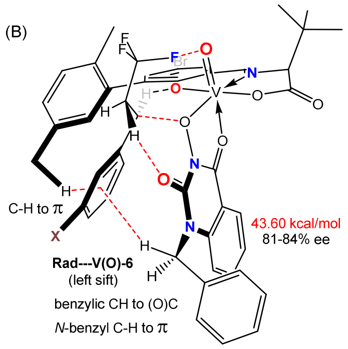

Total number of atoms: 101

C	1	-6.4698	2.6448	2.082
F	2	-4.4474	3.9721	2.249
F	3	-6.3481	4.839899	3.0309
F	4	-6.0391	4.6668	0.8278
C	5	-5.8177	4.0238	2.0388
C	6	-6.5596	-0.4831	-0.2498
C	7	-7.1001	-1.7659	-0.3218
C	8	-7.8381	-2.2692	0.7456
C	9	-8.0298	-1.4798	1.877199
C	10	-7.4807	-0.1993	1.9489
C	11	-6.0523	1.7179	0.956799
C	12	-6.7283	0.3166	0.886
O	13	-3.421	3.5764	-0.3612
Br	14	-4.0976	0.9566	-7.8316
V	15	-3.094	1.917099	-0.547
O	16	-1.6273	2.039099	0.7371
O	17	0.5861	1.6737	0.7703
O	18	-4.5195	1.840299	-1.869
N	19	-1.6977	2.2569	-1.8824
Br	20	-9.023	-2.1656	3.341799
C	21	-0.3851	2.081199	0.173699
C	22	-0.7288	5.010599	-0.8687
C	23	1.580099	4.0478	-0.8249
C	24	0.376	4.2874	-2.9857

C	25	-1.8513	2.0416	-3.1352
C	26	-3.1232	1.731299	-3.8421
C	27	0.188	3.9374	-1.4914
C	28	-0.3929	2.4936	-1.3087
C	29	-3.0608	1.5159	-5.225
C	30	-4.2125	1.251799	-5.96
C	31	-5.45	1.2262	-5.3282
C	32	-5.5388	1.450499	-3.9543
C	33	-4.3729	1.6824	-3.2072
O	34	-2.7754	0.004199	-0.7188
O	35	-4.3372	1.4001	0.882299
C	36	-3.1953	-0.6225	0.2238
N	37	-3.917	-0.0057	1.22
C	38	-6.8709	1.4466	-3.3082
C	39	-4.279	-0.5738	2.432
C	40	-3.4574	-2.6999	1.5285
C	41	-2.9624	-2.0643	0.3882
C	42	-3.2598	-4.0826	1.6444
C	43	-2.5784	-4.7965	0.6569
C	44	-2.0857	-4.1417	-0.4689
C	45	-2.2815	-2.7703	-0.6074
O	46	-4.7402	0.089599	3.330899
N	47	-4.1475	-1.9566	2.5416
C	48	-7.3868	2.598499	-2.6923
C	49	-8.6242	2.5291	-2.0439
C	50	-9.3591	1.3462	-2.0324
C	51	-8.8789	0.211	-2.6859
C	52	-7.6371	0.2766	-3.3236
C	53	-9.6883	-1.0608	-2.6791
C	54	-6.6775	3.930299	-2.7561
C	55	-4.7417	-2.6455	3.692799
C	56	-3.9264	-4.4997	5.208299
C	57	-2.9965	-5.0412	6.095199
C	58	-1.8584	-4.3158	6.4412
C	59	-1.6584	-3.0445	5.9068
C	60	-2.5904	-2.5027	5.022
C	61	-3.7244	-3.232	4.6472
H	62	-9.1041	-1.9307	-3.0531

H	63	-5.4386	-3.4394	3.340699
H	64	-5.3846	-1.9624	4.2916
H	65	-6.1943	2.2481	-0.0039
H	66	-7.6289	0.3922	2.865499
H	67	-6.2375	2.1815	3.066399
H	68	-7.5755	2.779999	2.046799
H	69	-5.9624	-0.1119	-1.0969
H	70	-6.9338	-2.3832	-1.2209
H	71	-8.2609	-3.2859	0.6918
H	72	-1.7045	5.073199	-1.3984
H	73	-0.9349	4.81	0.205999
H	74	-0.2679	6.022	-0.9282
H	75	2.2716	3.2535	-1.1854
H	76	1.5235	3.9708	0.2831
H	77	2.0625	5.026	-1.0474
H	78	0.9996	3.5303	-3.5119
H	79	-0.5939	4.3685	-3.5237
H	80	0.8849	5.2695	-3.1109
H	81	-0.9526	2.1144	-3.7683
H	82	0.3114	1.7999	-1.8255
H	83	-2.0902	1.555299	-5.746
H	84	-6.3657	1.040899	-5.9134
H	85	-3.6311	-4.6393	2.516499
H	86	-2.4262	-5.8836	0.769799
H	87	-1.5467	-4.7063	-1.2486
H	88	-1.9027	-2.2508	-1.5041
H	89	-9.0342	3.4244	-1.5476
H	90	-10.3338	1.3135	-1.5178
H	91	-7.2395	-0.6211	-3.8243
H	92	-10.5892	-0.9595	-3.3241
H	93	-10.0321	-1.3119	-1.6508
H	94	-7.4003	4.776	-2.7614
H	95	-6.0085	4.071799	-1.8796
H	96	-6.0614	4.033099	-3.6765
H	97	-4.8283	-5.0816	4.954299
H	98	-3.1642	-6.0419	6.5275
H	99	-1.1229	-4.7424	7.1434
H	100	-0.7638	-2.4633	6.1872

H	101	-2.4251	-1.491	4.6149

(C)

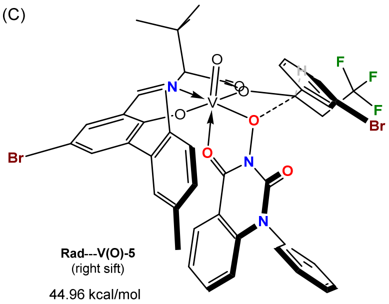

Rad---V(O)-5
(right sift)
44.96 kcal/mol

Total number of atoms: 98

C	1	-3.58444	0.176171	1.53336
F	2	-2.56291	1.99934	2.74976
F	3	-2.7653	0.029002	3.78422
F	4	-4.57482	1.29161	3.43709
C	5	-3.37577	0.879962	2.87155
C	6	-6.34353	2.30485	-0.295636
C	7	-7.72109	2.51707	-0.34736
C	8	-8.57817	1.70747	0.394325
C	9	-8.04759	0.6914	1.18629
C	10	-6.66936	0.483642	1.23796
C	11	-4.25811	1.08036	0.51527
C	12	-5.79727	1.27908	0.484098
O	13	-3.13228	2.89677	-1.86423
Br	14	-2.13558	0.578533	-9.4945
V	15	-2.46202	1.36319	-2.17294
O	16	-1.20527	1.56614	-0.692723
O	17	1.00701	1.73192	-0.362734
O	18	-3.66886	1.1631	-3.68105
N	19	-1.02437	2.15479	-3.24546
Br	20	-9.20682	-0.414583	2.20605
C	21	0.044935	1.97768	-1.05488
C	22	-0.76328	4.88634	-1.69811
C	23	1.69129	4.41192	-1.63274
C	24	0.545624	4.73261	-3.81651
C	25	-0.975576	2.06238	-4.52202
C	26	-2.01375	1.48029	-5.41624
C	27	0.379764	4.12763	-2.40384

C	28	0.113845	2.58578	-2.46767
C	29	-1.69484	1.34145	-6.77318
C	30	-2.59968	0.772629	-7.66402
C	31	-3.82718	0.309448	-7.20587
C	32	-4.17214	0.449071	-5.86072
C	33	-3.2882	1.08757	-4.97844
O	34	-1.7913	-0.425195	-2.53944
O	35	-3.78789	0.458089	-1.0467
C	36	-2.57237	-1.28695	-2.21408
N	37	-3.69883	-0.980686	-1.47326
C	38	-5.43685	-0.149028	-5.37414
C	39	-4.70494	-1.86917	-1.12407
C	40	-3.37525	-3.62241	-2.26308
C	41	-2.40588	-2.69031	-2.63076
C	42	-3.24615	-4.94476	-2.7045
C	43	-2.14648	-5.32697	-3.47434
C	44	-1.17347	-4.3907	-3.82057
C	45	-1.30512	-3.06697	-3.4051
N	46	-4.48063	-3.1905	-1.4778
C	47	-6.44235	0.609751	-4.75245
C	48	-7.58375	-0.040347	-4.26935
C	49	-7.75202	-1.41378	-4.4333
C	50	-6.77577	-2.16965	-5.08227
C	51	-5.62256	-1.52606	-5.53469
C	52	-6.94044	-3.65235	-5.3001
C	53	-6.37098	2.11258	-4.62728
H	54	-6.09177	-4.2184	-4.85509
H	55	-3.73135	2.05309	0.470811
H	56	-6.28016	-0.325477	1.8745
H	57	-2.58971	-0.145674	1.14846
H	58	-4.16714	-0.758859	1.68553
H	59	-5.6805	2.95478	-0.889474
H	60	-8.13181	3.32563	-0.975407
H	61	-9.66721	1.87447	0.35426
H	62	-1.70553	4.84688	-2.28631
H	63	-0.974711	4.47709	-0.685374
H	64	-0.51443	5.96337	-1.5677
H	65	2.54722	3.83596	-2.05076

H	66	1.60821	4.15756	-0.553447
H	67	1.96814	5.48892	-1.68181
H	68	1.35124	4.2234	-4.39147
H	69	-0.392431	4.67231	-4.41118
H	70	0.816387	5.81113	-3.76653
H	71	-0.053678	2.41137	-5.01408
H	72	1.01112	2.13369	-2.95214
H	73	-0.710693	1.67019	-7.14523
H	74	-4.52805	-0.177587	-7.90369
H	75	-4.00858	-5.70025	-2.45695
H	76	-2.04904	-6.37213	-3.81455
H	77	-0.306789	-4.6938	-4.43249
H	78	-0.544503	-2.32288	-3.69591
H	79	-8.37249	0.542128	-3.76489
H	80	-8.66755	-1.89751	-4.0548
H	81	-4.83489	-2.12551	-6.01986
H	82	-6.97449	-3.89104	-6.38644
H	83	-7.87756	-4.04309	-4.84513
H	84	-7.37163	2.5764	-4.77424
H	85	-6.01102	2.41337	-3.6186
H	86	-5.69545	2.56894	-5.3838
C	87	-5.43097	-4.14682	-1.03071
C	88	-5.09701	-5.03489	-0.006672
C	89	-6.03024	-5.97633	0.428457
C	90	-7.29396	-6.02908	-0.160911
C	91	-7.62774	-5.14145	-1.18405
C	92	-6.69486	-4.20068	-1.61891
H	93	-4.10124	-4.98383	0.462894
H	94	-5.77238	-6.67371	1.24331
H	95	-8.03418	-6.76822	0.189253
H	96	-8.63101	-5.1758	-1.64136
H	97	-6.9567	-3.48578	-2.41436
O	98	-5.69872	-1.52952	-0.528493

Figure S10:

(A)

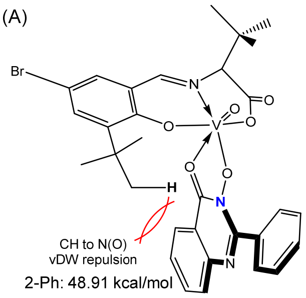

Total number of atoms: 73

O	1	-9.89268	1.725128	2.128324
Br	2	-16.879627	3.293322	-1.095626
V	3	-10.156735	0.822623	0.709251
O	4	-8.293484	1.063385	0.172891
O	5	-7.191651	1.891952	-1.599335
O	6	-12.009493	0.576433	1.259757
N	7	-10.458853	2.437923	-0.356487
C	8	-8.112162	1.983514	-0.818661
C	9	-8.486177	4.362512	1.121812
C	10	-7.572107	4.903533	-1.144519
C	11	-9.930123	5.462384	-0.589035
C	12	-11.625214	2.82033	-0.707899
C	13	-12.923728	2.269864	-0.240799
C	14	-8.82272	4.403419	-0.383159
C	15	-9.259014	3.001398	-0.926116
C	16	-14.073716	2.87294	-0.763144
C	17	-15.336884	2.449401	-0.376633
C	18	-15.470259	1.410794	0.535444
C	19	-14.344272	0.776915	1.080855
C	20	-13.060243	1.227775	0.695761
O	21	-10.430013	-0.346991	-0.826467
O	22	-9.758534	-0.869843	1.567602
C	23	-10.230425	-1.523035	-0.612063
N	24	-9.834063	-2.005421	0.617371
C	25	-14.531519	-0.371472	2.077871
C	26	-9.632179	-3.390453	0.829738
C	27	-10.193629	-3.899987	-1.258335
C	28	-10.409973	-2.567411	-1.633343
C	29	-10.375549	-4.893812	-2.222402

C	30	-10.759592	-4.570514	-3.524837
C	31	-10.969668	-3.239756	-3.882142
C	32	-10.797273	-2.233037	-2.933964
N	33	-9.82739	-4.264055	-0.085854
C	34	-13.848313	-0.059005	3.431775
C	35	-16.017957	-0.657046	2.415141
C	36	-13.962166	-1.687599	1.49582
C	37	-9.151251	-3.852541	2.160952
C	38	-8.308487	-3.064234	2.955937
C	39	-7.863085	-3.521388	4.196414
C	40	-9.511415	-5.123152	2.630183
C	41	-9.06659	-5.582147	3.869908
C	42	-8.244392	-4.779374	4.656669
H	43	-10.965766	-1.179305	-3.213092
H	44	-9.382807	4.128536	1.735862
H	45	-7.70547	3.605292	1.35578
H	46	-8.103081	5.345177	1.477506
H	47	-7.731345	4.893787	-2.24622
H	48	-6.673049	4.285962	-0.927913
H	49	-7.313287	5.948467	-0.861438
H	50	-10.24777	5.525984	-1.653932
H	51	-10.831556	5.252275	0.02779
H	52	-9.580384	6.477272	-0.294905
H	53	-11.680277	3.652929	-1.42704
H	54	-9.459139	3.130618	-2.015886
H	55	-13.985744	3.699914	-1.486473
H	56	-16.487916	1.102617	0.815537
H	57	-10.206806	-5.948989	-1.950445
H	58	-10.893593	-5.369712	-4.273601
H	59	-11.271595	-2.984093	-4.911994
H	60	-14.208676	0.90634	3.853367
H	61	-12.741638	0.004298	3.361501
H	62	-14.065693	-0.847605	4.186459
H	63	-16.519862	0.223843	2.876066
H	64	-16.112894	-1.48985	3.147582
H	65	-16.603318	-0.96375	1.519057
H	66	-14.440983	-1.940192	0.522936
H	67	-12.865869	-1.646496	1.320088

H	68	-14.140194	-2.545588	2.182046
H	69	-7.959312	-2.080375	2.604045
H	70	-7.196989	-2.890435	4.808888
H	71	-10.159189	-5.774942	2.020153
H	72	-9.362721	-6.58324	4.226124
H	73	-7.887805	-5.142522	5.635205

(B)

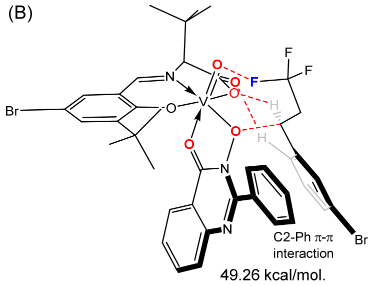

Total number of atoms: 93

C	1	8.579242	-3.117952	-1.302424
H	2	8.772933	-4.011327	-1.938549
C	3	7.429179	-0.230696	-1.109211
C	4	6.919579	1.053798	-0.914724
C	5	5.554528	1.249269	-0.716135
C	6	4.699679	0.150116	-0.700259
C	7	5.212852	-1.133106	-0.883871
C	8	7.097441	-2.790815	-1.316395
C	9	6.578667	-1.343003	-1.107974
O	10	6.718976	-5.913477	-2.186401
H	11	8.511805	-0.351577	-1.26515
Br	12	8.092006	2.547416	-0.924801
Br	13	3.758921	-8.800725	-9.048691
V	14	5.427642	-5.056176	-2.8902
O	15	4.616772	-4.741539	-1.140629
O	16	2.744925	-5.25258	-0.016641
O	17	6.186694	-5.441387	-4.642364
N	18	4.269542	-6.636985	-2.907274
H	19	6.125532	-8.080962	-1.188551
H	20	5.149137	2.263638	-0.56642
H	21	3.617402	0.292949	-0.539246
H	22	4.520307	-1.991635	-0.865282
C	23	9.084458	-3.406208	0.108712

H	24	2.767691	-9.5225	-2.090318
H	25	2.471385	-6.987348	-1.965014
H	26	3.18259	-8.031543	-3.903607
H	27	3.171928	-8.522089	-6.171129
H	28	9.176828	-2.29337	-1.745164
H	29	5.598109	-7.112324	0.231574
H	30	3.177228	-7.451606	1.127838
H	31	1.944038	-8.252101	0.09529
H	32	3.203103	-9.221458	0.91968
H	33	4.510933	-9.442202	-2.533714
H	34	4.001593	-10.313941	-1.06578
H	35	5.971337	-6.876396	-8.961268
H	36	5.642906	-8.898298	0.321643
C	37	3.593356	-5.582013	-0.814595
C	38	5.400634	-8.042007	-0.346863
C	39	3.018943	-8.263127	0.384212
C	40	3.796773	-9.409537	-1.681494
C	41	3.967289	-7.262246	-3.981973
C	42	4.562649	-7.077232	-5.333849
C	43	3.943263	-8.115989	-0.848019
C	44	3.541062	-6.851422	-1.679252
C	45	4.01599	-7.842062	-6.370028
C	46	4.524261	-7.756581	-7.658565
C	47	5.595833	-6.914448	-7.928306
C	48	6.174575	-6.131977	-6.918287
C	49	5.64669	-6.225354	-5.609869
O	50	4.002502	-4.012256	-3.706499
O	51	6.422456	-3.359999	-2.830672
C	52	4.372002	-2.916759	-4.049342
N	53	5.646403	-2.480281	-3.769189
C	54	7.356611	-5.220309	-7.255403
C	55	6.089508	-1.274099	-4.38738
C	56	4.083592	-0.770547	-5.200088
C	57	3.514318	-1.975354	-4.783435
C	58	3.277447	0.138252	-5.888634
C	59	1.935571	-0.147682	-6.148052
C	60	1.383103	-1.356014	-5.726055
C	61	2.173868	-2.275443	-5.039633

C	62	7.53241	-0.91436	-4.325461
H	63	3.710037	1.090477	-6.237564
H	64	1.312726	0.58112	-6.694146
H	65	0.32382	-1.584039	-5.934009
H	66	1.740706	-3.231309	-4.700133
H	67	6.572043	-3.485395	-0.635844
F	68	8.455382	-4.5061	0.676878
F	69	8.890961	-2.323936	0.956597
F	70	10.447019	-3.676513	0.095487
N	71	5.312771	-0.470732	-5.014229
C	72	6.922565	-3.742971	-7.134717
C	73	7.889077	-5.401472	-8.700189
H	74	8.224213	-6.445551	-8.894809
C	75	7.916121	0.432542	-4.285057
C	76	9.262604	0.79272	-4.239315
C	77	10.248599	-0.190426	-4.256956
C	78	8.535922	-1.889814	-4.368898
C	79	9.883788	-1.532275	-4.332489
H	80	11.313858	0.094037	-4.22516
H	81	8.274191	-2.956369	-4.451632
H	82	10.662462	-2.312925	-4.368908
H	83	7.149813	1.225943	-4.277289
H	84	9.546607	1.857678	-4.192432
C	85	8.566393	-5.499603	-6.329979
H	86	6.095614	-3.502404	-7.840054
H	87	6.560637	-3.492512	-6.116538
H	88	7.762735	-3.048959	-7.361159
H	89	7.130859	-5.130545	-9.468814
H	90	8.768769	-4.746415	-8.89038
H	91	8.867197	-6.570725	-6.371056
H	92	8.373573	-5.253883	-5.264688
H	93	9.454219	-4.899198	-6.629711

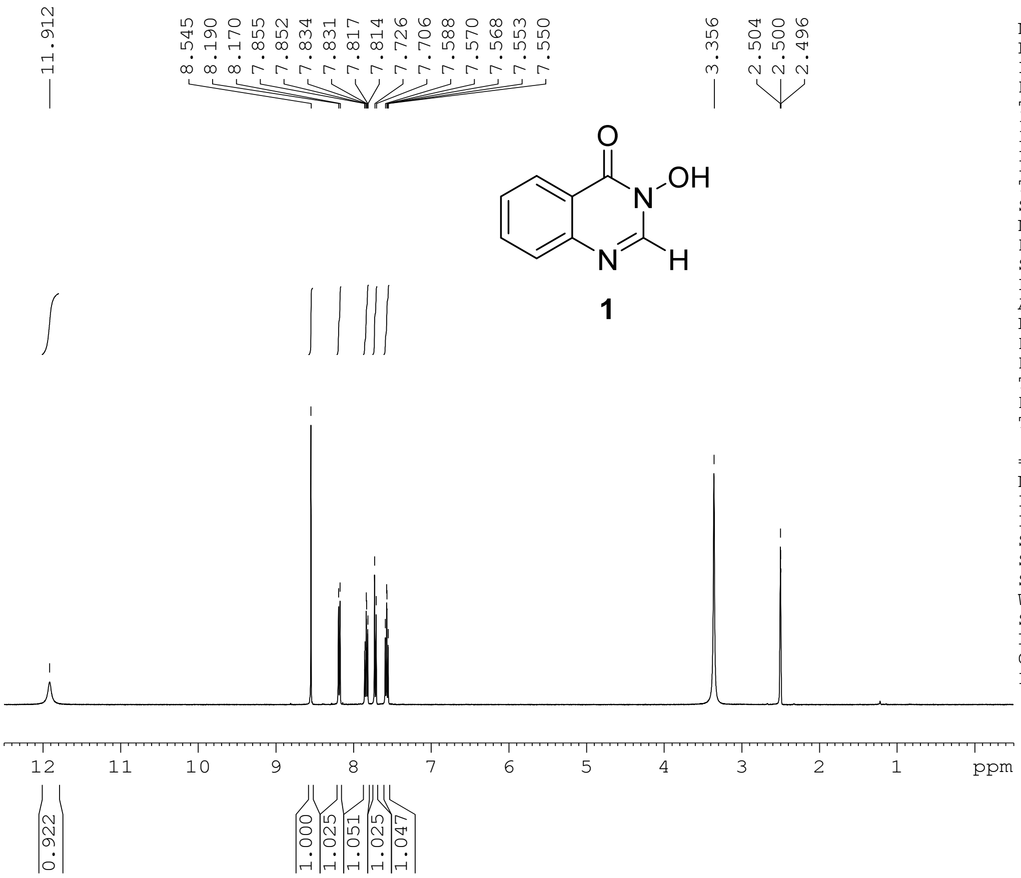

NAME	NHOH N=C
EXPNO	1
PROCNO	1
Date_	20210427
Time	12.10
INSTRUM	spect
PROBHD	5 mm DUL 13C-1
PULPROG	zg30
TD	32768
SOLVENT	DMSO
NS	32
DS	0
SWH	6410.256 Hz
FIDRES	0.195625 Hz
AQ	2.5559540 sec
RG	4
DW	78.000 usec
DE	6.00 usec
TE	300.0 K
D1	2.00000000 sec
TD0	1
======== CHANNEL f1 ========	
NUC1	1H
P1	10.00 usec
PL1	-2.40 dB
SFO1	400.1528010 MHz
SI	16384
SF	400.1500035 MHz
WDW	EM
SSB	0
LB	0.00 Hz
GB	0
PC	1.00

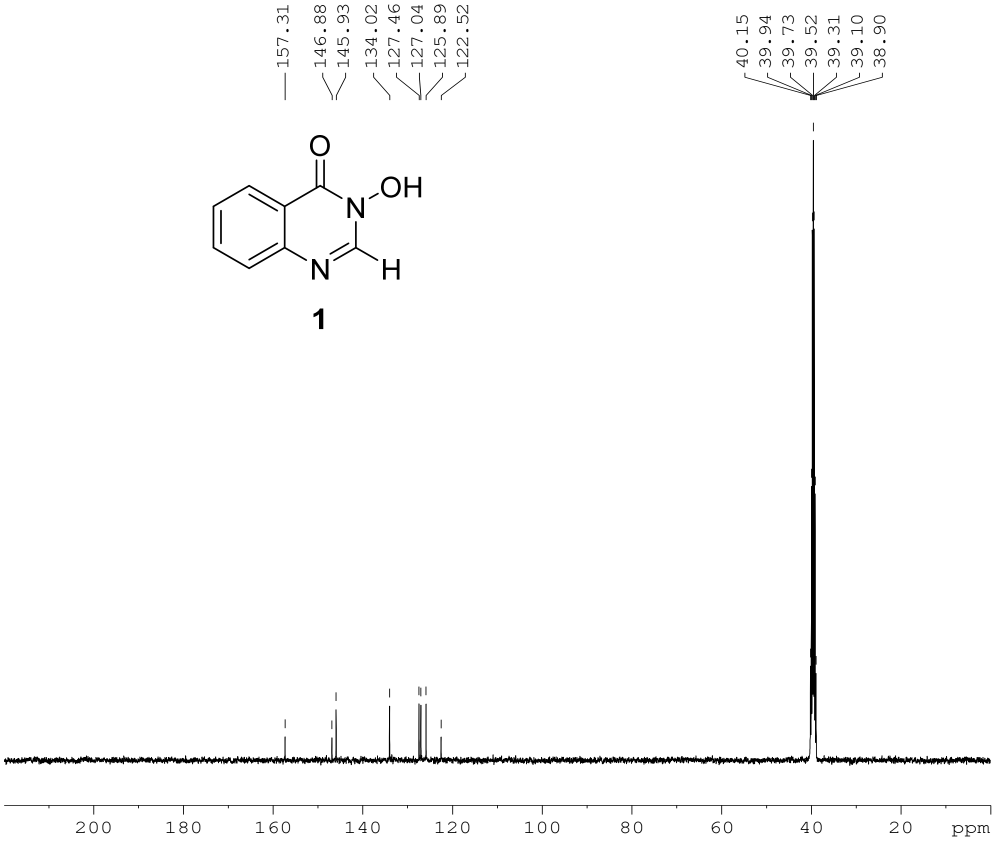

NAME	NHOH N=C
EXPNO	2
PROCNO	1
Date_	20210427
Time	12.14
INSTRUM	spect
PROBHD	5 mm DUL 13C-1
PULPROG	zgpg30
TD	65536
SOLVENT	DMSO
NS	200
DS	0
SWH	22727.273 Hz
FIDRES	0.346791 Hz
AQ	1.4418420 sec
RG	57
DW	22.000 usec
DE	6.00 usec
TE	300.0 K
D1	2.00000000 sec
d11	0.03000000 sec
DELTA	1.89999998 sec
TD0	1
======== CHANNEL f1 ========	
NUC1	13C
P1	9.70 usec
PL1	-0.50 dB
SFO1	100.6288660 MHz
======== CHANNEL f2 ========	
CPDPRG2	waltz16
NUC2	1H
PCPD2	90.00 usec
PL2	-2.40 dB
PL12	15.10 dB
PL13	18.10 dB
SFO2	400.1516010 MHz
SI	32768
SF	100.6178431 MHz
WDW	EM
SSB	0
LB	3.00 Hz
GB	0
PC	1.00

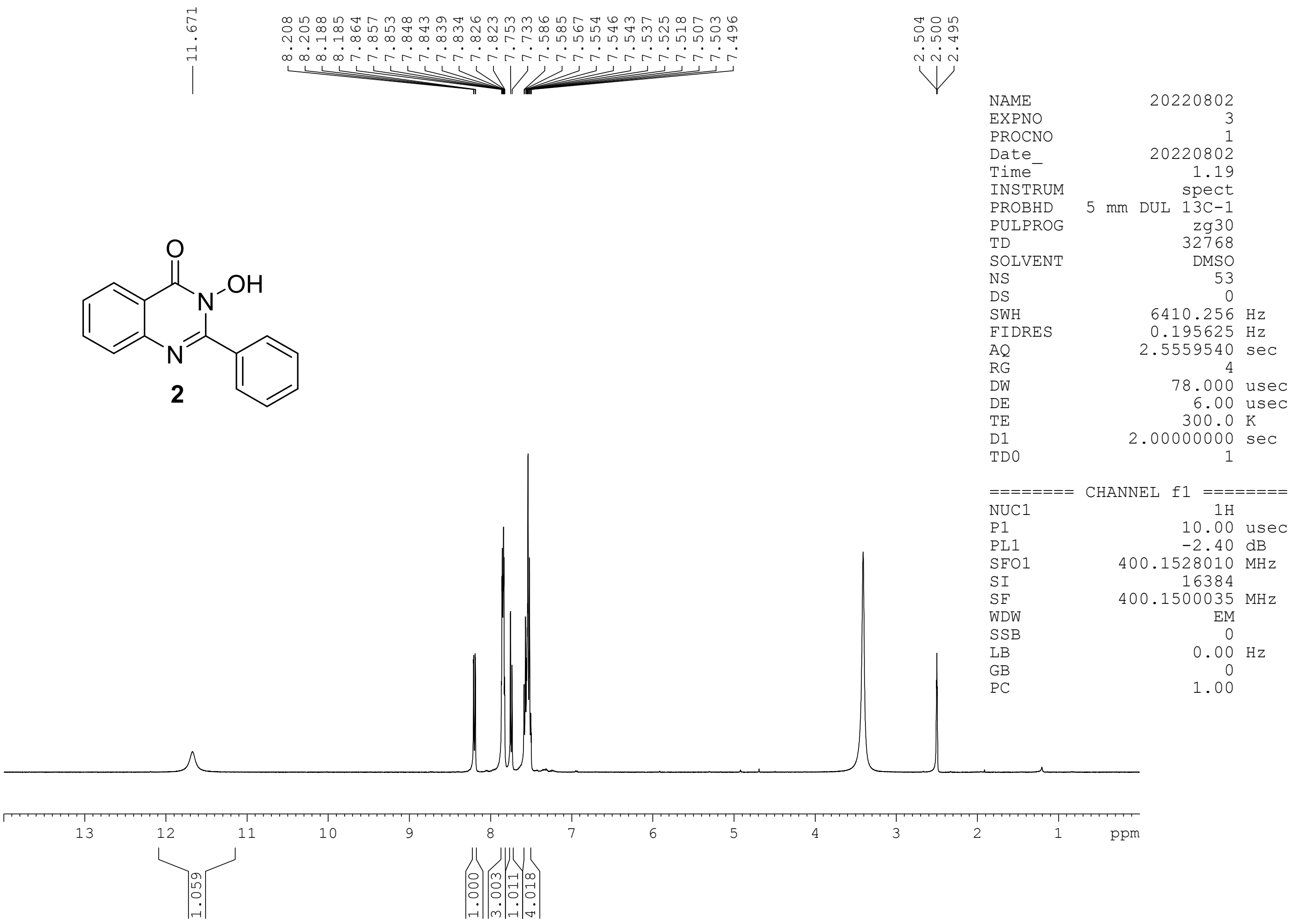
11.671
8.208 8.205 8.188 8.185 7.864 7.857 7.853 7.848 7.843 7.839 7.834 7.826 7.823 7.753 7.733 7.586 7.585 7.567 7.554 7.546 7.543 7.537 7.525 7.518 7.507 7.503 7.496
2.504 2.500 2.495
O
N
OH
N
2
NAME 20220802
EXPNO 3
PROCNO 1
Date_ 20220802
Time 1.19
INSTRUM spect
PROBHD 5 mm DUL 13C-1
PULPROG zg30
TD 32768
SOLVENT DMSO
NS 53
DS 0
SWH 6410.256 Hz
FIDRES 0.195625 Hz
AQ 2.5559540 sec
RG 4
DW 78.000 usec
DE 6.00 usec
TE 300.0 K
D1 2.00000000 sec
TD0 1
======== CHANNEL f1 ========
NUC1 1H
P1 10.00 usec
PL1 -2.40 dB
SFO1 400.1528010 MHz
SI 16384
SF 400.1500035 MHz
WDW EM
SSB 0
LB 0.00 Hz
GB 0
PC 1.00
13 12 11 10 9 8 7 6 5 4 3 2 1 ppm
1.059
1.000 3.003 1.011 4.018

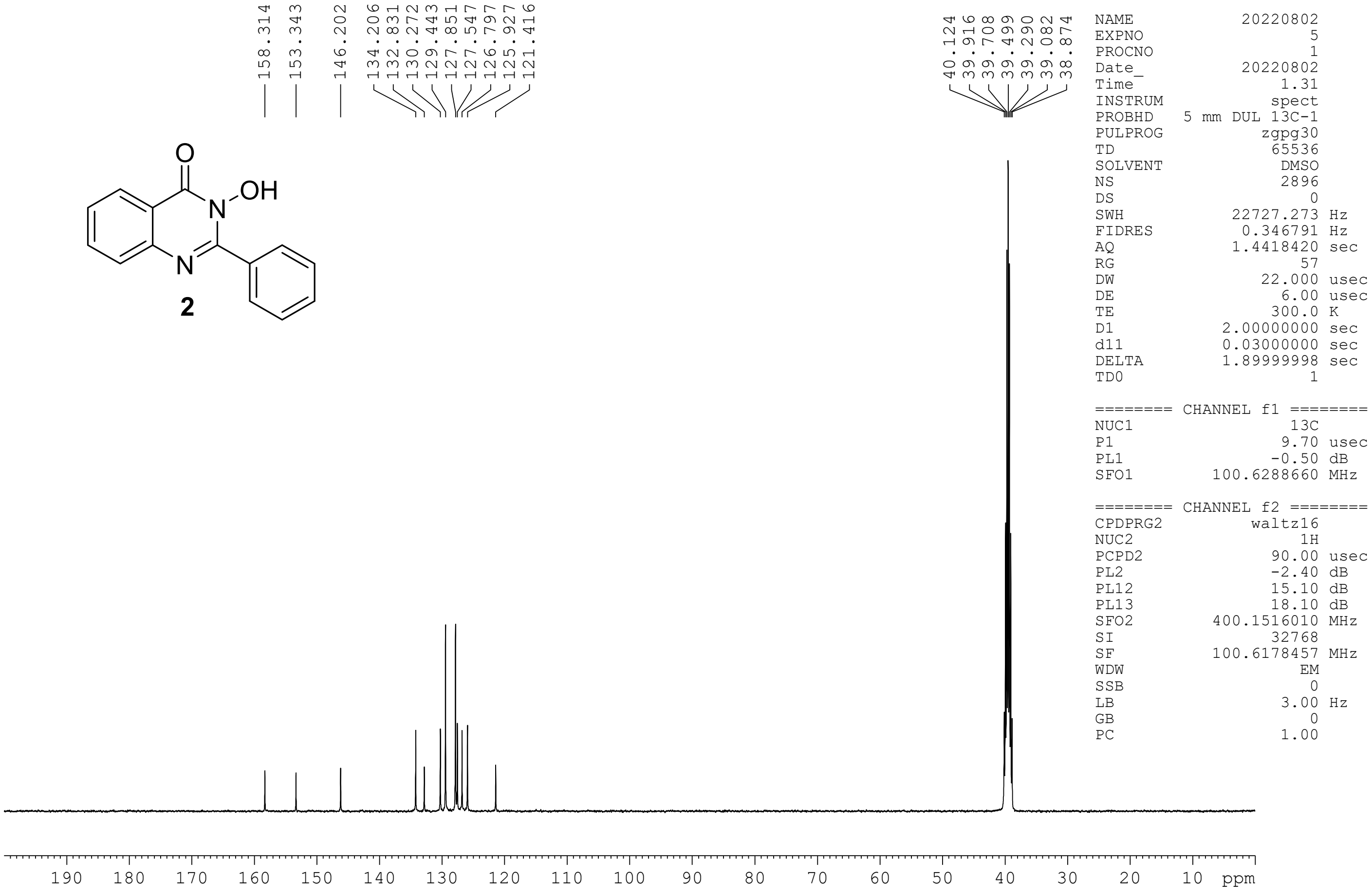
158.314
153.343
146.202
134.206 132.831 130.272 129.443 127.851 127.547 126.797 125.927 121.416
40.124 39.916 39.708 39.499 39.290 39.082 38.874
O
N
OH
N
2
NAME 20220802
EXPNO 5
PROCNO 1
Date_ 20220802
Time 1.31
INSTRUM spect
PROBHD 5 mm DUL 13C-1
PULPROG zgpg30
TD 65536
SOLVENT DMSO
NS 2896
DS 0
SWH 22727.273 Hz
FIDRES 0.346791 Hz
AQ 1.4418420 sec
RG 57
DW 22.000 usec
DE 6.00 usec
TE 300.0 K
D1 2.00000000 sec
d11 0.03000000 sec
DELTA 1.89999998 sec
TD0 1
======== CHANNEL f1 ========
NUC1 13C
P1 9.70 usec
PL1 -0.50 dB
SFO1 100.6288660 MHz
======== CHANNEL f2 ========
CPDPRG2 waltz16
NUC2 1H
PCPD2 90.00 usec
PL2 -2.40 dB
PL12 15.10 dB
PL13 18.10 dB
SFO2 400.1516010 MHz
SI 32768
SF 100.6178457 MHz
WDW EM
SSB 0
LB 3.00 Hz
GB 0
PC 1.00
190 180 170 160 150 140 130 120 110 100 90 80 70 60 50 40 30 20 10 ppm

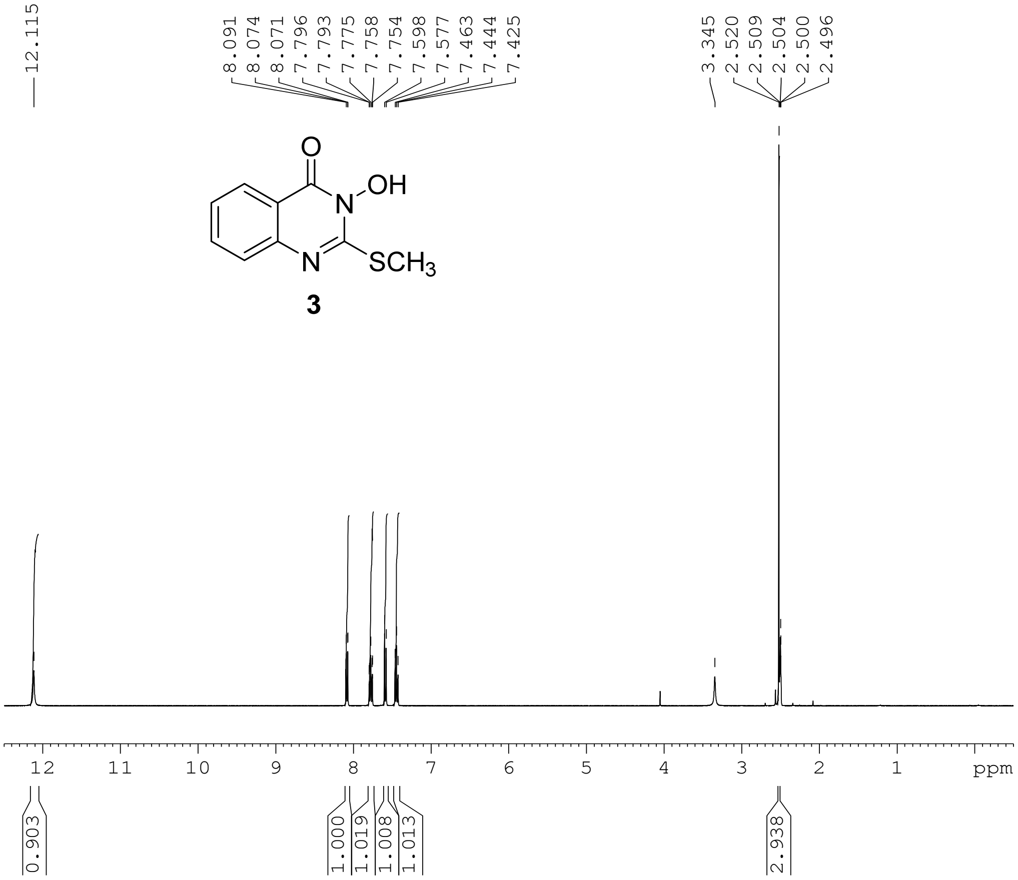

NAME s-ch3
EXPNO 10
PROCNO 1
Date_ 20210510
Time 21.04
INSTRUM spect
PROBHD 5 mm DUL 13C-1
PULPROG zg30
TD 32768
SOLVENT DMSO
NS 8
DS 0
SWH 6410.256 Hz
FIDRES 0.195625 Hz
AQ 2.5559540 sec
RG 4
DW 78.000 usec
DE 6.00 usec
TE 300.0 K
D1 2.00000000 sec
TD0 1

======== CHANNEL f1 ========
NUC1 1H
P1 10.00 usec
PL1 -2.40 dB
SFO1 400.1528010 MHz
SI 16384
SF 400.1500031 MHz
WDW EM
SSB 0
LB 0.00 Hz
GB 0
PC 1.00

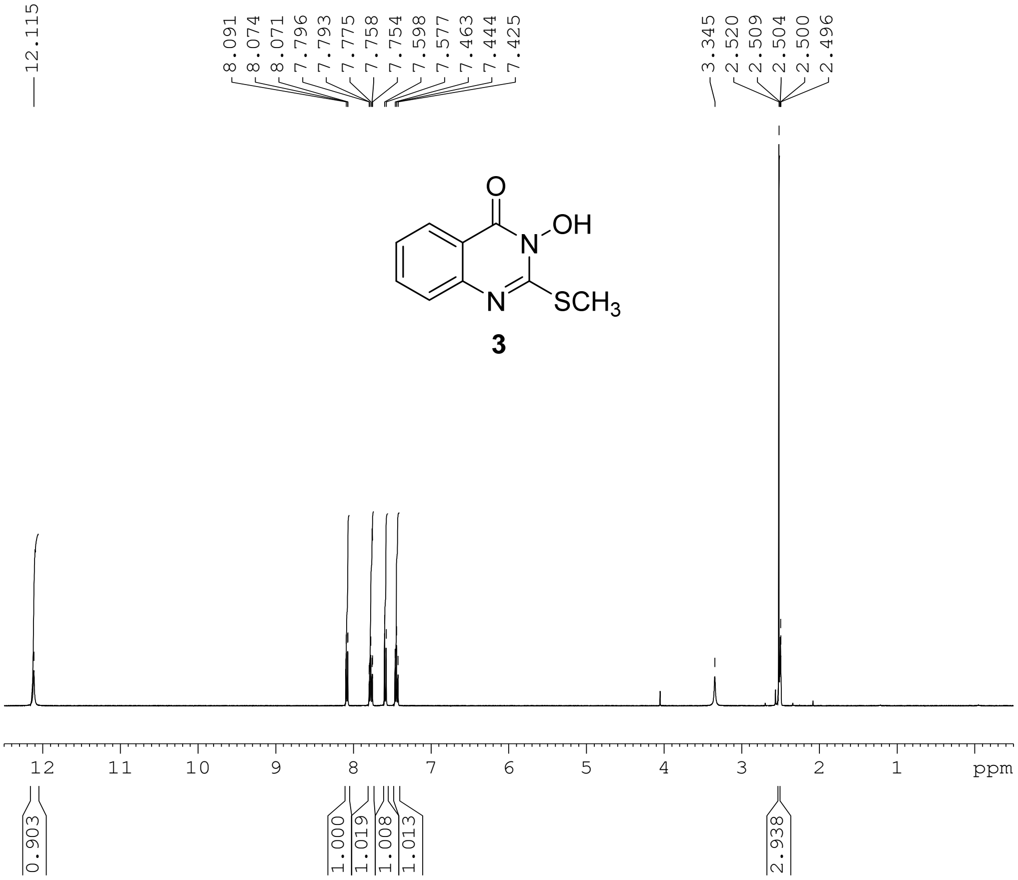

NAME s-ch3
EXPNO 10
PROCNO 1
Date_ 20210510
Time 21.04
INSTRUM spect
PROBHD 5 mm DUL 13C-1
PULPROG zg30
TD 32768
SOLVENT DMSO
NS 8
DS 0
SWH 6410.256 Hz
FIDRES 0.195625 Hz
AQ 2.5559540 sec
RG 4
DW 78.000 usec
DE 6.00 usec
TE 300.0 K
D1 2.00000000 sec
TD0 1

======== CHANNEL f1 ========
NUC1 1H
P1 10.00 usec
PL1 -2.40 dB
SFO1 400.1528010 MHz
SI 16384
SF 400.1500031 MHz
WDW EM
SSB 0
LB 0.00 Hz
GB 0
PC 1.00

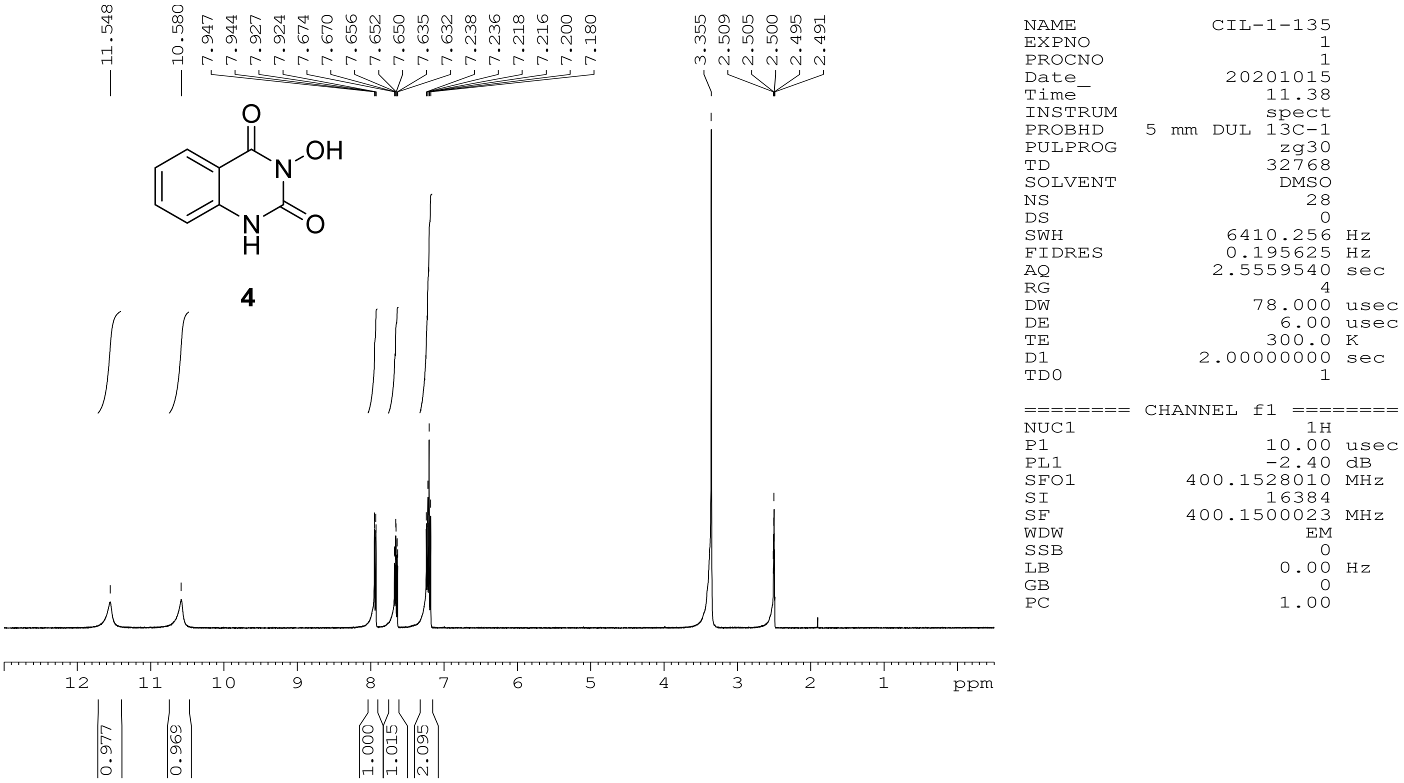
11.548
10.580
7.947 7.944 7.927 7.924 7.674 7.670 7.656 7.652 7.650 7.635 7.632 7.238 7.236 7.218 7.216 7.200 7.180
3.355 2.509 2.505 2.500 2.495 2.491
O
N
OH
N
H
O
4
12 11 10 9 8 7 6 5 4 3 2 1 ppm
0.977
0.969
1.000
1.015
2.095
NAME CIL-1-135
EXPNO 1
PROCNO 1
Date_ 20201015
Time 11.38
INSTRUM spect
PROBHD 5 mm DUL 13C-1
PULPROG zg30
TD 32768
SOLVENT DMSO
NS 28
DS 0
SWH 6410.256 Hz
FIDRES 0.195625 Hz
AQ 2.5559540 sec
RG 4
DW 78.000 usec
DE 6.00 usec
TE 300.0 K
D1 2.00000000 sec
TD0 1
======== CHANNEL f1 ========
NUC1 1H
P1 10.00 usec
PL1 -2.40 dB
SFO1 400.1528010 MHz
SI 16384
SF 400.1500023 MHz
WDW EM
SSB 0
LB 0.00 Hz
GB 0
PC 1.00

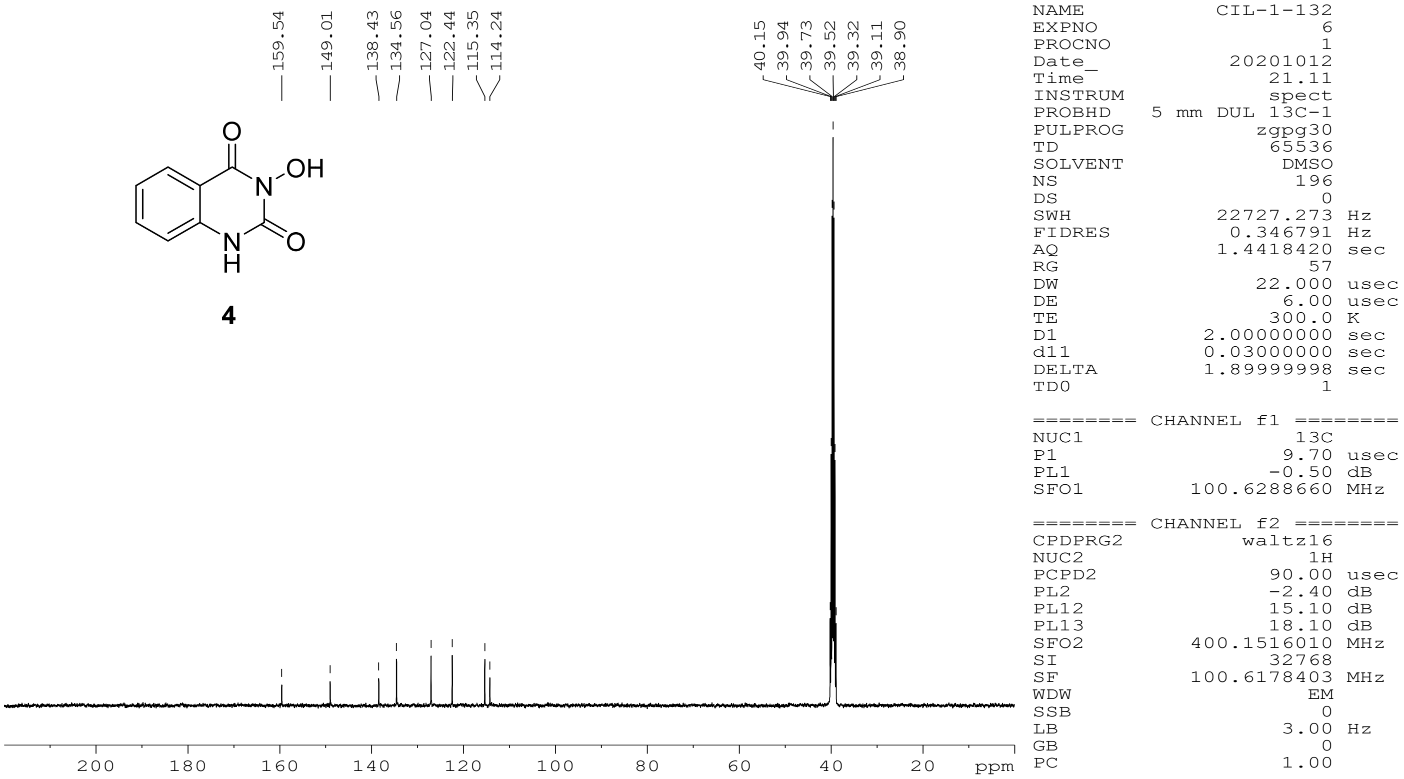
159.54
149.01
138.43 134.56
127.04 122.44
115.35 114.24
40.15 39.94 39.73 39.52 39.32 39.11 38.90
O
N
OH
N
H
O
4
200 180 160 140 120 100 80 60 40 20 ppm
NAME CIL-1-132
EXPNO 6
PROCNO 1
Date_ 20201012
Time 21.11
INSTRUM spect
PROBHD 5 mm DUL 13C-1
PULPROG zgpg30
TD 65536
SOLVENT DMSO
NS 196
DS 0
SWH 22727.273 Hz
FIDRES 0.346791 Hz
AQ 1.4418420 sec
RG 57
DW 22.000 usec
DE 6.00 usec
TE 300.0 K
D1 2.00000000 sec
d11 0.03000000 sec
DELTA 1.89999998 sec
TD0 1
======== CHANNEL f1 ========
NUC1 13C
P1 9.70 usec
PL1 -0.50 dB
SFO1 100.6288660 MHz
======== CHANNEL f2 ========
CPDPRG2 waltz16
NUC2 1H
PCPD2 90.00 usec
PL2 -2.40 dB
PL12 15.10 dB
PL13 18.10 dB
SFO2 400.1516010 MHz
SI 32768
SF 100.6178403 MHz
WDW EM
SSB 0
LB 3.00 Hz
GB 0
PC 1.00

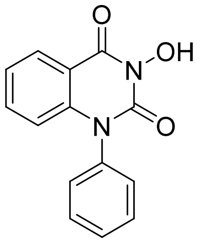

5

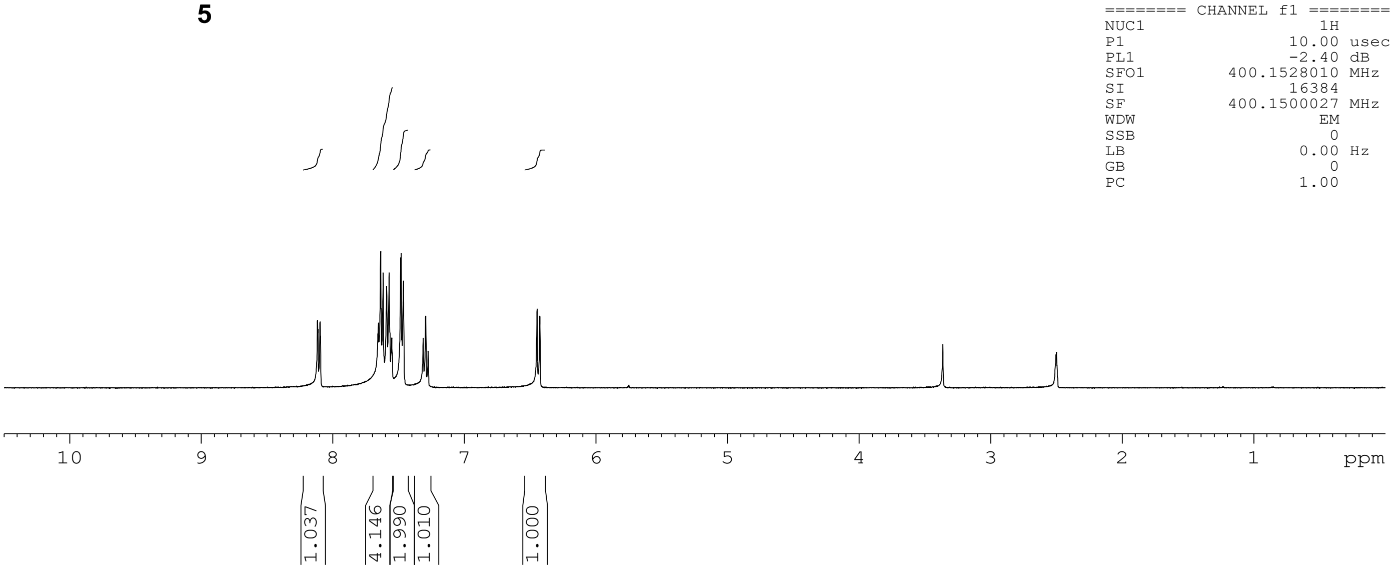
8.116
8.113
8.096
8.093
7.653
7.648
7.636
7.617
7.590
7.572
7.551
7.483
7.480
7.474
7.462
7.312
7.293
7.275
6.446
6.425
3.364
2.500
NAME YCC-1-57
EXPNO 1
PROCNO 1
Date_ 20210720
Time 19.09
INSTRUM spect
PROBHD 5 mm DUL 13C-1
PULPROG zg30
TD 32768
SOLVENT DMSO
NS 16
DS 0
SWH 6410.256 Hz
FIDRES 0.195625 Hz
AQ 2.5559540 sec
RG 4
DW 78.000 usec
DE 6.00 usec
TE 300.0 K
D1 2.00000000 sec
TD0 1
======== CHANNEL f1 ========
NUC1 1H
P1 10.00 usec
PL1 -2.40 dB
SFO1 400.1528010 MHz
SI 16384
SF 400.1500027 MHz
WDW EM
SSB 0
LB 0.00 Hz
GB 0
PC 1.00
10 9 8 7 6 5 4 3 2 1 ppm
1.037
4.146
1.990
1.010
1.000

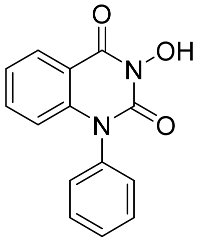

5

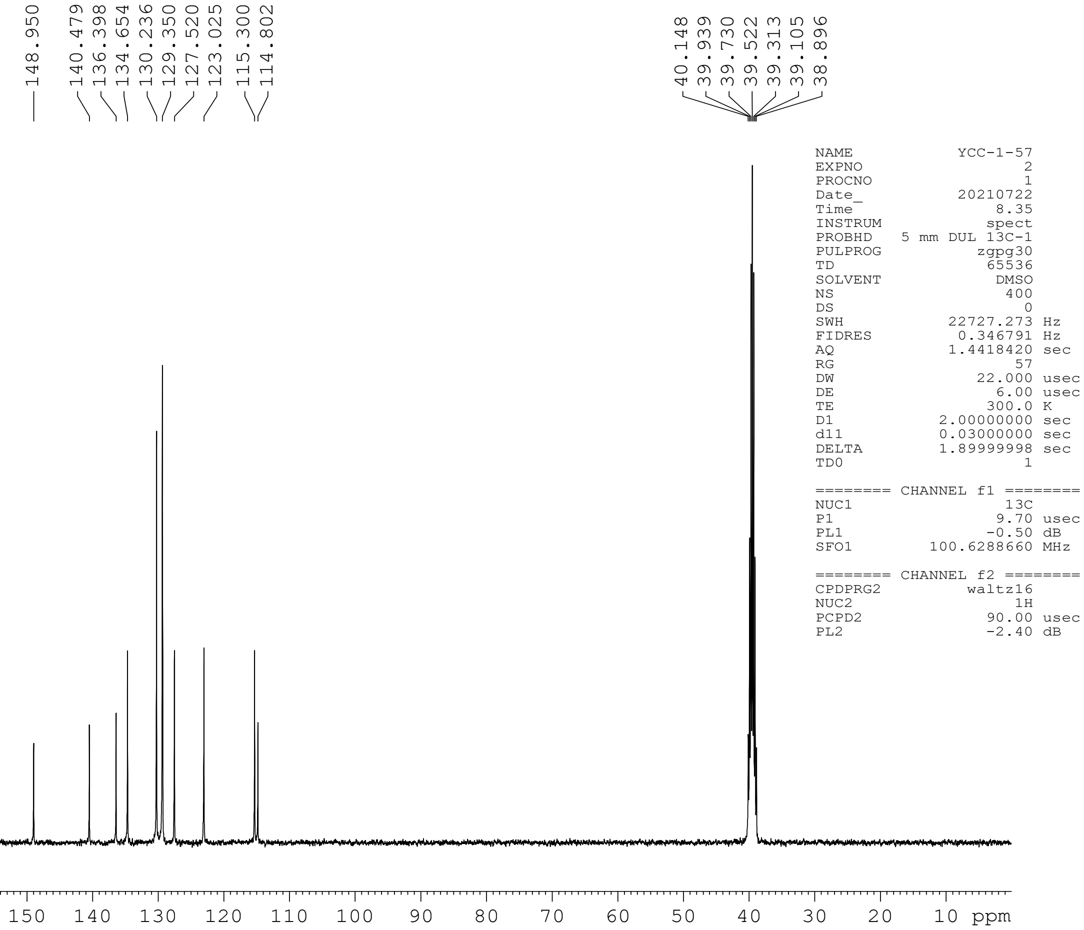
158.820
148.950
140.479
136.398
134.654
130.236
129.350
127.520
123.025
115.300
114.802
40.148
39.939
39.730
39.522
39.313
39.105
38.896
NAME YCC-1-57
EXPNO 2
PROCNO 1
Date_ 20210722
Time 8.35
INSTRUM spect
PROBHD 5 mm DUL 13C-1
PULPROG zgpg30
TD 65536
SOLVENT DMSO
NS 400
DS 0
SWH 22727.273 Hz
FIDRES 0.346791 Hz
AQ 1.4418420 sec
RG 57
DW 22.000 usec
DE 6.00 usec
TE 300.0 K
D1 2.00000000 sec
d11 0.03000000 sec
DELTA 1.89999998 sec
TD0 1
======== CHANNEL f1 ========
NUC1 13C
P1 9.70 usec
PL1 -0.50 dB
SFO1 100.6288660 MHz
======== CHANNEL f2 ========
CPDPRG2 waltz16
NUC2 1H
PCPD2 90.00 usec
PL2 -2.40 dB
190 180 170 160 150 140 130 120 110 100 90 80 70 60 50 40 30 20 10 ppm

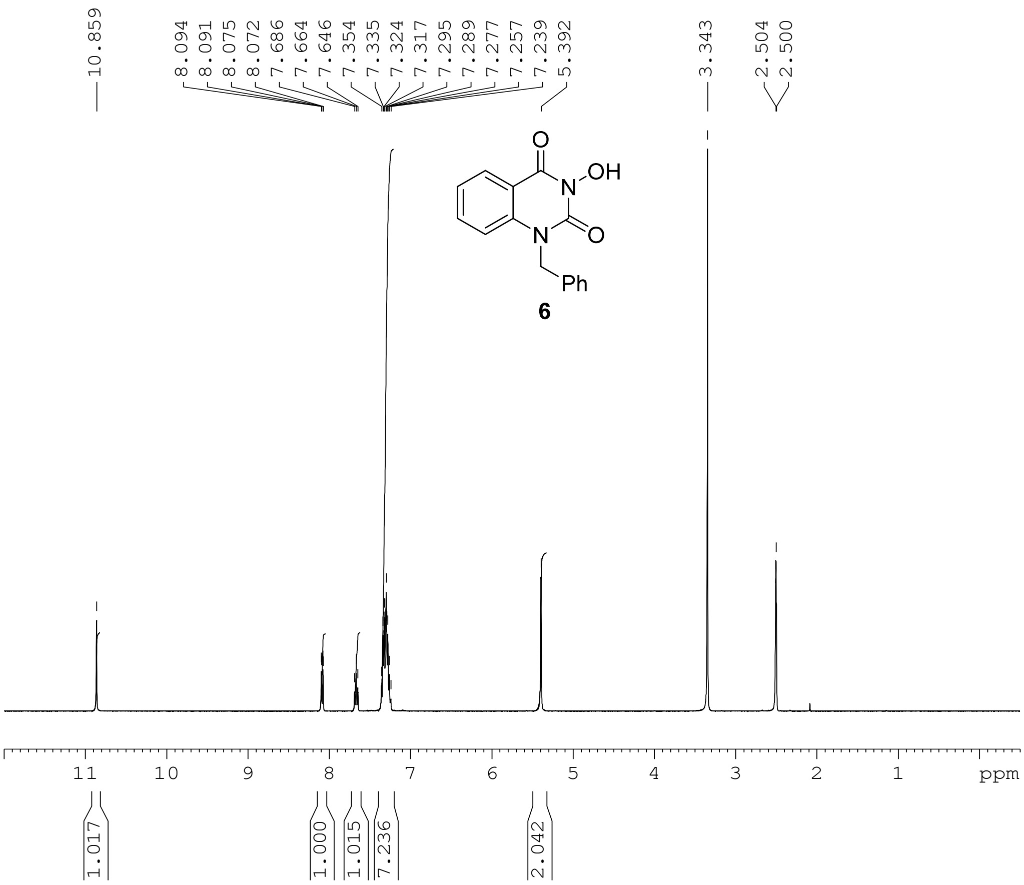


```
NAME             CIL-1-185
EXPNO                    5
PROCNO                   1
Date_             20210224
Time                 19.57
INSTRUM              spect
PROBHD   5 mm DUL 13C-1
PULPROG               zg30
TD                   32768
SOLVENT               DMSO
NS                      24
DS                       0
SWH               6410.256 Hz
FIDRES            0.195625 Hz
AQ               2.5559540 sec
RG                       4
DW                  78.000 usec
DE                    6.00 usec
TE                   300.0 K
D1              2.00000000 sec
TD0                      1

======== CHANNEL f1 ========
NUC1                    1H
P1                   10.00 usec
PL1                  -2.40 dB
SFO1           400.1528010 MHz
SI                   16384
SF             400.1500027 MHz
WDW                     EM
SSB                      0
LB                    0.00 Hz
GB                       0
PC                    1.00
```

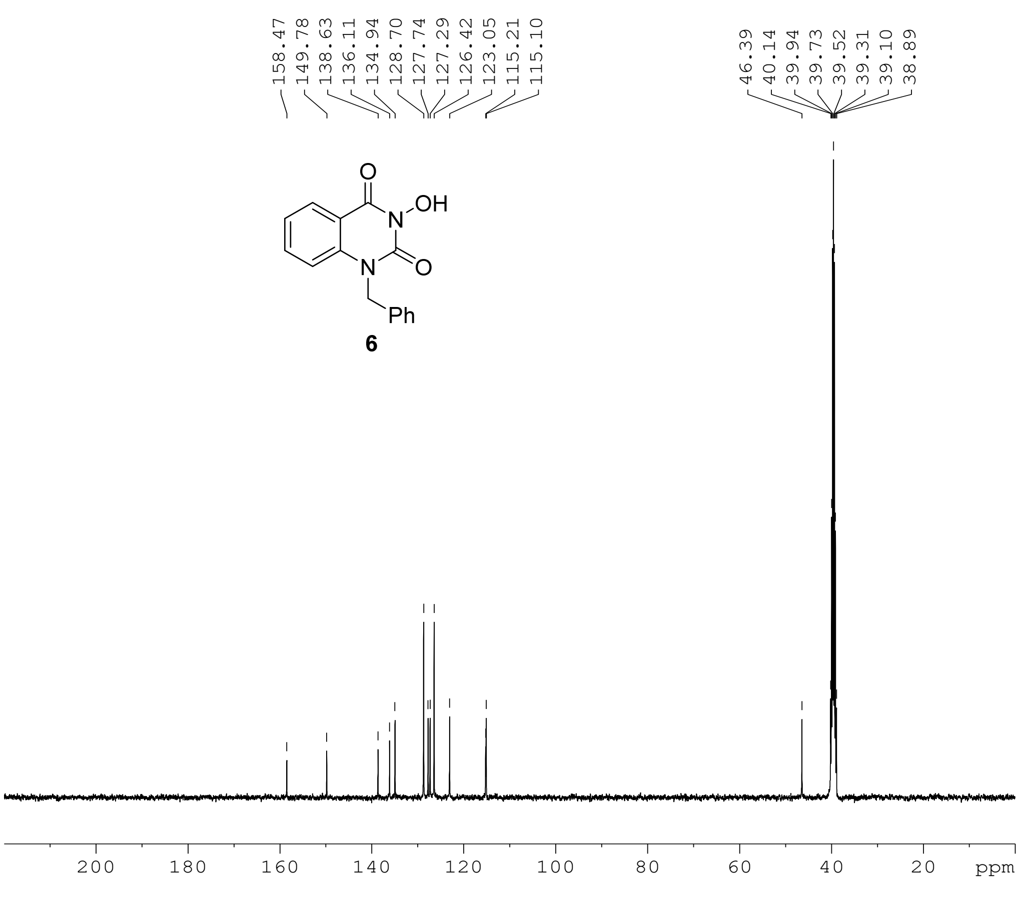


```
NAME             CIL-1-185
EXPNO                    4
PROCNO                   1
Date_             20210222
Time                 21.06
INSTRUM              spect
PROBHD   5 mm DUL 13C-1
PULPROG             zgpg30
TD                   65536
SOLVENT               DMSO
NS                     400
DS                       0
SWH              22727.273 Hz
FIDRES            0.346791 Hz
AQ               1.4418420 sec
RG                      57
DW                  22.000 usec
DE                    6.00 usec
TE                   300.0 K
D1              2.00000000 sec
d11             0.03000000 sec
DELTA           1.89999998 sec
TD0                      1

======== CHANNEL f1 ========
NUC1                   13C
P1                    9.70 usec
PL1                  -0.50 dB
SFO1           100.6288660 MHz

======== CHANNEL f2 ========
CPDPRG2            waltz16
NUC2                    1H
PCPD2                90.00 usec
PL2                  -2.40 dB
PL12                 15.10 dB
PL13                 18.10 dB
SFO2           400.1516010 MHz
SI                   32768
SF             100.6178466 MHz
WDW                     EM
SSB                      0
LB                    3.00 Hz
GB                       0
PC                    1.00
```

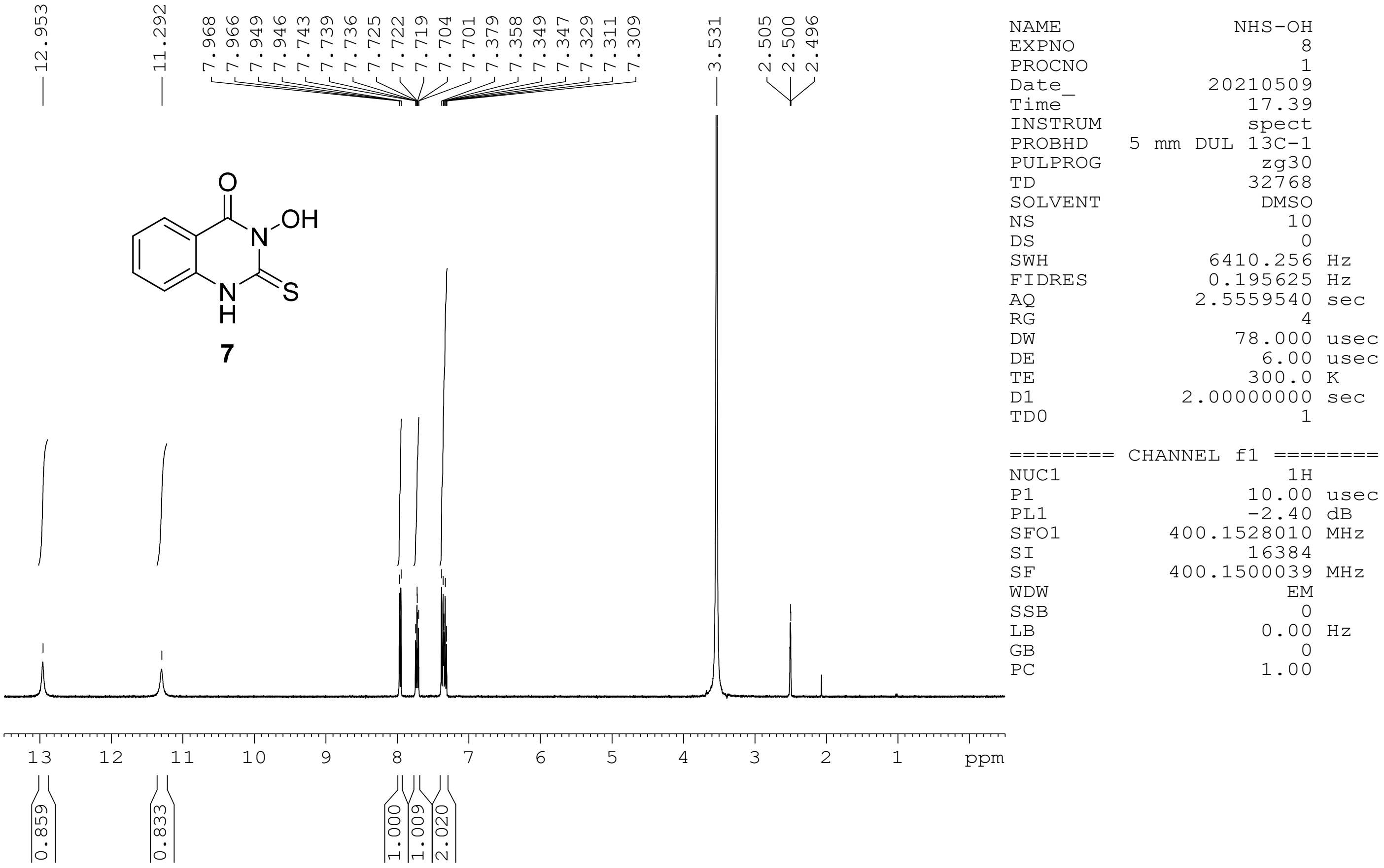
12.953
11.292
7.968 7.966 7.949 7.946 7.743 7.739 7.736 7.725 7.722 7.719 7.704 7.701 7.379 7.358 7.349 7.347 7.329 7.311 7.309
3.531
2.505 2.500 2.496
O
N
OH
N
H
S
7
0.859
0.833
1.000
1.009
2.020
13 12 11 10 9 8 7 6 5 4 3 2 1 ppm
NAME NHS-OH
EXPNO 8
PROCNO 1
Date_ 20210509
Time 17.39
INSTRUM spect
PROBHD 5 mm DUL 13C-1
PULPROG zg30
TD 32768
SOLVENT DMSO
NS 10
DS 0
SWH 6410.256 Hz
FIDRES 0.195625 Hz
AQ 2.5559540 sec
RG 4
DW 78.000 usec
DE 6.00 usec
TE 300.0 K
D1 2.00000000 sec
TD0 1
======== CHANNEL f1 ========
NUC1 1H
P1 10.00 usec
PL1 -2.40 dB
SFO1 400.1528010 MHz
SI 16384
SF 400.1500039 MHz
WDW EM
SSB 0
LB 0.00 Hz
GB 0
PC 1.00

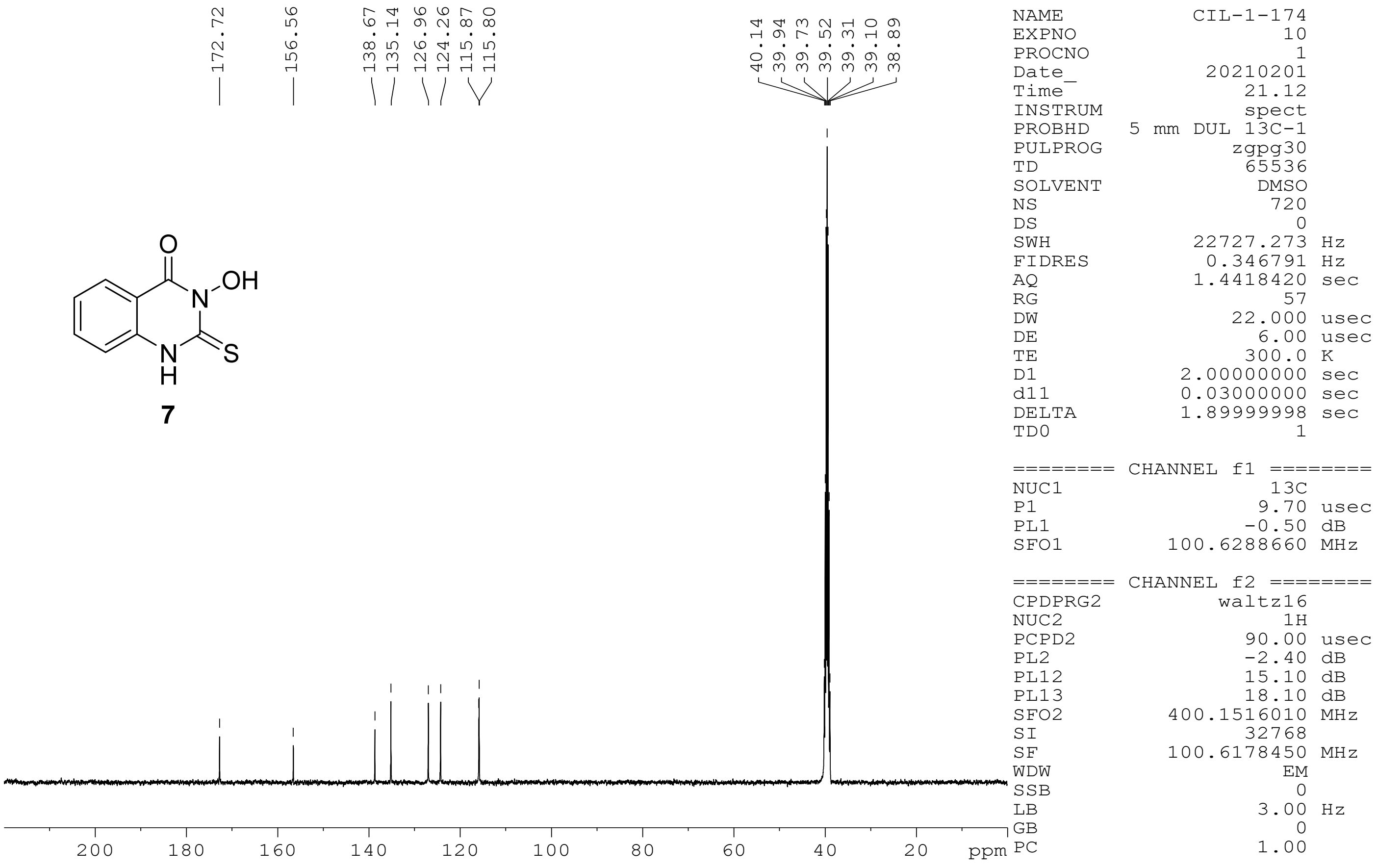
172.72
156.56
138.67 135.14 126.96 124.26 115.87 115.80
40.14 39.94 39.73 39.52 39.31 39.10 38.89
O
N
OH
N
H
S
7
200 180 160 140 120 100 80 60 40 20 ppm
NAME CIL-1-174
EXPNO 10
PROCNO 1
Date_ 20210201
Time 21.12
INSTRUM spect
PROBHD 5 mm DUL 13C-1
PULPROG zgpg30
TD 65536
SOLVENT DMSO
NS 720
DS 0
SWH 22727.273 Hz
FIDRES 0.346791 Hz
AQ 1.4418420 sec
RG 57
DW 22.000 usec
DE 6.00 usec
TE 300.0 K
D1 2.00000000 sec
d11 0.03000000 sec
DELTA 1.89999998 sec
TD0 1
======== CHANNEL f1 ========
NUC1 13C
P1 9.70 usec
PL1 -0.50 dB
SFO1 100.6288660 MHz
======== CHANNEL f2 ========
CPDPRG2 waltz16
NUC2 1H
PCPD2 90.00 usec
PL2 -2.40 dB
PL12 15.10 dB
PL13 18.10 dB
SFO2 400.1516010 MHz
SI 32768
SF 100.6178450 MHz
WDW EM
SSB 0
LB 3.00 Hz
GB 0
PC 1.00

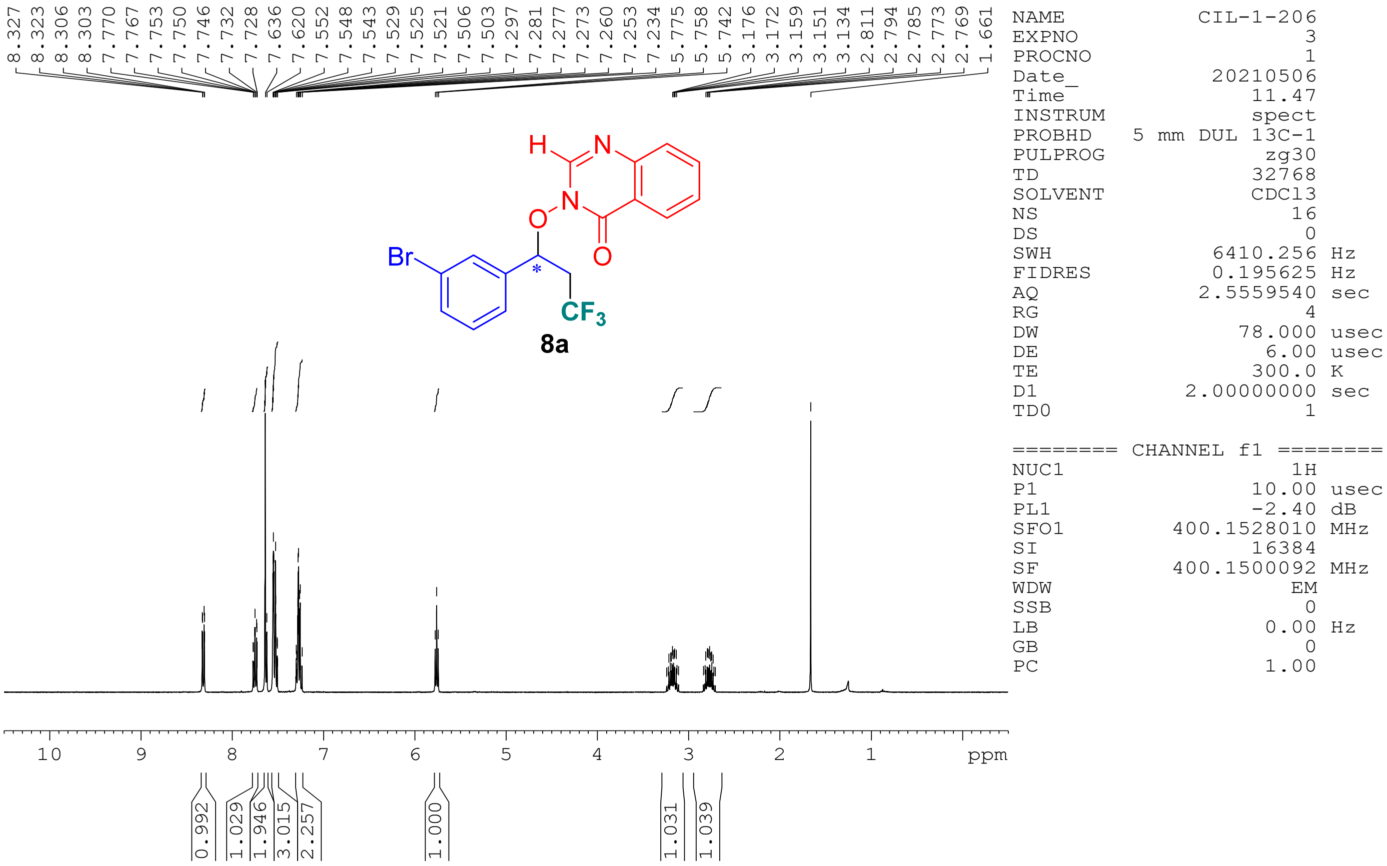

8.327 8.323 8.306 8.303 7.770 7.767 7.753 7.750 7.746 7.732 7.728 7.636 7.620 7.552 7.548 7.543 7.529 7.525 7.521 7.506 7.503 7.297 7.281 7.277 7.273 7.260 7.253 7.234 5.775 5.758 5.742 3.176 3.172 3.159 3.151 3.134 2.811 2.794 2.785 2.773 2.769 1.661
H
N
O
N
O
Br
*
CF3
8a
NAME CIL-1-206
EXPNO 3
PROCNO 1
Date_ 20210506
Time 11.47
INSTRUM spect
PROBHD 5 mm DUL 13C-1
PULPROG zg30
TD 32768
SOLVENT CDCl3
NS 16
DS 0
SWH 6410.256 Hz
FIDRES 0.195625 Hz
AQ 2.5559540 sec
RG 4
DW 78.000 usec
DE 6.00 usec
TE 300.0 K
D1 2.00000000 sec
TD0 1
======== CHANNEL f1 ========
NUC1 1H
P1 10.00 usec
PL1 -2.40 dB
SFO1 400.1528010 MHz
SI 16384
SF 400.1500092 MHz
WDW EM
SSB 0
LB 0.00 Hz
GB 0
PC 1.00
10 9 8 7 6 5 4 3 2 1 ppm
0.992 1.029 1.946 3.015 2.257 1.000 1.031 1.039

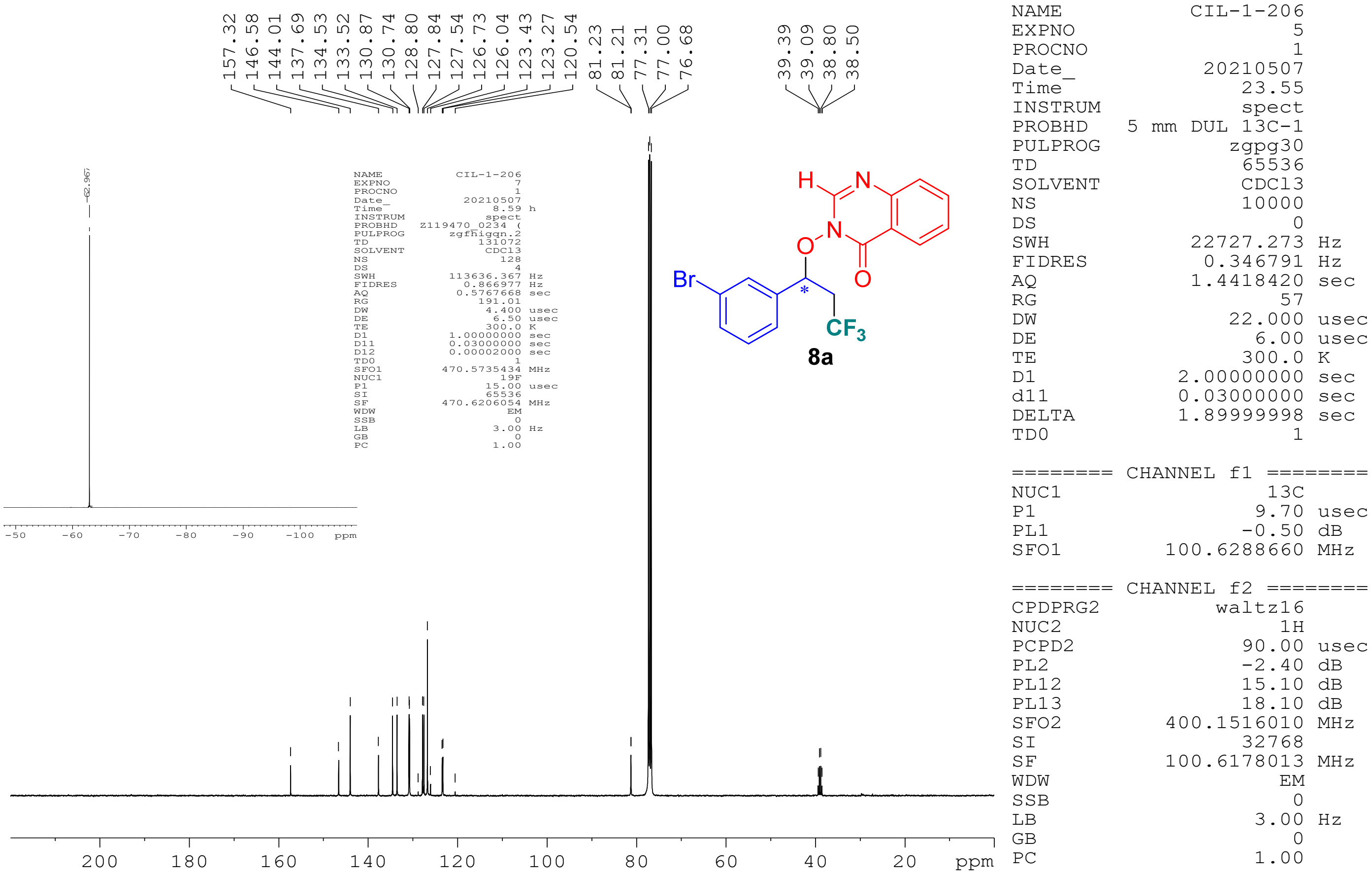

157.32 146.58 144.01 137.69 134.53 133.52 130.87 130.74 128.80 127.84 127.54 126.73 126.04 123.43 123.27 120.54 81.23 81.21 77.31 77.00 76.68 39.39 39.09 38.80 38.50
-62.967
-50 -60 -70 -80 -90 -100 ppm
H
N
O
N
O
Br
*
CF3
8a
NAME CIL-1-206
EXPNO 5
PROCNO 1
Date_ 20210507
Time 23.55
INSTRUM spect
PROBHD 5 mm DUL 13C-1
PULPROG zgpg30
TD 65536
SOLVENT CDCl3
NS 10000
DS 0
SWH 22727.273 Hz
FIDRES 0.346791 Hz
AQ 1.4418420 sec
RG 57
DW 22.000 usec
DE 6.00 usec
TE 300.0 K
D1 2.00000000 sec
d11 0.03000000 sec
DELTA 1.89999998 sec
TD0 1
======== CHANNEL f1 ========
NUC1 13C
P1 9.70 usec
PL1 -0.50 dB
SFO1 100.6288660 MHz
======== CHANNEL f2 ========
CPDPRG2 waltz16
NUC2 1H
PCPD2 90.00 usec
PL2 -2.40 dB
PL12 15.10 dB
PL13 18.10 dB
SFO2 400.1516010 MHz
SI 32768
SF 100.6178013 MHz
WDW EM
SSB 0
LB 3.00 Hz
GB 0
PC 1.00
200 180 160 140 120 100 80 60 40 20 ppm

(*S*)-**9a**

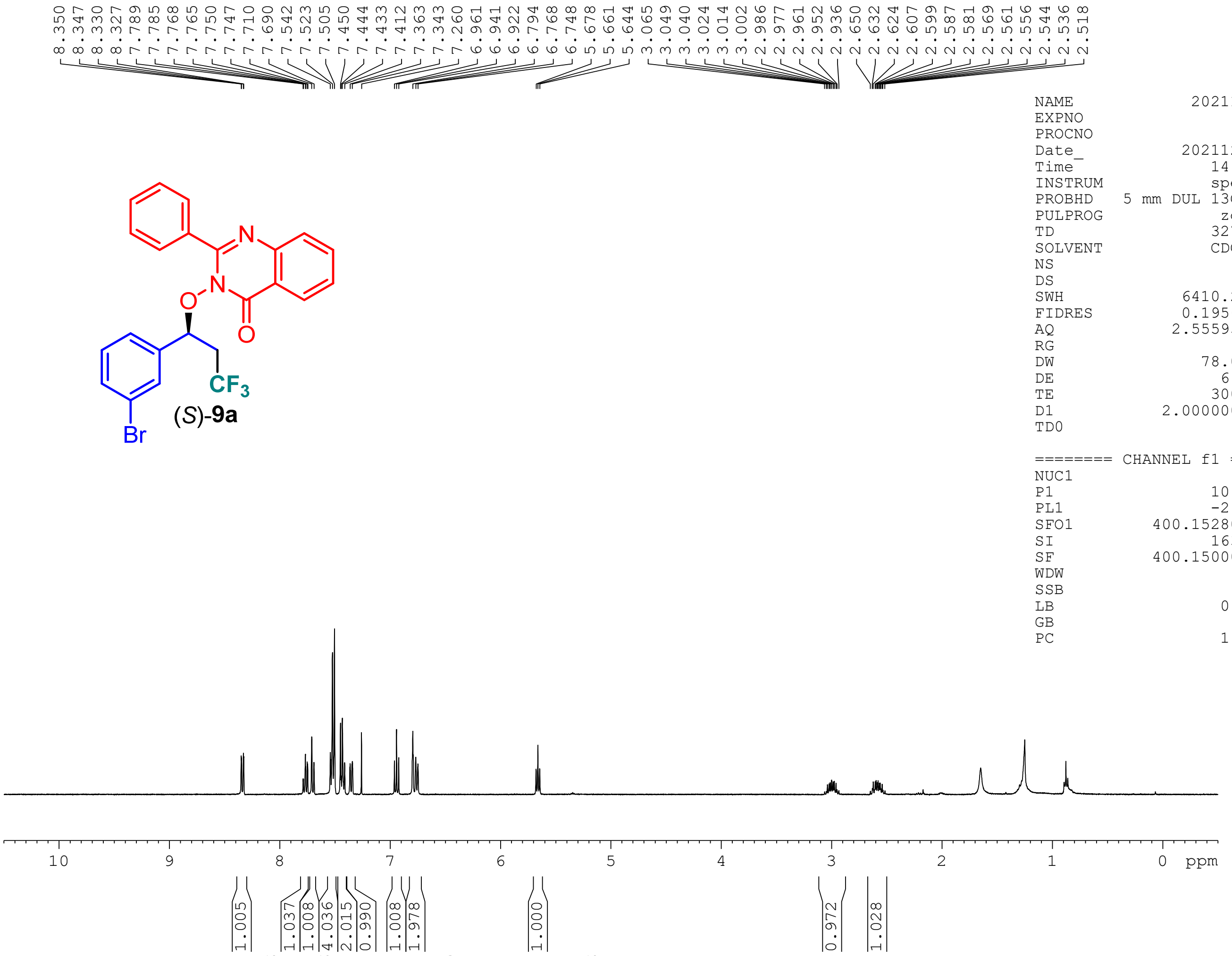

NAME 2021129
EXPNO 3
PROCNO 1
Date_ 20211229
Time 14.29
INSTRUM spect
PROBHD 5 mm DUL 13C-1
PULPROG zg30
TD 32768
SOLVENT CDCl3
NS 16
DS 0
SWH 6410.256 Hz
FIDRES 0.195625 Hz
AQ 2.5559540 sec
RG 4
DW 78.000 usec
DE 6.00 usec
TE 300.0 K
D1 2.00000000 sec
TD0 1

======== CHANNEL f1 ========
NUC1 1H
P1 10.00 usec
PL1 -2.40 dB
SFO1 400.1528010 MHz
SI 16384
SF 400.1500092 MHz
WDW EM
SSB 0
LB 0.00 Hz
GB 0
PC 1.00

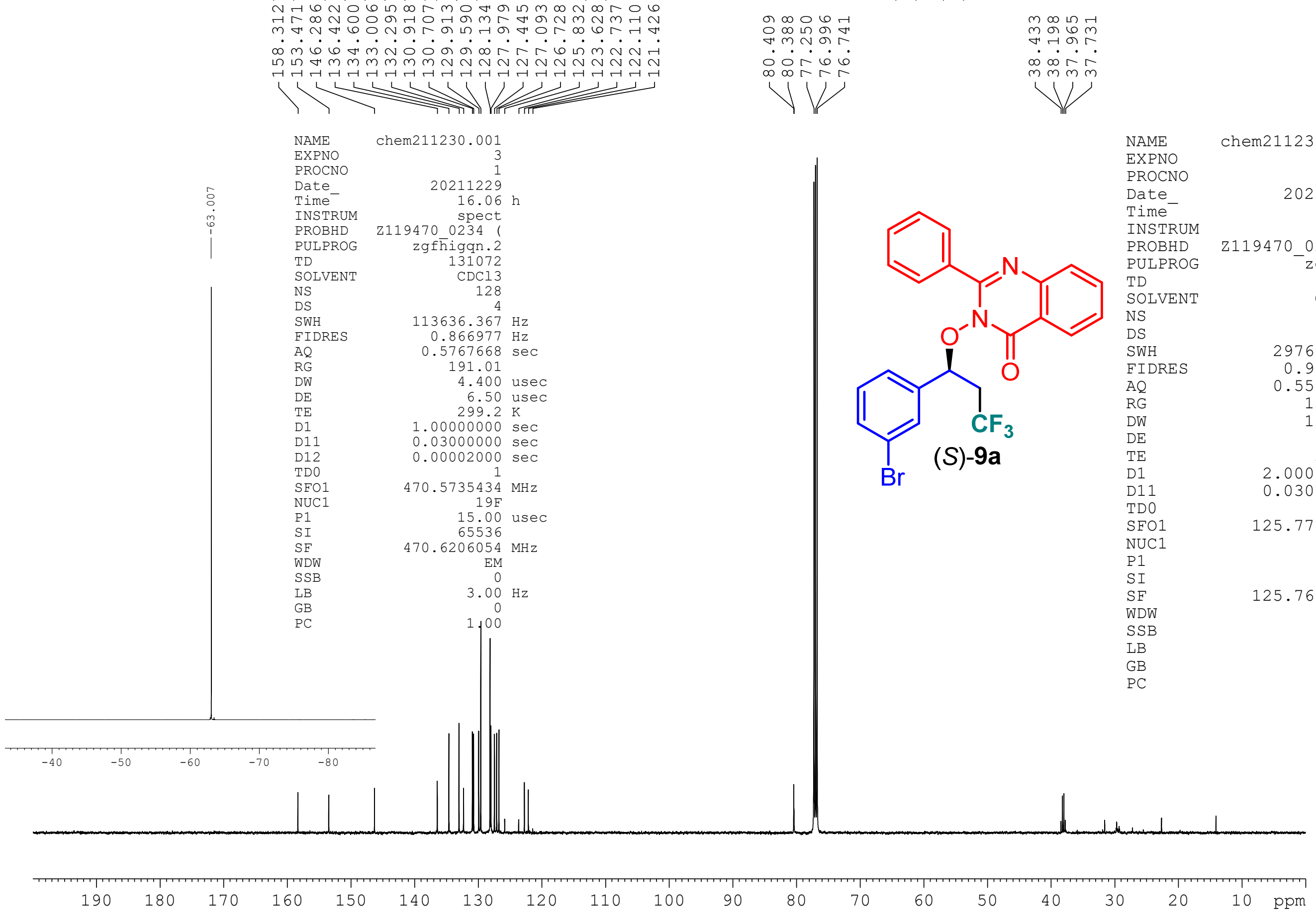

NAME chem211230.001
EXPNO 3
PROCNO 1
Date_ 20211229
Time 16.06 h
INSTRUM spect
PROBHD Z119470_0234 (
PULPROG zgfhigqn.2
TD 131072
SOLVENT CDCl3
NS 128
DS 4
SWH 113636.367 Hz
FIDRES 0.866977 Hz
AQ 0.5767668 sec
RG 191.01
DW 4.400 usec
DE 6.50 usec
TE 299.2 K
D1 1.00000000 sec
D11 0.03000000 sec
D12 0.00002000 sec
TD0 1
SFO1 470.5735434 MHz
NUC1 19F
P1 15.00 usec
SI 65536
SF 470.6206054 MHz
WDW EM
SSB 0
LB 3.00 Hz
GB 0
PC 1.00

(*S*)-**9a**

NAME chem211230.001
EXPNO 2
PROCNO 2
Date_ 20211230
Time 13.18 h
INSTRUM spect
PROBHD Z119470_0234 (
PULPROG zgpg30
TD 32768
SOLVENT CDCl3
NS 2485
DS 0
SWH 29761.904 Hz
FIDRES 0.908261 Hz
AQ 0.5505524 sec
RG 191.01
DW 16.800 usec
DE 6.50 usec
TE 299.9 K
D1 2.00000000 sec
D11 0.03000000 sec
TD0 1
SFO1 125.7785374 MHz
NUC1 13C
P1 10.00 usec
SI 32768
SF 125.7653353 MHz
WDW EM
SSB 0
LB 2.00 Hz
GB 0
PC 1.00

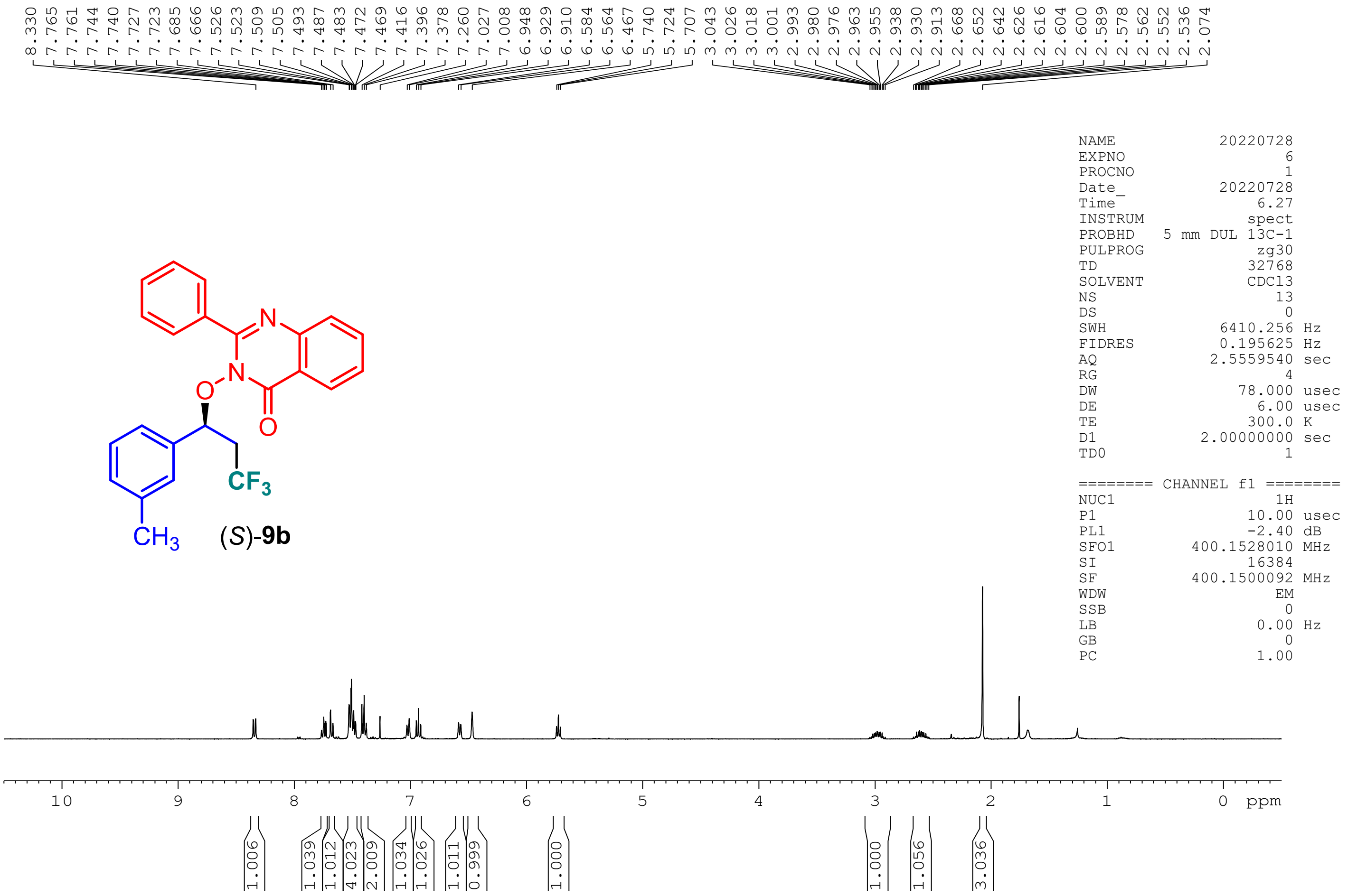
8.330 7.765 7.761 7.744 7.740 7.727 7.723 7.685 7.666 7.526 7.523 7.509 7.505 7.493 7.487 7.483 7.472 7.469 7.416 7.396 7.378 7.260 7.027 7.008 6.948 6.929 6.910 6.584 6.564 6.467 5.740 5.724 5.707 3.043 3.026 3.018 3.001 2.993 2.980 2.976 2.963 2.955 2.938 2.930 2.913 2.668 2.652 2.642 2.626 2.616 2.604 2.600 2.589 2.578 2.562 2.552 2.536 2.074
N
O
O
N
CF3
CH3
(S)-9b
NAME 20220728
EXPNO 6
PROCNO 1
Date_ 20220728
Time 6.27
INSTRUM spect
PROBHD 5 mm DUL 13C-1
PULPROG zg30
TD 32768
SOLVENT CDCl3
NS 13
DS 0
SWH 6410.256 Hz
FIDRES 0.195625 Hz
AQ 2.5559540 sec
RG 4
DW 78.000 usec
DE 6.00 usec
TE 300.0 K
D1 2.00000000 sec
TD0 1
======== CHANNEL f1 ========
NUC1 1H
P1 10.00 usec
PL1 -2.40 dB
SFO1 400.1528010 MHz
SI 16384
SF 400.1500092 MHz
WDW EM
SSB 0
LB 0.00 Hz
GB 0
PC 1.00
10 9 8 7 6 5 4 3 2 1 0 ppm
1.006 1.039 1.012 4.023 2.009 1.034 1.026 1.011 0.999 1.000 1.000 1.056 3.036

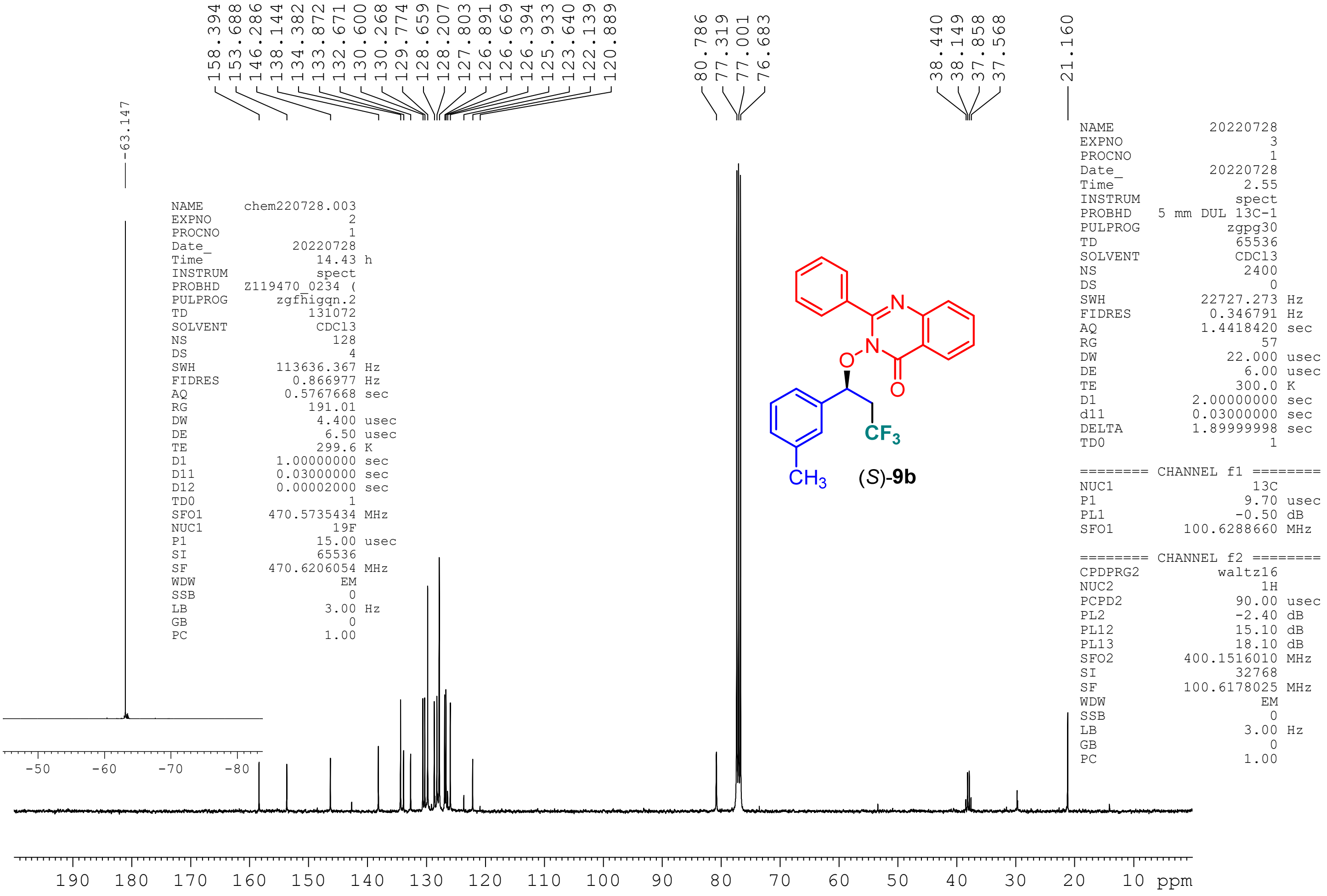
158.394 153.688 146.286 138.144 134.382 133.872 132.671 130.600 130.268 129.774 128.659 128.207 127.803 126.891 126.669 126.394 125.933 123.640 122.139 120.889
80.786 77.319 77.001 76.683
38.440 38.149 37.858 37.568
21.160
-63.147
NAME chem220728.003
EXPNO 2
PROCNO 1
Date_ 20220728
Time 14.43 h
INSTRUM spect
PROBHD Z119470_0234 (
PULPROG zgfhigqn.2
TD 131072
SOLVENT CDCl3
NS 128
DS 4
SWH 113636.367 Hz
FIDRES 0.866977 Hz
AQ 0.5767668 sec
RG 191.01
DW 4.400 usec
DE 6.50 usec
TE 299.6 K
D1 1.00000000 sec
D11 0.03000000 sec
D12 0.00002000 sec
TD0 1
SFO1 470.5735434 MHz
NUC1 19F
P1 15.00 usec
SI 65536
SF 470.6206054 MHz
WDW EM
SSB 0
LB 3.00 Hz
GB 0
PC 1.00
-50 -60 -70 -80
N
O
O
N
CF3
CH3
(S)-9b
NAME 20220728
EXPNO 3
PROCNO 1
Date_ 20220728
Time 2.55
INSTRUM spect
PROBHD 5 mm DUL 13C-1
PULPROG zgpg30
TD 65536
SOLVENT CDCl3
NS 2400
DS 0
SWH 22727.273 Hz
FIDRES 0.346791 Hz
AQ 1.4418420 sec
RG 57
DW 22.000 usec
DE 6.00 usec
TE 300.0 K
D1 2.00000000 sec
d11 0.03000000 sec
DELTA 1.89999998 sec
TD0 1
======== CHANNEL f1 ========
NUC1 13C
P1 9.70 usec
PL1 -0.50 dB
SFO1 100.6288660 MHz
======== CHANNEL f2 ========
CPDPRG2 waltz16
NUC2 1H
PCPD2 90.00 usec
PL2 -2.40 dB
PL12 15.10 dB
PL13 18.10 dB
SFO2 400.1516010 MHz
SI 32768
SF 100.6178025 MHz
WDW EM
SSB 0
LB 3.00 Hz
GB 0
PC 1.00
190 180 170 160 150 140 130 120 110 100 90 80 70 60 50 40 30 20 10 ppm

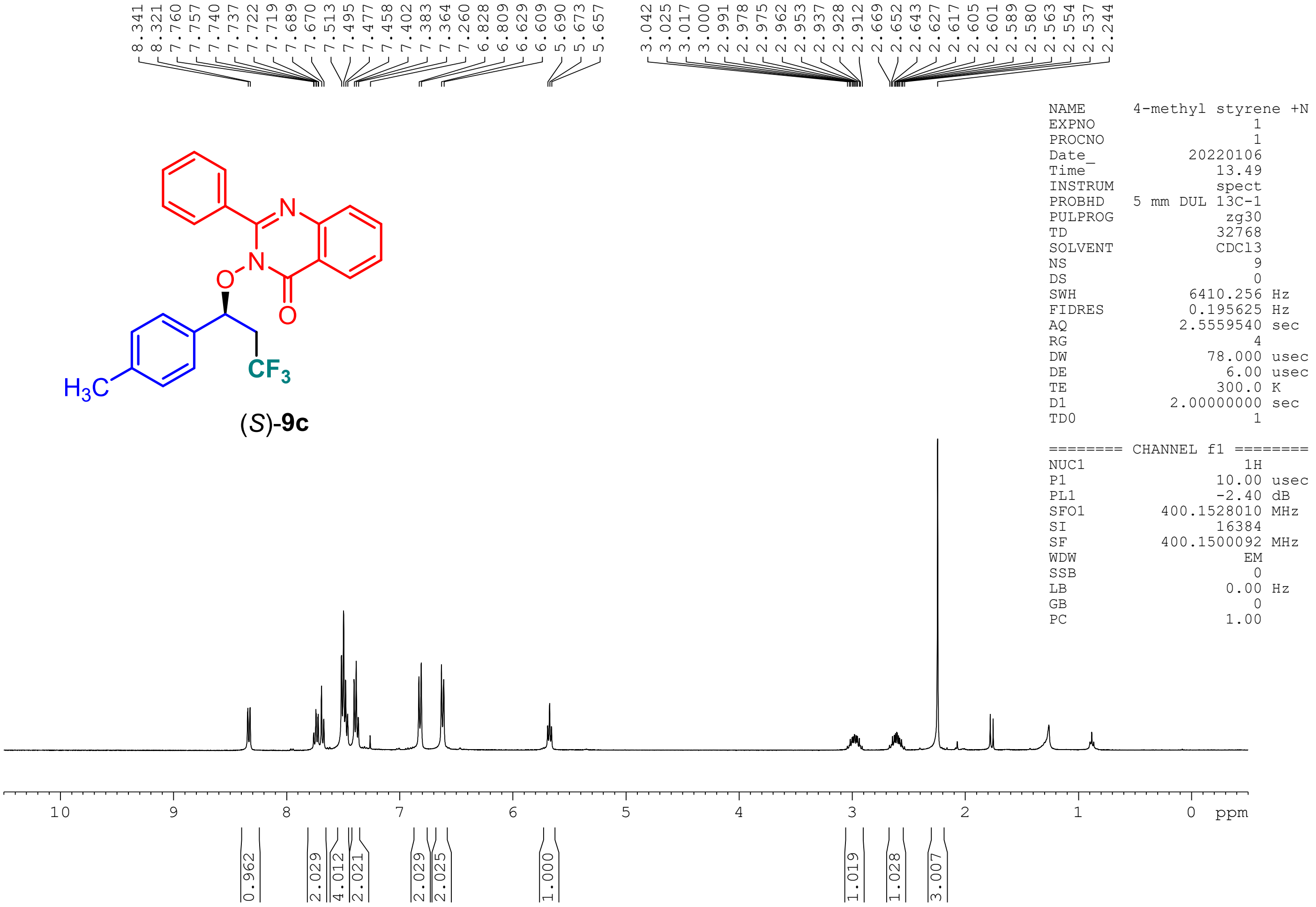
8.341
8.321
7.760
7.757
7.740
7.737
7.722
7.719
7.689
7.670
7.513
7.495
7.477
7.458
7.402
7.383
7.364
7.260
6.828
6.809
6.629
6.609
5.690
5.673
5.657
3.042
3.025
3.017
3.000
2.991
2.978
2.975
2.962
2.953
2.937
2.928
2.912
2.669
2.652
2.643
2.627
2.617
2.605
2.601
2.589
2.580
2.563
2.554
2.537
2.244
N
N
O
O
H3C
CF3
(S)-9c
NAME 4-methyl styrene +N
EXPNO 1
PROCNO 1
Date_ 20220106
Time 13.49
INSTRUM spect
PROBHD 5 mm DUL 13C-1
PULPROG zg30
TD 32768
SOLVENT CDCl3
NS 9
DS 0
SWH 6410.256 Hz
FIDRES 0.195625 Hz
AQ 2.5559540 sec
RG 4
DW 78.000 usec
DE 6.00 usec
TE 300.0 K
D1 2.00000000 sec
TD0 1
======== CHANNEL f1 ========
NUC1 1H
P1 10.00 usec
PL1 -2.40 dB
SFO1 400.1528010 MHz
SI 16384
SF 400.1500092 MHz
WDW EM
SSB 0
LB 0.00 Hz
GB 0
PC 1.00
10 9 8 7 6 5 4 3 2 1 0 ppm
0.962
2.029
4.012
2.021
2.029
2.025
1.000
1.019
1.028
3.007

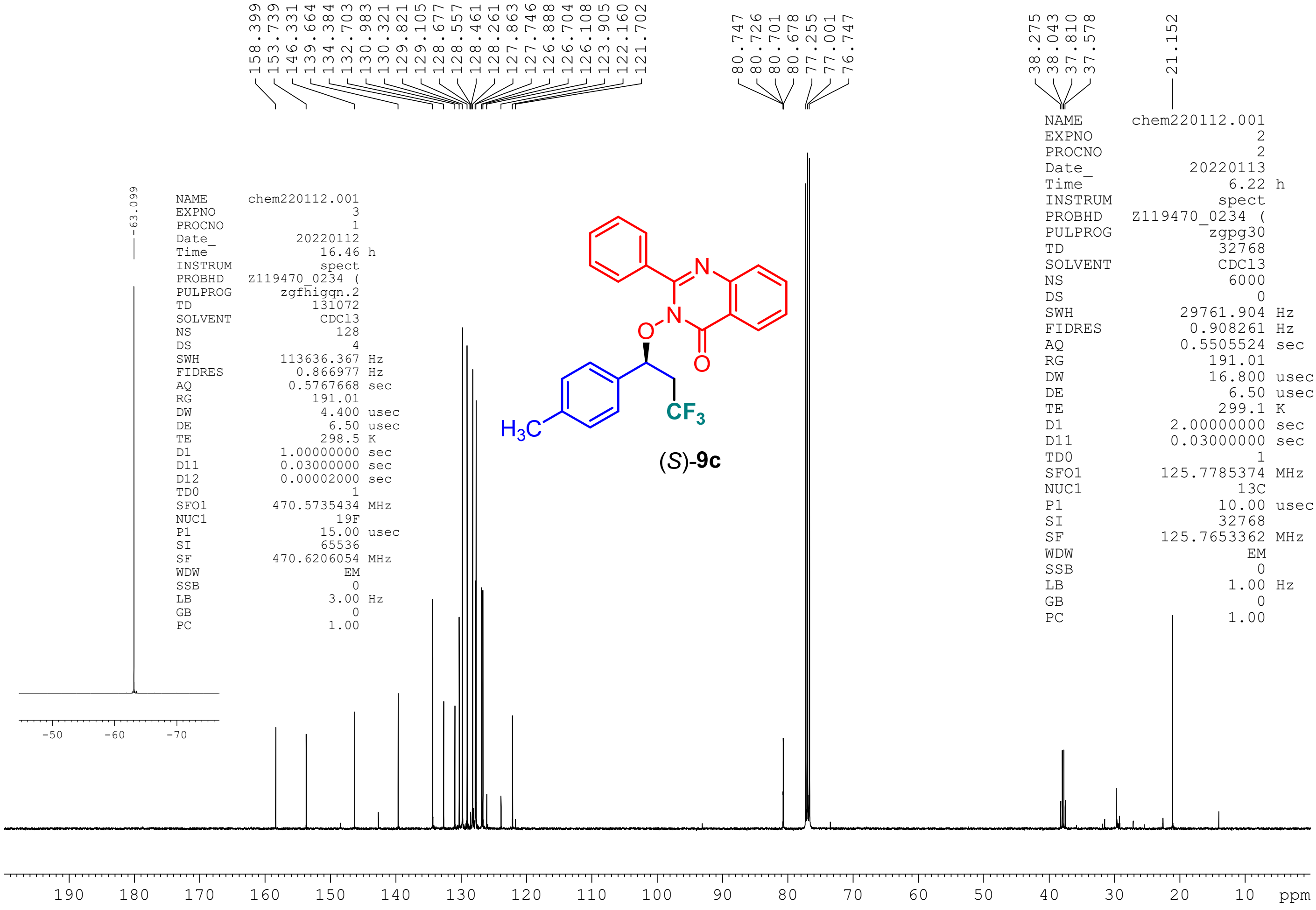
158.399
153.739
146.331
139.664
134.384
132.703
130.983
130.321
129.821
129.105
128.677
128.557
128.461
128.261
127.863
127.746
126.888
126.704
126.108
123.905
122.160
121.702
80.747
80.726
80.701
80.678
77.255
77.001
76.747
38.275
38.043
37.810
37.578
21.152
NAME chem220112.001
EXPNO 2
PROCNO 2
Date_ 20220113
Time 6.22 h
INSTRUM spect
PROBHD Z119470_0234 (
PULPROG zgpg30
TD 32768
SOLVENT CDCl3
NS 6000
DS 0
SWH 29761.904 Hz
FIDRES 0.908261 Hz
AQ 0.5505524 sec
RG 191.01
DW 16.800 usec
DE 6.50 usec
TE 299.1 K
D1 2.00000000 sec
D11 0.03000000 sec
TD0 1
SFO1 125.7785374 MHz
NUC1 13C
P1 10.00 usec
SI 32768
SF 125.7653362 MHz
WDW EM
SSB 0
LB 1.00 Hz
GB 0
PC 1.00
-63.099
NAME chem220112.001
EXPNO 3
PROCNO 1
Date_ 20220112
Time 16.46 h
INSTRUM spect
PROBHD Z119470_0234 (
PULPROG zgfhigqn.2
TD 131072
SOLVENT CDCl3
NS 128
DS 4
SWH 113636.367 Hz
FIDRES 0.866977 Hz
AQ 0.5767668 sec
RG 191.01
DW 4.400 usec
DE 6.50 usec
TE 298.5 K
D1 1.00000000 sec
D11 0.03000000 sec
D12 0.00002000 sec
TD0 1
SFO1 470.5735434 MHz
NUC1 19F
P1 15.00 usec
SI 65536
SF 470.6206054 MHz
WDW EM
SSB 0
LB 3.00 Hz
GB 0
PC 1.00
-50 -60 -70
N
N
O
O
H3C
CF3
(S)-9c
190 180 170 160 150 140 130 120 110 100 90 80 70 60 50 40 30 20 10 ppm

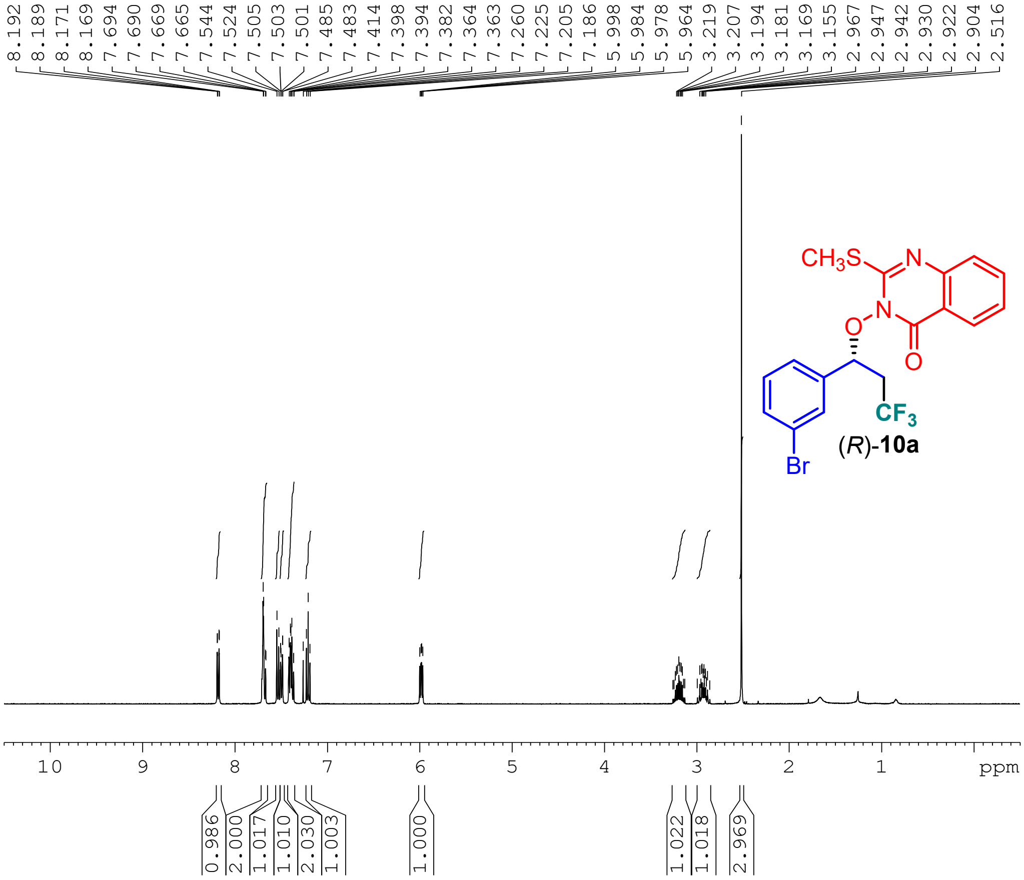

(*R*)-**10a**

NAME	CIL-1-212
EXPNO	3
PROCNO	1
Date_	20210518
Time	20.20
INSTRUM	spect
PROBHD	5 mm DUL 13C-1
PULPROG	zg30
TD	32768
SOLVENT	CDCl3
NS	8
DS	0
SWH	6410.256 Hz
FIDRES	0.195625 Hz
AQ	2.5559540 sec
RG	4
DW	78.000 usec
DE	6.00 usec
TE	300.0 K
D1	2.00000000 sec
TD0	1

======== CHANNEL f1 ========

NUC1	1H
P1	10.00 usec
PL1	-2.40 dB
SFO1	400.1528010 MHz
SI	16384
SF	400.1500092 MHz
WDW	EM
SSB	0
LB	0.00 Hz
GB	0
PC	1.00

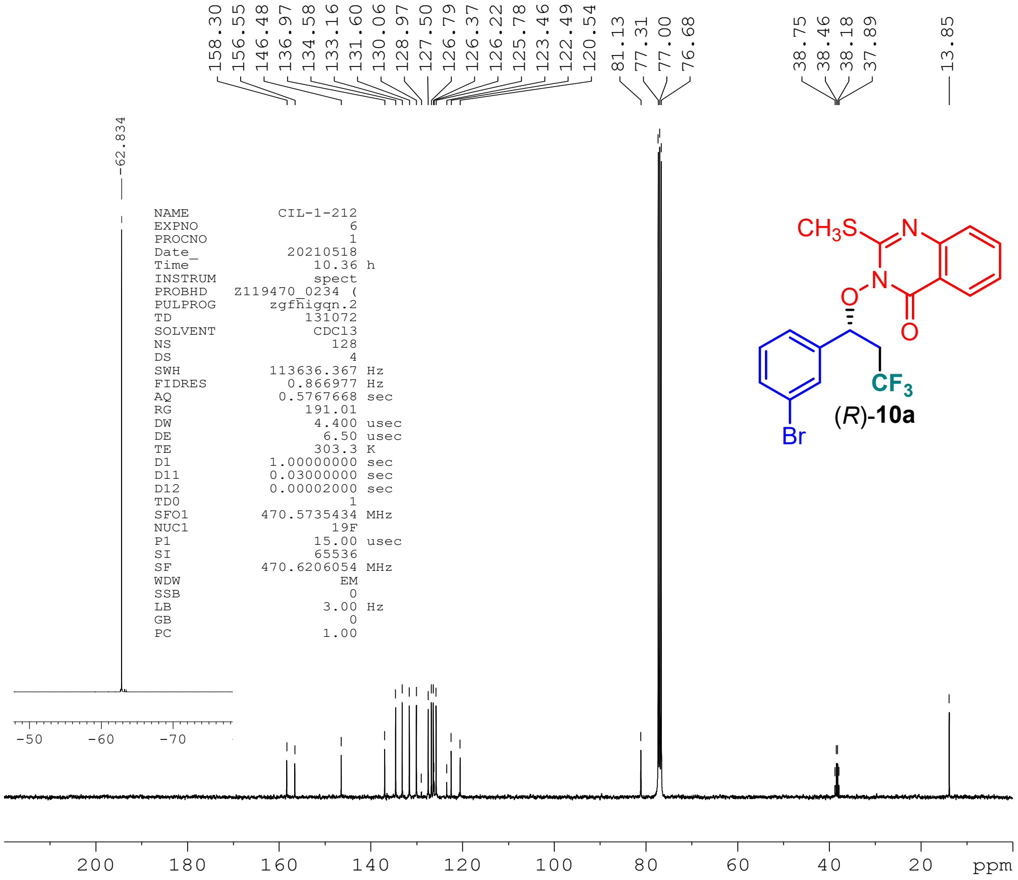

(*R*)-**10a**

NAME	CIL-1-212
EXPNO	4
PROCNO	1
Date_	20210518
Time	20.21
INSTRUM	spect
PROBHD	5 mm DUL 13C-1
PULPROG	zgpg30
TD	65536
SOLVENT	CDCl3
NS	11000
DS	0
SWH	22727.273 Hz
FIDRES	0.346791 Hz
AQ	1.4418420 sec
RG	57
DW	22.000 usec
DE	6.00 usec
TE	300.0 K
D1	2.00000000 sec
d11	0.03000000 sec
DELTA	1.89999998 sec
TD0	1

======== CHANNEL f1 ========

NUC1	13C
P1	9.70 usec
PL1	-0.50 dB
SFO1	100.6288660 MHz

======== CHANNEL f2 ========

CPDPRG2	waltz16
NUC2	1H
PCPD2	90.00 usec
PL2	-2.40 dB
PL12	15.10 dB
PL13	18.10 dB
SFO2	400.1516010 MHz
SI	32768
SF	100.6178006 MHz
WDW	EM
SSB	0
LB	3.00 Hz
GB	0
PC	1.00

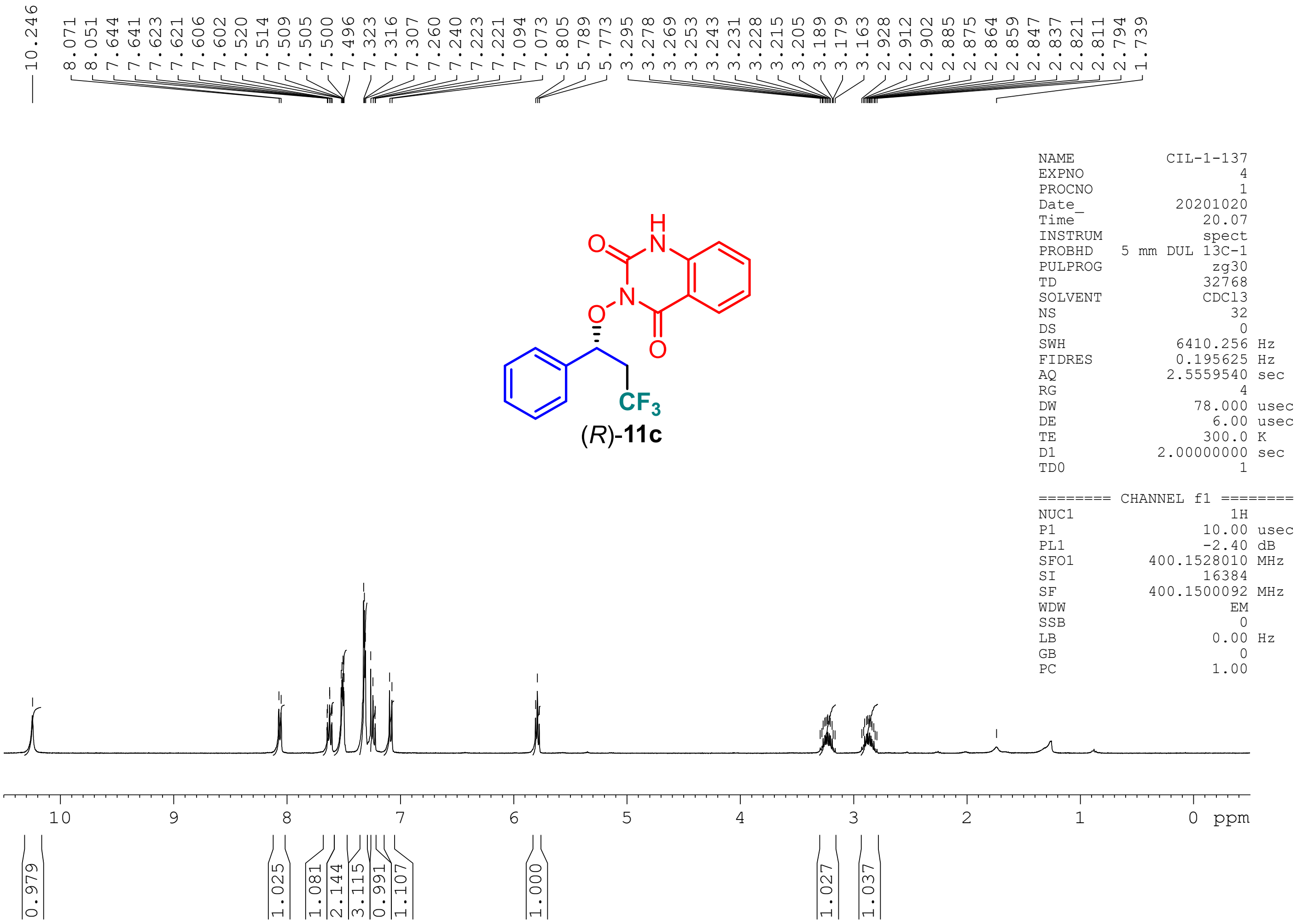

10.246
8.071 8.051 7.644 7.641 7.623 7.621 7.606 7.602 7.520 7.514 7.509 7.505 7.500 7.496 7.323 7.316 7.307 7.260 7.240 7.223 7.221 7.094 7.073 5.805 5.789 5.773 3.295 3.278 3.269 3.253 3.243 3.231 3.228 3.215 3.205 3.189 3.179 3.163 2.928 2.912 2.902 2.885 2.875 2.864 2.859 2.847 2.837 2.821 2.811 2.794 1.739
(R)-11c
NAME CIL-1-137
EXPNO 4
PROCNO 1
Date_ 20201020
Time 20.07
INSTRUM spect
PROBHD 5 mm DUL 13C-1
PULPROG zg30
TD 32768
SOLVENT CDCl3
NS 32
DS 0
SWH 6410.256 Hz
FIDRES 0.195625 Hz
AQ 2.5559540 sec
RG 4
DW 78.000 usec
DE 6.00 usec
TE 300.0 K
D1 2.00000000 sec
TD0 1
======== CHANNEL f1 ========
NUC1 1H
P1 10.00 usec
PL1 -2.40 dB
SFO1 400.1528010 MHz
SI 16384
SF 400.1500092 MHz
WDW EM
SSB 0
LB 0.00 Hz
GB 0
PC 1.00
10 9 8 7 6 5 4 3 2 1 0 ppm
0.979 1.025 1.081 2.144 3.115 0.991 1.107 1.000 1.027 1.037

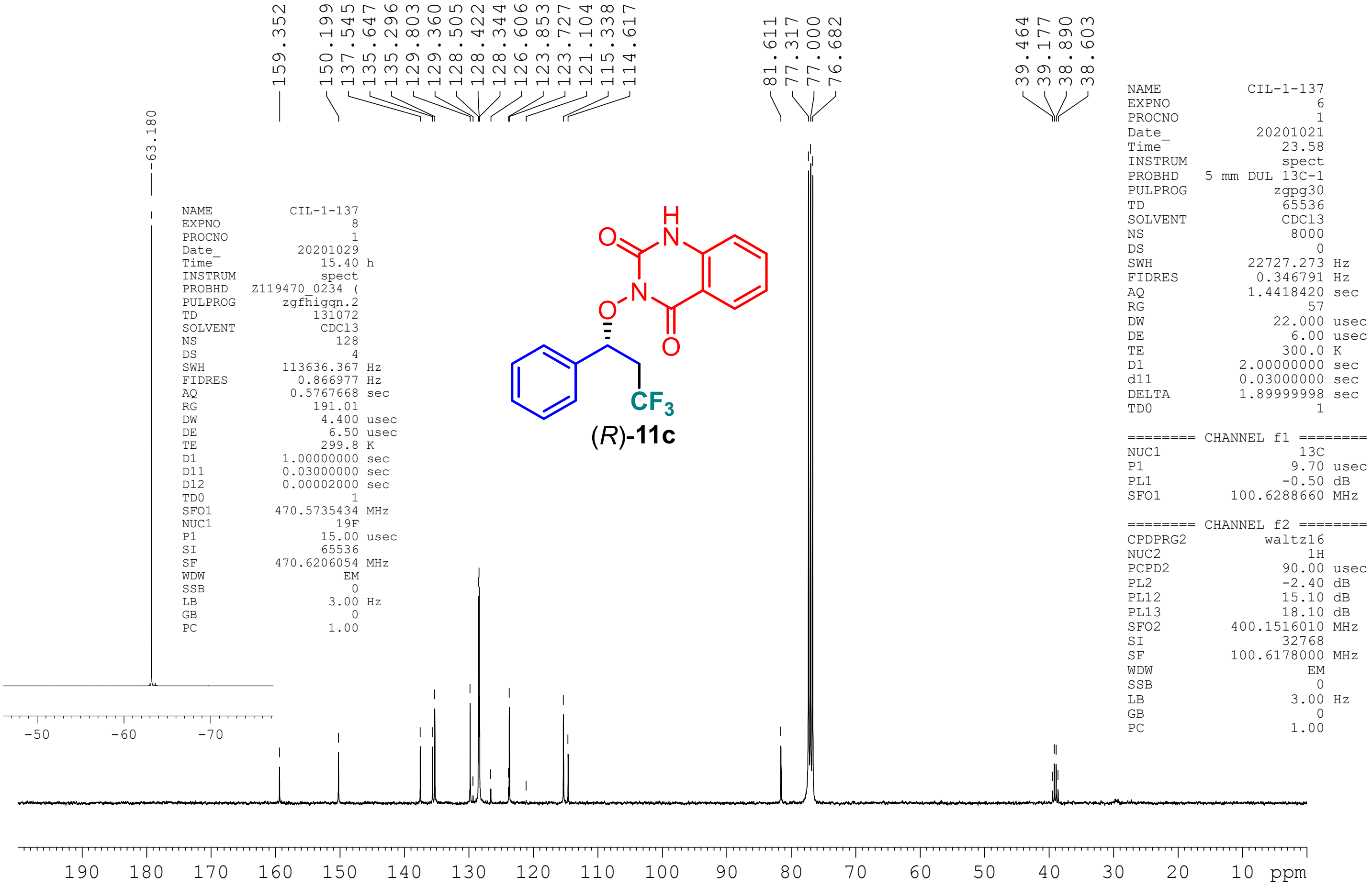

159.352 150.199 137.545 135.647 135.296 129.803 129.360 128.505 128.422 128.344 126.606 123.853 123.727 121.104 115.338 114.617 81.611 77.317 77.000 76.682 39.464 39.177 38.890 38.603
-63.180
NAME CIL-1-137
EXPNO 8
PROCNO 1
Date_ 20201029
Time 15.40 h
INSTRUM spect
PROBHD Z119470_0234 (
PULPROG zgfhigqn.2
TD 131072
SOLVENT CDCl3
NS 128
DS 4
SWH 113636.367 Hz
FIDRES 0.866977 Hz
AQ 0.5767668 sec
RG 191.01
DW 4.400 usec
DE 6.50 usec
TE 299.8 K
D1 1.00000000 sec
D11 0.03000000 sec
D12 0.00002000 sec
TD0 1
SFO1 470.5735434 MHz
NUC1 19F
P1 15.00 usec
SI 65536
SF 470.6206054 MHz
WDW EM
SSB 0
LB 3.00 Hz
GB 0
PC 1.00
-50 -60 -70
(R)-11c
NAME CIL-1-137
EXPNO 6
PROCNO 1
Date_ 20201021
Time 23.58
INSTRUM spect
PROBHD 5 mm DUL 13C-1
PULPROG zgpg30
TD 65536
SOLVENT CDCl3
NS 8000
DS 0
SWH 22727.273 Hz
FIDRES 0.346791 Hz
AQ 1.4418420 sec
RG 57
DW 22.000 usec
DE 6.00 usec
TE 300.0 K
D1 2.00000000 sec
d11 0.03000000 sec
DELTA 1.89999998 sec
TD0 1
======== CHANNEL f1 ========
NUC1 13C
P1 9.70 usec
PL1 -0.50 dB
SFO1 100.6288660 MHz
======== CHANNEL f2 ========
CPDPRG2 waltz16
NUC2 1H
PCPD2 90.00 usec
PL2 -2.40 dB
PL12 15.10 dB
PL13 18.10 dB
SFO2 400.1516010 MHz
SI 32768
SF 100.6178000 MHz
WDW EM
SSB 0
LB 3.00 Hz
GB 0
PC 1.00
190 180 170 160 150 140 130 120 110 100 90 80 70 60 50 40 30 20 10 ppm

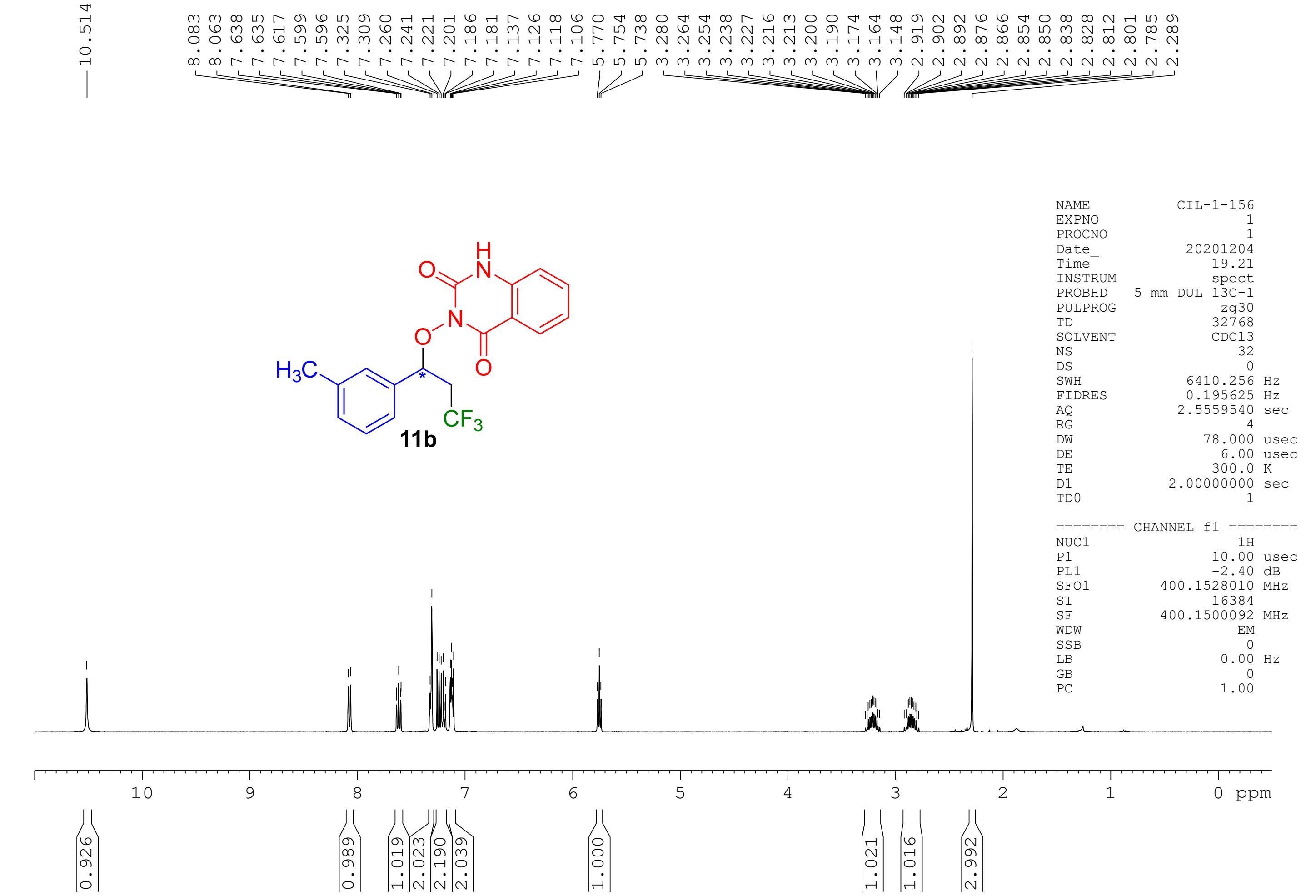
10.514
8.083 8.063 7.638 7.635 7.617 7.599 7.596 7.325 7.309 7.260 7.241 7.221 7.201 7.186 7.181 7.137 7.126 7.118 7.106 5.770 5.754 5.738 3.280 3.264 3.254 3.238 3.227 3.216 3.213 3.200 3.190 3.174 3.164 3.148 2.919 2.902 2.892 2.876 2.866 2.854 2.850 2.838 2.828 2.812 2.801 2.785 2.289
H3C
CF3
11b
NAME CIL-1-156
EXPNO 1
PROCNO 1
Date_ 20201204
Time 19.21
INSTRUM spect
PROBHD 5 mm DUL 13C-1
PULPROG zg30
TD 32768
SOLVENT CDCl3
NS 32
DS 0
SWH 6410.256 Hz
FIDRES 0.195625 Hz
AQ 2.5559540 sec
RG 4
DW 78.000 usec
DE 6.00 usec
TE 300.0 K
D1 2.00000000 sec
TD0 1
======== CHANNEL f1 ========
NUC1 1H
P1 10.00 usec
PL1 -2.40 dB
SFO1 400.1528010 MHz
SI 16384
SF 400.1500092 MHz
WDW EM
SSB 0
LB 0.00 Hz
GB 0
PC 1.00
0.926 0.989 1.019 2.023 2.190 2.039 1.000 1.021 1.016 2.992
ppm

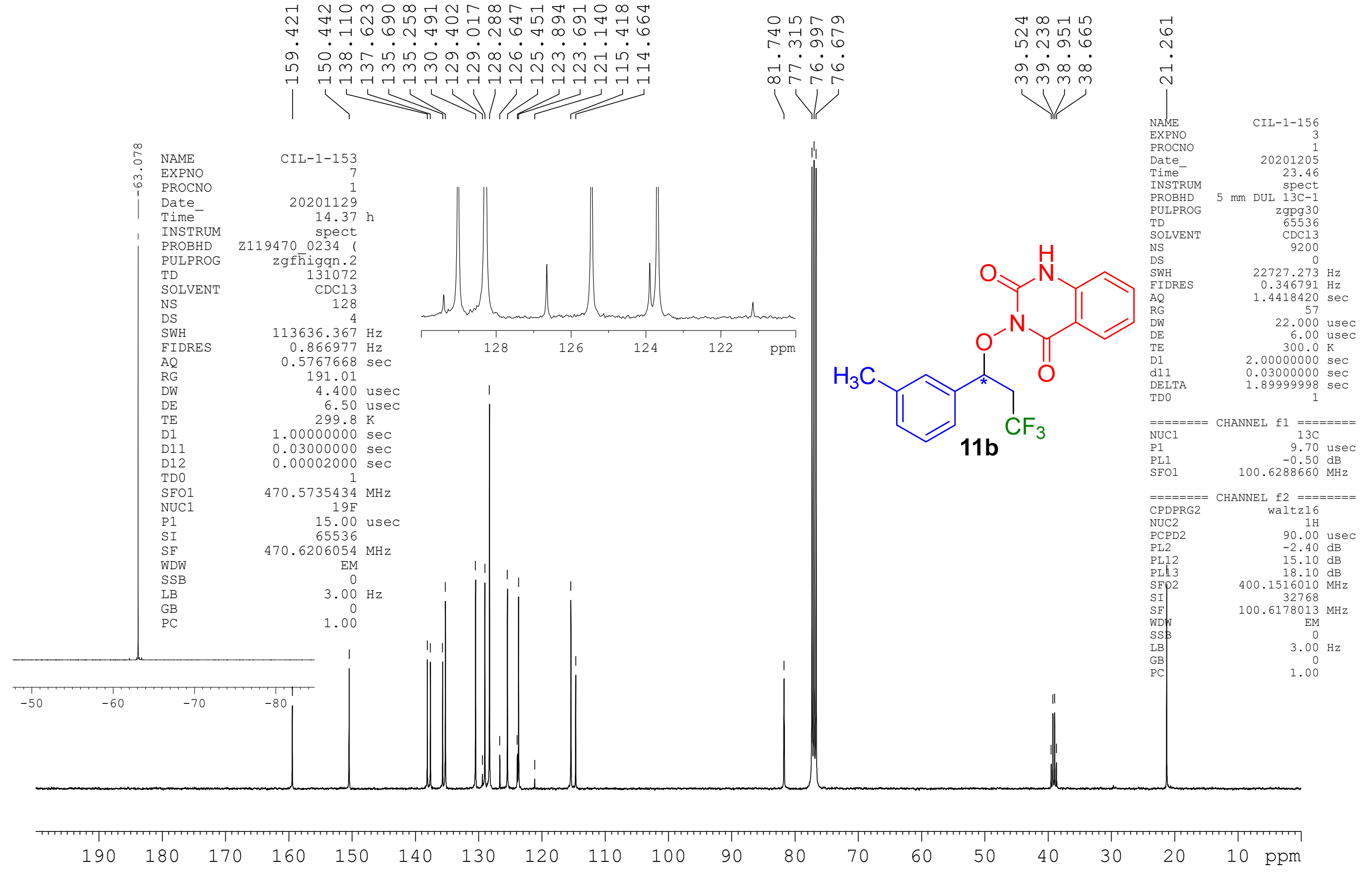
159.421 150.442 138.110 137.623 135.690 135.258 130.491 129.402 129.017 128.288 126.647 125.451 123.894 123.691 121.140 115.418 114.664 81.740 77.315 76.997 76.679 39.524 39.238 38.951 38.665 21.261
-63.078
NAME CIL-1-153
EXPNO 7
PROCNO 1
Date_ 20201129
Time 14.37 h
INSTRUM spect
PROBHD Z119470_0234 (
PULPROG zgfhigqn.2
TD 131072
SOLVENT CDCl3
NS 128
DS 4
SWH 113636.367 Hz
FIDRES 0.866977 Hz
AQ 0.5767668 sec
RG 191.01
DW 4.400 usec
DE 6.50 usec
TE 299.8 K
D1 1.00000000 sec
D11 0.03000000 sec
D12 0.00002000 sec
TD0 1
SFO1 470.5735434 MHz
NUC1 19F
P1 15.00 usec
SI 65536
SF 470.6206054 MHz
WDW EM
SSB 0
LB 3.00 Hz
GB 0
PC 1.00
H3C
CF3
11b
NAME CIL-1-156
EXPNO 3
PROCNO 1
Date_ 20201205
Time 23.46
INSTRUM spect
PROBHD 5 mm DUL 13C-1
PULPROG zgpg30
TD 65536
SOLVENT CDCl3
NS 9200
DS 0
SWH 22727.273 Hz
FIDRES 0.346791 Hz
AQ 1.4418420 sec
RG 57
DW 22.000 usec
DE 6.00 usec
TE 300.0 K
D1 2.00000000 sec
d11 0.03000000 sec
DELTA 1.89999998 sec
TD0 1
======== CHANNEL f1 ========
NUC1 13C
P1 9.70 usec
PL1 -0.50 dB
SFO1 100.6288660 MHz
======== CHANNEL f2 ========
CPDPRG2 waltz16
NUC2 1H
PCPD2 90.00 usec
PL2 -2.40 dB
PL12 15.10 dB
PL13 18.10 dB
SFO2 400.1516010 MHz
SI 32768
SF 100.6178013 MHz
WDW EM
SSB 0
LB 3.00 Hz
GB 0
PC 1.00
ppm

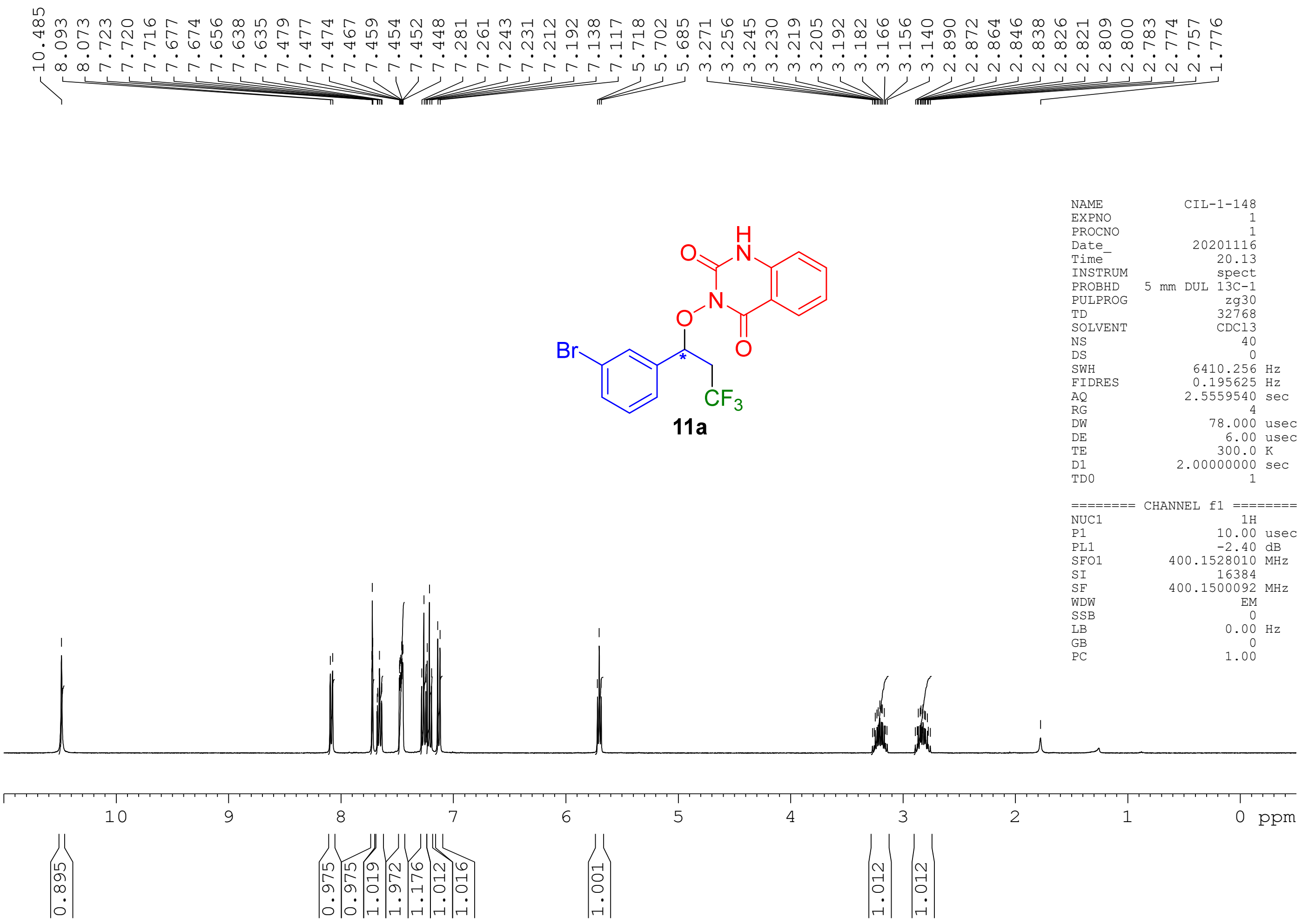
10.485
8.093
8.073
7.723
7.720
7.716
7.677
7.674
7.656
7.638
7.635
7.479
7.477
7.474
7.467
7.459
7.454
7.452
7.448
7.281
7.261
7.243
7.231
7.212
7.192
7.138
7.117
5.718
5.702
5.685
3.271
3.256
3.245
3.230
3.219
3.205
3.192
3.182
3.166
3.156
3.140
2.890
2.872
2.864
2.846
2.838
2.826
2.821
2.809
2.800
2.783
2.774
2.757
1.776
Br
O
H
N
N
O
O
*
CF3
11a
NAME CIL-1-148
EXPNO 1
PROCNO 1
Date_ 20201116
Time 20.13
INSTRUM spect
PROBHD 5 mm DUL 13C-1
PULPROG zg30
TD 32768
SOLVENT CDCl3
NS 40
DS 0
SWH 6410.256 Hz
FIDRES 0.195625 Hz
AQ 2.5559540 sec
RG 4
DW 78.000 usec
DE 6.00 usec
TE 300.0 K
D1 2.00000000 sec
TD0 1
======== CHANNEL f1 ========
NUC1 1H
P1 10.00 usec
PL1 -2.40 dB
SFO1 400.1528010 MHz
SI 16384
SF 400.1500092 MHz
WDW EM
SSB 0
LB 0.00 Hz
GB 0
PC 1.00
10
9
8
7
6
5
4
3
2
1
0 ppm
0.895
0.975
0.975
1.019
1.972
1.176
1.012
1.016
1.001
1.012
1.012

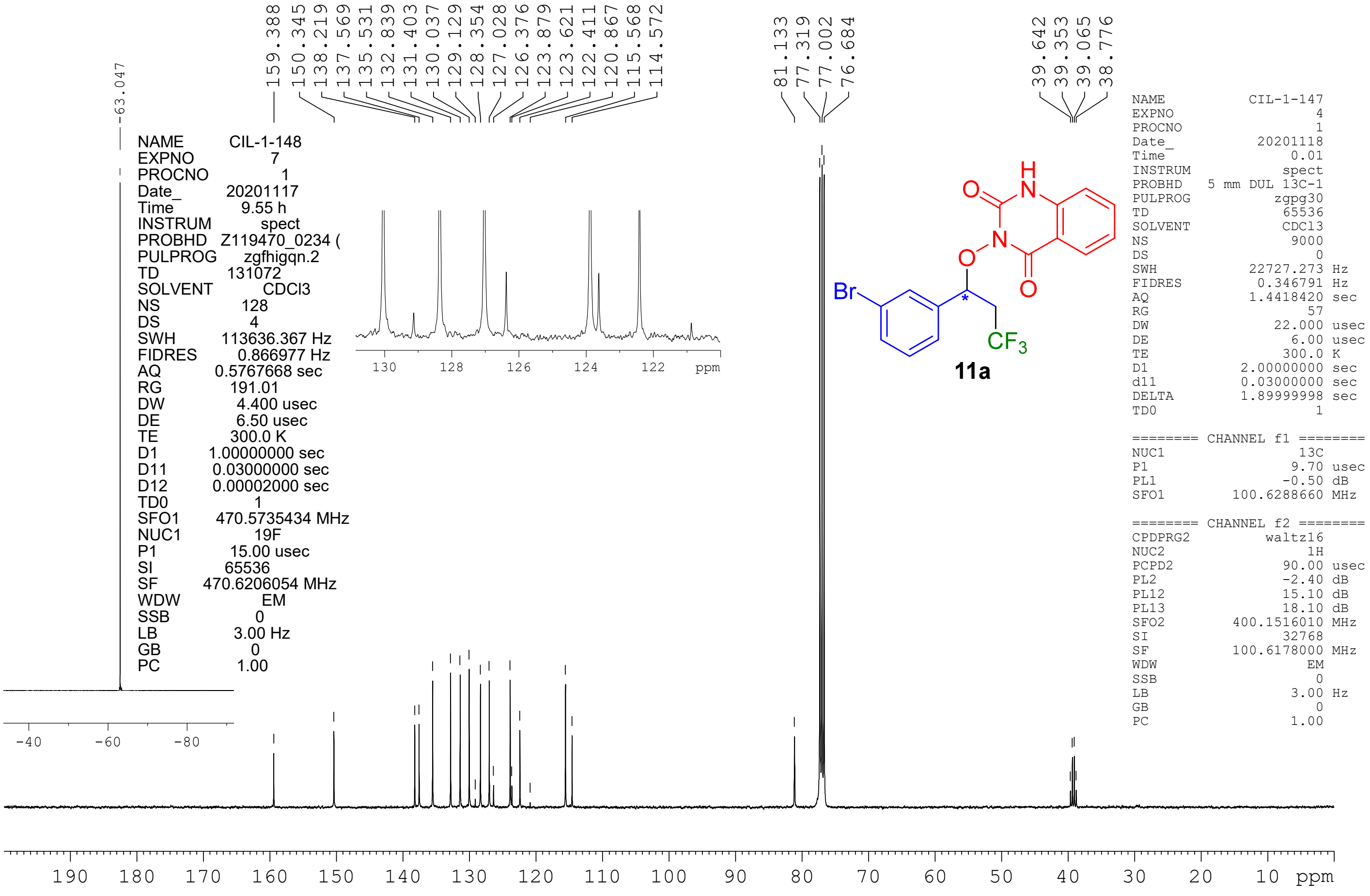
159.388
150.345
138.219
137.569
135.531
132.839
131.403
130.037
129.129
128.354
127.028
126.376
123.879
123.621
122.411
120.867
115.568
114.572
81.133
77.319
77.002
76.684
39.642
39.353
39.065
38.776
-63.047
NAME CIL-1-148
EXPNO 7
PROCNO 1
Date_ 20201117
Time 9.55 h
INSTRUM spect
PROBHD Z119470_0234 (
PULPROG zgfhigqn.2
TD 131072
SOLVENT CDCl3
NS 128
DS 4
SWH 113636.367 Hz
FIDRES 0.866977 Hz
AQ 0.5767668 sec
RG 191.01
DW 4.400 usec
DE 6.50 usec
TE 300.0 K
D1 1.00000000 sec
D11 0.03000000 sec
D12 0.00002000 sec
TD0 1
SFO1 470.5735434 MHz
NUC1 19F
P1 15.00 usec
SI 65536
SF 470.6206054 MHz
WDW EM
SSB 0
LB 3.00 Hz
GB 0
PC 1.00
-40
-60
-80
130
128
126
124
122
ppm
Br
O
H
N
N
O
O
*
CF3
11a
NAME CIL-1-147
EXPNO 4
PROCNO 1
Date_ 20201118
Time 0.01
INSTRUM spect
PROBHD 5 mm DUL 13C-1
PULPROG zgpg30
TD 65536
SOLVENT CDCl3
NS 9000
DS 0
SWH 22727.273 Hz
FIDRES 0.346791 Hz
AQ 1.4418420 sec
RG 57
DW 22.000 usec
DE 6.00 usec
TE 300.0 K
D1 2.00000000 sec
d11 0.03000000 sec
DELTA 1.89999998 sec
TD0 1
======== CHANNEL f1 ========
NUC1 13C
P1 9.70 usec
PL1 -0.50 dB
SFO1 100.6288660 MHz
======== CHANNEL f2 ========
CPDPRG2 waltz16
NUC2 1H
PCPD2 90.00 usec
PL2 -2.40 dB
PL12 15.10 dB
PL13 18.10 dB
SFO2 400.1516010 MHz
SI 32768
SF 100.6178000 MHz
WDW EM
SSB 0
LB 3.00 Hz
GB 0
PC 1.00
190
180
170
160
150
140
130
120
110
100
90
80
70
60
50
40
30
20
10 ppm

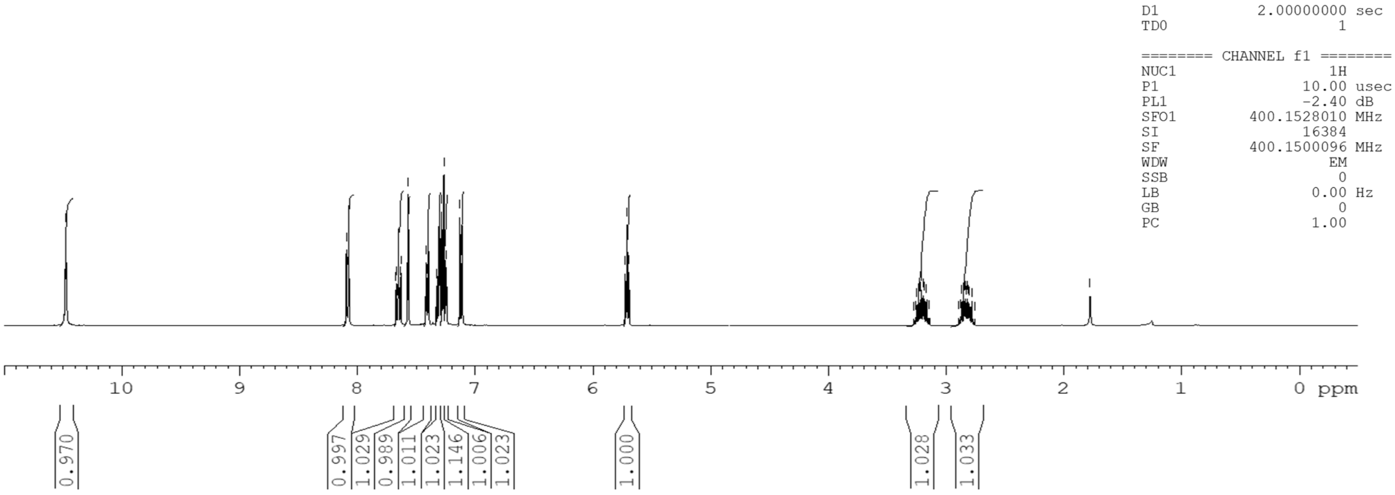

NAME CIL-1-153
EXPNO 2
PROCNO 1
Date_ 20201123
Time 21.29
INSTRUM spect
PROBHD 5 mm DUL 13C-1
PULPROG zg30
TD 32768
SOLVENT CDCl3
NS 100
DS 0
SWH 6410.256 Hz
FIDRES 0.195625 Hz
AQ 2.5559540 sec
RG 4
DW 78.000 usec
DE 6.00 usec
TE 300.0 K
D1 2.00000000 sec
TD0 1

======== CHANNEL f1 ========
NUC1 1H
P1 10.00 usec
PL1 -2.40 dB
SFO1 400.1528010 MHz
SI 16384
SF 400.1500096 MHz
WDW EM
SSB 0
LB 0.00 Hz
GB 0
PC 1.00

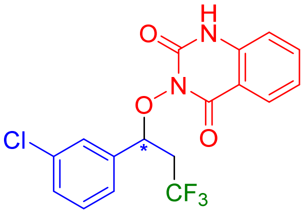

11a'

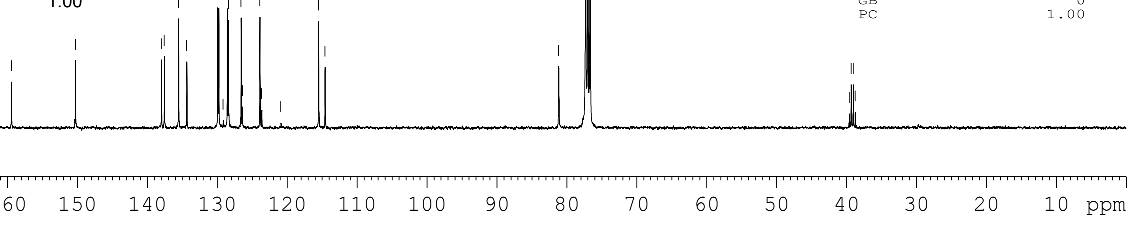

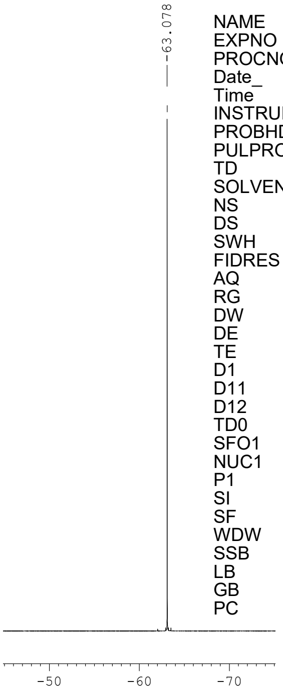

NAME CIL-1-153
EXPNO 7
PROCNO 1
Date_ 20201125
Time 14.37 h
INSTRUM spect
PROBHD Z119470_0234 (
PULPROG zgfhigqn.2
TD 131072
SOLVENT CDCl3
NS 128
DS 4
SWH 113636.367 Hz
FIDRES 0.866977 Hz
AQ 0.5767668 sec
RG 191.01
DW 4.400 usec
DE 6.50 usec
TE 299.8 K
D1 1.00000000 sec
D11 0.03000000 sec
D12 0.00002000 sec
TD0 1
SFO1 470.5735434 MHz
NUC1 19F
P1 15.00 usec
SI 65536
SF 470.6206054 MHz
WDW EM
SSB 0
LB 3.00 Hz
GB 0
PC 1.00

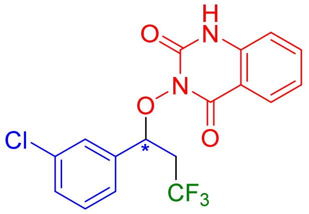

11a'

NAME CIL-1-153
EXPNO 4
PROCNO 1
Date_ 20201124
Time 0.05
INSTRUM spect
PROBHD 5 mm DUL 13C-1
PULPROG zgpg30
TD 65536
SOLVENT CDCl3
NS 8000
DS 0
SWH 22727.273 Hz
FIDRES 0.346791 Hz
AQ 1.4418420 sec
RG 57
DW 22.000 usec
DE 6.00 usec
TE 300.0 K
D1 2.00000000 sec
d11 0.03000000 sec
DELTA 1.89999998 sec
TD0 1

======== CHANNEL f1 ========
NUC1 13C
P1 9.70 usec
PL1 -0.50 dB
SFO1 100.6288660 MHz

======== CHANNEL f2 ========
CPDPRG2 waltz16
NUC2 1H
PCPD2 90.00 usec
PL2 -2.40 dB
PL12 15.10 dB
PL13 18.10 dB
SFO2 400.1516010 MHz
SI 32768
SF 100.6177993 MHz
WDW EM
SSB 0
LB 3.00 Hz
GB 0
PC 1.00

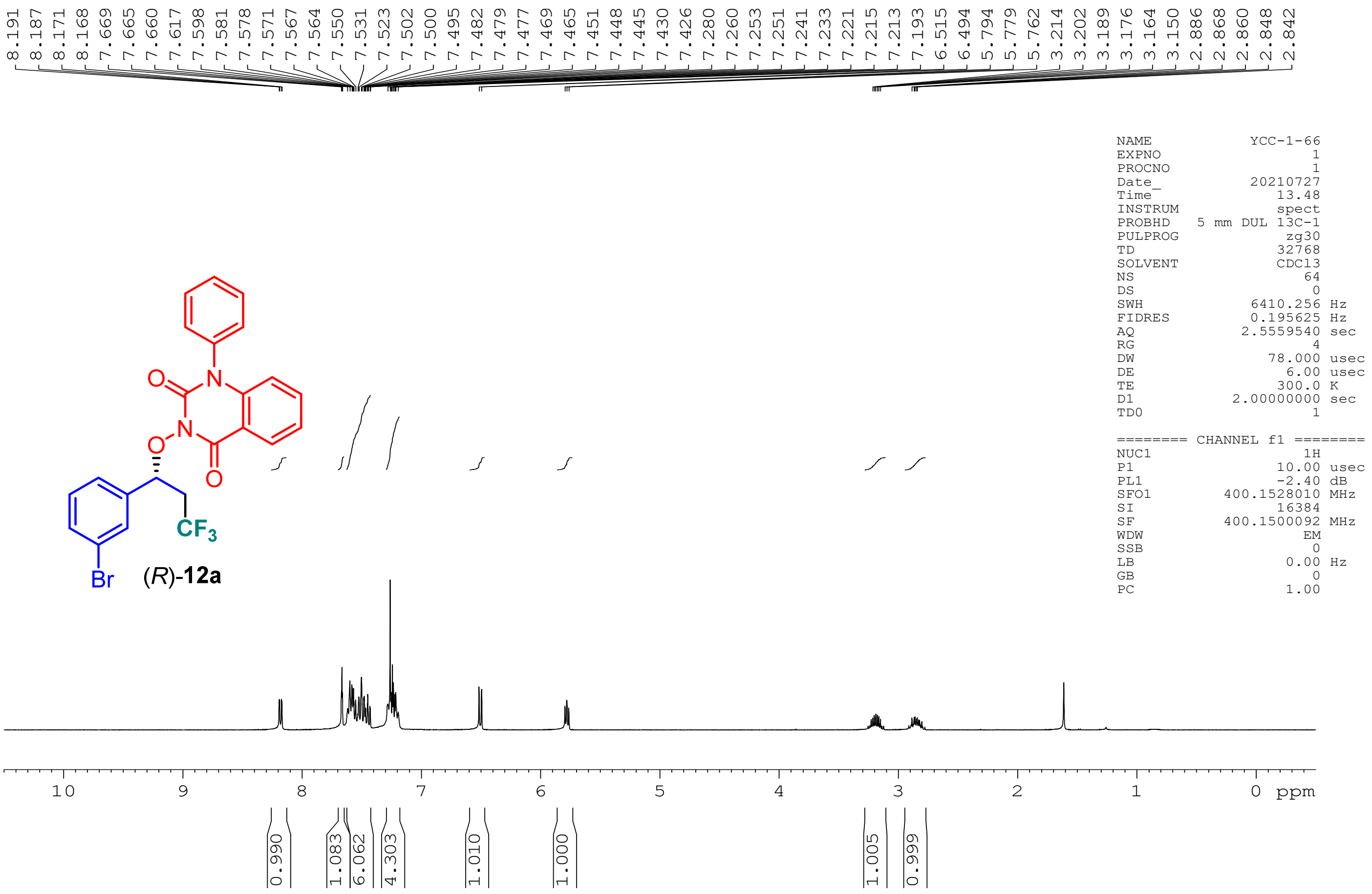
8.191 8.187 8.171 8.168 7.669 7.665 7.660 7.617 7.598 7.581 7.578 7.571 7.567 7.564 7.550 7.531 7.523 7.502 7.500 7.495 7.482 7.479 7.477 7.469 7.465 7.451 7.448 7.445 7.430 7.426 7.280 7.260 7.253 7.251 7.241 7.233 7.221 7.215 7.213 7.193 6.515 6.494 5.794 5.779 5.762 3.214 3.202 3.189 3.176 3.164 3.150 2.886 2.868 2.860 2.848 2.842
NAME YCC-1-66
EXPNO 1
PROCNO 1
Date_ 20210727
Time 13.48
INSTRUM spect
PROBHD 5 mm DUL 13C-1
PULPROG zg30
TD 32768
SOLVENT CDCl3
NS 64
DS 0
SWH 6410.256 Hz
FIDRES 0.195625 Hz
AQ 2.5559540 sec
RG 4
DW 78.000 usec
DE 6.00 usec
TE 300.0 K
D1 2.00000000 sec
TD0 1
======== CHANNEL f1 ========
NUC1 1H
P1 10.00 usec
PL1 -2.40 dB
SFO1 400.1528010 MHz
SI 16384
SF 400.1500092 MHz
WDW EM
SSB 0
LB 0.00 Hz
GB 0
PC 1.00
O N O N O O CF3 Br
(R)-12a
10 9 8 7 6 5 4 3 2 1 0 ppm
0.990 1.083 6.062 4.303 1.010 1.000 1.005 0.999

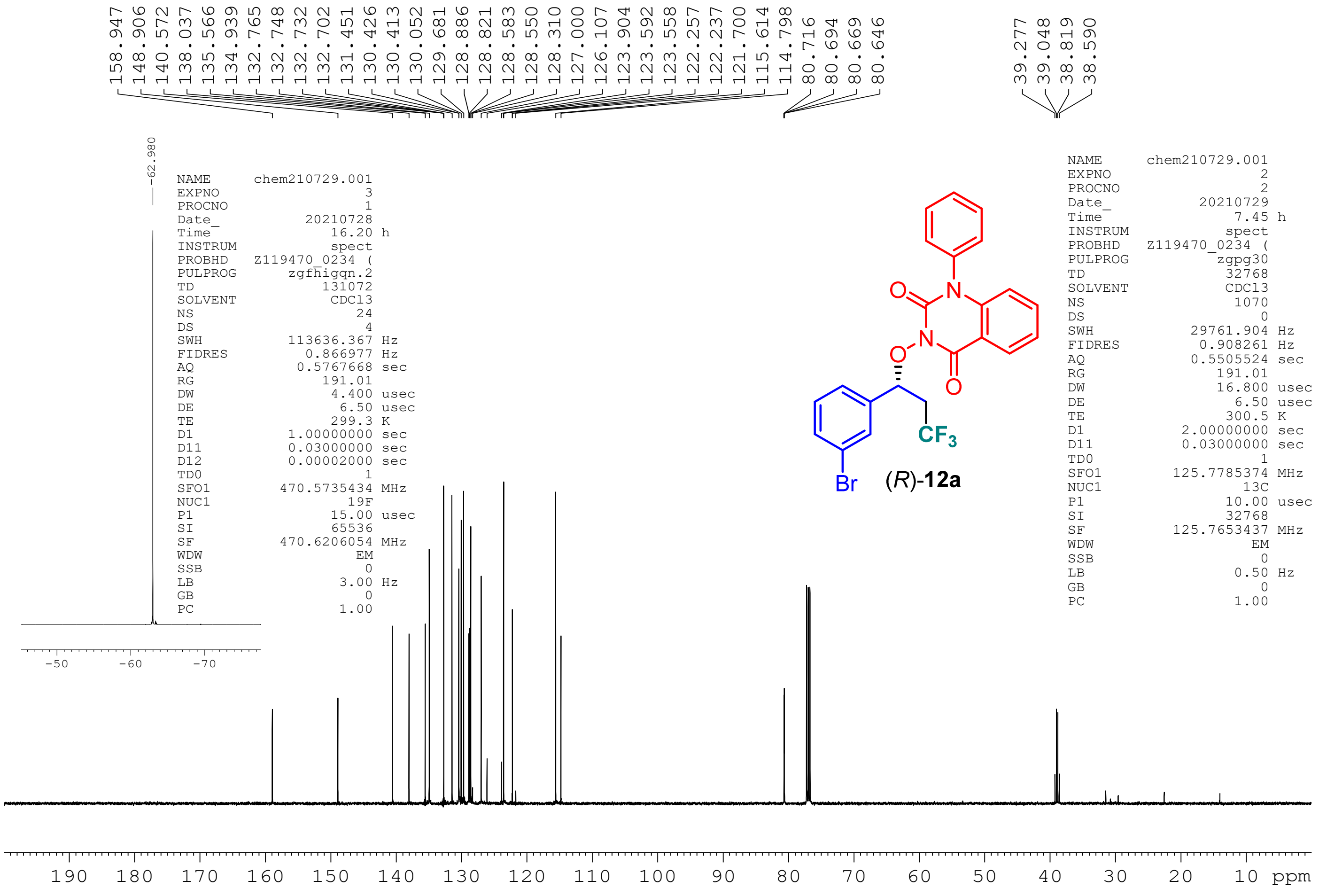
158.947 148.906 140.572 138.037 135.566 134.939 132.765 132.748 132.732 132.702 131.451 130.426 130.413 130.052 129.681 128.886 128.821 128.583 128.550 128.310 127.000 126.107 123.904 123.592 123.558 122.257 122.237 121.700 115.614 114.798 80.716 80.694 80.669 80.646
39.277 39.048 38.819 38.590
-62.980
NAME chem210729.001
EXPNO 3
PROCNO 1
Date_ 20210728
Time 16.20 h
INSTRUM spect
PROBHD Z119470_0234 (
PULPROG zgfhigqn.2
TD 131072
SOLVENT CDCl3
NS 24
DS 4
SWH 113636.367 Hz
FIDRES 0.866977 Hz
AQ 0.5767668 sec
RG 191.01
DW 4.400 usec
DE 6.50 usec
TE 299.3 K
D1 1.00000000 sec
D11 0.03000000 sec
D12 0.00002000 sec
TD0 1
SFO1 470.5735434 MHz
NUC1 19F
P1 15.00 usec
SI 65536
SF 470.6206054 MHz
WDW EM
SSB 0
LB 3.00 Hz
GB 0
PC 1.00
-50 -60 -70
NAME chem210729.001
EXPNO 2
PROCNO 2
Date_ 20210729
Time 7.45 h
INSTRUM spect
PROBHD Z119470_0234 (
PULPROG zgpg30
TD 32768
SOLVENT CDCl3
NS 1070
DS 0
SWH 29761.904 Hz
FIDRES 0.908261 Hz
AQ 0.5505524 sec
RG 191.01
DW 16.800 usec
DE 6.50 usec
TE 300.5 K
D1 2.00000000 sec
D11 0.03000000 sec
TD0 1
SFO1 125.7785374 MHz
NUC1 13C
P1 10.00 usec
SI 32768
SF 125.7653437 MHz
WDW EM
SSB 0
LB 0.50 Hz
GB 0
PC 1.00
O N O N O O CF3 Br
(R)-12a
190 180 170 160 150 140 130 120 110 100 90 80 70 60 50 40 30 20 10 ppm

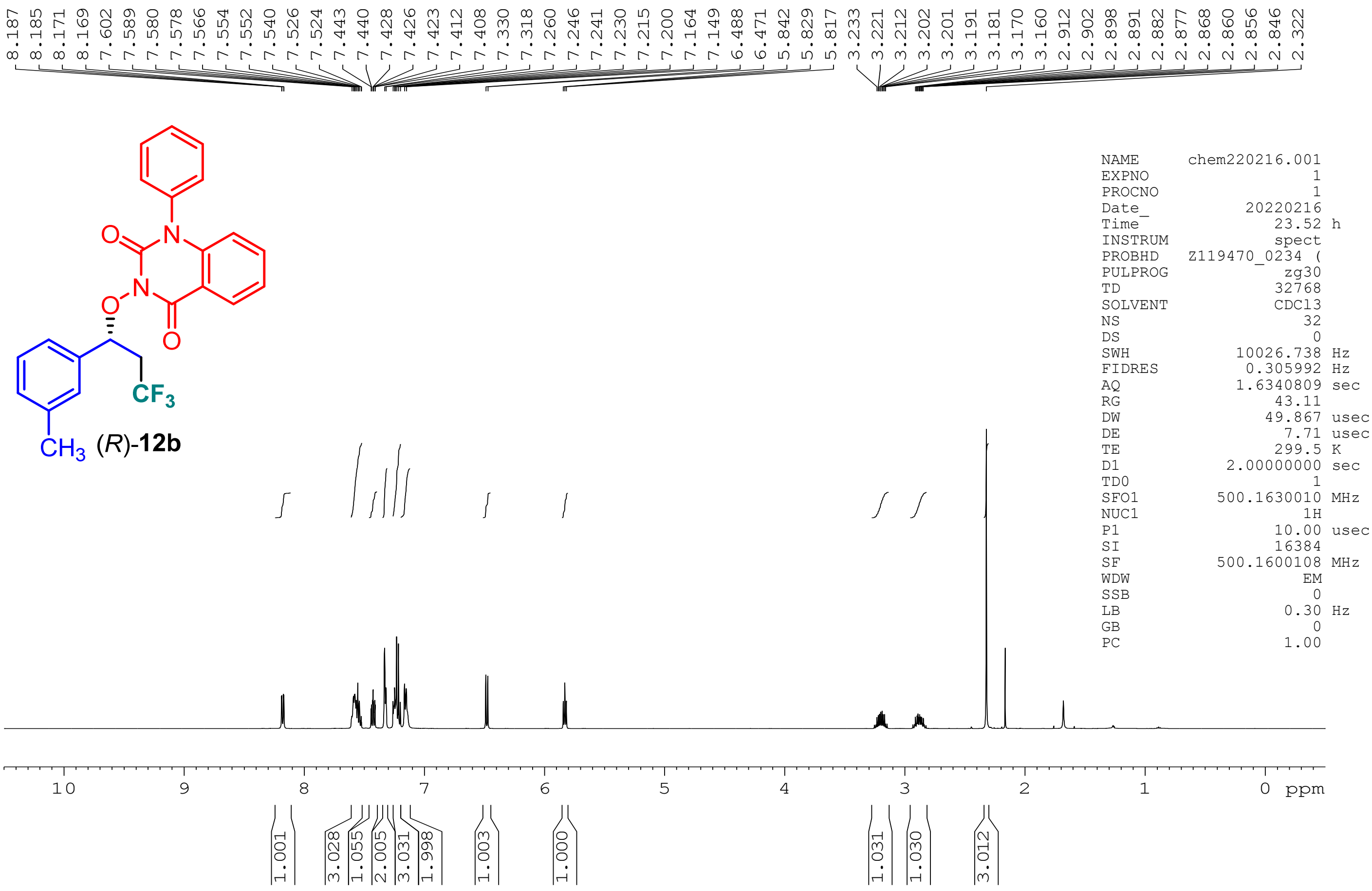
8.187
8.185
8.171
8.169
7.602
7.589
7.580
7.578
7.566
7.554
7.552
7.540
7.526
7.524
7.443
7.440
7.428
7.426
7.423
7.412
7.408
7.330
7.318
7.260
7.246
7.241
7.230
7.215
7.200
7.164
7.149
6.488
6.471
5.842
5.829
5.817
3.233
3.221
3.212
3.202
3.201
3.191
3.181
3.170
3.160
2.912
2.902
2.898
2.891
2.882
2.877
2.868
2.860
2.856
2.846
2.322
O
N
N
O
O
CF3
CH3 (R)-12b
NAME chem220216.001
EXPNO 1
PROCNO 1
Date_ 20220216
Time 23.52 h
INSTRUM spect
PROBHD Z119470_0234 (
PULPROG zg30
TD 32768
SOLVENT CDCl3
NS 32
DS 0
SWH 10026.738 Hz
FIDRES 0.305992 Hz
AQ 1.6340809 sec
RG 43.11
DW 49.867 usec
DE 7.71 usec
TE 299.5 K
D1 2.00000000 sec
TD0 1
SFO1 500.1630010 MHz
NUC1 1H
P1 10.00 usec
SI 16384
SF 500.1600108 MHz
WDW EM
SSB 0
LB 0.30 Hz
GB 0
PC 1.00
10 9 8 7 6 5 4 3 2 1 0 ppm
1.001
3.028
1.055
2.005
3.031
1.998
1.003
1.000
1.031
1.030
3.012

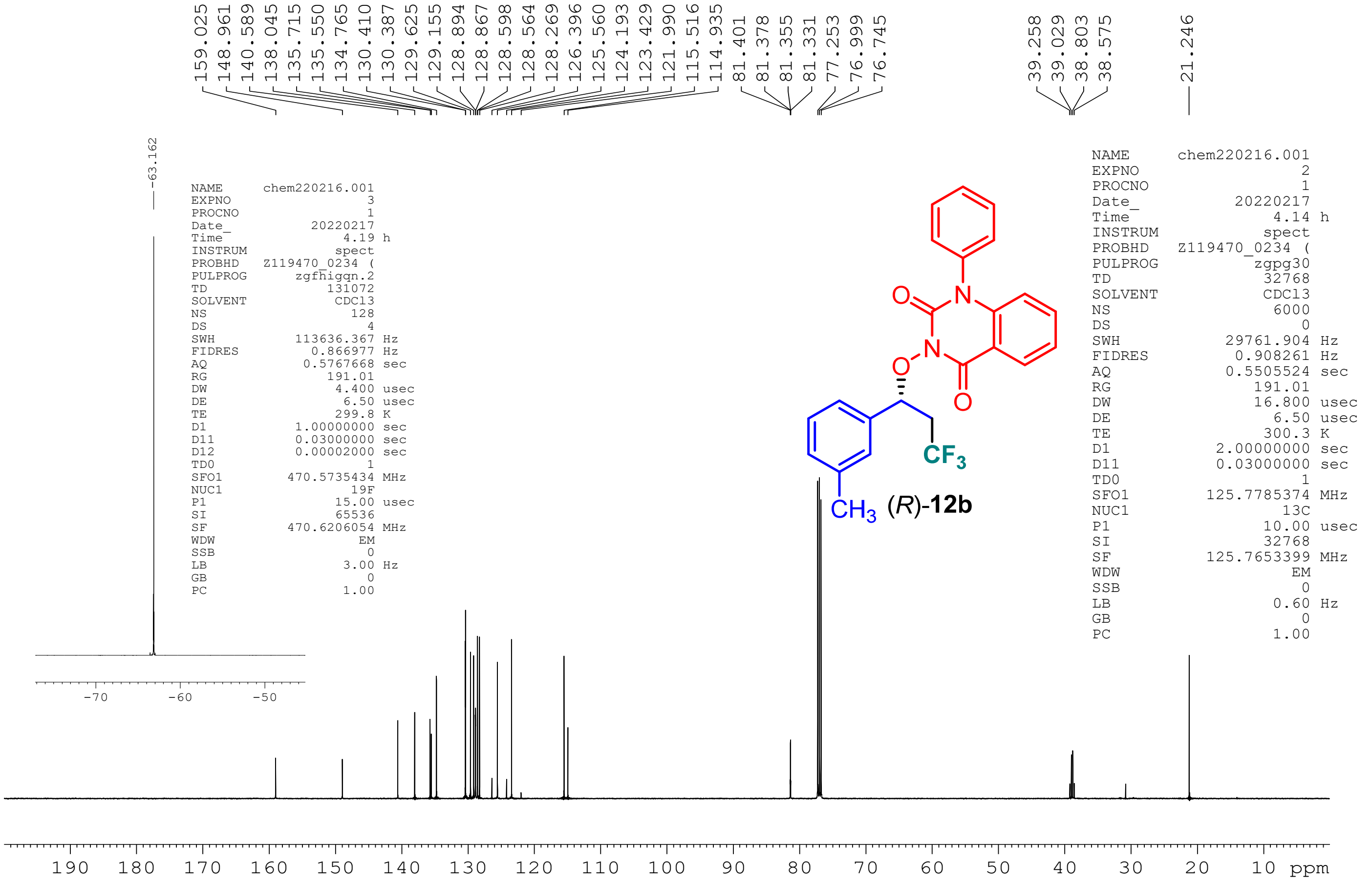
159.025
148.961
140.589
138.045
135.715
135.550
134.765
130.410
130.387
129.625
129.155
128.894
128.867
128.598
128.564
128.269
126.396
125.560
124.193
123.429
121.990
115.516
114.935
81.401
81.378
81.355
81.331
77.253
76.999
76.745
39.258
39.029
38.803
38.575
21.246
-63.162
NAME chem220216.001
EXPNO 3
PROCNO 1
Date_ 20220217
Time 4.19 h
INSTRUM spect
PROBHD Z119470_0234 (
PULPROG zgfhigqn.2
TD 131072
SOLVENT CDCl3
NS 128
DS 4
SWH 113636.367 Hz
FIDRES 0.866977 Hz
AQ 0.5767668 sec
RG 191.01
DW 4.400 usec
DE 6.50 usec
TE 299.8 K
D1 1.00000000 sec
D11 0.03000000 sec
D12 0.00002000 sec
TD0 1
SFO1 470.5735434 MHz
NUC1 19F
P1 15.00 usec
SI 65536
SF 470.6206054 MHz
WDW EM
SSB 0
LB 3.00 Hz
GB 0
PC 1.00
-70 -60 -50
O
N
N
O
O
CF3
CH3 (R)-12b
NAME chem220216.001
EXPNO 2
PROCNO 1
Date_ 20220217
Time 4.14 h
INSTRUM spect
PROBHD Z119470_0234 (
PULPROG zgpg30
TD 32768
SOLVENT CDCl3
NS 6000
DS 0
SWH 29761.904 Hz
FIDRES 0.908261 Hz
AQ 0.5505524 sec
RG 191.01
DW 16.800 usec
DE 6.50 usec
TE 300.3 K
D1 2.00000000 sec
D11 0.03000000 sec
TD0 1
SFO1 125.7785374 MHz
NUC1 13C
P1 10.00 usec
SI 32768
SF 125.7653399 MHz
WDW EM
SSB 0
LB 0.60 Hz
GB 0
PC 1.00
190 180 170 160 150 140 130 120 110 100 90 80 70 60 50 40 30 20 10 ppm

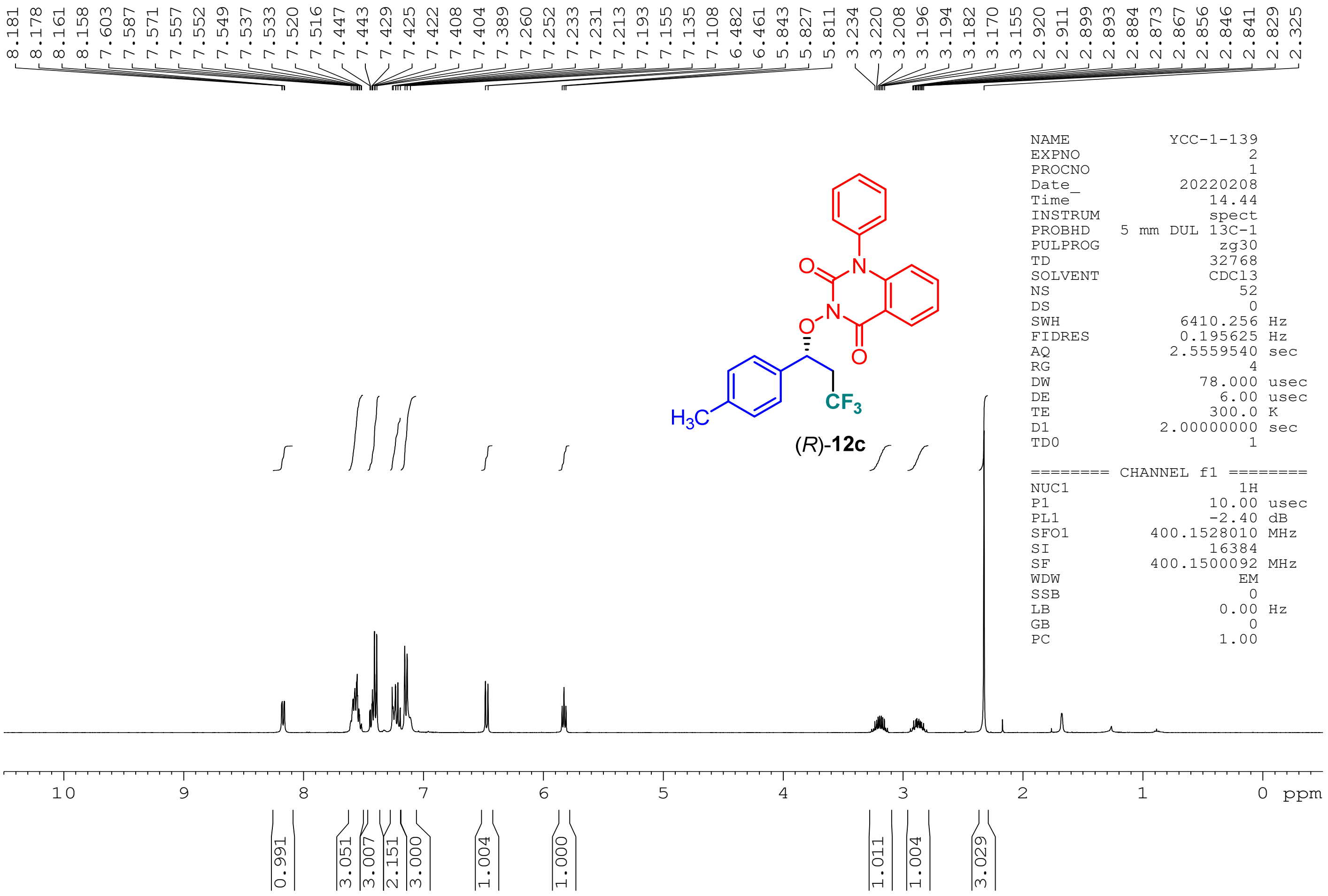
NAME YCC-1-139
EXPNO 2
PROCNO 1
Date_ 20220208
Time 14.44
INSTRUM spect
PROBHD 5 mm DUL 13C-1
PULPROG zg30
TD 32768
SOLVENT CDCl3
NS 52
DS 0
SWH 6410.256 Hz
FIDRES 0.195625 Hz
AQ 2.5559540 sec
RG 4
DW 78.000 usec
DE 6.00 usec
TE 300.0 K
D1 2.00000000 sec
TD0 1
======== CHANNEL f1 ========
NUC1 1H
P1 10.00 usec
PL1 -2.40 dB
SFO1 400.1528010 MHz
SI 16384
SF 400.1500092 MHz
WDW EM
SSB 0
LB 0.00 Hz
GB 0
PC 1.00
(R)-12c
ppm

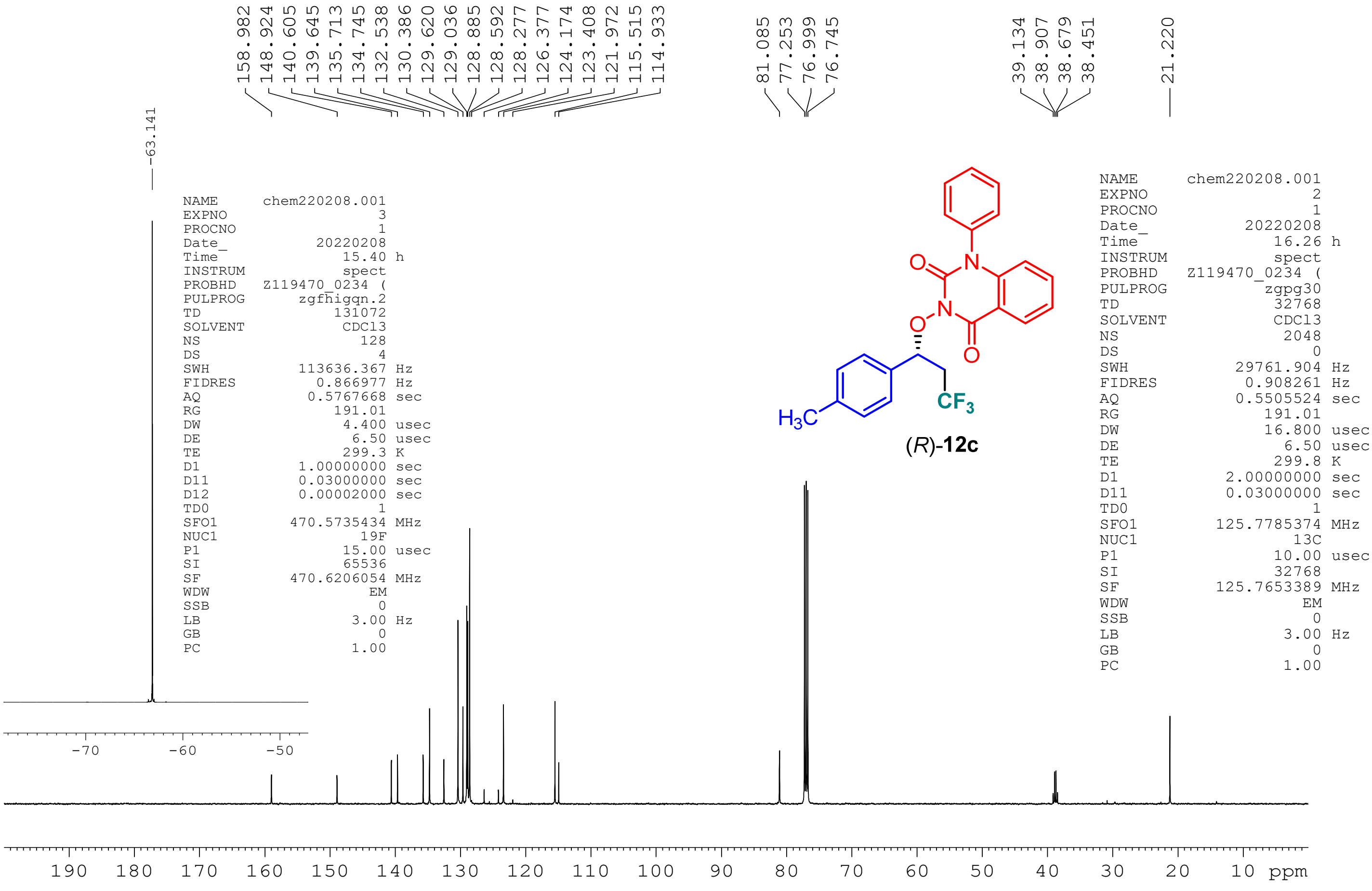
NAME chem220208.001
EXPNO 3
PROCNO 1
Date_ 20220208
Time 15.40 h
INSTRUM spect
PROBHD Z119470_0234 (
PULPROG zgfhigqn.2
TD 131072
SOLVENT CDCl3
NS 128
DS 4
SWH 113636.367 Hz
FIDRES 0.866977 Hz
AQ 0.5767668 sec
RG 191.01
DW 4.400 usec
DE 6.50 usec
TE 299.3 K
D1 1.00000000 sec
D11 0.03000000 sec
D12 0.00002000 sec
TD0 1
SFO1 470.5735434 MHz
NUC1 19F
P1 15.00 usec
SI 65536
SF 470.6206054 MHz
WDW EM
SSB 0
LB 3.00 Hz
GB 0
PC 1.00
-63.141
NAME chem220208.001
EXPNO 2
PROCNO 1
Date_ 20220208
Time 16.26 h
INSTRUM spect
PROBHD Z119470_0234 (
PULPROG zgpg30
TD 32768
SOLVENT CDCl3
NS 2048
DS 0
SWH 29761.904 Hz
FIDRES 0.908261 Hz
AQ 0.5505524 sec
RG 191.01
DW 16.800 usec
DE 6.50 usec
TE 299.8 K
D1 2.00000000 sec
D11 0.03000000 sec
TD0 1
SFO1 125.7785374 MHz
NUC1 13C
P1 10.00 usec
SI 32768
SF 125.7653389 MHz
WDW EM
SSB 0
LB 3.00 Hz
GB 0
PC 1.00
(R)-12c
ppm

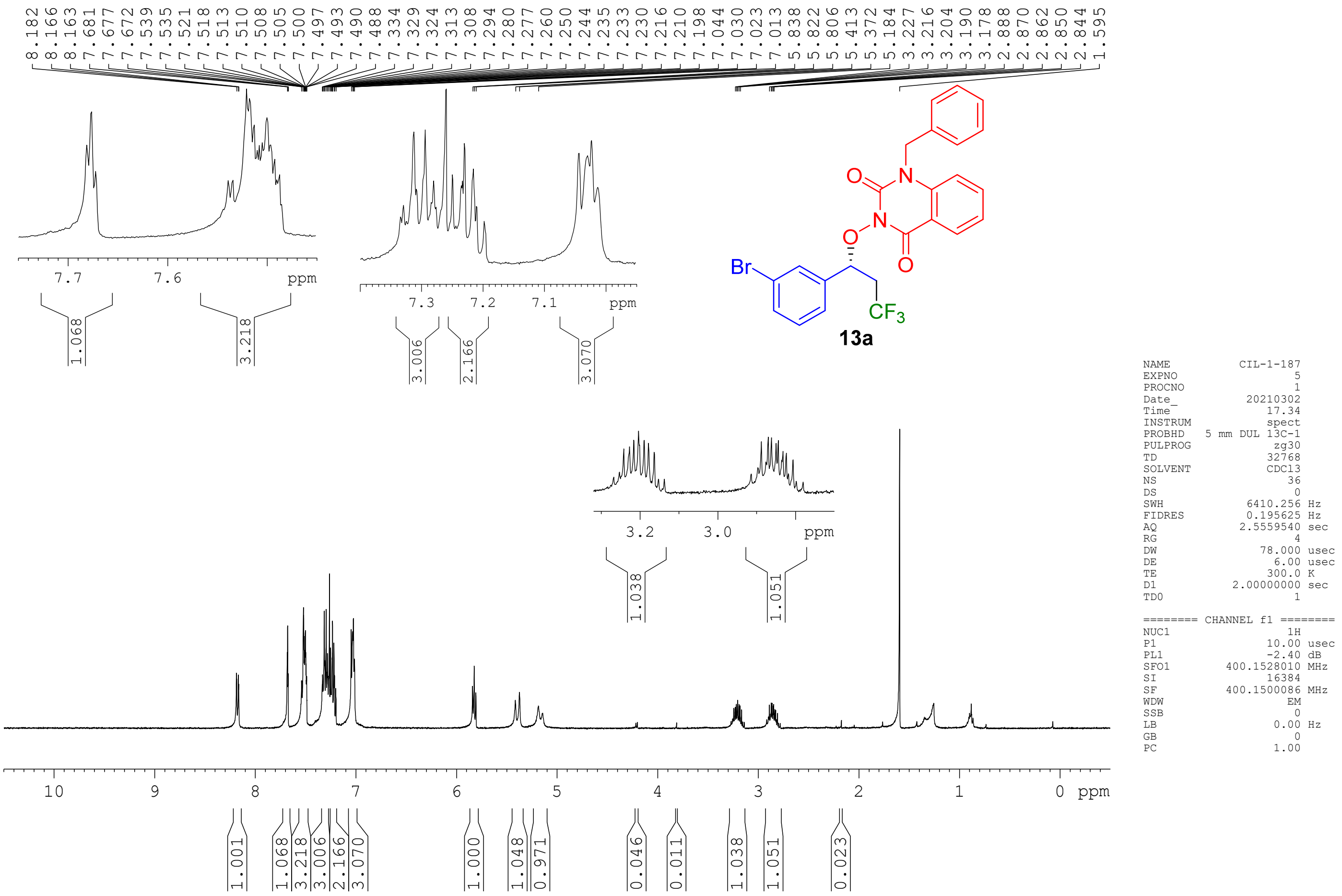
8.182 8.166 8.163 7.681 7.677 7.672 7.539 7.535 7.521 7.518 7.513 7.510 7.508 7.505 7.500 7.497 7.493 7.490 7.488 7.334 7.329 7.324 7.313 7.308 7.294 7.290 7.280 7.277 7.260 7.250 7.244 7.235 7.233 7.230 7.216 7.210 7.198 7.044 7.030 7.023 7.013 5.838 5.822 5.806 5.413 5.372 5.184 3.227 3.216 3.204 3.190 3.178 2.888 2.870 2.862 2.850 2.844 1.595
1.001 1.068 3.218 3.006 2.166 3.070 1.000 1.048 0.971 0.046 0.011 1.038 1.051 0.023
Br
CF3
13a
NAME CIL-1-187
EXPNO 5
PROCNO 1
Date_ 20210302
Time 17.34
INSTRUM spect
PROBHD 5 mm DUL 13C-1
PULPROG zg30
TD 32768
SOLVENT CDCl3
NS 36
DS 0
SWH 6410.256 Hz
FIDRES 0.195625 Hz
AQ 2.5559540 sec
RG 4
DW 78.000 usec
DE 6.00 usec
TE 300.0 K
D1 2.00000000 sec
TD0 1
======== CHANNEL f1 ========
NUC1 1H
P1 10.00 usec
PL1 -2.40 dB
SFO1 400.1528010 MHz
SI 16384
SF 400.1500086 MHz
WDW EM
SSB 0
LB 0.00 Hz
GB 0
PC 1.00

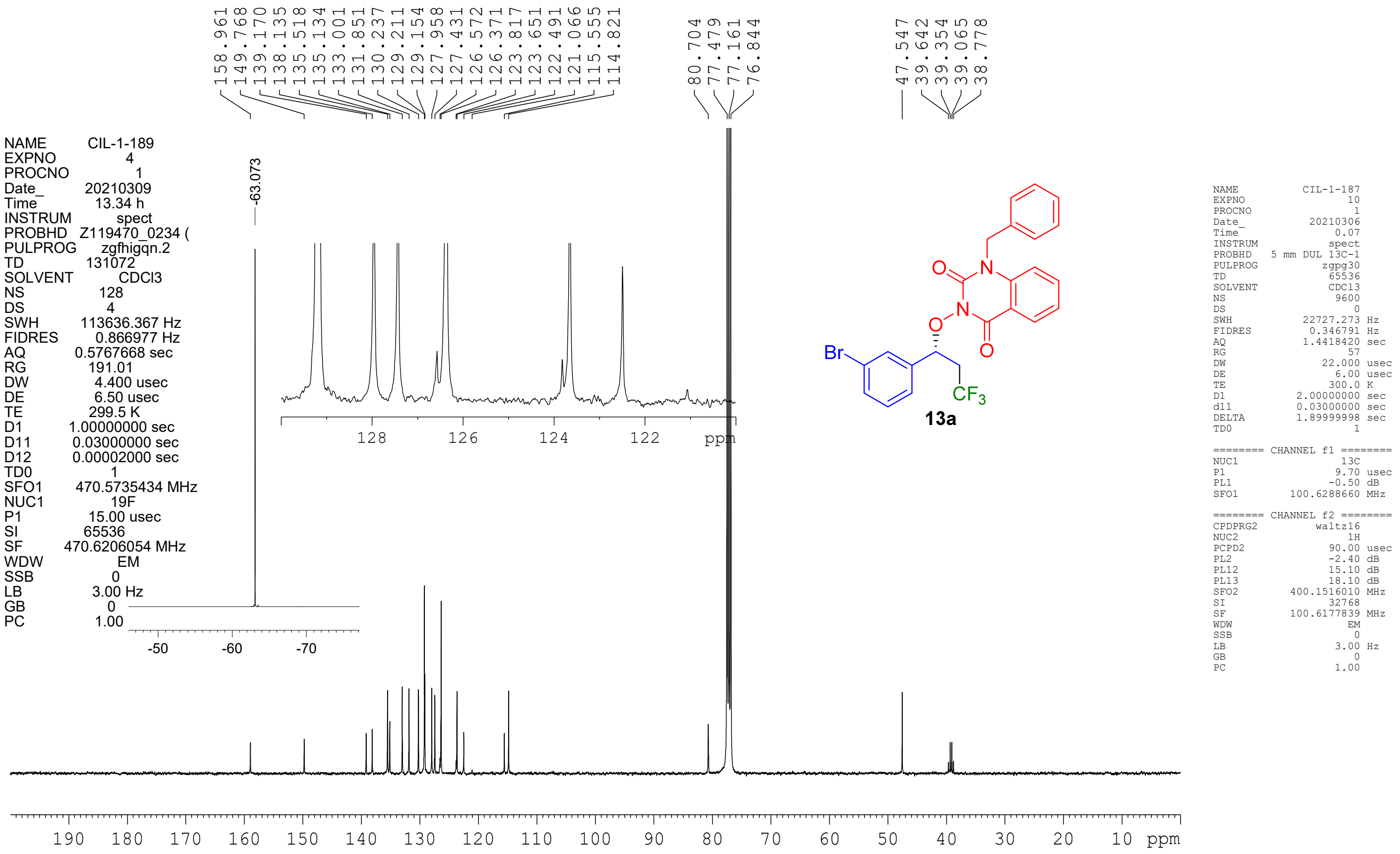
158.961 149.768 139.170 138.135 135.518 135.134 133.001 131.851 130.237 129.211 129.154 127.958 127.431 126.572 126.371 123.817 123.651 122.491 121.066 115.555 114.821 80.704 77.479 77.161 76.844 47.547 39.642 39.354 39.065 38.778
-63.073
NAME CIL-1-189
EXPNO 4
PROCNO 1
Date_ 20210309
Time 13.34 h
INSTRUM spect
PROBHD Z119470_0234 (
PULPROG zgfhigqn.2
TD 131072
SOLVENT CDCl3
NS 128
DS 4
SWH 113636.367 Hz
FIDRES 0.866977 Hz
AQ 0.5767668 sec
RG 191.01
DW 4.400 usec
DE 6.50 usec
TE 299.5 K
D1 1.00000000 sec
D11 0.03000000 sec
D12 0.00002000 sec
TD0 1
SFO1 470.5735434 MHz
NUC1 19F
P1 15.00 usec
SI 65536
SF 470.6206054 MHz
WDW EM
SSB 0
LB 3.00 Hz
GB 0
PC 1.00
Br
CF3
13a
NAME CIL-1-187
EXPNO 10
PROCNO 1
Date_ 20210306
Time 0.07
INSTRUM spect
PROBHD 5 mm DUL 13C-1
PULPROG zgpg30
TD 65536
SOLVENT CDCl3
NS 9600
DS 0
SWH 22727.273 Hz
FIDRES 0.346791 Hz
AQ 1.4418420 sec
RG 57
DW 22.000 usec
DE 6.00 usec
TE 300.0 K
D1 2.00000000 sec
d11 0.03000000 sec
DELTA 1.89999998 sec
TD0 1
======== CHANNEL f1 ========
NUC1 13C
P1 9.70 usec
PL1 -0.50 dB
SFO1 100.6288660 MHz
======== CHANNEL f2 ========
CPDPRG2 waltz16
NUC2 1H
PCPD2 90.00 usec
PL2 -2.40 dB
PL12 15.10 dB
PL13 18.10 dB
SFO2 400.1516010 MHz
SI 32768
SF 100.6177839 MHz
WDW EM
SSB 0
LB 3.00 Hz
GB 0
PC 1.00

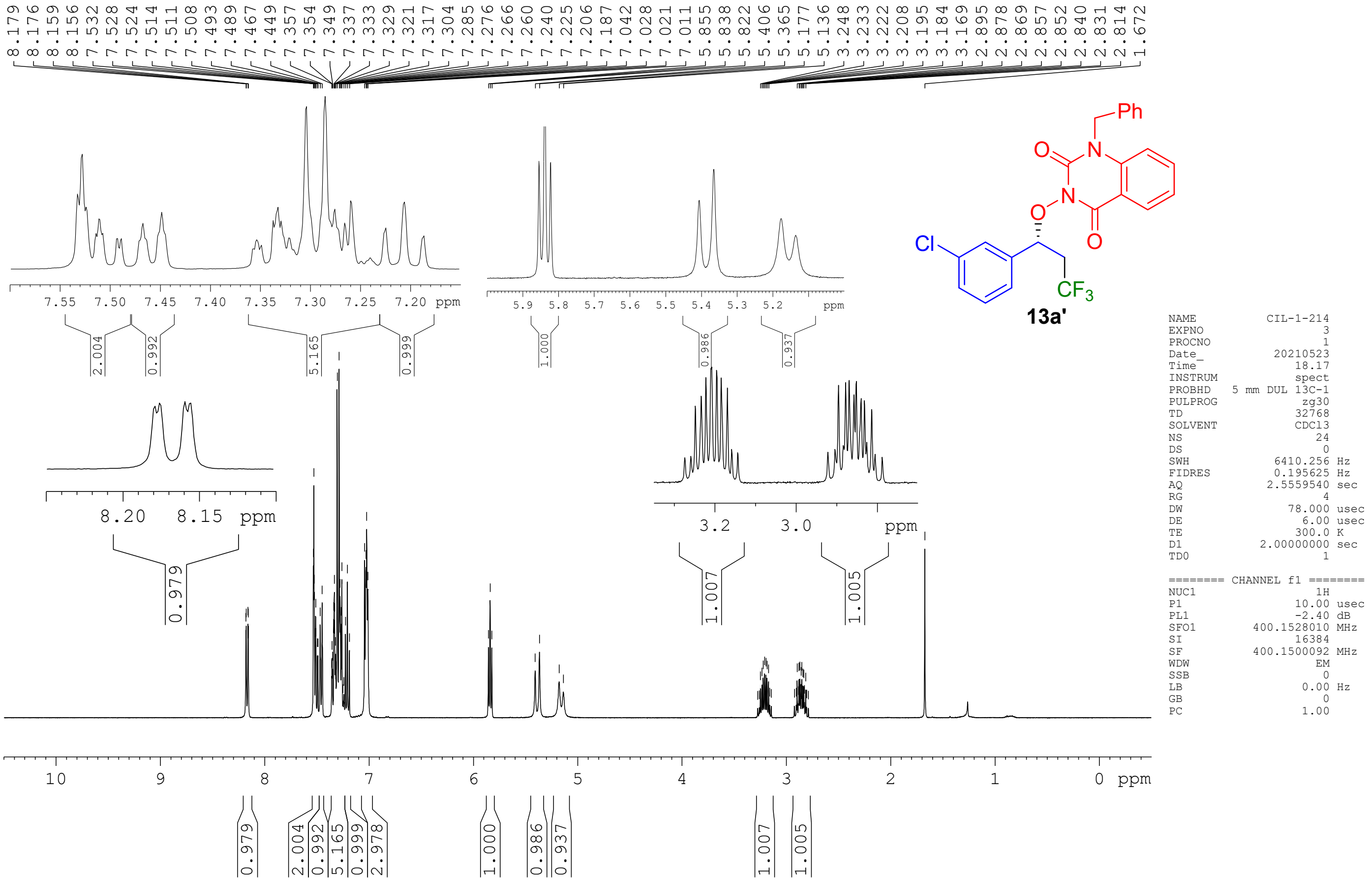
8.179
8.176
8.159
8.156
7.532
7.528
7.524
7.514
7.511
7.508
7.493
7.489
7.467
7.449
7.357
7.354
7.349
7.337
7.333
7.329
7.321
7.317
7.304
7.285
7.276
7.266
7.260
7.240
7.225
7.206
7.187
7.042
7.028
7.021
7.011
5.855
5.838
5.822
5.406
5.365
5.177
5.136
3.248
3.233
3.222
3.208
3.195
3.184
3.169
2.895
2.878
2.869
2.857
2.852
2.840
2.831
2.814
1.672
Ph
O
N
N
O
O
Cl
CF3
13a'
NAME CIL-1-214
EXPNO 3
PROCNO 1
Date_ 20210523
Time 18.17
INSTRUM spect
PROBHD 5 mm DUL 13C-1
PULPROG zg30
TD 32768
SOLVENT CDCl3
NS 24
DS 0
SWH 6410.256 Hz
FIDRES 0.195625 Hz
AQ 2.5559540 sec
RG 4
DW 78.000 usec
DE 6.00 usec
TE 300.0 K
D1 2.00000000 sec
TD0 1
======== CHANNEL f1 ========
NUC1 1H
P1 10.00 usec
PL1 -2.40 dB
SFO1 400.1528010 MHz
SI 16384
SF 400.1500092 MHz
WDW EM
SSB 0
LB 0.00 Hz
GB 0
PC 1.00
0.979
2.004
0.992
5.165
0.999
2.978
1.000
0.986
0.937
1.007
1.005
ppm

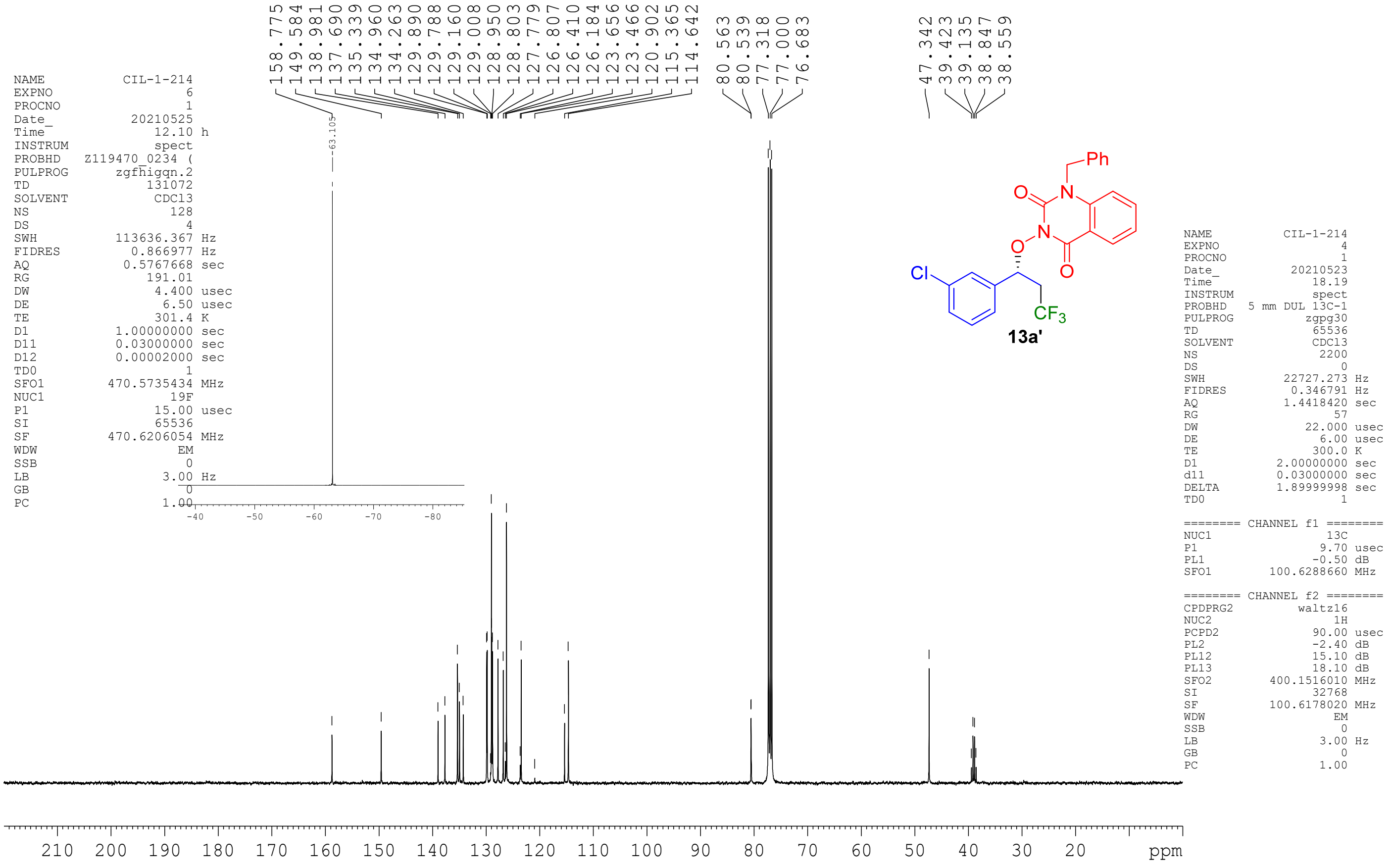
158.775
149.584
138.981
137.690
135.339
134.960
134.263
129.890
129.788
129.160
129.008
128.950
128.803
127.779
126.807
126.410
126.184
123.656
123.466
120.902
115.365
114.642
80.563
80.539
77.318
77.000
76.683
47.342
39.423
39.135
38.847
38.559
-63.105
NAME CIL-1-214
EXPNO 6
PROCNO 1
Date_ 20210525
Time 12.10 h
INSTRUM spect
PROBHD Z119470_0234 (
PULPROG zgfhigqn.2
TD 131072
SOLVENT CDCl3
NS 128
DS 4
SWH 113636.367 Hz
FIDRES 0.866977 Hz
AQ 0.5767668 sec
RG 191.01
DW 4.400 usec
DE 6.50 usec
TE 301.4 K
D1 1.00000000 sec
D11 0.03000000 sec
D12 0.00002000 sec
TD0 1
SFO1 470.5735434 MHz
NUC1 19F
P1 15.00 usec
SI 65536
SF 470.6206054 MHz
WDW EM
SSB 0
LB 3.00 Hz
GB 0
PC 1.00
Ph
O
N
N
O
O
Cl
CF3
13a'
NAME CIL-1-214
EXPNO 4
PROCNO 1
Date_ 20210523
Time 18.19
INSTRUM spect
PROBHD 5 mm DUL 13C-1
PULPROG zgpg30
TD 65536
SOLVENT CDCl3
NS 2200
DS 0
SWH 22727.273 Hz
FIDRES 0.346791 Hz
AQ 1.4418420 sec
RG 57
DW 22.000 usec
DE 6.00 usec
TE 300.0 K
D1 2.00000000 sec
d11 0.03000000 sec
DELTA 1.89999998 sec
TD0 1
======== CHANNEL f1 ========
NUC1 13C
P1 9.70 usec
PL1 -0.50 dB
SFO1 100.6288660 MHz
======== CHANNEL f2 ========
CPDPRG2 waltz16
NUC2 1H
PCPD2 90.00 usec
PL2 -2.40 dB
PL12 15.10 dB
PL13 18.10 dB
SFO2 400.1516010 MHz
SI 32768
SF 100.6178020 MHz
WDW EM
SSB 0
LB 3.00 Hz
GB 0
PC 1.00
ppm

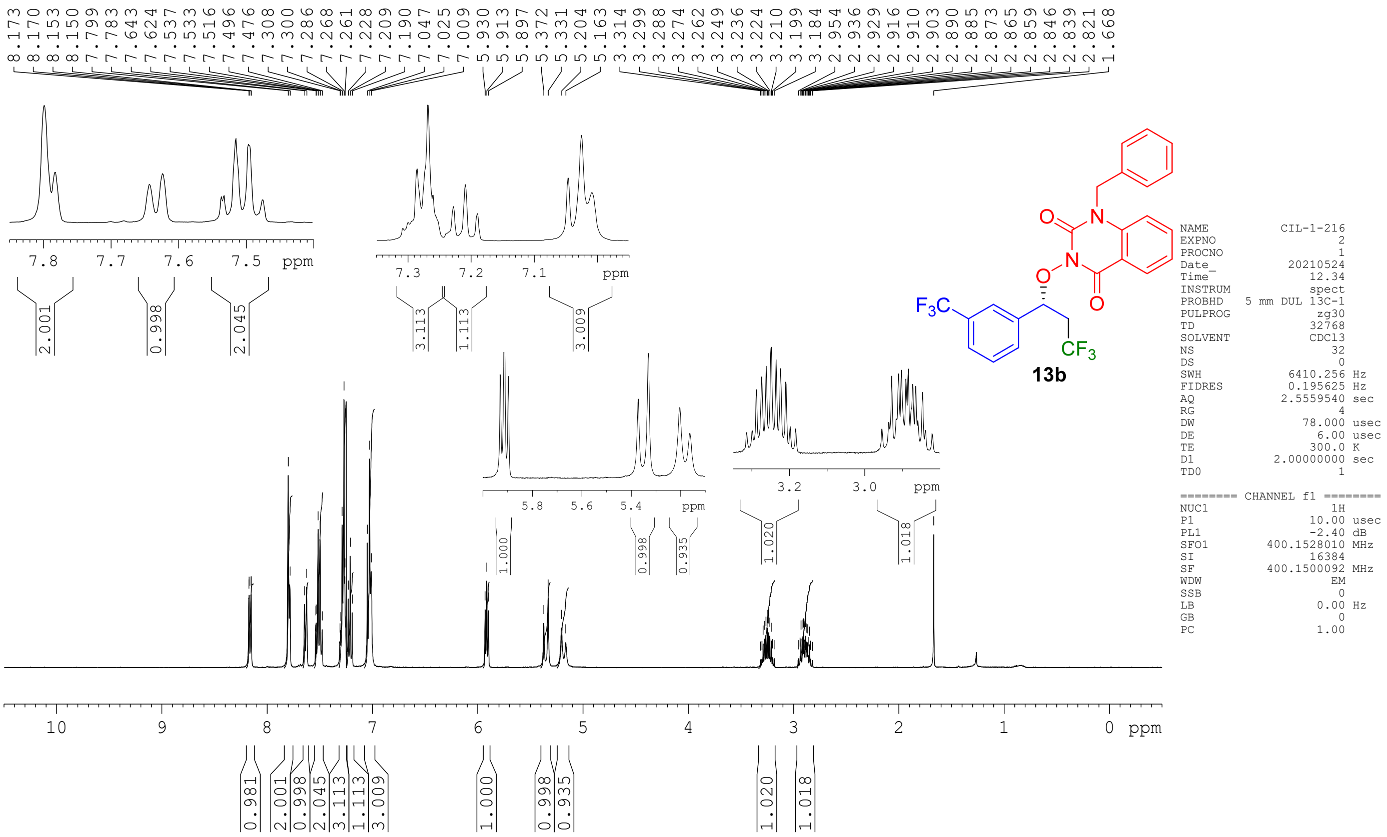

8.173 8.170 8.153 8.150 7.799 7.783 7.643 7.624 7.537 7.533 7.516 7.496 7.476 7.308 7.300 7.286 7.268 7.261 7.228 7.209 7.190 7.047 7.025 7.009 5.930 5.913 5.897 5.372 5.331 5.204 5.163 3.314 3.299 3.288 3.274 3.262 3.249 3.236 3.224 3.210 3.199 3.184 2.954 2.936 2.929 2.916 2.910 2.903 2.890 2.885 2.873 2.865 2.859 2.846 2.839 2.821 1.668
13b
NAME CIL-1-216
EXPNO 2
PROCNO 1
Date_ 20210524
Time 12.34
INSTRUM spect
PROBHD 5 mm DUL 13C-1
PULPROG zg30
TD 32768
SOLVENT CDCl3
NS 32
DS 0
SWH 6410.256 Hz
FIDRES 0.195625 Hz
AQ 2.5559540 sec
RG 4
DW 78.000 usec
DE 6.00 usec
TE 300.0 K
D1 2.00000000 sec
TD0 1
======== CHANNEL f1 ========
NUC1 1H
P1 10.00 usec
PL1 -2.40 dB
SFO1 400.1528010 MHz
SI 16384
SF 400.1500092 MHz
WDW EM
SSB 0
LB 0.00 Hz
GB 0
PC 1.00
10 9 8 7 6 5 4 3 2 1 0 ppm
0.981 2.001 0.998 2.045 3.113 1.113 3.009 1.000 0.998 0.935 1.020 1.018

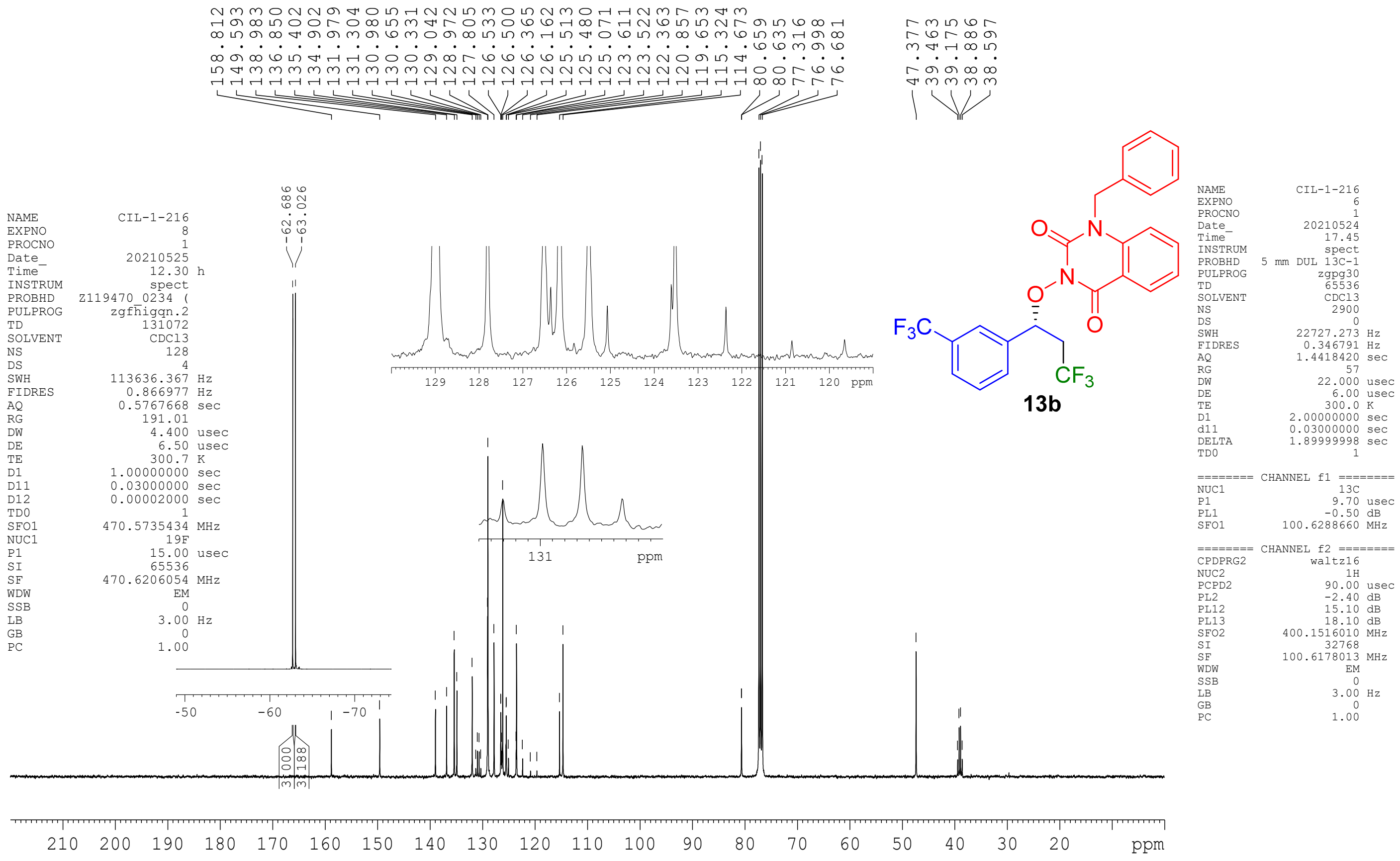

158.812 149.593 138.983 136.850 135.402 134.902 131.979 131.304 130.980 130.655 130.331 129.042 128.972 127.805 126.533 126.500 126.365 126.162 125.513 125.480 125.071 123.611 123.522 122.363 120.857 119.653 115.324 114.673 80.659 80.635 77.316 76.998 76.681 47.377 39.463 39.175 38.886 38.597
-62.686 -63.026
NAME CIL-1-216
EXPNO 8
PROCNO 1
Date_ 20210525
Time 12.30 h
INSTRUM spect
PROBHD Z119470_0234 (
PULPROG zgfhigqn.2
TD 131072
SOLVENT CDCl3
NS 128
DS 4
SWH 113636.367 Hz
FIDRES 0.866977 Hz
AQ 0.5767668 sec
RG 191.01
DW 4.400 usec
DE 6.50 usec
TE 300.7 K
D1 1.00000000 sec
D11 0.03000000 sec
D12 0.00002000 sec
TD0 1
SFO1 470.5735434 MHz
NUC1 19F
P1 15.00 usec
SI 65536
SF 470.6206054 MHz
WDW EM
SSB 0
LB 3.00 Hz
GB 0
PC 1.00
13b
NAME CIL-1-216
EXPNO 6
PROCNO 1
Date_ 20210524
Time 17.45
INSTRUM spect
PROBHD 5 mm DUL 13C-1
PULPROG zgpg30
TD 65536
SOLVENT CDCl3
NS 2900
DS 0
SWH 22727.273 Hz
FIDRES 0.346791 Hz
AQ 1.4418420 sec
RG 57
DW 22.000 usec
DE 6.00 usec
TE 300.0 K
D1 2.00000000 sec
d11 0.03000000 sec
DELTA 1.89999998 sec
TD0 1
======== CHANNEL f1 ========
NUC1 13C
P1 9.70 usec
PL1 -0.50 dB
SFO1 100.6288660 MHz
======== CHANNEL f2 ========
CPDPRG2 waltz16
NUC2 1H
PCPD2 90.00 usec
PL2 -2.40 dB
PL12 15.10 dB
PL13 18.10 dB
SFO2 400.1516010 MHz
SI 32768
SF 100.6178013 MHz
WDW EM
SSB 0
LB 3.00 Hz
GB 0
PC 1.00
210 200 190 180 170 160 150 140 130 120 110 100 90 80 70 60 50 40 30 20 ppm

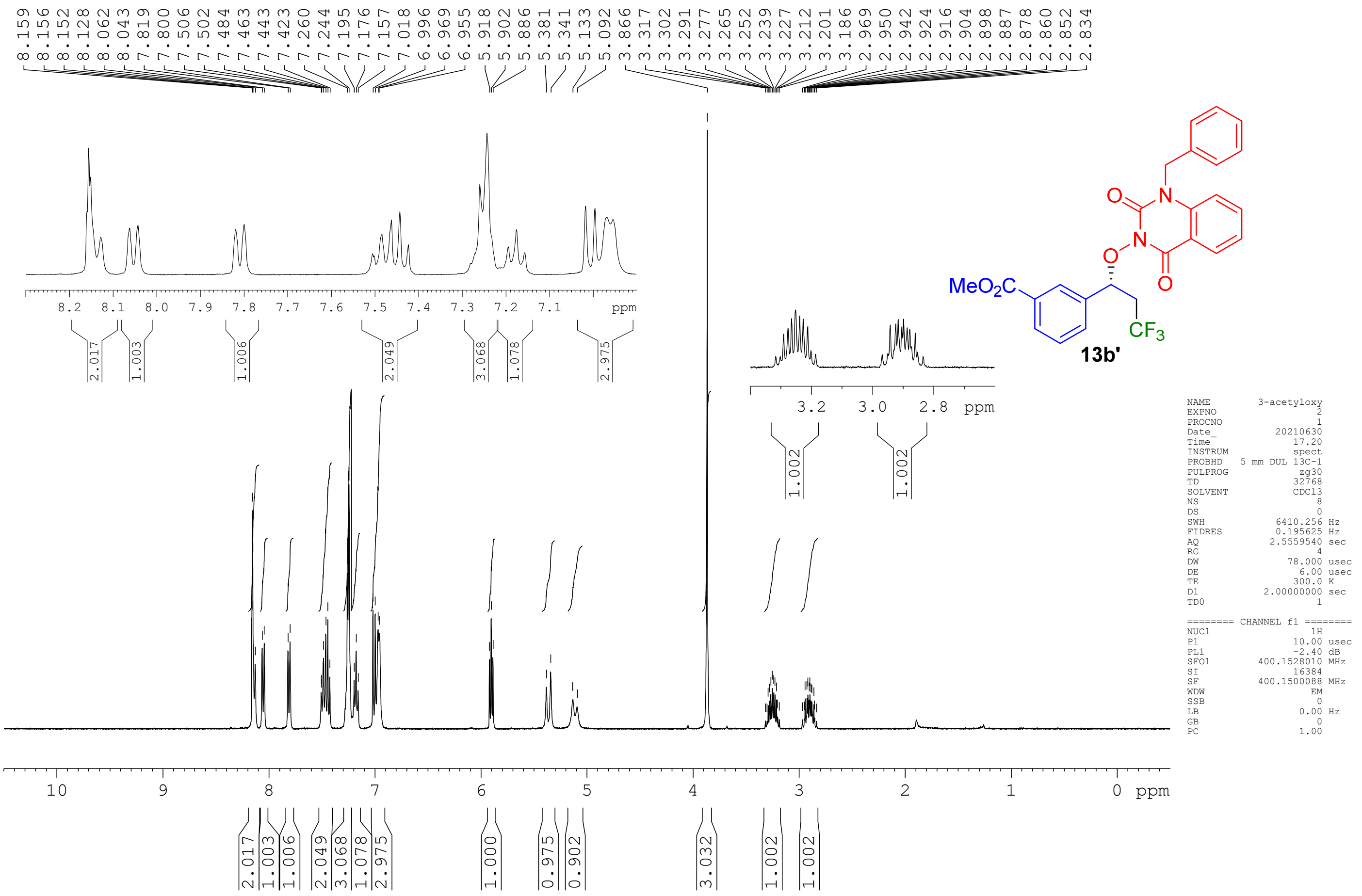
8.159 8.156 8.152 8.128 8.062 8.043 7.819 7.800 7.506 7.502 7.484 7.463 7.443 7.423 7.260 7.244 7.195 7.176 7.157 7.018 6.996 6.969 6.955 5.918 5.902 5.886 5.381 5.341 5.133 5.092 3.866 3.317 3.302 3.291 3.277 3.265 3.252 3.239 3.227 3.212 3.201 3.186 2.969 2.950 2.942 2.924 2.916 2.904 2.898 2.887 2.878 2.860 2.852 2.834
MeO2C
CF3
13b'
2.017 1.003 1.006 2.049 3.068 1.078 2.975 1.000 0.975 0.902 3.032 1.002 1.002
ppm

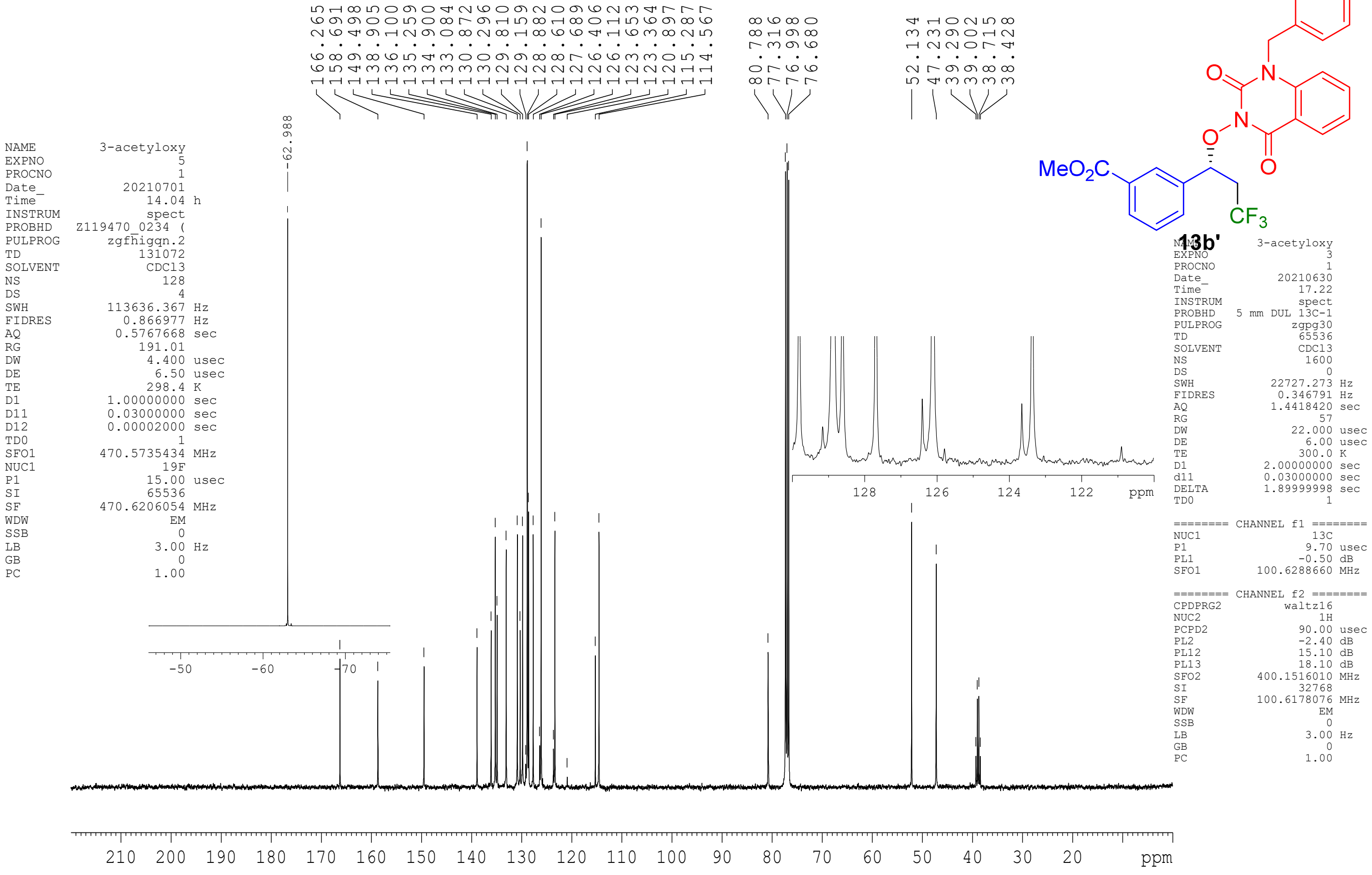
166.265 158.691 149.498 138.905 136.100 135.259 134.900 133.084 130.872 130.296 129.810 129.159 128.882 128.610 127.689 126.406 126.112 123.653 123.364 120.897 115.287 114.567 80.788 77.316 76.998 76.680 52.134 47.231 39.290 39.002 38.715 38.428
-62.988
MeO2C
CF3
13b'
ppm

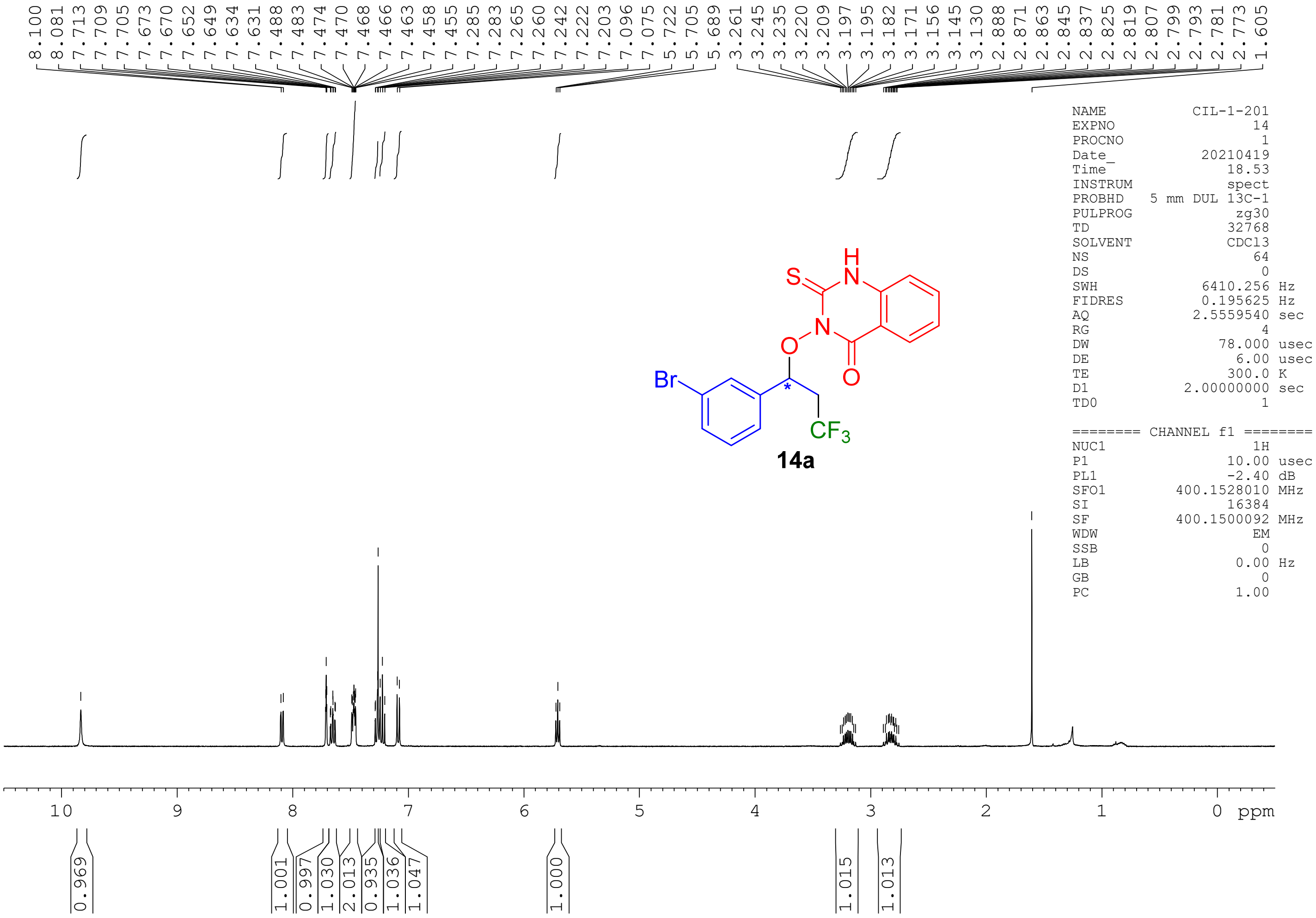
8.100 8.081 7.713 7.709 7.705 7.673 7.670 7.652 7.649 7.634 7.631 7.488 7.483 7.474 7.470 7.468 7.466 7.463 7.458 7.455 7.285 7.283 7.265 7.260 7.242 7.222 7.203 7.096 7.075 5.722 5.705 5.689 3.261 3.245 3.235 3.220 3.209 3.197 3.195 3.182 3.171 3.156 3.145 3.130 2.888 2.871 2.863 2.845 2.837 2.825 2.819 2.807 2.799 2.793 2.781 2.773 1.605
NAME CIL-1-201
EXPNO 14
PROCNO 1
Date_ 20210419
Time 18.53
INSTRUM spect
PROBHD 5 mm DUL 13C-1
PULPROG zg30
TD 32768
SOLVENT CDCl3
NS 64
DS 0
SWH 6410.256 Hz
FIDRES 0.195625 Hz
AQ 2.5559540 sec
RG 4
DW 78.000 usec
DE 6.00 usec
TE 300.0 K
D1 2.00000000 sec
TD0 1
======== CHANNEL f1 ========
NUC1 1H
P1 10.00 usec
PL1 -2.40 dB
SFO1 400.1528010 MHz
SI 16384
SF 400.1500092 MHz
WDW EM
SSB 0
LB 0.00 Hz
GB 0
PC 1.00
14a
10 9 8 7 6 5 4 3 2 1 0 ppm
0.969 1.001 0.997 1.030 2.013 0.935 1.036 1.047 1.000 1.015 1.013

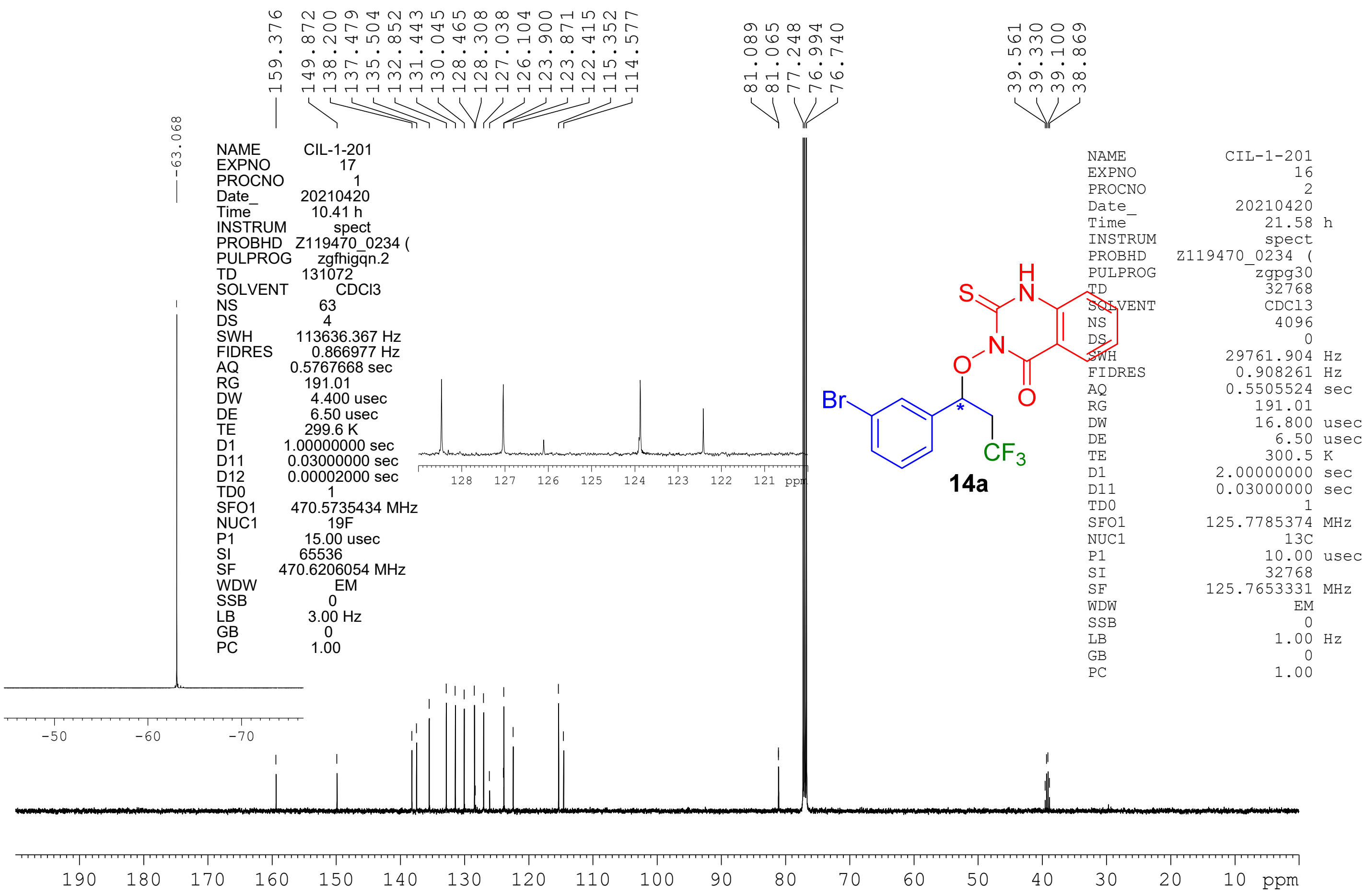
159.376 149.872 138.200 137.479 135.504 132.852 131.443 130.045 128.465 128.308 127.038 126.104 123.900 123.871 122.415 115.352 114.577 81.089 81.065 77.248 76.994 76.740 39.561 39.330 39.100 38.869
-63.068
NAME CIL-1-201
EXPNO 17
PROCNO 1
Date_ 20210420
Time 10.41 h
INSTRUM spect
PROBHD Z119470_0234 (
PULPROG zgfhigqn.2
TD 131072
SOLVENT CDCl3
NS 63
DS 4
SWH 113636.367 Hz
FIDRES 0.866977 Hz
AQ 0.5767668 sec
RG 191.01
DW 4.400 usec
DE 6.50 usec
TE 299.6 K
D1 1.00000000 sec
D11 0.03000000 sec
D12 0.00002000 sec
TD0 1
SFO1 470.5735434 MHz
NUC1 19F
P1 15.00 usec
SI 65536
SF 470.6206054 MHz
WDW EM
SSB 0
LB 3.00 Hz
GB 0
PC 1.00
-50 -60 -70
128 127 126 125 124 123 122 121 ppm
NAME CIL-1-201
EXPNO 16
PROCNO 2
Date_ 20210420
Time 21.58 h
INSTRUM spect
PROBHD Z119470_0234 (
PULPROG zgpg30
TD 32768
SOLVENT CDCl3
NS 4096
DS 0
SWH 29761.904 Hz
FIDRES 0.908261 Hz
AQ 0.5505524 sec
RG 191.01
DW 16.800 usec
DE 6.50 usec
TE 300.5 K
D1 2.00000000 sec
D11 0.03000000 sec
TD0 1
SFO1 125.7785374 MHz
NUC1 13C
P1 10.00 usec
SI 32768
SF 125.7653331 MHz
WDW EM
SSB 0
LB 1.00 Hz
GB 0
PC 1.00
14a
190 180 170 160 150 140 130 120 110 100 90 80 70 60 50 40 30 20 10 ppm
